# Three-Component Synthesis of 2-Amino-3-cyano-4*H*-chromenes, In Silico Analysis of Their Pharmacological Profile, and In Vitro Anticancer and Antifungal Testing

**DOI:** 10.3390/ph14111110

**Published:** 2021-10-30

**Authors:** Alberto Feliciano, Omar Gómez-García, Carlos H. Escalante, Mario A. Rodríguez-Hernández, Mariana Vargas-Fuentes, Dulce Andrade-Pavón, Lourdes Villa-Tanaca, Cecilio Álvarez-Toledano, María Teresa Ramírez-Apan, Miguel A. Vázquez, Joaquín Tamariz, Francisco Delgado

**Affiliations:** 1 Departamento de Química Orgánica, Escuela Nacional de Ciencias Biológicas, Instituto Politécnico Nacional, Prolongación de Carpio y Plan de Ayala S/N, Mexico City 11340, Mexico; a.feliciano@ugto.mx (A.F.); escalantecah@gmail.com (C.H.E.); mario.arctic.505@gmail.com (M.A.R.-H.); marianavargasme@gmail.com (M.V.-F.); jtamarizm@yahoo.com.mx (J.T.); 2 Departamento de Química, Universidad de Guanajuato, Noria Alta S/N, Guanajuato 36050, Mexico; mvazquez@ugto.mx; 3 Laboratorio de Biología Molecular de Bacterias y Levaduras, Departamento de Microbiología, Escuela Nacional de Ciencias Biológicas, Instituto Politécnico Nacional, Prolongación de Carpio y Plan de Ayala S/N, Mexico City 11340, Mexico; andrade_eclud88@hotmail.com (D.A.-P.); mvillat@ipn.mx (L.V.-T.); 4 Departamento de Fisiología, Escuela Nacional de Ciencias Biológicas, Instituto Politécnico Nacional, Av. Wilfrido Massieu S/N, Mexico City 07738, Mexico; 5 Instituto de Química-UNAM, Circuito Exterior, Ciudad Universitaria, Coyoacán, C.P., Mexico City 04510, Mexico; cecilio@unam.mx (C.Á.-T.); mtrapan@unam.mx (M.T.R.-A.)

**Keywords:** 2-amino-3-cyano-4*H*-chromenes, cytotoxicity, topoisomerase I, antifungal activity, CYP51, *Candida* spp.

## Abstract

Chromenes are compounds that may be useful for inhibiting topoisomerase and cytochrome, enzymes involved in the growth of cancer and fungal cells, respectively. The aim of this study was to synthesize a series of some novel 2-amino-3-cyano-4-aryl-6,7-methylendioxy-4*H*-chromenes **4a–o** and 2-amino-3-cyano-5,7-dimethoxy-4-aryl-4*H*-chromenes **6a–h** by a three-component reaction, and test these derivatives for anticancer and antifungal activity. Compounds **4a** and **4b** were more active than cisplatin (**9**) and topotecan (**7**) in SK-LU-1 cells, and more active than **9** in PC-3 cells. An evaluation was also made of the series of compounds **4** and **6** as potential antifungal agents against six *Candida* strains, finding their MIC_50_ to be less than or equal to that of fluconazole (**8**). Molecular docking studies are herein reported, for the interaction of **4** and **6** with topoisomerase IB and the active site of CYP51 of *Candida* spp. Compounds **4a–o** and **6a–h** interacted in a similar way as **7** with key amino acids of the active site of topoisomerase IB and showed better binding energy than **8** at the active site of CYP51. Hence, **4a–o** and **6a–h** are good candidates for further research, having demonstrated their dual inhibition of enzymes that participate in the growth of cancer and fungal cells.

## 1. Introduction

Heterocyclic systems, in particular oxygen-containing structures, constitute a privileged group of compounds based on their wide range of biological activity. Derivatives of 4*H*-chromenes have attracted attention because of containing a pharmacophore group that is found in many molecules with pharmacological effects, including anticancer [1], antifungal [2], antibacterial, antioxidant [3], and antiprotozoal [4] agents. They show promise as inhibitors of topoisomerase and lanosterol 14α-demethylase, enzymes linked to the growth of cancer and fungal cells, respectively.

Cancer is a serious health problem, placed as the second leading cause of death worldwide [5,6]. It is characterized by the presence of a group of cells that multiply uncontrollably and autonomously, invading neighboring and remote organs. This disease is engendered by external factors (chemicals, tobacco, infectious organisms, and radiation) and internal conditions (immune problems, inherited mutations, hormones, and mutations generated by metabolism). It is most commonly manifested in the prostate, lungs, breasts, colon, liver, bronchi, uterine corpus, stomach, and blood (leukemia), according to a study in the United States in 2016 [7].

The most commonly used cancer treatments are surgery, radiation, chemotherapy, hormonal therapy, and targeted therapy. Several enzymes involved in the pathology of different carcinomas are considered important therapeutic targets, including topoisomerases I and II [8,9], aurora kinase A [10], CDKs [11], tubulin [12], telomerase [13], and caspase [14]. For instance, topoisomerases break and reattach the DNA strands needed by cells for growth. Consequently, their inhibition can destroy cancer cells [8]. Topotecan (**7**), a selective topoisomerase I inhibitor, is the first line drug in the treatment of some types of cancer (e.g., ovarian and lung). Since this drug is costly and generates many side effects, it is necessary to develop new specific topoisomerase I inhibitors with a better pharmacological profile and lower toxicity for healthy cells.

Mycoses, a group of infectious diseases produced by pathogenic mycelia and unicellular fungi (yeasts), have diverse clinical manifestations, and represent an increasingly difficult medical challenge for two main reasons. Firstly, fungi are ubiquitous microorganisms in nature, widely distributed in the environment, and impossible to eradicate. Secondly, drug-resistant pathogenic strains are the cause of a growing number of hospital infections in immunocompromised patients.

One target for treating fungal infections is lanosterol 14α-demethylase, an enzyme of the cytochrome P450 family (CYP51) [15]. It participates in the biosynthesis of sterols, including the main sterol, ergosterol, in the yeast membrane. Indeed, the inhibition of CYP51 in yeasts is the mechanism of action of azoles (fluconazole (**8**) and itraconazole), the most widely used drugs in antifungal therapy. Unfortunately, the number of *Candida* yeast strains developing resistance mechanisms against these and other antifungal drugs is on the rise [16], creating the need for the discovery of new antifungal agents [17].

Chromene derivatives have demonstrated antifungal activity in vitro against several kind of pathogenic fungi, such as *Cryptococcus neoformans*, *Torulopsis glabrata*, *Trichosporon cutaneum*, *Candida albicans*, *C. parapsilosis* and *C. tropicalis* [18]. Moreover, there are descriptions in the literature of 4*H*-chromenes as specific inhibitors of enzymes involved in tumor growth, including topoisomerases I and II [19,20], tubulin [21], caspase [22], COT kinase [23], 17β-dehydrogenase-4 (HSD17B4) [24], telomerase [25], and tyrosinase [26]. However, few reports exist on topoisomerase IB, and no molecular docking studies have yet been carried out, to our knowledge, for 2-amino-3-cyano-4*H*-chromenes at the active site of this enzyme.

Among the different approaches for synthesizing 4*H*-chromenes are multicomponent reactions [27,28] assisted by microwave irradiation [29], sonication [30], electrocatalysis [31], mechanochemistry [32], and infrared radiation (IR) [33]. They can also be obtained by cyclocondensation reactions [34] with heterogeneous phase catalysts [35], through the oxidation of 2*H*-chromenes and dihydro-1-benzoxepines with hypervalent iodine [36]. Additionally, the formation of these compounds is promoted by potassium carbonate [4], triethylamine [4], silica-supported catalysts [37], cyclodextrins [38], magnetic nanoparticles [39], including the use of ionic liquids [40]. 

Here, two homologous series of 2-amino-3-cyano-4*H*-chromenes, **4a–o** and **6a–h**, were synthesized by a three-component reaction under mild conditions. They were analyzed in silico to determine their pharmacological profile and toxicity, and to observe molecular docking on the active site of two enzymes: CYP51 of *Candida* spp. and topoisomerase IBJK. The compounds were tested in vitro for possible cytotoxicity against human prostate (PC-3) and lung (SK-LU-1) cancer cell lines, with topotecan (**7**) as the reference drug, and for antifungal activity against six species of *Candida* spp., with fluconazole (**8**) as the reference. The results showed that 2-amino-3-cyano-4*H*-chromenes, **4a–o** and **6a–h** exhibited a high anticancer activity in human prostate (PC-3) and lung (SK-LU-1) cancer cell lines, as well as a better antifungal activity in the six species of *Candida* genus with respect to the reference drugs (Topotecan and Fluconazole). Likewise, the molecular coupling studies revealed that both series of 2-amino-3-cyano-4*H*-chromenes, **4a–o** and **6a–h**, recognize the active site of the enzymes: DNA Topoisomerase IBJK and CYP51 of *Candida* spp. Such findings suggest that the compounds proposed here could be considered in the future as alternatives in anticancer and antifungal therapy.

## 2. Materials and Methods

### 2.1. Chemicals and Instruments

The synthesized compounds were purified by flash column chromatography with silica gel (Natland 230–400 mesh). TLC was performed with silica plates and visualized by using a UV lamp at 254 nm or iodine. The aldehydes, malononitrile and phenols were purchased from Sigma-Aldrich (St. Louis, MO, USA). Infrared spectra were recorded on a Perkin Elmer 2000 FT-IR instrument. Melting points were determined with a Fisher-Johns melting point apparatus. High resolution mass spectra (HRMS) were captured with electron ionization (70 eV) on a JEOL JSM-GC Mate II spectrometer. NMR spectra were obtained on Varian Mercury (300 MHz), Varian VNMR System (500 MHz), and Bruker 600 AVANCE III (600 MHz) instruments, utilizing acetone-*d*_6_ and CDCl_3_ as solvents. Most of the NMR assignments are based on extensive 2D homo- and hetero-nuclear experiments. The purity of compounds was determined by high performance liquid chromatography (HPLC) with UV-visible detector Agilent Technologies 1260 model with Eclipse XDB-C18 column of 5 µm and 4.6 × 250 mm. All samples were run with a flow of 100% MeCN (1 mL/min).

### 2.2. General Procedure for the Preparation of 2-Amino-3-cyano-4H-chromenes **4a–o** and **6a–h**

A mixture of aldehyde (**1a–i**), malononitrile (**2**) and 3,4-methylenedioxyphenol (**3**) or 3,5-dimethoxyphenol (**5**) (1:1:1 mol equiv) were combined in ethanol (15 mL), and then piperidine (0.2 mol equiv) was added and stirred at room temperature for 20 h. The solvent was removed under vacuum and the residue purified by flash chromatography on silica gel using *n*-hexane/EtOAc (8:2) as eluent, resulting in the corresponding 2-amino-3-cyano-4*H*-chromenes **4a–o** and **6a–h**. To our knowledge, there are no previous reports of **4d**, **4e**, **4i–o**, **6b**, **6d** or **6e**. Although **4a**, **4b**, **4c**, **4f**, **4g**, **4h**, **6a**, **6c** and **6f–h** are not new compounds, some of them have not been fully characterized in the literature. Consequently, their reported melting points are indicated, and the missing spectroscopic data are herein provided for their proper structural characterization.

#### 2.2.1. 2-Amino-3-cyano-4-phenyl-6,7-methylendioxy-4*H*-chromene (**4a**)

According to the general method, aldehyde **1a** (0.23 g, 2.17 mmol), malononitrile (**2**) (0.143 g, 2.17 mmol), phenol **3** (0.3 g, 2.17 mmol), and piperidine (0.037 g, 0.434 mmol) were mixed in ethanol. The crude was separated by flash column chromatography, affording 0.47 g (74%) of **4a** as a white solid; mp 203–205 °C (Lit. [41] 207–210 °C). HRMS (EI): *m*/*z* [M]^+^ calcd for C_17_H_12_N_2_O_3_: 292.0848; found: 292.0838.

#### 2.2.2. 2-Amino-3-cyano-4-(4-fluorophenyl)-6,7-methylendioxy-4*H*-chromene (**4b**)

Following the general method, **1b** (0.27 g, 2.17 mmol), **2** (0.143 g, 2.17 mmol), **3** (0.3 g, 2.17 mmol), and piperidine (0.037 g, 0.434 mmol) were combined in ethanol, furnishing 0.43 g (64%) of **4b** as an orange solid; mp 200–203 °C. FT-IR (CH_2_Cl_2_) v_max_ 3451, 3333, 3223, 2194, 1672 cm^−1^. HRMS (EI): *m*/*z* [M]^+^ calcd for C_17_H_11_N_2_O_3_F: 310.0754; found: 310.0754 (Lit. [42] 311.0826).

#### 2.2.3. 2-Amino-3-cyano-4-(4-chlorophenyl)-6,7-methylendioxy-4*H*-chromene (**4c**)

According to the general method, **1c** (0.305 g, 2.17 mmol), **2** (0.143 g, 2.17 mmol), **3** (0.3 g, 2.17 mmol), and piperidine (0.037 g, 0.434 mmol) were mixed in ethanol, delivering 0.433 g (61%) of **4c** as a beige solid; mp 202–204 °C (Lit. [41], 205–208 °C). HRMS (EI): *m*/*z* [M]^+^ calcd for C_17_H_11_N_2_O_3_CI: 326.0458; found: 326.0459.

#### 2.2.4. 2-Amino-4-(4-bromophenyl)-3-cyano-6,7-methylendioxy-4*H*-chromene (**4d**)

Following the general method, **1d** (0.402 g, 2.17 mmol), **2** (0.143 g, 2.17 mmol), **3** (0.3 g, 2.17 mmol), and piperidine (0.037 g, 0.434 mmol) were combined in ethanol, obtaining 0.572 g (71%) of **4d** as a pale orange solid; mp 220–223 °C. FT-IR (CH_2_Cl_2_) v_max_ 3450, 3338, 3222, 2193, 1671 cm^−1^. ^1^H NMR (600 MHz, acetone-d_6_) δ 4.68 (s, 1H, H-4), 5.96 (d, *J* = 22.2 Hz, 2H, H-13), 6.21 (bs, 2H, NH_2_), 6.49 (s, 1H, H-5), 6.58 (s, 1H, H-8), 7.21 (d, *J* = 8.4, 2H, H-10), 7.50 (d, *J* = 8.4, 2H, H-11). ^13^C NMR (150 MHz, acetone-d_6_) δ 41.7 (C-4), 58.3 (C-2), 98.5 (C-5), 102.8 (C-13), 108.2 (C-8), 115.8 (C-5a), 120.0 (CN), 121.1 (C-9), 130.5 (C-10), 132.5 (C-11), 144.0 (C-8a), 145.6 (C-7), 146.2 (C-12), 148.3 (C-6), 161.0 (C-3). HRMS (EI): *m*/*z* [M]^+^ calcd for C_17_H_11_N_2_O_3_Br: 369.9953; found: 369.9968.

#### 2.2.5. 2-Amino-3-cyano-4-(4-cyanophenyl)-6,7-methylendioxy-4*H*-chromene (**4e**)

According to the general method, **1e** (0.285 g, 2.17 mmol), **2** (0.143 g, 2.17 mmol), **3** (0.3 g, 2.17 mmol), and piperidine (0.037 g, 0.434 mmol) were mixed in ethanol, giving 0.47 g (68%) of **4e** as a beige solid; mp 250–252 °C. FT-IR (CH_2_Cl_2_) v_max_ 3422, 3297, 3178, 3178, 2155, 1655 cm^−1^. ^1^H NMR (600 MHz, acetone-d_6_) δ 4.81 (s, 1H, H-4), 5.97 (d, *J* = 21 Hz, 2H, H-13), 6.24 (bs, 2H, NH_2_), 6.51 (s, 1H, H-8), 6.60 (s, 1H, H-5), 7.46 (d, *J* = 7.8 Hz, 2H, H-10), 7.74 (d, *J* = 8.4 Hz, 2H, H-11). ^13^C NMR (150 MHz, acetone-d_6_) δ 42.3 (C-4), 58.0 (C-2), 98.7 (C-5), 102.9 (C-13), 108.1 (C-8), 111.6 (C-9), 115.1 (C-5a), 119.2 (Ar-CN), 119.8 (CN), 129.5 (C-10), 133.5 (C-13), 144.2 (C-8a), 145.8 (C-7), 148.5 (C-6), 152.0 (C-12), 161.3 (C-3). HRMS (EI): *m*/*z* [M]^+^ calcd for C_18_H_11_N_3_O_3_: 317.0800; found: 317.0801.

#### 2.2.6. 2-Amino-3-cyano-6,7-methylendioxy-4-(4-nitrophenyl)-4*H*-chromene (**4f**)

Following the general method, **1f** (0.328 g, 2.17 mmol), **2** (0.143 g, 2.17 mmol), **3** (0.3 g, 2.17 mmol), and piperidine (0.37 g, 0.434 mmol) were combined in ethanol, generating 0.47 g (64%) of **4f** as a pale orange solid; mp 258–260 °C (Lit. [43] 162–165 °C). ^13^C NMR (75 MHz, acetone-d_6_) δ 41.2 (C-4), 59.260.5 (C-2), 98.3 (C-5), 102.0 (C-13), 104.8 (C-8), 113.2 (C-5a), 119.3 (CN), 124.3 (C-11), 128.8 (C-10), 143.2 (C-8a), 145.2 (C-6), 147.3 (C-12), 147.9 (C-7), 151.7 (C-9), 159.5 (C-3).

#### 2.2.7. 2-Amino-3-cyano-6,7-methylendioxy-4-(p-tolyl)-4*H*-chromene (**4g**)

According to the general method, **1g** (0.261 g, 2.17 mmol) **2** (0.143 g, 2.17 mmol), **3** (0.3 g, 2.17 mmol), and piperidine (0.037 g, 0.434 mmol) were mixed in ethanol, producing 0.56 g (84%) of **4g** as a cream-colored solid; mp 208–210 °C (Lit. [41,42] 208–211 °C). HRMS (EI): *m*/*z* [M]^+^ calcd for C_18_H_14_N_2_O_3_: 306.1005; found: 306.1004.

#### 2.2.8. 2-Amino-3-cyano-6,7-methylendioxy-4-(4-methoxyphenyl)-4*H*-chromene (**4h**)

Following the general method, **1h** (0.296 g, 2.17 mmol), **2** (0.143 g, 2.17 mmol), **3** (0.3 g, 2.17 mmol), and piperidine (0.037 g, 0.434 mmol) were combined in ethanol, yielding 0.336 g (48%) of **4h** as a pale orange solid; mp 190–192 °C (Lit. [41,42] 197–201 °C). HRMS (EI): *m*/*z* [M]^+^ calcd for C_18_H_14_N_2_O_4_: 322.0954; found: 322.0951.

#### 2.2.9. 2-Amino-3-cyano-6,7-methylendioxy-4-(pyridin-4-yl)-4*H*-chromene (**4i**)

According to the general method, **1i** (0.232 g, 2.17 mmol), **2** (0.143 g, 2.17 mmol), **3** (0.3 g, 2.17 mmol), and piperidine (0.037 g, 0.434 mmol) were mixed in ethanol, leading to 0.35 g (55%) of **4i** as a yellow solid; mp 225–227 °C. FT-IR (CH_2_Cl_2_) v_max_ 3309, 3081, 2159, 1660, 1597 cm^−1^. ^1^H NMR (600 MHz, acetone-d_6_) δ 4.71 (s, 1H, H-4), 5.98 (d, *J* = 22.2 Hz, 2H, H-12), 6.27 (bs, 2H, NH_2_), 6.54 (s, 1H, H-8), 6.61 (s, 1H, H-5), 7.22–7.24 (m, 2H, H-10), 8.50–8.53 (m, 2H, H-11). ^13^C NMR (150 MHz, acetone-d_6_) δ 41.7 (C-4), 57.5 (C-2), 98.7 (C-5), 102.8 (C-12), 108.0 (C-8), 114.9 (C-5a), 119.8 (CN), 123.4 (C-10), 144.3 (C-8a), 145.7 (C-7), 148.5 (C-6), 151.1 (C-11), 154.8 (C-9), 161.5 (C-3). HRMS (EI): *m*/*z* [M]^+^ calcd for C_16_H_11_N_3_O_3_: 293.0800; found: 293.0805.

#### 2.2.10. 2-Amino-3-cyano-4-(3-fluorophenyl)-6,7-methylendioxy-4*H*-chromene (**4j**)

According to general method, **1a** (0.192 g, 1.81 mmol), **2** (0.119 g, 1.81 mmol), **3** (0.25 g, 1.81 mmol) and piperidine (0.031 g, 0.362 mmol) were mixed in ethanol, leading to 0.32 g (57%) of **4j** as a pale-yellow solid; mp 194–196 °C. FT-IR (CH_2_Cl_2_) v_max_ 3452, 3330, 3213, 2903, 2192, 1660 cm^–1^. NMR ^1^H (600 MHz, DMSO-d_6_) δ 4.69 (s, 1H, H-4), 6.00 (d, *J* = 32.4 Hz, 2H, H-15), 6.60 (s, 1H, H-8), 6.69 (s, 1H, H-5), 6.90–7.20 (m, 5H, H-10, H-12, H-14, NH_2_), 7.33–7.46 (m, 1H, H-13). NMR ^13^C (150 MHz, DMSO-d_6_) δ 40.8 (C-4), 55.6 (C-2), 98.3 (C-5), 102.2 (C-15), 107.7 (C-8), 114.1 (C-10, *J* = 20.8 Hz), 114.4 (C-12, *J* = 21.3 Hz), 115.3 (C-5a), 120.9 (CN), 123.8 (C-14), 131.2 (C-13, *J* = 7.95 Hz), 143.2 (C-8a), 144.6 (C-7), 147.4 (C-6), 149.3 (C-9, *J* = 6 Hz), 161.0 (C-3), 162.7 (C-11, *J* = 242.8 Hz). HRMS (EI): *m*/*z* calcd for C_17_H_11_N_2_O_3_F [M]^+^ 310.0754 found; 310.0756.

#### 2.2.11. 2-Amino-3-cyano-4-(3-cyanophenyl)-6,7-methylendioxy-4*H*-chromene (**4k**)

According to general method, **1a** (0.237 g, 1.81 mmol), **2** (0.119 g, 1.81 mmol), 3 (0.25 g, 1.81 mmol) and piperidine (0.031 g, 0.362 mmol) were mixed in ethanol, leading to 0.4 g (70%) of **4k** as a pale-yellow solid; mp 232–234 °C. FT-IR (CH_2_Cl_2_) v_max_ 3441, 3342, 2905, 2228, 2188, 1656 cm^–1^. NMR ^1^H (600 MHz, DMSO-d_6_) δ 4.77 (s, 1H, H-4), 6.00 (d, *J* = 33 Hz, 2H, H-15), 6.58 (s, 1H, H-8), 6.70 (s, 1H, H-5), 7.00 (br s, 2H, NH_2_), 7.50–7.60 (m, 2H, H-13, H-14), 7.68 (s, 1H, H-10), 7.69–7.75 (m, 1H, H-12). NMR ^13^C (150 MHz, DMSO-d_6_) δ 40.6 (C-4), 55.2 (C-2), 98.3 (C-5), 102.2 (C-15), 107.7 (C-8), 112.0 (C-11), 114.7 (C-5a), 119.2 (CN’), 120.7 (CN), 130.6 (C-13), 131.3 (C-10), 131.3 (C-12), 132.8 (C-14), 143.2 (C-8a), 144.7 (C-7), 147.5 (C-6), 147.8 (C-9), 161.0 (C-3). HRMS (EI): *m*/*z* calcd for C_18_H_11_N_3_O_3_ [M]^+^ 317.0800 found; 317.0801.

#### 2.2.12. 2-Amino-3-cyano-4-(2-fluorophenyl)-6,7-methylendioxy-4*H*-chromene (**4l**)

According to general method, **1a** (0.225 g, 1.81 mmol), **2** (0.119 g, 1.81 mmol), 3 (0.25 g, 1.81 mmol) and piperidine (0.031 g, 0.363 mmol) were mixed in ethanol, leading to 0.47 g (84%) of **4l** as a beige solid; mp 198–200 °C. FT-IR (CH_2_Cl_2_) v_max_ 3459, 3328, 3197, 2887, 2197, 1663 cm^–1^. NMR ^1^H (600 MHz, DMSO-d_6_) δ 4.91 (s, 1H, H-4), 5.98 (d, *J* = 30 Hz, 2H, H-15), 6.48 (s, 1H, H-8), 6.69 (s, 1H, H-5), 6.91 (br s, 2H, NH_2_), 7.13–7.24 (m, 3H, H-11, H-12, H-13), 7.26–7.35 (m, 1H, H-14). NMR ^13^C (150 MHz, DMSO-d_6_) δ 35.6 (C-4), 54.6 (C-2), 98.2 (C-5), 102.2 (C-15), 107.4 (C-8), 114.5 (C-5a), 116.2 (C-11, *J* = 21.45 Hz), 120.8 (CN), 125.2 (C-13), 129.5 (C-14, *J* = 8.1 Hz), 130.2 (C-12), 132.7 (C-9, *J* = 12.45 Hz), 143.5 (C-8a), 144.6 (C-7), 147.4 (C-6), 160.4 (C-10, *J* = 244.2 Hz), 161.2 (C-3). HRMS (EI): *m*/*z* calcd for C_17_H_11_N_2_O_3_F [M]^+^ 310.0754 found; 310.0753.

#### 2.2.13. 2-Amino-3-cyano-4-(2-chlorophenyl)-6,7-methylendioxy-4*H*-chromene (**4m**)

According to the general method, **1a** (0.254 g, 1.81 mmol), **2** (0.119 g, 1.81 mmol), 3 (0.25 g, 1.81 mmol) and piperidine (0.031 g, 0.363 mmol) were mixed in ethanol, leading to 0.3 g (51%) of **4m** as a pale yellow solid; mp 218–220 °C. FT-IR (CH_2_Cl_2_) v_max_ 3442, 3327, 3201, 2899, 2204, 1664 cm^–1^. NMR ^1^H (600 MHz, DMSO-d_6_) δ 5.14 (s, 1H, H-4), 5.98 (d, *J* = 28.8 Hz, 2H, H-15), 6.39 (s, 1H, H-8), 6.69 (s, 1H, H-5), 6.92 (s, 2H, NH_2_), 7.19–7.23 (m, 1H, H-14), 7.25–7.35 (m, 2H, H-13, H-12), 7.42–7.46 (m, 1H, H-11). NMR ^13^C (150 MHz, DMSO-d_6_) δ 38.5 (C-4), 54.8 (C-2), 98.2 (C-5), 102.2 (C-15), 107.0 (C-8), 114.5 (C-5a), 120.6 (CN), 128.3 (C-14), 129.2 (C-12), 130.3 (C-11), 131.2 (C-13), 132.2 (C-10), 142.8 (C-9), 143.4 (C-8a), 144.6 (C-7), 147.5 (C-6) 161.0 (C-3). HRMS (EI): *m*/*z* calcd for C_17_H_11_N_2_O_3_Cl [M]^+^ 326.0458 found; 326.0460.

#### 2.2.14. 2-Amino-3-cyano-4-(2-bromophenyl)-6,7-methylendioxy-4*H*-chromene (**4n**)

According to the general method, **1a** (0.335 g, 1.81 mmol), **2** (0.119 g, 1.81 mmol), 3 (0.25 g, 1.81 mmol) and piperidine (0.031 g, 0.363 mmol) were mixed in ethanol, leading to 0.46 g (68%) of **4n** as a dark yellow solid; mp 223–225 °C. FT-IR (CH_2_Cl_2_) v_max_ 3442, 3327, 3201, 2899, 2204, 1664 cm^–1^. FT-IR (CH_2_Cl_2_) v_max_ 3444, 3326, 3199, 2204, 1664 cm^–1^. NMR ^1^H (600 MHz, DMSO-d_6_) δ 5.16 (s, 1H, H-4), 5.98 (d, *J* = 28.2 Hz, 2H, H-15), 6.37 (s, 1H, H-8), 6.70 (s, 1H, H-5), 6.93 (br s, 2H, NH_2_), 7.16–7.25 (m, 2H, H-13, H-14), 7.33–7.40 (m, 1H, H-12), 7.58–7.64 (m, 1H, H-11). NMR ^13^C (150 MHz, DMSO-d_6_) δ 40.6 (C-4), 55.1 (C-2), 98.3 (C-5), 102.2 (C-15), 106.8 (C-8), 114.6 (C-5a), 120.5 (CN), 122.6 (C-10), 129.0 (C-12), 129.5 (C-13), 131.4 (C-14), 133.4 (C-11), 143.3 (C-7), 144.6 (C-9, C-8a), 147.5 (C-6), 160.9 (C-3). HRMS (EI): *m*/*z* calcd for C_17_H_11_N_2_O_3_Br [M]^+^ 369.9953 found; 369.9954.

#### 2.2.15. 2-Amino-3-cyano-4-(2-nitrophenyl)-6,7-methylendioxy-4*H*-chromene (**4o**)

According to the general method, **1a** (0.273 g, 1.81 mmol), **2** (0.119g, 1.81 mmol), 3 (0.25 g, 1.81 mmol) and piperidine (0.031 g, 0.363 mmol) were mixed in ethanol, leading to 0.43 g (70%) of **4n** as a pale yellow solid; mp 208–210 °C. FT-IR (CH_2_Cl_2_) v_max_ 3442, 3350, 2185, 1656 cm^–1^. NMR ^1^H (600 MHz, DMSO-d_6_) δ 4.67 (s, 1H, H-4), 5.98 (d, *J* = 31.8 Hz, 2H, H-15), 6.58 (s, 1H, H-8), 6.69 (s, 1H, H-5), 6.96 (br s, 2H, NH_2_), 7.15–7.20 (m, 1H, H-14), 7.24 (s, 1H, H-13), 7.27–7.32 (m, 1H, H-12), 7.33–7.40 (m, 1H, H-11). NMR ^13^C (150 MHz, DMSO-d_6_) δ 40.7 (C-4), 55.6 (C-2), 98.3 (C-5), 102.2 (C-15), 107.7 (C-8), 115.2 (C-5a), 120.8 (CN), 126.5 (C-14), 127.3 (C-12), 127.5 (C-13), 131.1 (C-11), 133.7 (C-9), 143.2 (C-8a), 144.6 (C-7), 147.4 (C-6), 148.9 (C-10), 161.0 (C-3). HRMS (EI): *m*/*z* calcd for C_17_H_11_N_3_O_5_ [M]^+^ 337.0699 found; 337.0694.

#### 2.2.16. 2-Amino-3-cyano-5,7-dimethoxy-4-phenyl-4*H*-chromene (**6a**)

Following the general method, **1a** (0.138 g, 1.3 mmol), **2** (0.086 g, 1.3 mmol), **5** (0.2 g, 1.3 mmol), and piperidine (0.022 g, 0.259 mmol) were combined in ethanol, resulting in 0.23 g (57%) of **6a** as a beige solid; mp 176–178 °C (Lit. [44], 212–214 °C). FT-IR (KBr): v_max_ 3427, 3344, 3002, 2190, 1655, 1619, 1585, 1500, 1457, 1403, 1202, 1147, 1105, 827, 809, 753, 705 cm^−1^. HRMS (EI): *m*/*z* [M]^+^ calcd for C_18_H_16_N_2_O_3_: 308.1161; found: 308.1159.

#### 2.2.17. 2-Amino-3-cyano-5,7-dimethoxy-4-(4-fluorophenyl)-4*H*-chromene (**6b**)

According to the general method, **1b** (0.161 g, 1.3 mmol), **2** (0.086 g, 1.3 mmol), **5** (0.2 g, 1.3 mmol), and piperidine (0.022g, 0.259 mmol) were mixed in ethanol, providing 0.192 g (45%) of **6b** as a beige solid; mp 188–190 °C. FT-IR (CH_2_Cl_2_): v_max_ 3475, 3329, 2962, 2196, 1658, 1582, 1505, 1399, 1223, 1204, 1147, 1105, 1049, 822, 798 cm^−1^. ^1^H NMR (600 MHz, acetone-d_6_) δ 3.67 (s, 3H, 5-OCH_3_), 3.78 (s, 3H, 7-OCH_3_), 4.62 (s, 1H, H-4), 6.15 (br s, 2H, NH_2_), 6.24 (d, *J* = 2.3 Hz, 1H, H-8), 6.30 (d, *J* = 2.2 Hz, 1H, H-6), 6.98–7.02 (m, 2H, H-11), 7.22–7.17 (m, 2H, H-10). ^13^C NMR (150 MHz, acetone-d_6_) δ 36.3 (C-4), 55.1 (7-OCH_3_), 55.4 (5-OCH_3_), 59.6 (C-3), 93.1 (C-8), 95.1 (C-6), 105.1 (C-4a), 114.8, 114.9 (C-11, *J* = 21.25 Hz), 119.5 (CN), 129.1, 129.2 (C-10, *J* = 21.25 Hz), 142.2 (C-9, *J* = 2.55 Hz), 150.4 (C-8a), 157.7 (C-5), 160.3 (C-2), 160.7 (C-7), 162.3 (C-12, *J* = 241.05 Hz). HRMS (EI): *m*/*z* [M]^+^ calcd for C_18_H_15_N_2_O_3_F: 326.1067; found: 326.1070.

#### 2.2.18. 2-Amino-3-cyano-5,7-dimethoxy-4-(4-chlorophenyl)-4*H*-chromene (**6c**)

Following the general method, **1c** (0.182 g, 1.3 mmol), **2** (0.86 g, 1.3 mmol), **5** (0.2 g, 1.3 mmol), and piperidine (0.022 g, 0.259 mmol) were combined in ethanol, furnishing 0.191 g (43%) of **6c** as a white solid; mp 193–195 °C (Lit. [44], 234–236 °C). FT-IR (CH_2_Cl_2_): v_max_ 3334, 2193, 1652, 1621, 1582, 1471, 1397, 1204, 1146, 1103, 818, 740 cm^−1^. HRMS (EI): *m*/*z* [M]^+^ calcd for C_18_H_15_N_2_O_3_Cl: 342.0771; found: 342.0768.

#### 2.2.19. 2-Amino-4-(4-bromophenyl)-3-cyano-5,7-dimethoxy-4*H*-chromene (**6d**)

According to the general method, **1d** (0.24 g, 1.3 mmol), **2** (0.086 g, 1.3 mmol), **5** (0.2 g, 1.3 mmol), and piperidine (0.022 g, 0.259 mmol) were mixed in ethanol, affording 0.238 g (47%) of **6d** as a cream-colored solid; mp 192–194 °C. FT-IR (KBr): v_max_ 3472, 3328, 3207, 2196, 1660, 1587, 1400, 1205, 1147, 1101, 1058, 825, 806, 790 cm^−1^. ^1^H NMR (600 MHz, acetone-d_6_) δ 3.67 (s, 3H, 5-OCH_3_), 3.78 (s, 3H, 7-OCH_3_), 4.60 (s, 1H, H-4), 6.17 (br s, 2H, NH_2_), 6.24 (d, *J* = 2.0 Hz, 1H, H-8), 6.30 (d, *J* = 2.0 Hz, 1H, H-6), 7.12 (d, *J* = 8.3 Hz, 2H, H-10), 7.43 (d, *J* = 8.3 Hz, 2H, H-11). ^13^C NMR (150 MHz, acetone-d_6_) δ 37.2 (C-4), 55.8 (7-OCH_3_), 56.1 (5-OCH_3_), 59.9 (C-3), 93.8 (C-8), 95.8 (C-6), 105.35 (C-4a), 120.1 (CN), 120.4 (C-12), 130.2 (C-10), 132.0 (C-11), 146.2 (C-9), 151.2 (C-8a), 158.4 (C-5), 161.0 (C-2), 161.5 (C-7). HRMS (EI): *m*/*z* [M]^+^ calcd for C_18_H_15_N_2_O_3_Br: 386.0266; found: 386.0262.

#### 2.2.20. 2-Amino-3-cyano-4-(4-cyanophenyl)-5,7-dimethoxy-4*H*-chromene (**6e**)

Following the general method, **1e** (0.17 g, 1.3 mmol), **2** (0.086 g, 1.3 mmol), **5** (0.2 g, 1.3 mmol), and piperidine (0.022 g, 0.259 mmol) were combined in ethanol, delivering 0.2 g (46%) of **6e** as a white solid; mp 243–245 °C. FT-IR (KBr): v_max_ 3400, 3331, 3217, 2232, 2190, 1662, 1633, 1585, 1500, 1405, 1204, 1149, 1103, 1055, 820, 795 cm^−1^. ^1^H NMR (500 MHz, acetone-d_6_) δ 3.67 (s, 3H, 5-OCH_3_), 3.79 (s, 3H, 7-OCH_3_), 4.71 (s, 1H, H-4), 6.20 (br s, 1H, NH_2_), 6.26 (d, *J* = 2.3 Hz, 1H, H-8), 6.32 (d, *J* = 2.3 Hz, 1H, H-6), 7.36 (d, *J* = 8.5 Hz, 2H, H-10), 7.67 (d, *J* = 8 Hz, 2H, H-11). ^13^C NMR (125 MHz, acetone-d_6_) δ 37.9 (C-4), 55.9 (7-OCH_3_), 56.2 (5-OCH_3_), 59.3 (C-3), 94.0 (C-8), 95.9 (C-6), 104.7 (C-4a), 111.0 (C-12), 119.3 (Ar-CN), 119.9 (CN), 129.2 (C-10), 133.0 (C-11), 151.2 (C-8a), 152.2 (C-9), 158.5 (C-5), 161.1 (C-2), 161.7 (C-7). HRMS (EI): *m*/*z* [M]^+^ calcd for C_19_H_15_N_3_O_3_: 333.1113; found: 333.1108.

#### 2.2.21. 2-Amino-3-cyano-5,7-dimethoxy-4-(4-nitrophenyl)-4*H*-chromene (**6f**)

According to the general method, **1f** (0.196 g, 1.3 mmol), **2** (0.086 g, 1.3 mmol), **5** (0.2 g, 1.3 mmol), and piperidine (0.022 g, 0.259 mmol) were mixed in ethanol, obtaining 0.207 g (45%) of **6f** as a cream-colored solid; mp 230–232 °C (Lit. [44], 245–247 °C). FT-IR(KBr): v_max_ 3434, 3316, 3198, 2980, 2200, 1655, 1579, 1515, 1500, 1405, 1349, 1208, 1148, 1053, 824, 711 cm^−1^. HRMS (EI): *m*/*z* [M]^+^ calcd for C_18_H_15_N_3_O_5_: 353.1012; found: 353.1014.

#### 2.2.22. 2-Amino-3-cyano-5,7-dimethoxy-4-(p-tolyl)-4*H*-chromene (**6g**)

Following the general method, **1g** (0.156 g, 1.3 mmol), **2** (0.086 g, 1.3 mmol), **5** (0.2 g, 1.3 mmol), and piperidine (0.022 g, 0.259 mmol) were combined in ethanol, giving 0.184 g (44%) of **6g** as a pale-yellow solid; mp 172–174 °C (Lit. [44], 217–219 °C). FT-IR(KBr): v_max_ 3475, 3329, 2962, 2196, 1658 cm^−1^. HRMS (EI): *m*/*z* [M]^+^ calcd for C_19_H_18_N_2_O_3_: 322.1318; found: 322.1318.

#### 2.2.23. 2-Amino-3-cyano-5,7-dimethoxy-4-(4-methoxyphenyl)-4*H*-chromene (**6h**)

According to the general method, **1h** (0.176 g, 1.3 mmol), **2** (0.086 g, 1.3 mmol), **5** (0.2 g, 1.3 mmol), and piperidine (0.022 g, 0.259 mmol) were mixed in ethanol, generating 0.132 g (30%) of **6h** as a cream-colored solid; mp 186–188 °C (Lit. [44], 220–222 °C). FT-IR(KBr): v_max_ 3331, 2942, 2197, 1660, 1622, 1397, 1204, 1146, 1102, 1057, 798 cm^−1^. HRMS (EI): *m*/*z* [M]^+^ calcd for C_19_H_18_N_2_O_4_: 338.1267; found: 338.1268.

### 2.3. In-Silico Analysis of 2-Amino-3-cyano-4-aryl-6,7-methylendioxy-4H-chromenes **4a–o** and 2-Amino-3-cyano-5,7-dimethoxy-4-aryl-4H-chromenes **6a–h**

The physicochemical and toxicological properties of 2-amino-3-cyano-4*H*-chromenes **4a–o** and **6a–h** were determined on the OSIRIS DataWarrior V4.7.2 program (https://openmolecules.org/datawarrior/, accessed on 26 October 2021) [45]. The drug similarity and pharmacokinetic properties, including molar refractivity and the number of rotatable bonds, were calculated with the SwissADME server platform [46]. The physicochemical properties were evaluated based on Lipinski’s rules, considering Log P, molecular weight, hydrogen bond donors, and hydrogen bond acceptors [47].

#### 2.3.1. Multiple Sequence Alignment and Generation of 3D CYP51 from *Candida* spp. through Homology Modeling

The sequences of the lanosterol 14α-demethylase enzymes (CYP51) were downloaded from the NCBI database (https://www.ncbi.nlm.nih.gov, accessed on 26 October 2021) [48] for *C. albicans* AATCC 10231 (CYP51CA), *C. dubliniensis* CD36 (CYP51CD), *C. glabrata* CBS138 (CYP51CG), *C. kefyr* (CYP51CKE), *C. krusei* ATCC 6358 (CYP51CK) and *C. parapsilosis* ATCC 22019 (CYP51CP). The percentage of identity of each of the CYP51 sequences of *Candida* spp. with the target protein CYP51 of *Saccharomyces cerevisiae* (CYP51SC), complexed with fluconazole (**8**) in the active site (PDB: 4WMZ), was determined with the BLASTp (protein query-protein database) server. The multiple alignment of sequences was performed with CLUSTAL X [49]. The sequences of CYP51 *Candida* spp. were used for the elaboration of 3D models, based on the homology modeling technique with the Modeller 9.23 program [50]. For these models, the crystallized structure of *S. cerevisiae* CYP51 (PDB: 4wmz), deposited in the protein data bank (https://www.rcsb.org/structure/4WMZ, accessed on 26 October 2021) [51], served as a template. The overlapping of the six selected CYP51 models of *Candida* spp. was carried out on the Discovery Studio Visualizer [52].

#### 2.3.2. Evaluation and Validation of the 3D Model of CYP51 from *Candida* spp.

Of the fifteen models obtained for each CYP51 of the different *Candida* spp., the one with the minimum score was chosen. The 3D models of CYP51 were examined with the DOPE method [53] and validated with the VERIFY3D [54] and PROCHECK [55] programs. The former was utilized to assess the compatibility of an atomic model (3D) with its own amino acid sequence (1D), and the latter to check the stereochemical quality of the Ramachandran plots that show the amino acid residues in the allowed regions.

#### 2.3.3. Molecular Docking on CYP51

The 4*H*-chromenes **4a–o** and **6a–h** were docked on the active site of the CYP51 enzymes of *C. albicans*, *C. dubliniensis*, *C. glabrata*, *C. kefyr*, *C. krusei*, and *C. parapsilosis* by using the AutoDock4 program [56]. The CYP51 enzymes of the *Candida* spp. were previously modeled with the crystallized proteins of CYP51 from *Saccharomyces cerevisiae* (CYP51SC) (PDB: 4WMZ), deposited in the protein data bank [57], as a template. The proteins were prepared by adding hydrogen atoms and removing water molecules around them, and then were optimized on the Nanoscale Molecular Dynamics (NAMD) program [58].

Fluconazole (**8**) and the 2-amino-3-cyano-4*H*-chromenes **4a–o** and **6a–h** were drawn with ChemBioDraw Ultra 12.0 software [59], converted to 3D on the Open Babel GUI program [60], and optimized with GaussView 6.0 software [61], which involves the addition of hydrogen atoms and the verification of the bond lengths and angles.

The docking parameters were established with AutoDock Tools, with grid dimensions of 44 × 44 × 44 Å^3^ and points separated by 0.375 Å. The grid center used for the CYP51 *Candida* spp. was (X = 27.7, Y = 10.1 and Z = 18.95). The Lamarckian Genetic algorithm was used with 100 docking runs. The docked model with the lowest binding energy was selected. The docking results were edited in Discovery Studio [52].

#### 2.3.4. Molecular Docking on Topoisomerase I

The 3D structure of topoisomerase I was obtained from the Protein Data Bank (PDB entry no. 1SC7, https://www.rcsb.org/structure/1SC7, accessed on 26 October 2021) [62]. The water molecules were removed from the structure to delimit the grid, and the active site was identified with AutoGrid. Hydrogen atoms were added to the polar atoms (considering a pH of 7.4), and the Kollman charges were assigned. The topotecan 3D structure was downloaded from Zincdatabase [63]. The 2D structures of the 2-amino-3-cyano-4*H*-chromenes **4a–o** and **6a–h** ligands were drawn with ChemSketch (https://www.acdlabs.com/resources/freeware/chemsketch/index.php, accessed on 26 October 2021), and subsequently, the optimization of the structure to an AM1 level was performed with Gaussian 98 [61], in order to find the lowest energy conformation for each ligand.

Molecular docking simulations were carried out with AutoDock version 4.0 [56]. For topoisomerase I, the grid dimensions were 90 × 90 × 90 Å^3^ with the points separated by 0.375 Å. The grid center was X = 85.385, Y = −10.629 and Z = 10945. Default values of translation, quaternation, torsion steps, atomic partial charges, and non-polar hydrogens were utilized for the simulation. The hybrid Lamarckian Genetic Algorithm (at default settings) was applied for minimization. The number of docking runs was 100, with a population size of 150. The maximum number of energy evaluation ranges from 27,000 to 250,000, the latter being the maximum number of generations. The mutation rate was 0.02 and the cross-over rate was 0.8. The docked model with the lowest binding energy was considered for all further simulations. Docking results were analyzed on AutoDockTools and edited with Discovery 4.0 Client [52].

### 2.4. Antifungal Activity Assays

To determine the in vitro minimum inhibitory concentrations (MIC) for fluconazole (**8**) and the 2-amino-3-cyano-4*H*-chromenes **4a–i** and **6a–h** on six *Candida* species (*C. albicans*, *C. dubliniensis*, *C. glabrata*, *C. kefyr*, *C. krusei*, and *C parapsilosis*), the standard guidelines of the National Committee for Clinical Laboratory Standards (NCCLS) were employed, with an RPMI of 1640 (Sigma) buffered with 0.165 MMOPS (Sigma) as the test medium [64]. The MIC value was defined as the lowest concentration of a compound that generated a culture with turbidity less than or equal to 100% inhibition when compared with the growth of the control. The 4*H*-chromene derivatives **4a–i** and **6a–h** were dissolved in DMSO solvent and serially dripped into the growth medium. The concentration gradient of **8** was from 64–0.033 µg/mL and for the derivatives 160–0.078 µg/mL. The inoculum was prepared by resuspending colonies (from a yeast culture of 24 h growth) in a tube with saline solution (0.85% NaCl), and then adjusting them to an optical density 0.5 McFarland. Subsequently, they were incubated at 37 °C in an incubator. The MIC results were quantified in a Multiskan™ GO microplate spectrophotometer by agitation of the plates, followed by a spectrophotometric reading at 450 nm. The value is expressed as the average of three replicates.

### 2.5. Cytotoxicity Assay

The in vitro cytotoxicity of the compounds was evaluated on PC-3 and SK-LU-1 cell lines (human prostatic and lung adenocarcinoma, respectively), using the protein binding dye sulforhodamine B (SRB) in a microculture assay to measure cell growth [65]. The cell lines were grown in RPMI-1640 medium (Sigma Chemical Co., Ltd., St. Louis, MO, USA) supplemented with 10% fetal bovine serum (Invitrogen Corporation, Waltham, MA, USA), 2 mM L-glutamine, 10,000 units/mL of penicillin G, 10,000 µg/mL streptomycin, and 0.25 µg/mL of fungizone (Gibco, Amarillo, TX, USA). The cells were maintained at 37 °C in a 5% CO_2_ atmosphere with 95% humidity.

Preparations were made with 7.5104 cell/mL of the PC-3 cell line and 10104 cell/mL of the SK-LU-1 cell line, adding 100 mL of each of these suspensions to the wells of 96-well plates. Incubation was carried out for 24 h to promote cell attachment, and then 100 µL per well of a test compound, topotecan (**7**) or cisplatin (**9**) (as controls) were added. After 48 h, the adhered cell cultures were fixed in situ with a cold 50% (*w*/*v*) solution of trichloroacetic acid, followed by incubation at 4 °C for 1 h. Upon completion of this time, the supernatant was discarded and the plates were washed and air dried. Cells cultured with TCA were stained with 100 µL of 0.4% SRB solution for 30 min. The protein-bound dye was extracted with a 10 nm unbuffered tris base and the optical density was read at 515 nm on a Synergy HT Microplate Reader (Elx 808, BIO-TEK Instruments, Inc, Winooski, VT, USA). IC_50_ values were determined by the Monks protocol. Accordingly, a concentration–response curve was constructed for each of the 2-amino-3-cyano-4*H*-chromenes **4a–o** and **6a–h**, and the values corresponding to an inhibition of 50% (IC_50_) were calculated from non-linear regression equations. IC_50_ values are expressed as the mean ± standard error (SE) [66].

## 3. Results and Discussion

### 3.1. Chemistry

In view of the relevance of 4*H*-chromene derivatives as potential therapeutic agents, a synthetic access to 2-amino-3-cyano-4*H*-chromenes **4a–o** and **6a–h** was explored. Of these compounds, **4d**, **e**, **i–o** as well as **6b**, **d**, and **e** are reported for the first time. The synthesis took place via a three-component reaction, evaluating the effect of the base, solvent, and reaction temperature. Multicomponent reactions (MCRs) represent a powerful tool that can be utilized to form novel and complex molecules by reacting to three or more different starting materials in a one-pot strategy, resulting in great efficiency and atom economy [33]. The multicomponent approach included the cooperation of benzaldehydes (**1a–o**), malononitrile (**2**) and 3,4-methylenedioxyphenol (**3**) or 3,5-dimethoxyphenol (**5**), considering their reactivity and selectivity on the efficiency of the process.

Initially, the method focused on the efficient one-pot reaction of *p*-nitrobenzaldehyde (**1f**), **2**, and **3** (1:1:1 mol equiv), with Et_3_N (0.2 mol equiv) and ethanol as the solvent, at room temperature for 20 h. Moreover, 2-amino-3-cyano-6,7-methylendioxy-4-(4-nitrophenyl)-4*H*-chromene (**4f**) was provided in low yield (36%) (Table 1, entry 1). Besides **4f**, only the starting material and the Knoevenagel product were identified in the crude reaction mixture, without evidence of any other 4*H*-chromene regioisomer.

In order to improve the yield, bases such as piperidine and potassium carbonate were tested under identical reaction conditions. A better yield (64%) was obtained with piperidine (Table 1, entry 2), but an increase in its concentration and the reaction temperature did not lead to further improvement (Table 1, entries 4 and 8). Finally, to analyze the possible effect of the solvent on the reactivity and selectivity of the process, protic and aprotic solvents were used (ethanol, water, dioxane and acetonitrile) under the same reaction conditions. A protic polar solvent (ethanol) generated the best yields (Table 1, entry 2). 

Under the optimized reaction conditions, a variety of aromatic aldehydes with electron-donating and electron-withdrawing groups were employed to determine the scope of this methodology. The reactions of aryl aldehydes **1a**–**o**, **2** and **3** afforded 2-amino-3-cyano-4*H*-chromenes **4a**–**o** in 48–84% yields (Table 2, entries 1–15). By utilizing 3,5-dimethoxyphenol (**5**) rather than **3** under similar conditions, the respective 2-amino-3-cyano-4*H*-chromenes **6a**–**h** were produced in 30–57% yields (Table 2, entries 16–23). The yields were higher when the aryl aldehyde contained no substituent or electron-withdrawing group (Table 2, entries 1–6, 10–15 and 16–21).

The structures of 2-amino-3-cyano-4*H*-chromenes **4a**–**o** and **6a**–**h** were elucidated on the basis of their spectrometric data (1D and 2D experiment, IR and HRMS). For example, the ^1^H NMR spectrum of **4d** showed the presence of four singlets observed at 4.68, 6.21, 6.49 and 6.58 ppm, attributed to H-4, NH_2_ (as a broad singlet integrating for two protons), H-5 and H-8, respectively. Three doublets at 5.96, 7.21 and 7.50 ppm were assigned to the H-13, H-10 and H-11 protons, respectively. The ^13^C NMR spectrum displayed signals for the cyano group at 120.0 ppm, ten signals for aromatic carbons in the region of 98.5–148.3 ppm, two signals for sp^2^ carbon atoms at 58.3 (C-2) and 161.0 ppm (C-3), and two signals for sp^3^ carbons at 41.7 (C-4) and 102.8 (C-13) ppm. Furthermore, the HMBC spectrum of **4d** revealed two- and three-bond C/H long-range correlations between the proton at 4.68 ppm and the quaternary carbon atoms at 161.0 (C-3), 115.8 (C-5a), 121.1 (C-9), 58.3 (C-2), 144.0 (C-8a) and 120.0 (CN) ppm. Likewise, the signal at 5.96 ppm (H-13) indicated a three-bond C/H long-range correlation with the aromatic quaternary carbon atoms at 145.6 (C-7) and 148.3 (C-6) ppm. Hence, all the NMR data coincide with the existence of a 4*H*-chromene core. Finally, the HRMS (EI) of **4d** confirmed the expected mass (*m*/*z* 369.9968), while the infrared spectrum exhibited absorption bands at 3453 and 3327 cm^−1^, characteristic of NH_2_, and at 2193 cm^−1^, characteristic of CN [32,38].

**Scheme 1:**
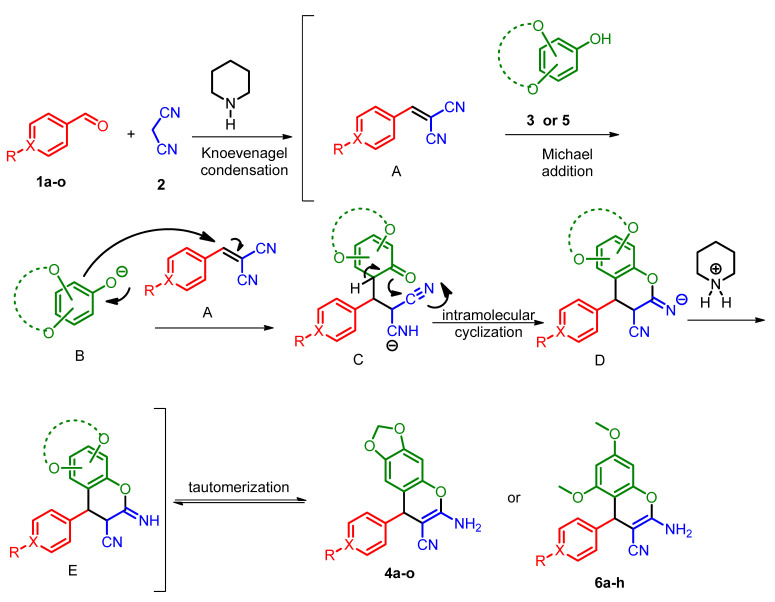
Plausible mechanism for the synthesis of 2-amino-3-cyano-4*H*-chromenes **4a**–**o** and **6a**–**h**.

A plausible mechanism of formation of 2-amino-3cyano-4*H*-chromenes has been proposed (Scheme 1). First, piperidine generates the deprotonation of the hydrogen acid in **2**, and the resulting malononitrile carbanion reacts with aromatic benzaldehydes (**1a**–**o**) to produce 2-arylidenemalononitrile intermediate A. Subsequently, piperidine reacts with the acidic protons of phenol (**3** or **5**) to form the corresponding phenoxide ion B, which undergoes a Michael addition with the arylidenemalononitrile to give rise to intermediate C. The latter cyclizes intramolecularly, evolving into D. Through a series of acid-base reactions, intermediate E is afforded and then converted by tautomerization to products **4a**–**o** or **6a**–**h**, respectively [30].

### 3.2. In Silico Analysis of Physicochemical, Pharmacokinetic, Drug-likeness and Toxicological Properties of 2-Amino-3-cyano-4H-chromenes **4a–o** and **6a–h**

Various pharmacological properties were evaluated with the OSIRIS DataWarrior program to predict the efficacy and behavior in the human body of the two series of 2-amino-3-cyano-4*H*-chromenes, **4a**–**o** and **6a**–**h** [45]. The first descriptor to be examined was lipophilicity (octanol/water partition coefficient, Log P), which indicates the concentration ratio of a neutral substance between two immiscible solvents in equilibrium, and reveals the differential solubility of this substance in the two solvents. Usually, one of these solvents is water and the other, a hydrophobic solvent, is octanol.

Lipophilicity is a very important physicochemical parameter because it influences the bioavailability, permeability and toxicity of a drug. Permeability is high, with Log P values close to 5 and low with negative values. The Log P values of **4a–o** and **6a–h** were acceptable, all under 5.0 and none of them negative. The highest values (3.21 and 3.26) correspond to **4d** and **6d** (R = Br). Compounds **4f** and **6f**, containing the polar substituent (R = NO_2_), had Log P values of 1.76 and 1.92, respectively (Table 3 and Table 4) [67].

Aqueous solubility (Log S) influences the absorption and distribution of a biologically active compound in an organism [68]. According to the calculated values, most of the present 2-amino-3-cyano-4*H*-chromenes are moderately soluble in water (−6 > Log S > −4), and only some of them (**4a**, **4e**, **4i**, **4k**, **6a** and **6e**) have a slight tendency to solubilize (−4 > Log S > −2). Another effective descriptor for predicting the drug solubility and transport properties of a molecule is the polar surface area (PSA), which is defined as the sum of the surfaces of its polar (N and O) and slightly polar atoms (S and P), and the hydrogen atoms attached to them. PSA is an indicator of gastrointestinal absorption and penetration of the blood–brain barrier. PSA values of 77.50 to 123.32 Å^2^ were found in the two series, **4a–o** and **6a–h**, evidencing an acceptable permeability. The best values were generated by the derivatives bearing halogen substituents on the phenyl aromatic ring. Compounds **4f** and **6f**, with nitro substituents and thus a greater number of electronegative atoms, showed the highest values of PSA (123.32 Å^2^), and consequently, the lowest permeability of cell membranes [69].

A different and useful parameter, ligand efficiency (LE), evaluates the effectiveness of a drug in its interaction with the active site of a therapeutic target. It is obtained by dividing the free binding energy of each molecule by the number of heavy non-hydrogen atoms in the structure (LE = ΔG_interaction_/[number of heavy non-hydrogen atoms]). Therefore, it considers the affinity and size of the ligand molecule, but not the size and topological properties of the molecular target [70]. Compounds **4a, 4i** and **4j** exhibited the highest ligand efficiency value in the series (LE = 0.407 and 0.512), which was above that of **7** (LE = 0.377; Table 3). This coincides with the cytotoxicity assays with human lung adenocarcinoma (SKLU), finding a better IC_50_ for **4a** than **7** (IC_50_ = 0.52 ± 0.03 vs. 2.0 ± 0.1; Table S11).

The ligand-lipophilicity efficiency (LLE) is also a relevant parameter for designing new drugs. It correlates the in vitro potency of a drug with the partition coefficient (log P) or the distribution coefficient (log D). A molecule with an LLE value of 0 (based on cLog P, or target affinity) has the same affinity for a therapeutic target as does *n*-octanol. In contrast, a compound with an LLE value of 6 has a million times more affinity towards the therapeutic target than *n*-octanol. Poor affinity for the therapeutic target is indicated by a negative LLE value, which was not the case for any of the derivatives in the two series, **4a–o** and **6a–h** (Table 3 and Table 4). These compounds all have a greater affinity for the active site of the target enzyme than *n*-octanol (due to specific interactions between the compounds and the amino acid side chain of the enzyme) [71]. 

Ligand efficiency-dependent lipophilicity (LELP) is a global descriptor involving lipophilicity, molecular size and potency. The optimal range of LELP values is −10 to 10 and corresponds to a range of Log P values of −3 to 3. The values of the test compounds are within the optimal range. Taking all the parameters evaluated into account, the two series of 2-amino-3-cyano-4*H*-chromenes can be considered as drug candidates [72].

The analysis of the data (Tables S1 and S2) demonstrates that all the 2-amino-3-cyano-4*H*-chromenes in the two series, **4a–o** and **6a–h**, comply with Lipinski’s rule of five (RO5) by meeting the following requirements: (a) molecular weight <500 g/mol; (b) <5 hydrogen bond donors (NH, OH or SH); (c) <10 hydrogen bond acceptors; and (d) Log P < 4.15 [47]. Hence, the compounds should have efficient absorption and good oral bioavailability [73]. 

The drug-like properties were also determined [74,75], predicting good lipid solubility for the present 2-amino-3-cyano-4*H*-chromenes based on WLog P in the range of −0.4 < WLog P < 5.6 and PSA < 131. The steric descriptors of the test compounds, like those of topotecan (**7**), are within the desirable values: (a) 120 < molecular weight < 480, (b) 40 < molar refractivity < 130, and (c) the number of rings <7 [74,75]. According to the results, 2-amino-3-cyano-4*H*-chromenes **4a–o** and **6a–h** should have good absorption and an appropriate steric volume. Because of complying with the acceptable range of values of key parameters established for screening compounds in the drug development process, **4a–o** and **6a–h** can be considered drug candidates.

Regarding the pharmacokinetic properties (Tables S3 and S4), all the test compounds are characterized by good gastrointestinal absorption, like **7**, thus allowing for oral administration. Most should be able to cross the blood–brain barrier and reach targets in the central nervous system. However, this is not the case for the compounds with a greater number of polar functional groups, such as **4e**, **4k** and **6e** (R = CN), **4f**, **4o** and **6f** (R = NO_2_), and **4h**, **6h** (R = OMe), due to their strong inductive effect.

Log Kp (Tables S3 and S4) is a parameter that indicates the ability of a compound to pass through the skin, with a more negative value translating into greater permeability. In both series, the derivatives with a substituent R = CN (**4e**, **4k** and **6e**) and R = NO_2_ (**4f**, **4o** and **6f**) gave more negative values, while those with a substituent R = Cl (**4c**, **4m** and **6c**) and R = Me (**4g** and **6g**) had less negative values and therefore a lower permeability [76]. Additionally, the capacity of **4a–o** and **6a–h** to cross membranes was evaluated in relation to two types of transporters: the P450 cytochrome enzyme superfamily (CYP) and the ATP binding transporters (P-gp substrate). Almost all the compounds in the **4a–o** series are substrates of CYP transporters, allowing them to be metabolized and excreted by these transporters (Tables S3 and S4). Similarly, most compounds in the **6a–h** series bind to all CYP transporters. No affinity was shown for CYP2D6 by **6c–e** (with substituents R = Cl, R = Br and R = CN). Nevertheless, their excretion can be favored because they have affinity for the other four CYP transporters (CYP1A2, CYP2C19, CYP2C9 and CYP3A4). For its part, topotecan (**7**) is a substrate of the ATP binding transporters (P-gp substrate), leading to its elimination through the membrane.

The possible risk of toxicity was assessed for both series of compounds, **4a–o** and **6a–h** (Tables S5 and S6). There was no evidence of tumorigenicity, mutagenicity, irritation, or reproductive effects, with the exception of the reproductive effects found for **4h** and **6h**. The results for the rest of the compounds demonstrate an ample margin of safety between the effective dose and any serious risk to human health.

### 3.3. Multiple Sequence Alignment of CYP51 Enzymes of Candida spp.

Multiple sequence alignment was performed for CYP51 enzymes of *Candida* spp. with the crystallized CYP51SC enzyme sequence [57] (Figure S55). A large proportion of the amino acid residues in these enzymes proved to be highly conserved, especially the FGGGRHRCIG sequence motif. Within this sequence, a cysteine residue involved in binding the heme iron is conserved in the C-terminal of CYP51s [77]. Additionally, two conserved arginine residues are in the fifth and seventh positions of the motif and surround a histidine residue [78]. Other amino acids that are part of the active site of the CYP51 enzymes are Tyr118 and Thr311, which have been identified in *C. albicans*. Due to the aforementioned characteristics, the CYP51 protein family of yeast enzymes represents an excellent model for the study of new inhibitors with possible antifungal activity [79].

### 3.4. Generating 3D Models of CYP51 Enzymes of Candida spp.

Fifteen models of the CYP51 enzyme of each *Candida* species were provided by homology modeling, selecting the best of the fifteen based on its discrete optimized protein energy (DOPE) score. The overlap of the CYP51 models with the *S. cerevisiae* template demonstrated an identity of over 60% (Figure S56). CYP51CG displayed the highest value of identity. All the models obtained were evaluated, graphing their DOPE potential compared to the template for *S. cerevisiae* (CYP51SC, PDB 4WMZ) [57]. The graphs reveal a high degree of similarity between these structures (Figures S57–S62). The validation of the three-dimensional (3D) models was determined by compatibility with their own 1D amino acid sequence. In all the models analyzed, over 80% of the residues have an average 3D-1D score ≥0.2 (Figures S63–S68). Ramachandran plots were also created for each model (Figures S69–S74), showing over 90% of the amino acid residues in the most favorable regions. Moreover, the models proved to be of good quality, as in previous studies by our group [80].

### 3.5. Molecular Docking Studies

#### 3.5.1. Molecular Docking of 2-Amino-3-cyano-4*H*-chromenes **4a–o** and **6a–h** and Fluconazole (**8**) with CYP51 from *Candida* spp.

**Figure 1:**
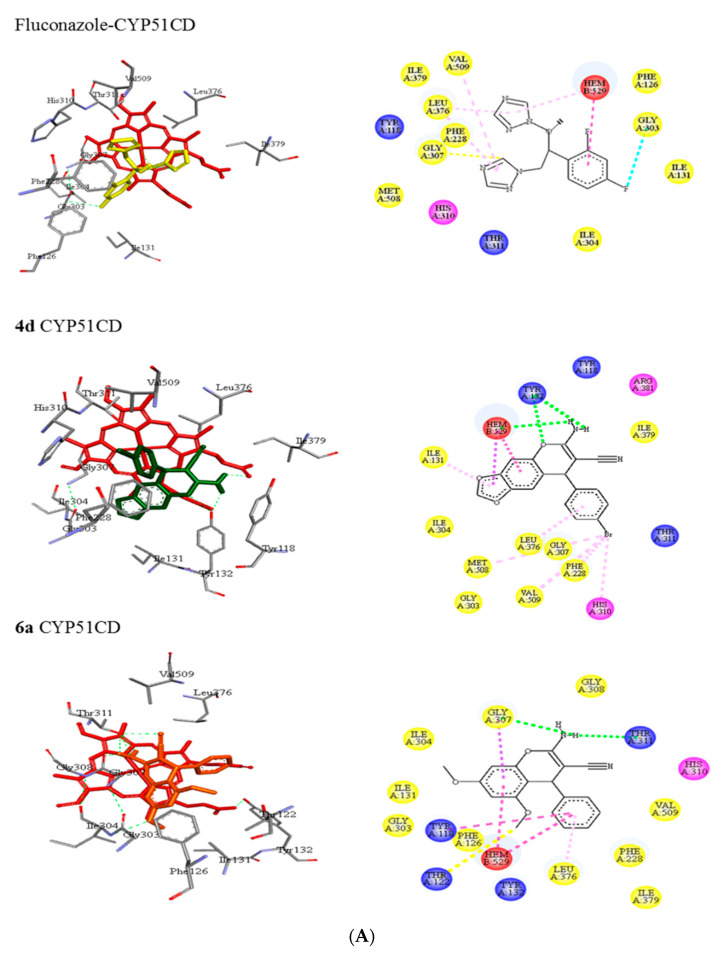

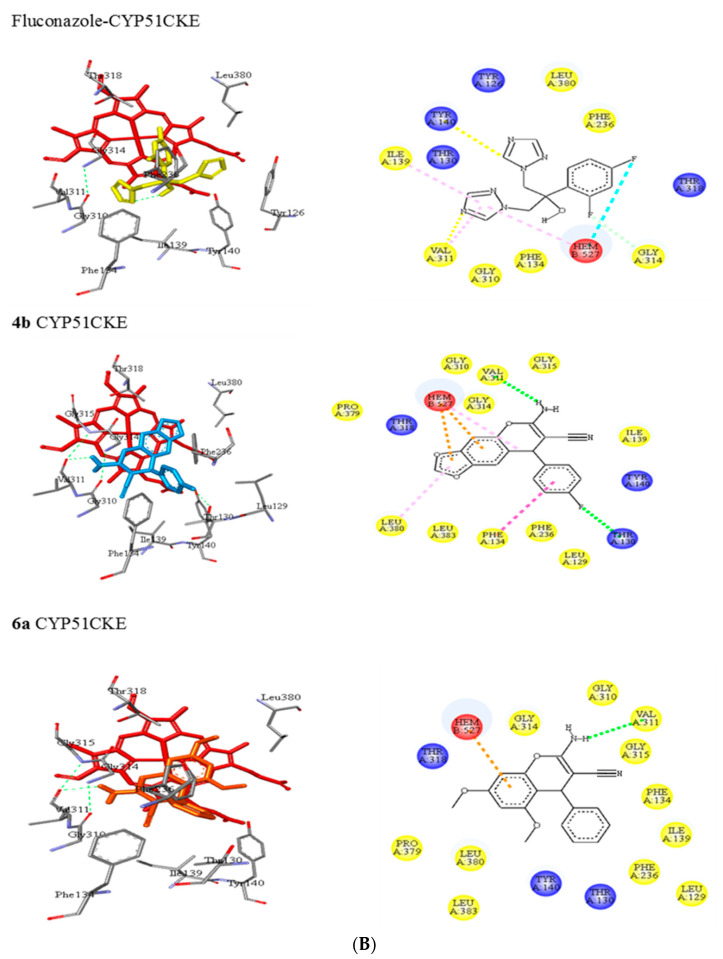
Schematic representation of the following binding modes: (**A**) **4d**, **6a**, and **8** at the active site of CYP51CD (*C. dubliniensis*), and (**B**) **4b**, **6a** and **8** at the active site of CYP51CKE (*C. kefyr*). Here, 3D and 2D models are shown. The 3D model depicts the hydrophilic bonds and amino acid residues that are part of the active site of CYP51. In the 2D model, the interactions denoted with dotted lines are conventional hydrogen bonds (green), carbon–hydrogen (yellow), π–cation (orange), π–sigma (purple), π–alkyl (pink), π–π T-shaped (fuchsia), and halogen (cyan). The heme group is in red. The solvent accessible surface is illustrated for the amino acid residues and ligands. The amino acids are highlighted in pink (basic), yellow (hydrophobic), and blue (hydrophilic).

Docking simulations were carried out for the two series of 2-amino-3-cyano-4*H*-chromenes, **4a–o** and **6a–h**, at the active site of the CYP51 of *Candida* spp. Some of these compounds with high binding energy values and the reference drug **8** are illustrated in (Figure 1 and Figure S139–S150), bound to the active site of the CYP51 enzyme (the heme group). As has been previously reported with azole derivatives [81,82], series **4a–o** and **6a–h** all display the same binding mode. This suggests that the CYP51 of yeasts could be a molecular target of these derivatives.

The orientation in relation to each of the CYP51 enzymes herein tested was determined for the 2-amino-3-cyano-4*H*-chromenes **4d** and **6a**, as well as fluconazole (**8**) (Figures S151–S156). Many of the derivatives exhibited a similar orientation within the active site.

With molecular docking, the binding energy was determined for each of the derivatives (Table S7), finding better values (ranging from −9.09 to −10.88 Kcal/mol) for the 2-amino-3-cyano-4*H*-chromenes than the reference drug **8**. Among all the test compounds, the best one (in bold type, Table S7) bound in a way notably more akin to the species of *Candida*, which suggests that the nature of the substituent influences the binding mode. For example, the presence of the electron withdrawing halogen substituents, R = Cl and R = Br in **4m** and **4n**, as well as R = CN in **4k**, favors interaction with the active site of the enzymes CYP51CD and CYP51CKE, respectively. 

In the series **4a–o**, derivative **4m** (with R = Cl on the phenyl ring) exhibits the best binding energy with the CYP51 of three *Candida* species (*C. albicans*, *C. glabrata* and *C. kefyr*). In the series **6a–h**, a similar result is found for **6d** (with R = Br on the phenyl ring). Accordingly, the substituents R = Me and R = Br could be involved in the inhibitory effect on the yeasts (vide infra). The highest binding energy shown by most of the 2-amino-3-cyano-4*H*-chromenes **4a–o** (−10.83 kcal/mol) and **6a–h** (−10.25 kcal/mol) was in relation to the CYP51 enzyme of *C. kefyr*.

Regarding the binding of the two series of 2-amino-3-cyano-4*H*-chromenes (**4a**–**o** and **6a**–**h**) and fluconazole (**8**) with the CYP51 enzymes of *Candida* spp., the amino acid residues involved, the binding mode, and the protein–ligand interactions are shown (Table 5 and Table S8). The predominant amino acids in the interactions between the compounds and CYP51 are hydrophobic in nature (Ile, Phe, Gly, Leu, Val and Met). They frequently establish π–alkyl, π–π T-shaped and π–sigma interactions, which were found presently and have been previously reported in relation to the reference drug **8** [83].

Most ligand–protein interactions include Tyr and Thr, hydrophilic amino acid residues that form carbon–hydrogen and conventional hydrogen bonds. Tyr118 and Tyr132 are observed in three of the *Candida* species (*C. albicans*, *C. dubliniensis* and *C. parapsilosis*), and they have influence in the interaction of the CYP51 of *C. albicans* with other azole derivatives [60]. As additional evidence of the importance of these amino acid residues in the activity of CYP51 of *Candida* spp., their mutation is known to cause yeast resistance mechanisms to the inhibitory effects of azoles [84,85].

Three amino acids with basic side chains, His, Arg and Lys, also greatly influence the mechanism of action of CYP51. The most frequent positions for such interactions are 310, 312, 372, 462, 463 and 468 for His, 379 and 381 for Arg, and 143 for Lys. In this research, as well as in previous studies, we corroborated that the following amino acids: Tyr118, Phe145, Ile231, Met306, His310, Thr311, Ser378, Val509 and Val511 are involved in the active site of CYP51 of *C. albicans* [80,83,86].

According to the present findings, the 2-amino-3-cyano-4*H*-chromenes **4a–o** and **6a–h** could possibly be administered to target the CYP51 enzymes of many *Candida* species, given that they bind to the active site. Hence, further research is warranted on their antifungal activity.

#### 3.5.2. Molecular Docking of 2-Amino-3-cyano-4*H*-chromenes **4a–o**, **6a–h** and Topotecan (**7**) on Topoisomerase I

A molecular docking study was carried out to determine the affinity of **4a–o** and **6a–h** with the active site of the topoisomerase I enzyme. Some key amino acids of the active site of the enzyme that are known to interact with **7** (the most common topoisomerase I inhibitor) also interact with **4a–o** and **6a–h**. Among such residues are Arg364, Arg488, Lys532, Asp533, Ile535, His632 and Thr718. Additionally, both series of 2-amino-3-cyano-4*H*-chromenes interact with certain DNA fragments, including TGP11, DG12 and DT10 [87,88]. Better binding energies were found for **4a–o** (Table 6) than **6a–h** (Table S9), a finding in agreement with the results of the biological activity tests, in which **4a–o** were more active than **6a–h** (see Section 3.7).

Compounds **4a**–**d** and **4g** showed the greatest affinity to the active site of the protein (−8.42 to −10.05 kcal/mol). Indeed, the binding energy was higher for **4c**, **4d** and **4g** than **7** (−9.86, −10.05 and −9.75 kcal/mol, respectively, vs. −9.56 kcal/mol). Like **7**, these three compounds interact with most of amino acid side chain of the active site of topoisomerase I.

For **4g** and **7**, there was a similar π–alkyl hydrophobic coupling with the TGP11 nucleotide of the complex. With a binding energy of −9.27 kcal/mol, **4b** displayed most of the same interactions as **4c**, **4d** and **4g**. Although the binding energy was lower (−8.42 Kcal/mol) for **4a**, this compound also exhibited similarities with the binding interactions of **7**, such as hydrophilic interactions with the TGP11 DNA fragment and π–anion electrostatic interactions with the residue Asp533.

In addition, we carried out the docking study with the series of enantiomers of the compounds that showed greater cytotoxic activity **4a–c**, **4g**, as well as of the compounds **4j–o**, where we can observe a greater affinity shown by the *S* series (−7.66 to −10.0 kcal/mol) for the active site of the enzyme topoisomerase I with respect to the *R* series (−7.37 to −8.37 kcal/mol) (Table S10). Moreover, we can observe that both series of enantiomers share key amino acids of the active site of the enzyme and DNA fragments just like topotecan.

In the series of 2-amino-3-cyano-5,7-dimethoxy-4-aryl-4*H*-chromenes **6a–h**, the best binding energies were found for **6a**, **6c** and **6d** (−8.42 to −9.10 kcal/mol; Table S9). Moreover, **6a–f**, **6h** and **7** each have conventional hydrogen bond and carbon–hydrogen bond interactions with DNA fragments, including TGP11 and/or DG12, and these compounds share interactions with Arg364, Asp533 and Thr718 in the active site of the enzyme.

**Figure 2:**
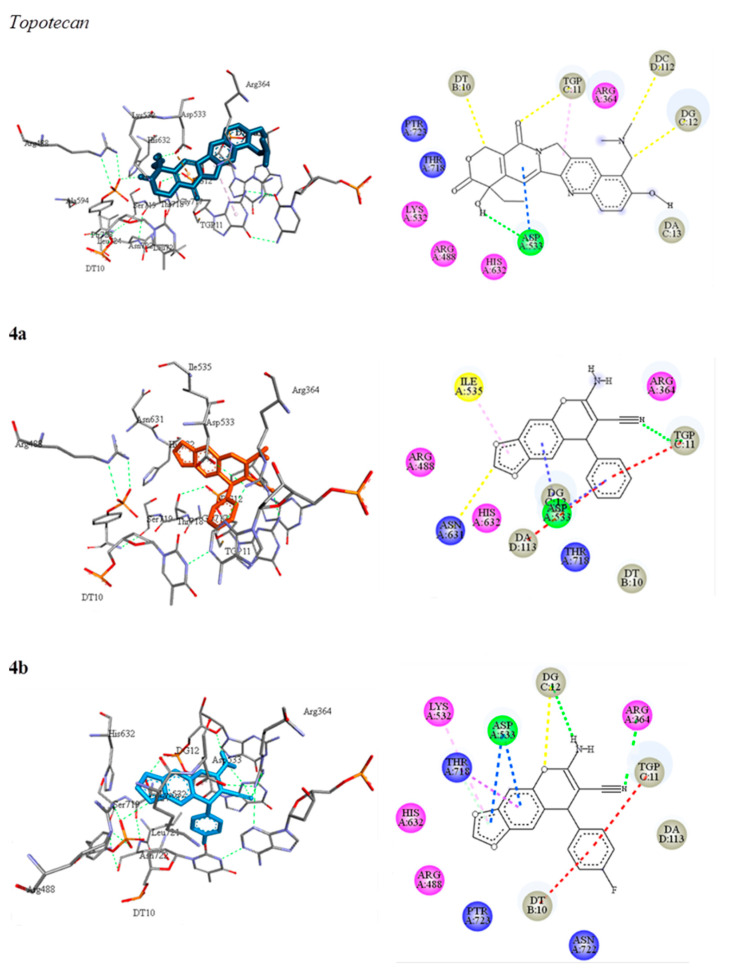

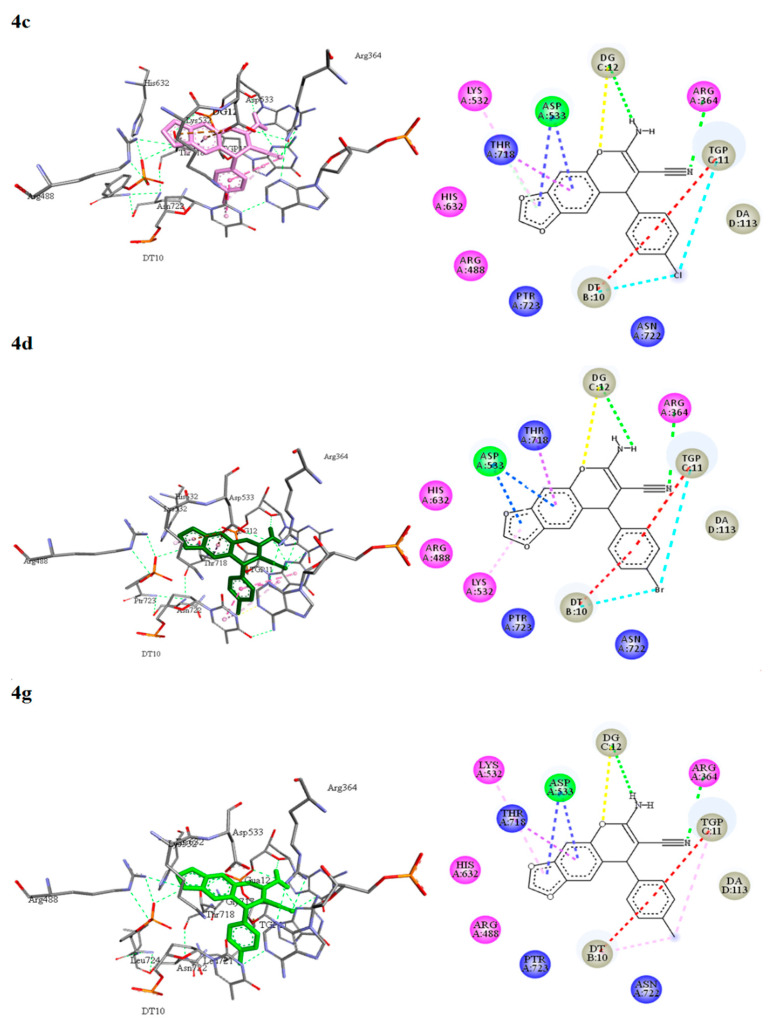
Schematic representation of the binding mode of 2-amino-3-cyano-6,7-methylendioxy-4*H*-chromenes **4a**, **4b**, **4c**, **4d** and **4g** and topotecan (**7**) at the active site of topoisomerase I. The 3D models portray the hydrophilic bonds and amino acid residues found in the active site of the enzyme. In the 2D models, the following types of interactions are denoted with dotted lines: conventional hydrogen bond (green), carbon–hydrogen (yellow), π–cation (orange), π–anion (blue), π–sigma (purple), π–alkyl (pink), π–π-stacked (red), π–π T-shaped (fuchsia), and halogen (cyan). The solvent accessible surface is illustrated for the amino acid residues and ligands. The amino acids are depicted in pink (basic), green (acidic), yellow (hydrophobic) and blue (hydrophilic). The DNA residues appear in gray.

The structures of **4a**, **4b**, **4c**, **4d**, **4g** and **7** display π–anion electrostatic interactions with the amino acid side chain residue Asp533 in the active site of the enzyme (Figure 2). For **4b**, **4c**, **4d** and **4g**, there are additional linkages not shared by **7**, such as a π–sigma interaction with the side chain amino acid Thr718. For **4b**, **4c** and **4d**, π–π stacked interactions were observed with the DNA residues DT10 and TGP11, and for 4g with TGP11 and DA113. Furthermore, conventional hydrogen bonds were found between the Arg364 residue and the nitrogen atom of the cyano group of **4b**, **4c**, **4d** and **4g**. These same four chromenes and **7** share important hydrophilic interactions (involving carbon–hydrogen bonds) with the DNA fragment DG12. Overall, the 4*H*-chromene derivatives and **7** act in a very similar manner on the active site of topoisomerase I, revealing that they may all have the same mechanism of action. The interactions of the 2-amino-3-cyano-4*H*-chromenes **4e–f**, **4h–i** and **6a–h** are illustrated in Figures S93 and S94.

The binding mode was analyzed for **7** and **4a**–**o** at the active site of topoisomerase I (Figures S95 and S96). There were three binding orientations adopted by the 2-amino-3-cyano-4*H*-chromenes within the active site. One orientation is shared by **4a**, **4b**, **4c** and **4g** (Figure S95B), and a distinct one by **4e**, **4f** and **4h** (Figure S96). Finally, the conformations of **4a** and **4i** were similar to one another, but slightly different from the aforementioned groups.

The binding mode of the structures of 2-amino-3-cyano-5,7-dimethoxy-4*H*-chromenes **6a–h** and reference drug **7** at the active site of the topoisomerase I enzyme (3D representation, Figure S97A) indicates that **6d** and **6f** are oriented in an identical manner, and that **6c**, **6b** and **6h** adopt a conformation distinct from the former group, but similar among themselves (Figure S97B).

Overall, the two chromene series **4a–o** and **6a–h** have interactions very similar to **7** at the binding site of the topoisomerase I enzyme, and therefore are likely to have the same mechanism of action.

### 3.6. Antifungal Activity

Given the good binding energy found for the 2-amino-3-cyano-4*H*-chromenes **4a**–**i** and **6a**–**h** with the CYP51 enzymes, the antifungal activity of the compounds was explored experimentally. When tested against *Candida* spp., both series of compounds exhibited a MIC_50_ less than or equal to that of the reference drug **8** (0.063–0.5 µg/mL vs. 0.50–4.0 µg/mL; Table 7). Thus, the experimental and docking data are in agreement, considering the better binding energy of the derivatives than **8**.

For the 2-amino-3-cyano-4*H*-chromenes currently evaluated in relation to antifungal activity against six *Candida* species, the MIC values were similar to or lower than those described in previous reports for some 2-amino-3-cyano-4*H*-chromenes tested on *C. albicans*, *Aspergillus niger*, *A. fumigatus*, *A. flavus* and *S. cerevisiae* [15,89]. Since scant research has been carried out on *Candida* species, the inhibitors **4a**–**i** and **6a**–**h** were presently assayed against different species of this genus. The ability of these two series of chromenes to bind to the CYP51 enzyme and their strong inhibitory effect on the six *Candida* species herein tested suggest that both series, **4a**–**i** and **6a**–**h**, are potential antifungals.

### 3.7. Cytotoxicity Assay

In order to evaluate the cytotoxic activity of compounds, we carried out the determination at 25 μM for **4a–i** and **6a–h** in six tumor cells: U251 (human glioblastoma), PC-3 (human prostatic adenocarcinoma), K-562 (human chronic myelogenous leukemia), HCT-15 (human colorectal adenocarcinoma), MCF-7 (human mammary adenocarcinoma), and SKLU-1 (human lung adenocarcinoma). However, only four compounds (**4a**, **4b**, **4c** and **4g**) showed an inhibition percentage greater than 50% in the SK-LU-1 and PC-3 cell lines. More detailed studies on the action of these compounds are shown in Supplementary material1.

## 4. Conclusions

Two series of 2-amino-3-cyano-4*H*-chromenes, **4a**–**o** and **6a**–**h**, were synthesized through an efficient and environmentally friendly methodology. This is the first report, to our knowledge, of **4d**, **e**, **i–o**, as well as **6b**, **d** and **e**. Regarding the evaluation of the physicochemical, pharmacokinetic, toxicological and drug-like properties of the test compounds, the values of key parameters fall within the acceptable range established for screening compounds in the drug development process. Moreover, the in silico data revealed an ample margin of safety between the effective dose of the series of compounds **4** and **6** and any possible toxicity.

The molecular docking studies provided evidence that **4a**–**o**, **6a**–**h**, and fluconazole (**8**) (the reference drug) interact with the active site of the CYP51 enzymes of *C. albicans*, *C. dubliniensis*, *C. glabrata*, *C. kefyr*, *C. krusei* and *C. parapsilosis*. CYP51 is vital for the production of ergosterol in the yeast cell membrane. The test compounds appear to have the same mechanism of action as the reference drug, since they interact with the same amino acids in the active site of the CYP51 enzyme. Better in silico binding energy was found for **4a**–**o** and **6a**–**h** than **8** on this enzyme, and the in vitro MIC_50_ of the test compounds was less than or equal to that of **8** (0.063–0.50 vs. 0.5–4.0 µg/mL, respectively).

According to the results of molecular docking on topoisomerase I (involved in the growth of cancer cells), **4a**–**o** and **6a**–**h** interact with the same amino acids of the active site of the enzyme as one of the reference drugs **7**. Compounds **4c**, **4d** and **4g** showed better binding energy than **7**. The experimental assays revealed that **4a–o** were more cytotoxic than **6a–h**. Furthermore, **4a** and **4b** were more active than topotecan (**7**) and cisplatin (**9**). This is the first report, to our knowledge, of molecular docking studies on 2-amino-3-cyano-4*H*-chromenes **4a**–**o** and **6a**–**h** with the active site of CYP51 of *Candida* spp., and topoisomerase I. Due to the encouraging results, in which some chromene compounds displayed better binding energy, inhibition and cytotoxicity than the reference compounds, the two series of derivatives are good candidates for future research on the design and development of new 2-amino-3-cyano-4*H*-chromenes, with dual activity against cancer and fungal infections.

## Figures and Tables

**Table 1:** Optimization of the reaction conditions for the synthesis of 2-amino-3-cyano-6,7-methylendioxy-4-(4-nitrophenyl)-4*H*-chromene **4f** ^a^.

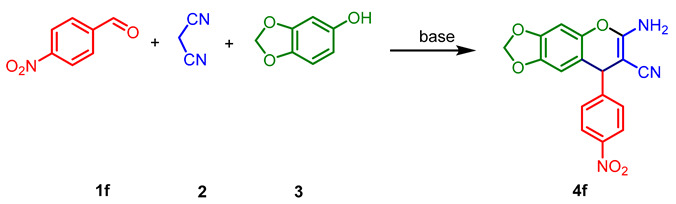					
Entry	Solvent	Base (Mol Equiv)	Temperature (°C)	Time (h)	Yield (%) ^b^
1	EtOH	Et_3_N (0.2)	rt	20	36
2	EtOH	piperidine (0.2)	rt	20	64
3	EtOH	K_2_CO_3_ (0.2)	rt	20	12
4	EtOH	piperidine (2.0)	rt	20	55
5	H_2_O	piperidine (0.2)	rt	20	34
6	dioxane	piperidine (0.2)	rt	20	16
7	MeCN	piperidine (0.2)	rt	20	50
8	EtOH	piperidine (0.2)	78	4	42

^a^ All reactions were carried out with an equimolecular mixture of **1f**, **2** and **3**. ^b^ Isolated yield after purification by column chromatography.

**Table 2:** Synthesis of 2-amino-3-cyano-4*H*-chromenes **4a–o** and **6a–h** ^a^.

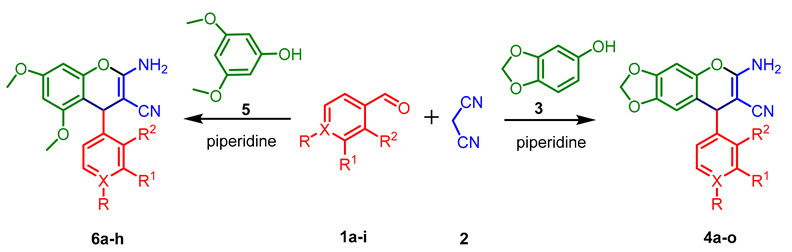								
Entry	Aldehyde	R	R^1^	R^2^	X	Phenol	Product	Yield (%) ^b^
1	**1a**	H	H	H	C	**3**	**4a**	74
2	**1b**	F	H	H	C	**3**	**4b**	64
3	**1c**	Cl	H	H	C	**3**	**4c**	61
4	**1d**	Br	H	H	C	**3**	**4d** ^c^	71
5	**1e**	CN	H	H	C	**3**	**4e** ^c^	68
6	**1f**	NO_2_	H	H	C	**3**	**4f**	64
7	**1g**	Me	H	H	C	**3**	**4g**	84
8	**1h**	OMe	H	H	C	**3**	**4h**	48
9	**1i**	H	H	H	N	**3**	**4i** ^c^	55
10	**1j**	H	F	H	C	**3**	**4j** ^c^	57
11	**1k**	H	CN	H	C	**3**	**4k** ^c^	70
12	**1l**	H	H	F	C	**3**	**4l** ^c^	84
13	**1m**	H	H	Cl	C	**3**	**4m** ^c^	51
14	**1n**	H	H	Br	C	**3**	**4n** ^c^	68
15	**1o**	H	H	NO_2_	C	**3**	**4o** ^c^	70
16	**1a**	H	H	H	C	**5**	**6a**	57
17	**1b**	F	H	H	C	**5**	**6b** ^c^	45
18	**1c**	Cl	H	H	C	**5**	**6c**	43
19	**1d**	Br	H	H	C	**5**	**6d** ^c^	47
20	**1e**	CN	H	H	C	**5**	**6e** ^c^	46
21	**1f**	NO_2_	H	H	C	**5**	**6f**	45
22	**1g**	Me	H	H	C	**5**	**6g**	44
23	**1h**	OMe	H	H	C	**5**	**6h**	30

**^a^** All reactions were carried out at room temperature for 20 h with an equimolecular mixture of aryl aldehyde, 2 and phenol in ethanol, using piperidine (0.2 mol equiv) as the base. ^b^ Isolated yield after purification by column chromatography. ^c^ Previously unreported compounds.

**Table 3:** Calculated physicochemical properties of 2-amino-3-cyano-4*H*-chromenes **4a–o** and topotecan **7**.

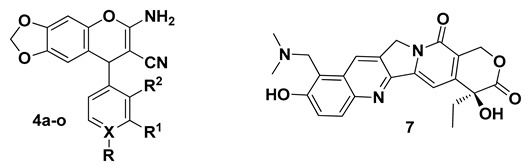													
Compound	X	R	R^1^	R^2^	MW	Log P	Log S	PSA	LE	LLE	LELP	H-A	H-D
					(g/mol)								
**4a**	C	H	H	H	292.293	2.5	−3.95	77.5	0.407	4.041	6.118	5	1
**4b**	C	F	H	H	310.283	2.82	−4.11	77.5	0.388	3.914	6.682	6	1
**4c**	C	Cl	H	H	326.738	3.09	−4.54	77.5	0.386	3.386	8.011	5	1
**4d**	C	Br	H	H	371.189	3.21	−4.86	77.5	0.383	3.212	8.391	5	1
**4e**	C	CN	H	H	317.303	2.83	−3.89	101.29	0.371	4.169	6.269	6	1
**4f**	C	NO_2_	H	H	337.29	1.76	−4.01	123.32	0.355	6.627	−0.437	8	1
**4g**	C	Me	H	H	306.32	2.837	−4.25	77.5	0.388	3.676	7.302	5	1
**4h**	C	OMe	H	H	322.319	2.5	−4.02	86.73	0.371	4.068	6.53	6	1
**4i**	N	-	H	H	293.276	1.77	−3.28	90.39	0.407	5.04	3.663	6	1
**4j**	C	F	H	H	310.283	2.81	−4.11	77.5	0.512	5.992	5.065	5	1
**4k**	C	H	CN	H	317.303	2.27	−3.89	101.29	0.371	4.169	6.269	6	1
**4l**	C	H	H	F	310.283	2.81	−4.11	77.5	0.388	3.914	6.682	5	1
**4m**	C	H	H	Cl	326.738	3.03	−4.54	77.5	0.386	3.386	8.011	5	1
**4n**	C	H	H	Br	371.189	3.11	−4.86	77.5	0.383	3.212	8.391	5	1
**4o**	C	H	H	NO_2_	337.29	1.77	−4.01	123.32	0.355	6.627	−0.437	8	1
**7**	-	-	-	-	421.452	0.461	−1.959	103.2	0.377	8.061	1.223	8	2

MW, molecular weight; Log P, partition coefficient; Log S, water solubility; PSA, polar surface area; LE, ligand efficiency; LLE, ligand-lipophilicity efficiency; LELP, ligand efficiency-dependent lipophilicity; H-A, hydrogen-bond acceptors; H-D, hydrogen-bond donors.

**Table 4:** Calculated physicochemical properties of 2-amino-3-cyano-4*H*-chromenes **6a–h** and topotecan **7** as inhibitors of topoisomerase I.

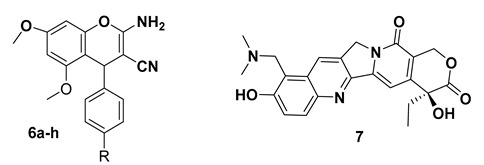										
Compound	R	MW	Log P	Log S	PSA	LE	LLE	LELP	H-A	H-D
		(g/mol)								
**6a**	H	308.336	2.65	−3.98	77.5	0.388	4.269	5.772	5	1
**6b**	F	326.326	2.96	−4.14	77.5	0.37	4.143	6.318	5	1
**6c**	Cl	342.781	3.19	−4.57	77.5	0.369	3.617	7.706	5	1
**6d**	Br	387.232	3.26	−4.89	77.5	0.366	3.445	8.095	5	1
**6e**	CN	333.346	2.43	−3.92	101.29	0.355	4.399	5.844	6	1
**6f**	NO_2_	353.101	1.92	−4.04	123.32	0.34	6.858	−1.194	8	1
**6g**	Me	322.363	2.99	−4.28	103.2	0.371	3.906	6.968	5	1
**6h**	OMe	338.362	2.65	−4.05	86.73	0.355	4.298	6.116	6	1
**7**	-	421.452	0.461	−1.959	103.2	0.377	8.061	1.223	8	2

MW, molecular weight; Log P, partition coefficient; Log S, water solubility; PSA, polar surface area; LE, ligand efficiency; LLE, ligand-lipophilicity efficiency; LELP, ligand efficiency-dependent lipophilicity; H-A, hydrogen-bond acceptors; H-D, hydrogen-bond donors.

**Table 5:** Of the two series, the compounds with the best binding energy were chosen, along with **8** to analyze their main interactions with CYP51 enzymes of *Candida* spp.

Compound	Interacting Residues	Interactions	
		Polar	Hydrophobic
**CD ^a^**	Tyr118, Phe126, Ile131, Phe228, Gly303, Ile304, Gly307, His310, Thr311, Leu376, Ile379, Met508, Val509	N^…..^H-O (Gly307)	halogen (Gly303) π-alkyl (Leu376) π-alkyl (Val509)
**8**			
**4d**	Tyr118, Ile131, Tyr132, Phe228, Gly303, Ile304, Gly307, His310, Thr311, Leu376, Ile379, Arg381, Met508, Val509	N-H^…..^O (Tyr132)	π-alkyl (Ile131) π-alkyl (Phe228) π-alkyl (Gly307) π-alkyl (His310) π-alkyl (Leu376) π-alkyl (Met508) π-alkyl (Val509)
**6a**	Tyr118, Thr122, Phe126, Ile131, Tyr132, Phe228, Gly303, Ile304, Gly307, Gly308, His310, Thr311, Leu376, Ile379, Val509	C-H^…..^O (Thr122) N-H^…..^O (Gly307) N^…..^H-O (Thr311)	π-π T-shaped (Tyr118) π-sigma (Gly307) π-alkyl (Leu376)
**CKE ^a^**	Tyr126, Thr130, Phe134, Ile139, Tyr140, Phe236, Gly310, Val311, Gly314, Thr318, Leu380	C-H^…..^O (Tyr140) N^…..^C-H (Val311)	π-alkyl (Ile139) π-alkyl (Val311)
**8**			
**4b**	Leu129, Thr130, Phe134, Ile139, Tyr140, Phe236, Gly310, Val311, Gly314, Gly315, Thr318, Pro379, Leu380, Leu383	N-H^…..^O (Val311)	π-π T-shaped (Phe134) π-alkyl (Leu380)
**6a**	Leu129, Thr130, Phe134, Ile139, Tyr140, Phe236, Gly310, Val311, Gly314, Gly315, Thr318, Pro379, Leu380, Leu383	N-H^…..^O (Val311)	-
**6d**	Tyr126, Ile139, Tyr140, Phe231, Gly305, Val306, Gly309, Gly310, His312, Thr313, Leu376, Ile379, His473, Met510, Val511	N-H^…..^O (Tyr140) C-H^…..^O (Gly309)	π-alkyl (Ile139) π-alkyl (Val306) π-sigma (Leu376)
**CP ^a^**	Thr122, Phe126, Ile131, Tyr132, Gln142, Lys143, Phe228, Leu300, Gly303, Val304, Gly307, Gly308, Thr311	C-H^…..^O (Gly303) O^…..^H-C (Gly304)	π-sigma (Ile131) halogen (Gly303) halogen (Val304) halogen (Gly307)
**8**			
**4a**	Leu121, Thr122, Phe126, Ile131, Tyr132, Leu139, Gln142, Lys143, Phe228, Leu300, Gly303, Val304, Met306, Gly307, Gly308, Gln309, Thr311	N-H^…..^O (Val304) O^…..^H-C (Val304)	π-alkyl (Ile131) π-sigma (Ile131)
**4e**	Tyr118, Ile131, Tyr132, Phe228, Leu300, Gly303, Val304, Gly307, Gly308, His310, Thr311, Leu376, His462, Met502, Val503	N-H^…..^O (Tyr132) C-H^…..^O (Thr311) C-H^…..^N (Met502)	π-alkyl (Ile131)

**^a^ CD** (C. *dubliniensis*), CKE (C. *kefyr*), and CP (C. *parapsilosis*).

**Table 6:** Binding energy and interactions of the 2-amino-3-cyano-6,7-methylendioxy-4*H*-chromenes **4a–d** and **4g** and topotecan (**7**) at the active site of topoisomerase I (PDB entry no. 1SC7).

Compound	Binding Energy ΔG (Kcal/mol)	Interacting Residues	Interactions	
			Polar	Hydrophobic
**7**	−9.56	Arg364, Arg488, Lys532, Asp533, His632, Gly717, Thr718, Ser719, Ptr723. DT10, TGP11, DG12, DC112, DA113.	C-H^......^O (DT10) O^...^H-C (TGP11) C-H^....^N (DG12) C-H^..^O (DC112) O-H^..^O (Asp533)	π-alkyl (TGP11). π-anion (Asp533)
**4a**	−8.42	Arg364, Arg488, Asp533, Ile535, Asn631, His632, Thr718. DT10, TGP11, DG12, DA113.	N^......^H-N (TGP11) C-H^......^O (Asn631)	π-anion (Asp533) π-π stacked (TGP11, DA113)
**4b**	−9.27	Arg364, Arg488, Lys532, Asp533, His632, Thr718, Asn722, Ptr723. DT10, TGP11, DG12, DA113.	N-H^......^O (DG12) O^......^H-C (DG12) N^...^H-N (Arg364)	π-alkyl (Lys532). π-anion (Asp533) π-sigma (Thr718) π-π stacked (DT10, TGP11)
**4c**	−9.86	Arg364, Arg488, Lys532, Asp533, His632, Thr718, Asn722, Ptr723. DT10, TGP11, DG12, DA113.	N-H^......^O (DG12) O^......^H-C (DG12) N^......^H-N (Arg364)	π-alkyl (Lys532). π-anion (Asp533) π-sigma (Thr718) π-π stacked (DT10, TGP11) halogen (DT10, TGP11)
**4d**	−10.05	Arg364, Arg488, Lys532, Asp533, His632, Thr718, Asn722, Ptr723. DT10, TGP11, DG12, DA113.	N-H^......^O (DG12) O^......^H-C (DG12) N^......^H-N (Arg364)	π-alkyl (Lys532). π-anion (Asp533) π-sigma (Thr718) π-π stacked (DT10, TGP11) halogen (DT10, TGP11)
**4g**	−9.75	Arg364, Arg488, Lys532, Asp533, His632, Thr718, Asn722, Ptr723, DT10, TGP11, DG12, DA113.	N-H^......^O (DG12) O^......^H-C (DG12) N^......^H-N (Arg364)	π-alkyl (Lys532, DT10, TGP11). π-anion (Asp533) π-sigma (Thr718) π-π stacked (TGP11, DA113)

**Table 7:** Antifungal activity against *Candida* spp. by 2-amino-3-cyano-4*H*-chromenes **4a**–**i** and **6a**–**h** and fluconazole (**8**).

Compound	MIC_50_ (µg/mL)					
	CA ^a^	CD ^a^	CG ^a^	CKE ^a^	CK ^a^	CP ^a^
**8**	0.500	0.500	4.000	0.500	4.000	2.000
**4a**	0.125	0.250	0.063	0.125	0.125	0.500
**4b**	0.125	0.125	0.063	0.063	0.125	0.500
**4c**	0.250	0.063	0.125	0.063	0.125	0.125
**4d**	0.250	0.063	0.063	0.063	0.25	1.000
**4e**	0.125	0.063	0.125	0.125	0.125	0.250
**4f**	0.063	0.250	0.125	0.125	0.125	0.063
**4g**	0.25	0.125	0.063	0.125	0.250	0.250
**4h**	0.063	0.063	0.063	0.063	0.250	0.063
**4i**	0.125	0.250	0.125	0.063	0.250	0.063
**6a**	0.125	0.125	0.250	0.063	0.125	0.125
**6b**	0.125	0.063	0.250	0.063	0.500	0.063
**6c**	0.125	0.063	0.063	0.063	0.500	0.125
**6d**	0.063	0.063	0.063	0.063	0.125	0.063
**6e**	0.063	0.063	0.250	0.063	0.250	0.063
**6f**	0.125	0.063	0.250	0.125	0.250	0.250
**6g**	0.125	0.063	0.125	0.125	0.25	0.25
**6h**	0.125	0.063	0.125	0.125	0.125	0.125

**^a^** CA (C. *albicans*), CD (C. *dubliniensis*), CG (C. *glabrata*), CKE (C. *kefyr*), CK (C. *krusei*), and CP (C. *parapsilosis*).

## Data Availability

Data is contained within the article and supplementary material1.

## Supplementary Materials

The supplementary materials (Figures S1–S161, Tables S1–S13) are available online at https://www.mdpi.com/article/10.3390/ph14111110/s1.
