# Supplementary material for: Three-Component Synthesis of 2-Amino-3-cyano-4H-chromenes, In Silico Analysis of Their Pharmacological Profile, and In Vitro Anticancer and Antifungal Testing
Source: Pharmaceuticals (Basel). 2021 Oct 30;14(11):1110. doi: 10.3390/ph14111110 (PMC8623194; doi:10.3390/ph14111110)
Supplement: Supplementary file 1 [file pharmaceuticals-14-01110-s001.zip › pharmaceuticals-1388615-supplementary.pdf]

Supplementary Material

Three-Component Synthesis of 2-Amino-3-Cyano-4*H*-Chromenes, In Silico Analysis of Their Pharmacological Profile, and In Vitro Anticancer and Antifungal Testing

Alberto Feliciano [1,2], Omar Gómez-García [1,*], Carlos H. Escalante [1], Mario A. Rodríguez-Hernández [1], Mariana Vargas-Fuentes [1], Dulce Andrade-Pavón [3,4], Lourdes Villa-Tanaca [3], Cecilio Álvarez-Toledano [5], María Teresa Ramírez-Apan [5], Miguel A. Vázquez [2], Joaquín Tamariz [1] and Francisco Delgado [1,*]

1 Departamento de Química Orgánica, Escuela Nacional de Ciencias Biológicas, Instituto Politécnico Nacional, Prolongación de Carpio y Plan de Ayala S/N, Mexico City 11340, Mexico; a.feliciano@ugto.mx (A.F.); escalantecah@gmail.com (C.H.E.); mario.arctic.505@gmail.com (M.A.R.-H.); marianavargasme@gmail.com (M.V.-F.); jtamarizm@yahoo.com.mx (J.T.)

2 Departamento de Química, Universidad de Guanajuato, Noria Alta s/n, Guanajuato 36050, Mexico; mvazquez@ugto.mx

3 Laboratorio de Biología Molecular de Bacterias y Levaduras, Departamento de Microbiología, Escuela Nacional de Ciencias Biológicas, Instituto Politécnico Nacional, Prolongación de Carpio y Plan de Ayala S/N, Mexico City 11340, Mexico; andrade_eclud88@hotmail.com (D.A.-P.); mvillat@ipn.mx (L.V.-T.)

4 Departamento de Fisiología, Escuela Nacional de Ciencias Biológicas, Instituto Politécnico Nacional, Av. Wilfrido Massieu S/N, Mexico City 07738, Mexico

5 Instituto de Química-UNAM, Circuito Exterior, Ciudad Universitaria, Coyoacán, C.P., Ciudad de Mexico 04510, Mexico; cecilio@unam.mx (C.Á.-T.); mtrapan@unam.mx (M.T.R.-A.)

* Correspondence: jogomezga@ipn.mx or gogamanj@hotmail.com (O.G.-G.); jfdelgador@gmail.com (F.D.)

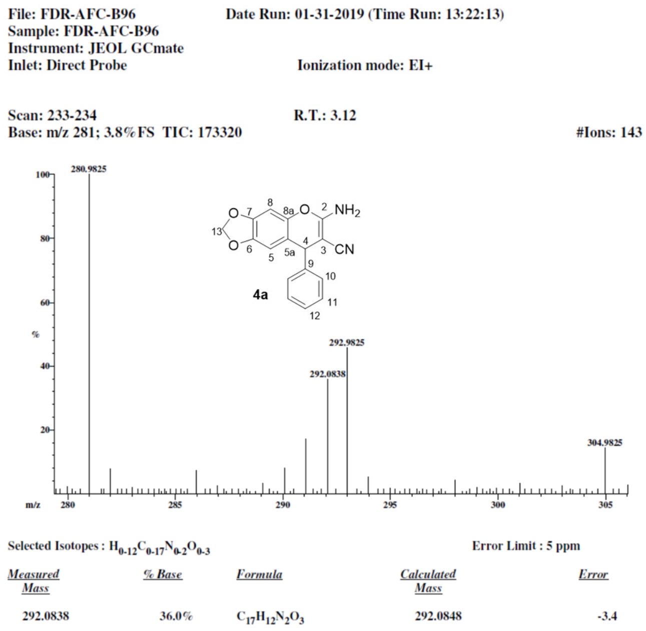

Figure S1. HRMS (EI) $[M]^+$ of 2-Amino-3-cyano-4-phenyl-6,7-methylendioxy-4*H*-chromene **4a**.

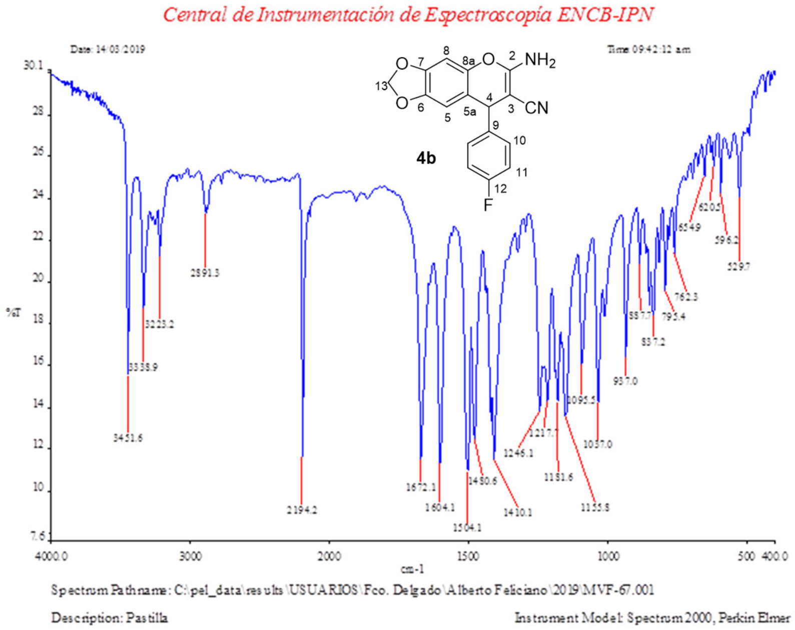

Figure S2. FT-IR of 2-Amino-3-cyano-4-(4-fluorophenyl)-6,7-methylendioxy-4*H*-chromene **4b**.

File: FDR-MVF-67 Date Run: 02-13-2019 (Time Run: 12:24:59)
Sample: FDR-MVF-67
Instrument: JEOL GCmate
Inlet: Direct Probe Ionization mode: EI+

Scan: 477 R.T.: 6.32
Base: m/z 310; 4%FS TIC: 282272 #Ions: 193

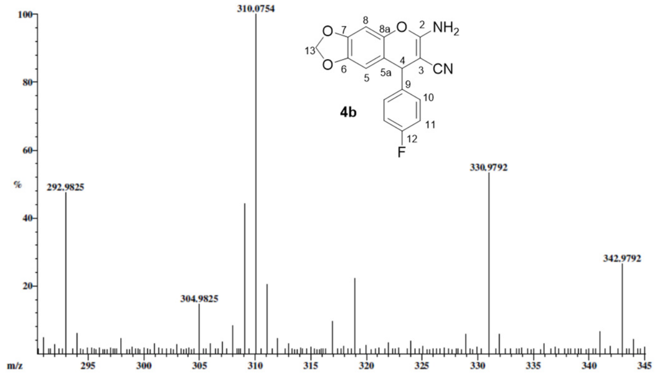

Selected Isotopes : $H_{0-11}C_{0-17}N_{0-2}O_{0-3}F_{0-1}$ Error Limit : 5 ppm

Measured Mass	% Base	Formula	Calculated Mass	Error
310.0754	100.0%	$C_{17}H_{11}N_2O_3F$	310.0754	0.1

Figure S3. HRMS (EI) $[M]^+$ of 2-Amino-3-cyano-4-(4-fluorophenyl)-6,7-methylendioxy-4*H*-chromene **4b**.

File: FDR-AFC-B97 Date Run: 01-31-2019 (Time Run: 16:32:58)
Sample: FDR-AFC-B97
Instrument: JEOL GCmate
Inlet: Direct Probe Ionization mode: EI+

Scan: 235 R.T.: 3.13
Base: m/z 326; 6.3%FS TIC: 354048 #Ions: 178

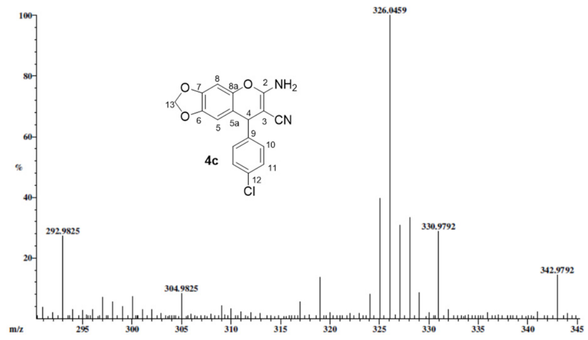

Selected Isotopes : $H_{0-11}C_{0-17}N_{0-2}O_{0-3}Cl_{0-1}$ Error Limit : 5 ppm

Measured Mass	% Base	Formula	Calculated Mass	Error
326.0459	100.0%	$C_{17}H_{11}N_2O_3Cl$	326.0458	0.2

Figure S4. HRMS (EI) $[M]^+$ of 2-Amino-3-cyano-4-(4-chlorophenyl)-6,7-methylendioxy-4*H*-chromene **4c**.

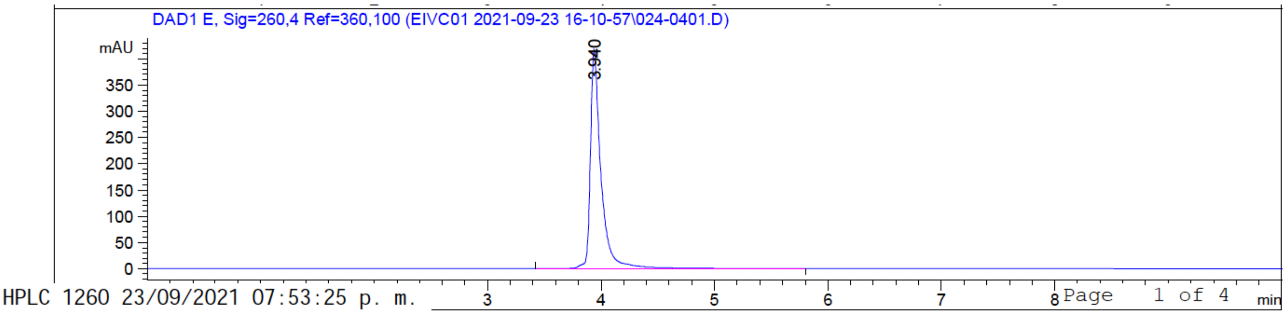

Signal 5: DAD1 E, Sig=260,4 Ref=360,100

Peak #	RetTime [min]	Type	Width [min]	Area [mAU*s]	Height [mAU]	Area %
1	3.940	BB	0.0947	2746.34375	417.65527	100.0000
Totals :				2746.34375	417.65527	

Figure S5. **HPLC** of -Amino-3-cyano-4-(4-chlorophenyl)-6,7-methylendioxy-4*H*-chromene **4c**. 0.1 mg/ml, MeCN, 0.4 ml/min, 260 nm, purity = 100%.

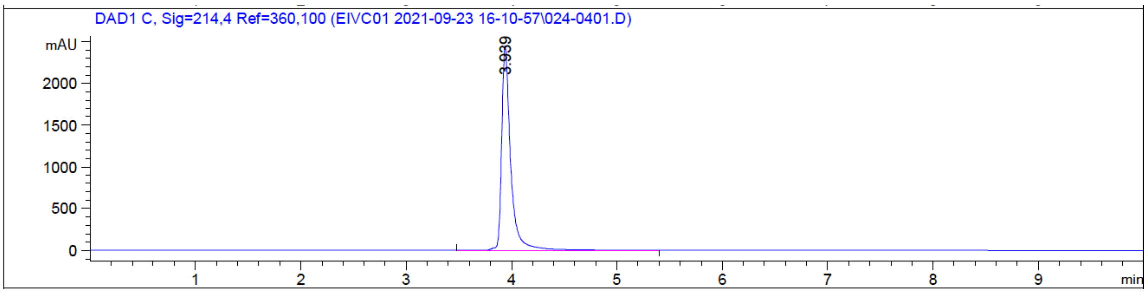

Signal 3: DAD1 C, Sig=214,4 Ref=360,100

Peak #	RetTime [min]	Type	Width [min]	Area [mAU*s]	Height [mAU]	Area %
1	3.939	BB	0.0878	1.46352e4	2445.02661	100.0000
Totals :				1.46352e4	2445.02661	

Figure S6. **HPLC** of -Amino-3-cyano-4-(4-chlorophenyl)-6,7-methylendioxy-4*H*-chromene **4c**. 0.1 mg/ml, MeCN, 0.4 ml/min, 214 nm, purity = 100%.

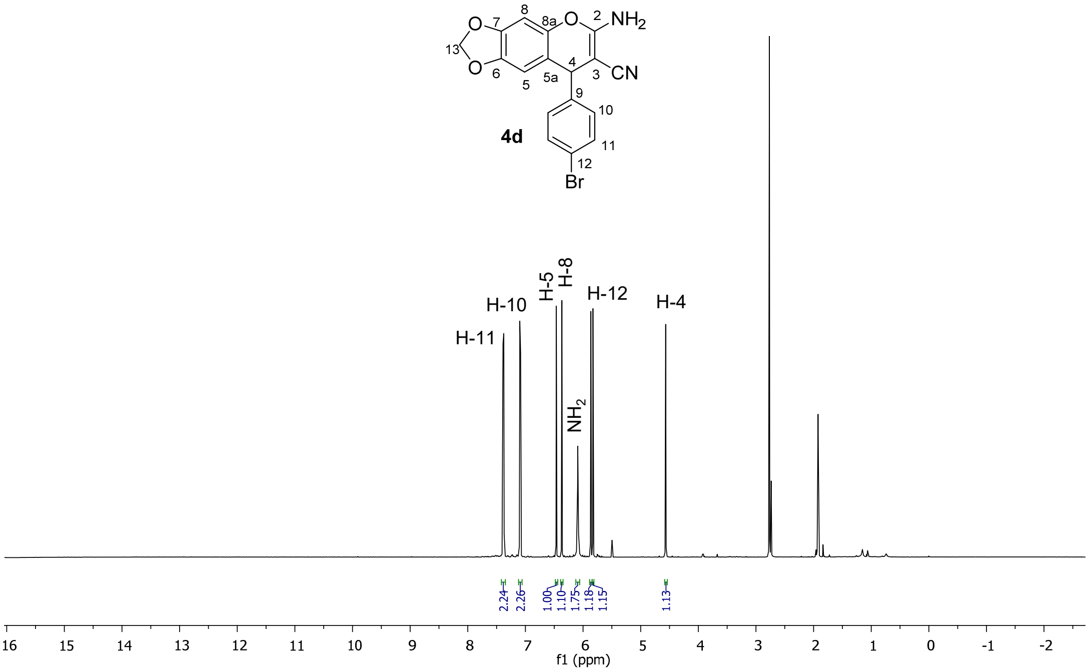

Figure S7. ^{1}H NMR (600 MHz, acetone d$_6$) of 2-Amino-4-(4-bromophenyl)-3-cyano-6,7-methylendioxy-4*H*-chromene **4d**.

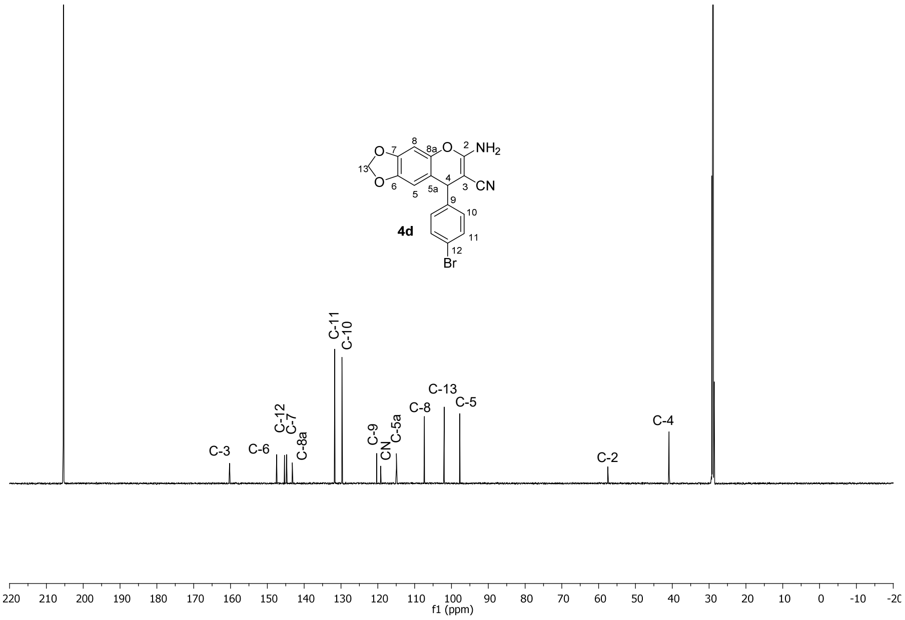

Figure S8. ^{13}C NMR (150 MHz, acetone d$_6$) of 2-Amino-4-(4-bromophenyl)-3-cyano-6,7-methylendioxy-4*H*-chromene **4d**.

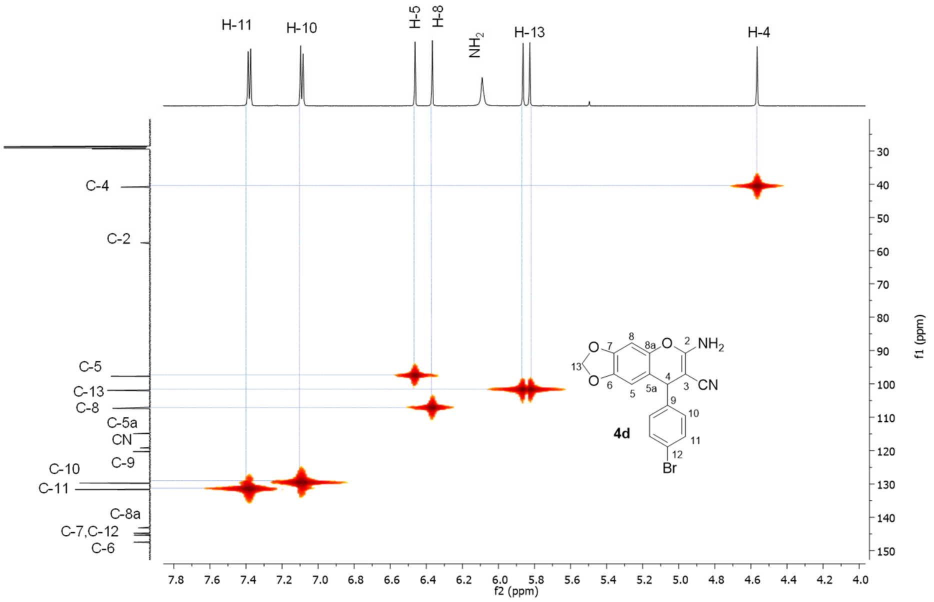

Figure S9. HMQC experiment of 2-Amino-4-(4-bromophenyl)-3-cyano-6,7-methylendioxy-4*H*-chromene **4d**.

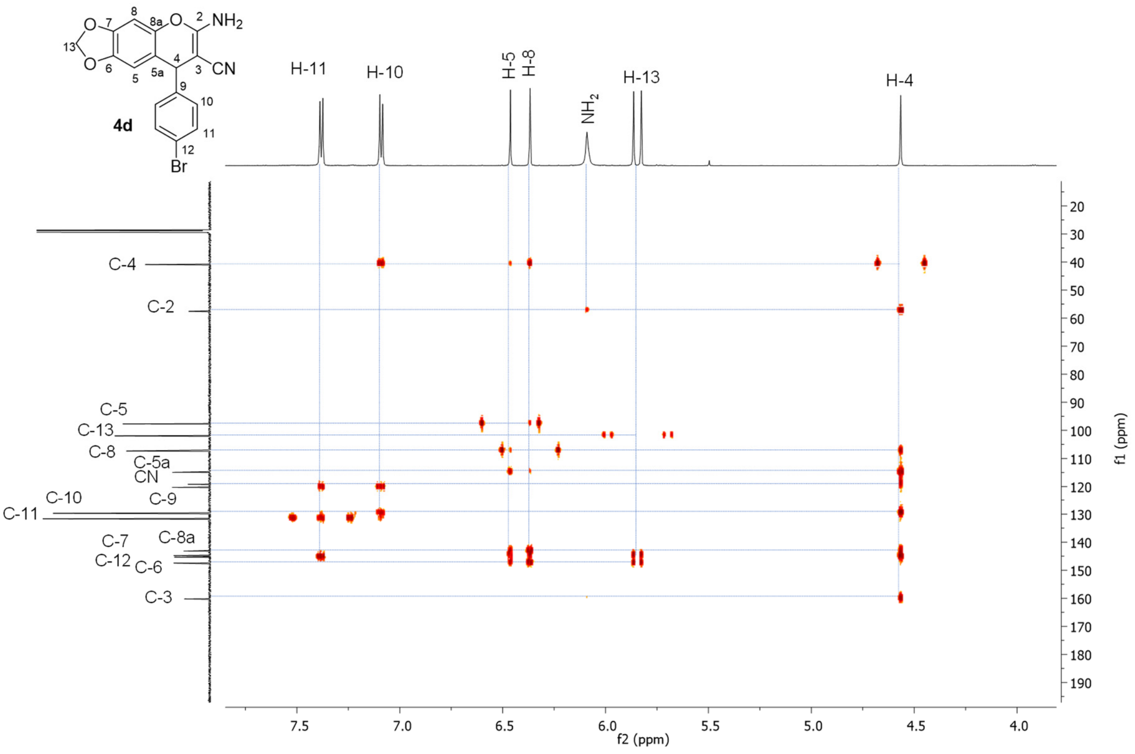

Figure S10. HMBC experiment of 2-Amino-4-(4-bromophenyl)-3-cyano-6,7-methylendioxy-4*H*-chromene **4d**.

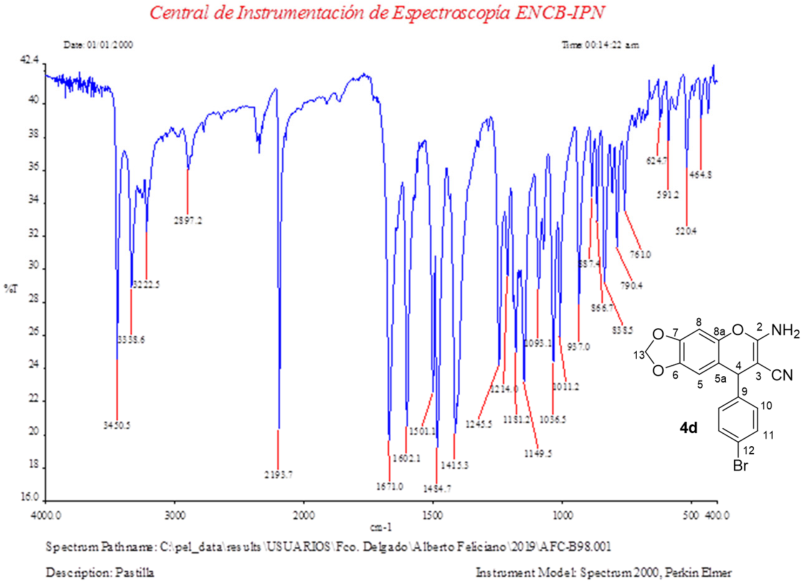

Figure S11. FT-IR of 2-Amino-4-(4-bromophenyl)-3-cyano-6,7-methylendioxy-4*H*-chromene **4d**.

File: FDR-AFC-B98 Date Run: 01-31-2019 (Time Run: 18:14:44)
Sample: FDR-AFC-B98
Instrument: JEOL GCmate
Inlet: Direct Probe Ionization mode: EI+

Scan: 507 R.T.: 6.74
Base: m/z 370; 4.3%FS TIC: 283744 #Ions: 139

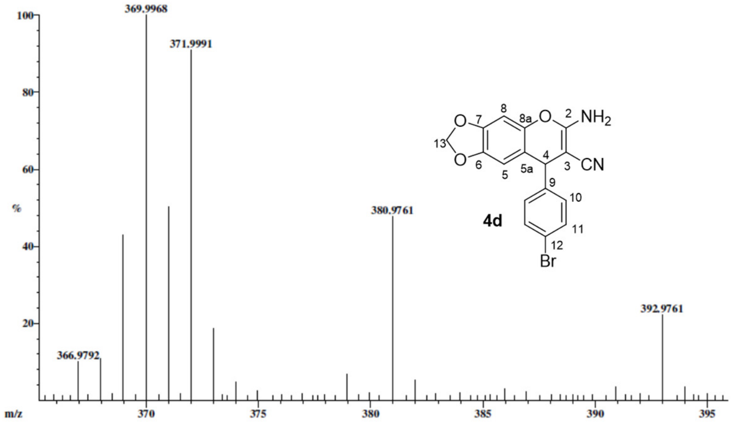

Selected Isotopes : $H_{0-11}C_{0-17}N_{0-2}O_{0-3}Br_{0-1}$ Error Limit : 5 ppm

Measured Mass	% Base	Formula	Calculated Mass	Error
369.9968	100.0%	$C_{17}H_{11}N_2O_3Br$	369.9953	4.0

Figure S12. HRMS (EI) $[M]^+$ of 2-Amino-4-(4-bromophenyl)-3-cyano-6,7-methylendioxy-4*H*-chromene **4d**.

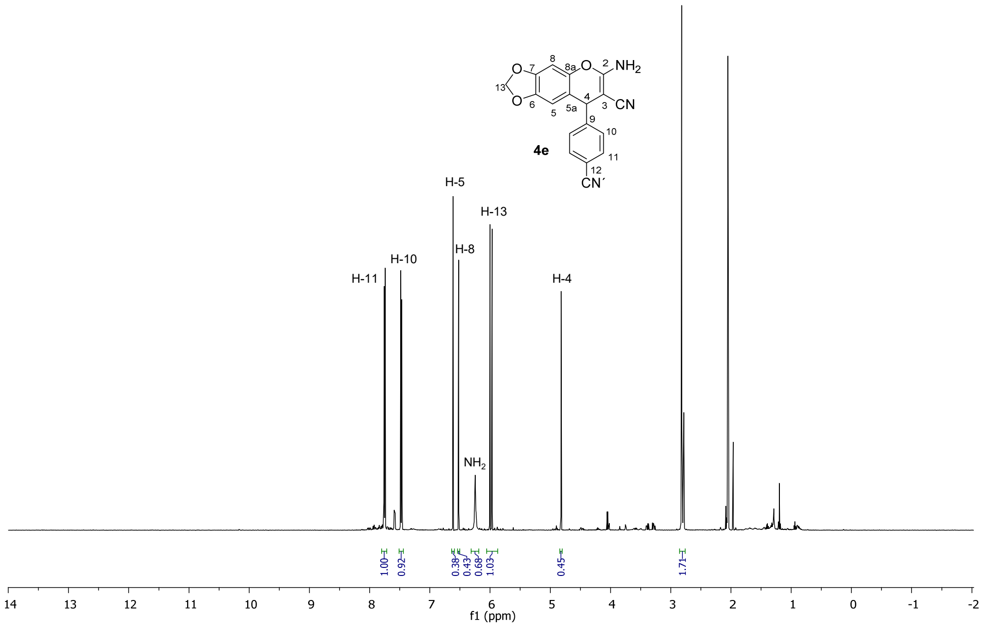

Figure S13. ^{1}H NMR (600 MHz, acetone d$_6$) of 2-Amino-3-cyano-4-(4-cyanophenyl)-6,7-methylendioxy-4*H*-chromene **4e**.

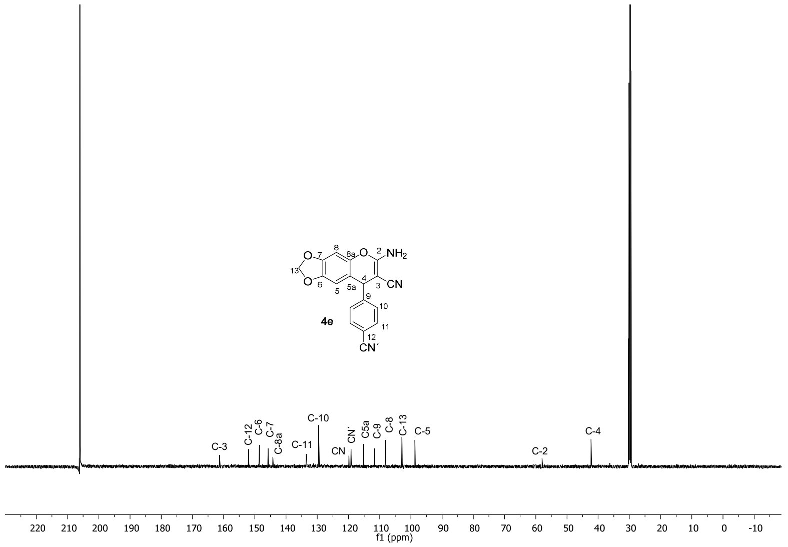

Figure S14. ^{13}C NMR (150 MHz, acetone d$_6$) of 2-Amino-3-cyano-4-(4-cyanophenyl)-6,7-methylendioxy-4*H*-chromene **4e**

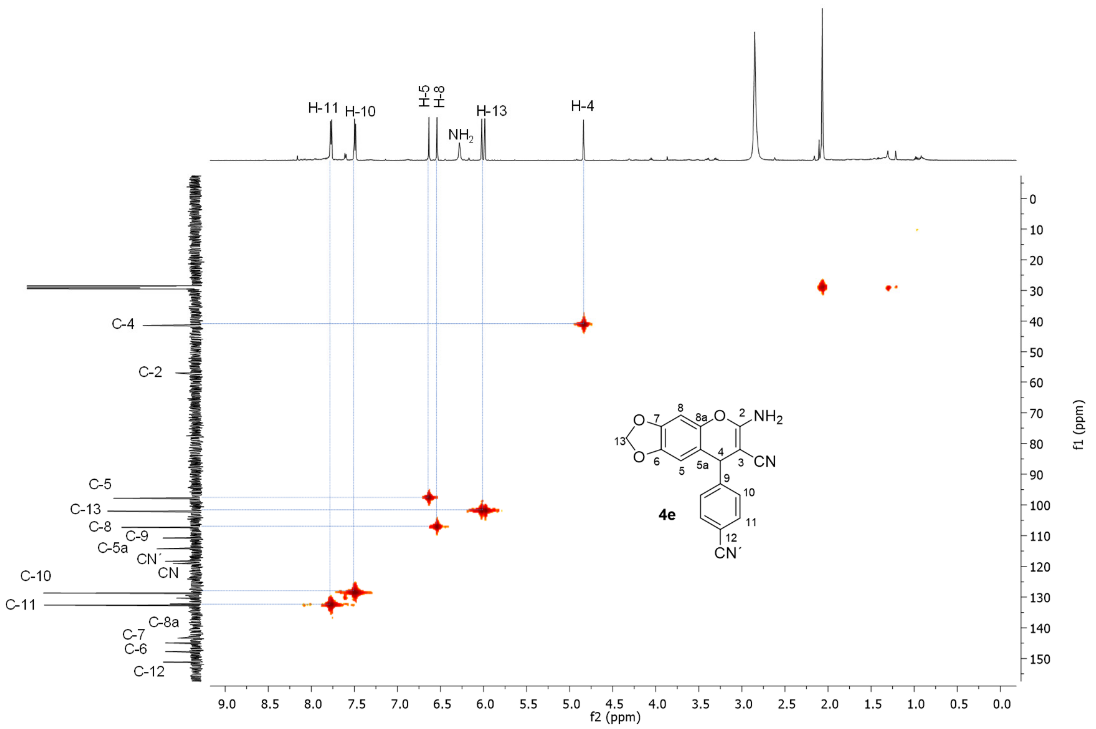

Figure S15. HMQC experiment of 2-Amino-3-cyano-4-(4-cyanophenyl)-6,7-methylendioxy-4*H*-chromene **4e**

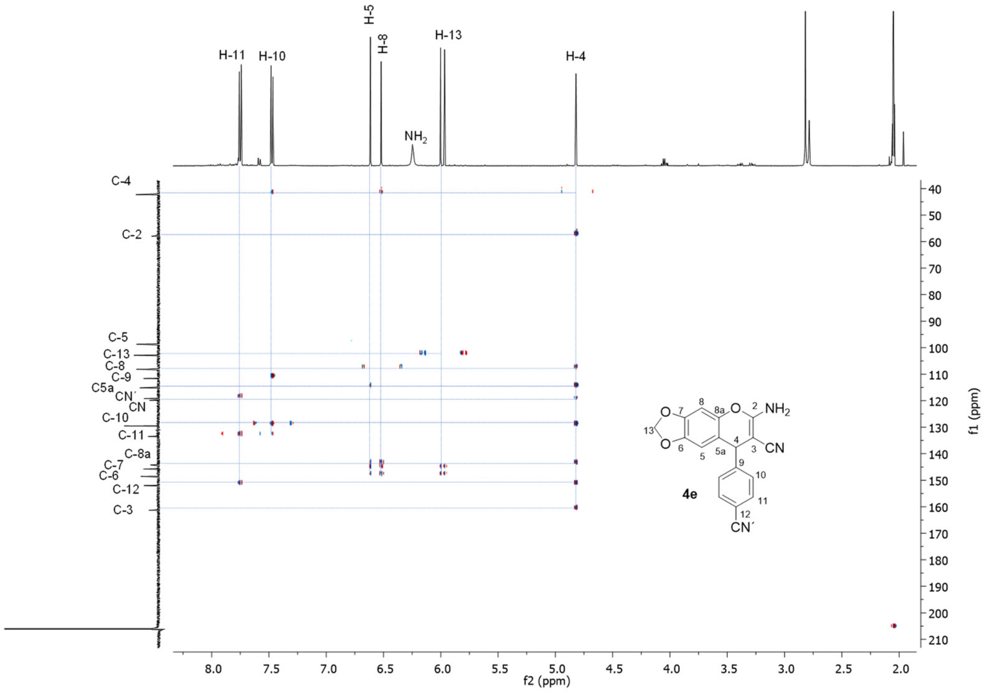

Figure S16. HMBC experiment of 2-Amino-3-cyano-4-(4-cyanophenyl)-6,7-methylendioxy-4*H*-chromene **4e**.

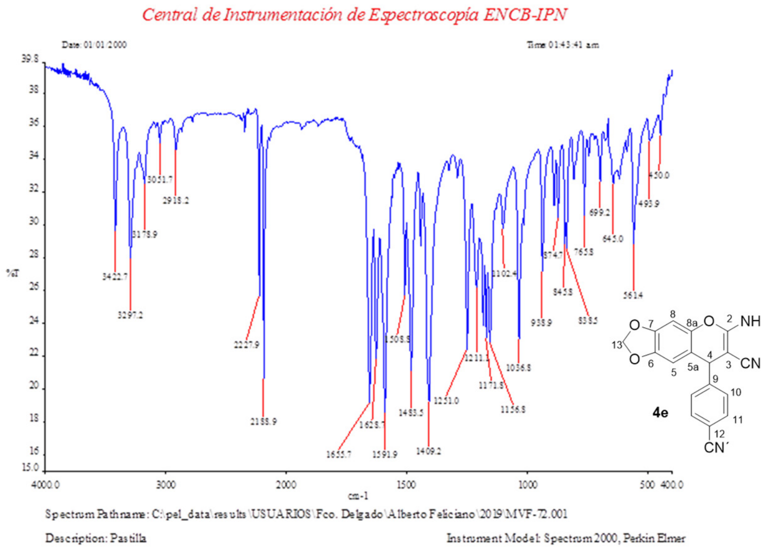

Figure S17. FT-IR of 2-Amino-3-cyano-4-(4-cyanophenyl)-6,7-methylendioxy-4*H*-chromene **4e**.

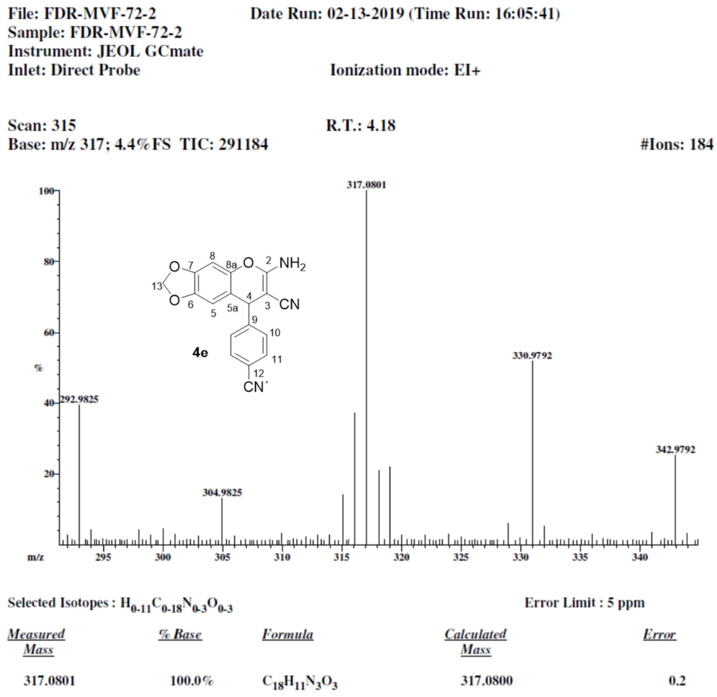

Figure S18. HRMS (EI) $[M]^+$ of 2-Amino-3-cyano-4-(4-cyanophenyl)-6,7-methylendioxy-4*H*-chromene **4e**.

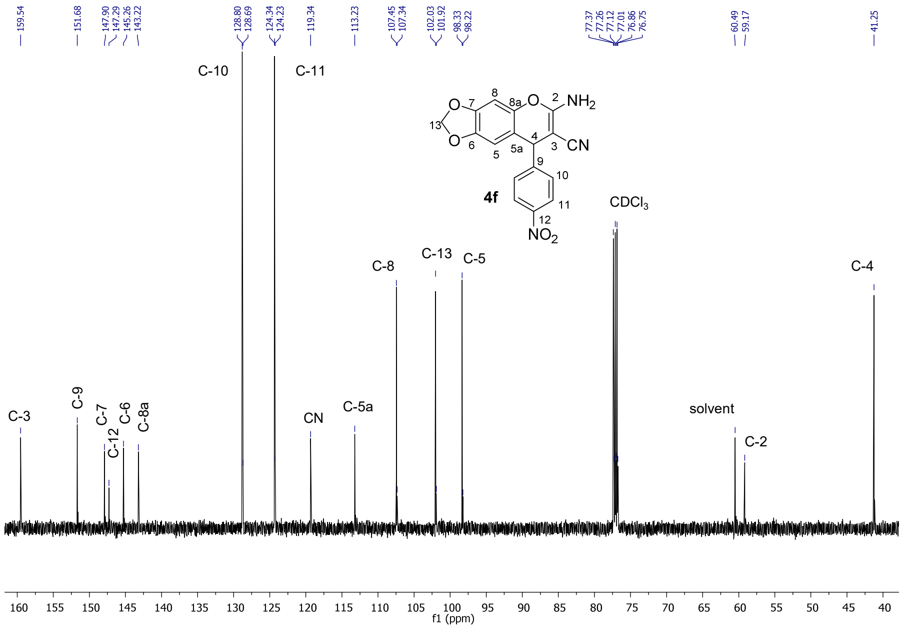

Figure S19. ^{13}C NMR (75 MHz, $CDCl_3$) of 2-Amino-3-cyano-6,7-methylendioxy-4-(4-nitrophenyl)-4*H*-chromene **4f**.

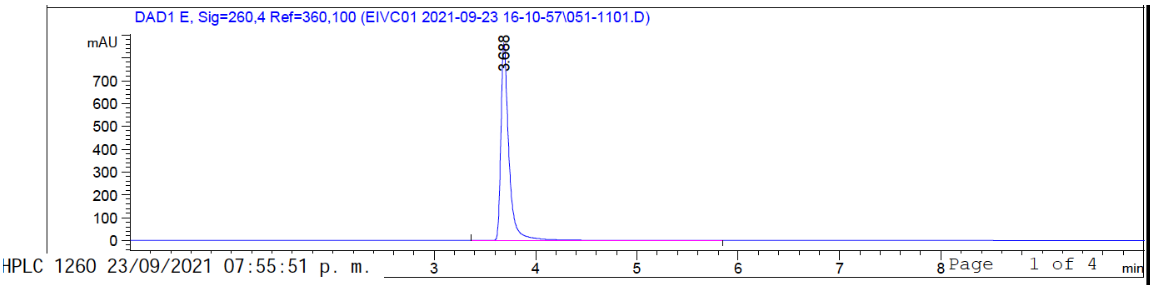

Signal 5: DAD1 E, Sig=260,4 Ref=360,100

Peak #	RetTime [min]	Type	Width [min]	Area [mAU*s]	Height [mAU]	Area %
1	3.688	BB	0.0820	4793.32666	859.96552	100.0000
Totals :				4793.32666	859.96552	

Figure S20. **HPLC** of 2-Amino-3-cyano-6,7-methylendioxy-4-(4-nitrophenyl)-4*H*-chromene **4f**. 0.1 mg/ml, MeCN, 0.4 ml/min, 260 nm, purity = 100%.

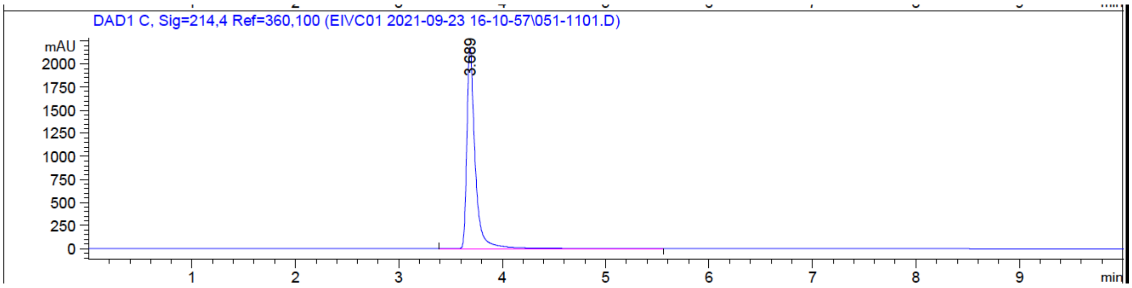

Signal 3: DAD1 C, Sig=214,4 Ref=360,100

Peak #	RetTime [min]	Type	Width [min]	Area [mAU*s]	Height [mAU]	Area %
1	3.689	BB	0.0826	1.22252e4	2174.58374	100.0000
Totals :				1.22252e4	2174.58374	

Figure S21. **HPLC** of 2-Amino-3-cyano-6,7-methylendioxy-4-(4-nitrophenyl)-4*H*-chromene **4f**. 0.1 mg/ml, MeCN, 0.4 ml/min, 214 nm, purity = 100%.

File: FDR-MVF-70 Date Run: 02-13-2019 (Time Run: 12:02:36)
Sample: FDR-MVF-70
Instrument: JEOL GCmate
Inlet: Direct Probe Ionization mode: EI+

Scan: 174 R.T.: 2.31
Base: m/z 331; 1.6%FS TIC: 183040 #Ions: 200

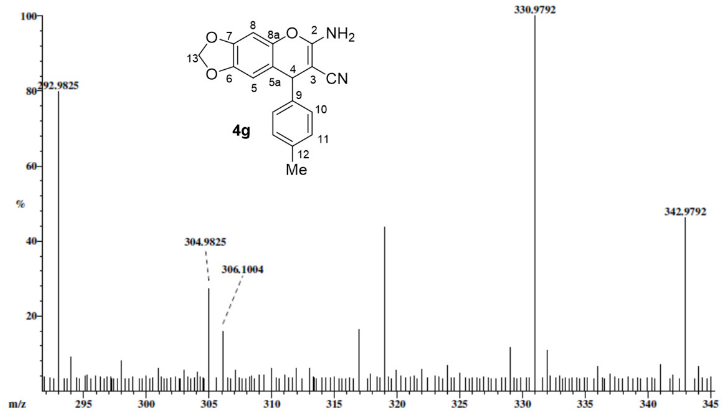

Selected Isotopes : $H_{0-14}C_{0-18}N_{0-2}O_{0-3}$ Error Limit : 5 ppm

Measured Mass	% Base	Formula	Calculated Mass	Error
306.1004	15.8%	$C_{18}H_{14}N_2O_3$	306.1005	-0.2

Figure S22. HRMS (EI) $[M]^+$ of 2-Amino-3-cyano-6,7-methylendioxy-4-(*p*-tolyl)-4*H*-chromene **4g**.

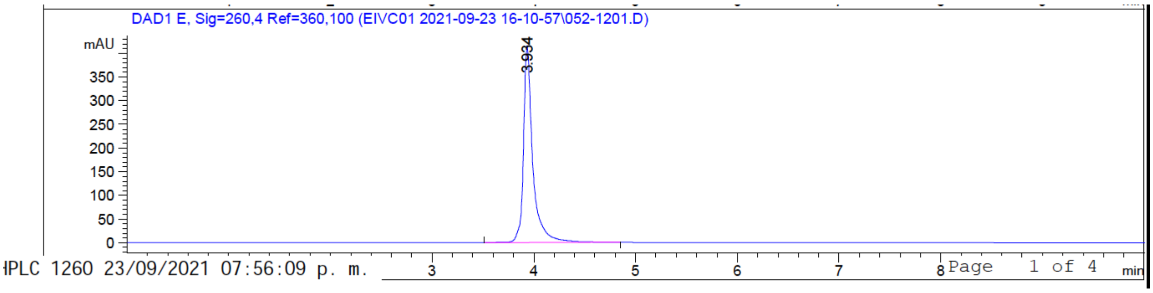

Signal 5: DAD1 E, Sig=260,4 Ref=360,100

Peak #	RetTime [min]	Type	Width [min]	Area [mAU*s]	Height [mAU]	Area %
1	3.934	BB	0.0921	2681.58374	416.48093	100.0000

Totals : 2681.58374 416.48093

Figure S23. **HPLC** of 2-Amino-3-cyano-6,7-methylendioxy-4-(*p*-tolyl)-4*H*-chromene **4g**. 0.1 mg/ml, MeCN, 0.4 ml/min, 260 nm, purity = 100%.

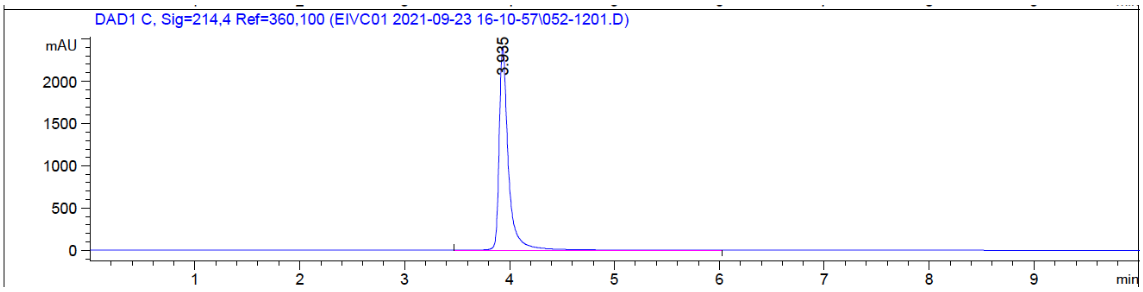

Signal 3: DAD1 C, Sig=214,4 Ref=360,100

Peak #	RetTime [min]	Type	Width [min]	Area [mAU*s]	Height [mAU]	Area %
1	3.935	BB	0.0900	1.48660e4	2408.74023	100.0000

Totals : 1.48660e4 2408.74023

Figure S24. **HPLC** of 2-Amino-3-cyano-6,7-methylendioxy-4-(*p*-tolyl)-4*H*-chromene **4g**. 0.1 mg/ml, MeCN, 0.4 ml/min, 214 nm, purity = 100%.

File: FDR-AFC-B99 Date Run: 02-13-2019 (Time Run: 13:01:08)
Sample: FDR-AFC-B99
Instrument: JEOL GCmate
Inlet: Direct Probe Ionization mode: EI+

Scan: 211 R.T.: 2.79
Base: m/z 331; 10.6%FS TIC: 682176 #Ions: 156

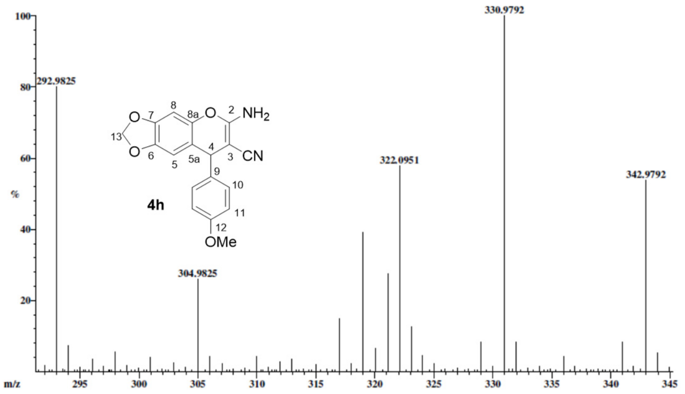

Selected Isotopes : $H_{0-14}C_{0-18}N_{0-2}O_{0-4}$ Error Limit : 5 ppm

Measured Mass	% Base	Formula	Calculated Mass	Error
322.0951	57.9%	$C_{18}H_{14}N_2O_4$	322.0954	-0.8

Figure S25. HRMS (EI) $[M]^+$ of 2-Amino-3-cyano-6,7-methylendioxy-4-(4-methoxyphenyl)-4*H*-chromene **4h**.

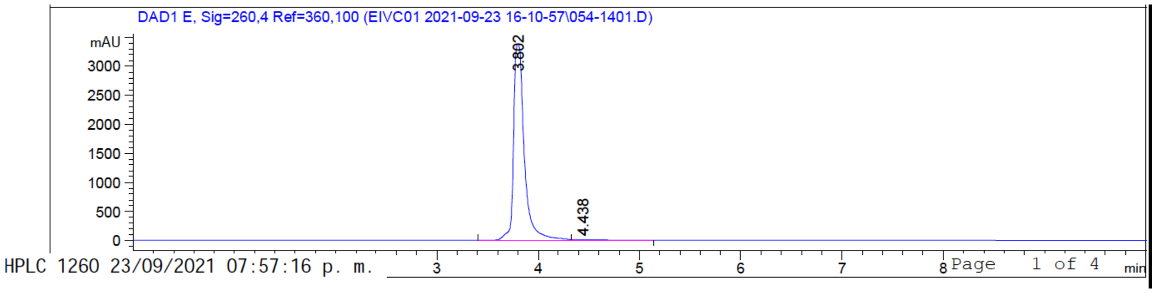

Signal 5: DAD1 E, Sig=260,4 Ref=360,100

Peak #	RetTime [min]	Type	Width [min]	Area [mAU*s]	Height [mAU]	Area %
1	3.802	BV R	0.1103	2.50654e4	3388.76807	99.1286
2	4.438	VB E	0.2834	220.34758	10.64773	0.8714
Totals :				2.52857e4	3399.41580	

Figure S26. **HPLC** of 2-Amino-3-cyano-6,7-methylendioxy-4-(4-methoxyphenyl)-4*H*-chromene **4h**. 0.1 mg/ml, MeCN, 0.4 ml/min, 260 nm, purity = 99.12%.

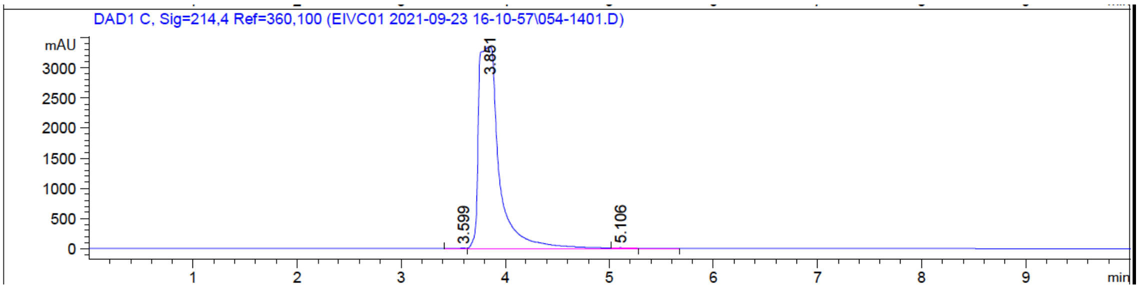

Signal 3: DAD1 C, Sig=214,4 Ref=360,100

Peak #	RetTime [min]	Type	Width [min]	Area [mAU*s]	Height [mAU]	Area %
1	3.599	BV E	0.0550	33.72494	9.14167	0.0729
2	3.851	VV R	0.1836	4.61891e4	3355.85645	99.8576
3	5.106	VB E	0.0915	32.13812	5.24058	0.0695
Totals :				4.62550e4	3370.23869	

Figure S27. **HPLC** of 2-Amino-3-cyano-6,7-methylendioxy-4-(4-methoxyphenyl)-4*H*-chromene **4h**. 0.1 mg/ml, MeCN, 0.4 ml/min, 214 nm, purity = 99.85%.

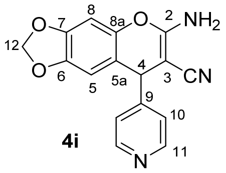

4i

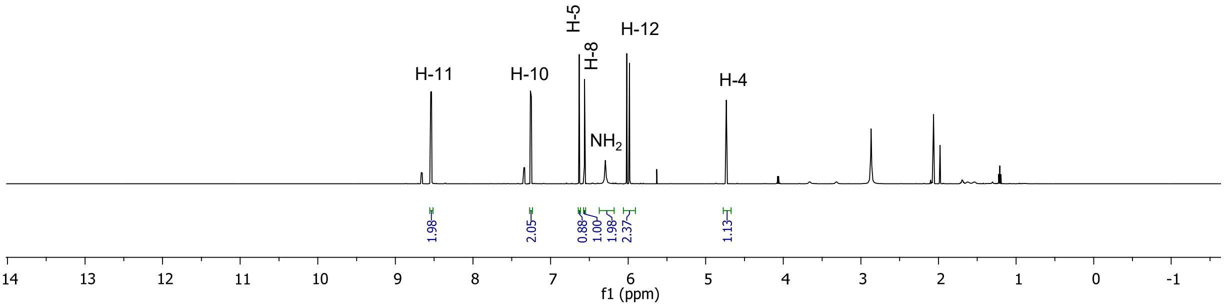

Figure S28. ^{1}H NMR (600 MHz, acetone d6) of 2-Amino-3-cyano-6,7-methylendioxy-4-(pyridin-4-yl)-4*H*-chromene **4i**.

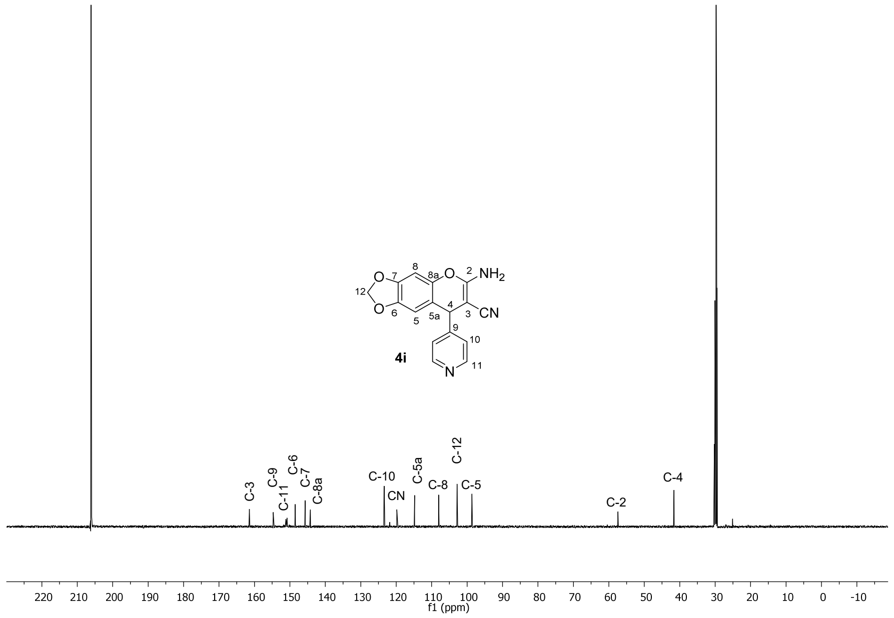

Figure S29. ^{13}C NMR (150 MHz, acetone d$_6$) of 2-Amino-3-cyano-6,7-methylendioxy-4-(pyridin-4-yl)-4*H*-chromene **4i**.

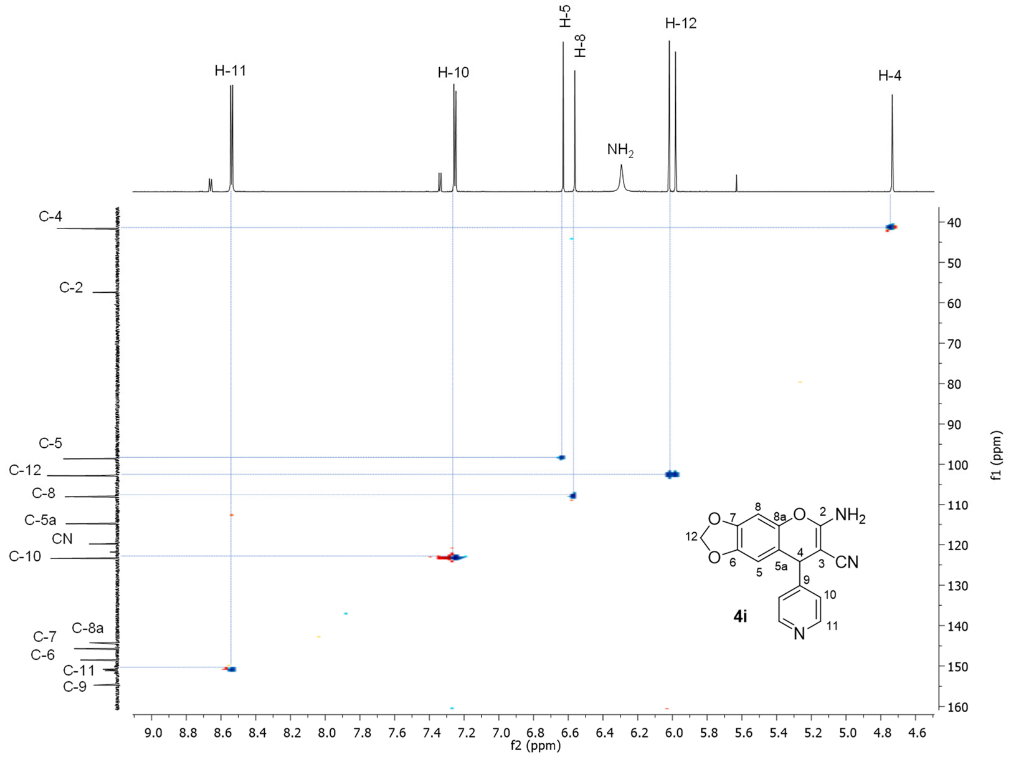

Figure S30. HMQC experiment of 2-Amino-3-cyano-6,7-methylendioxy-4-(pyridin-4-yl)-4*H*-chromene **4i**.

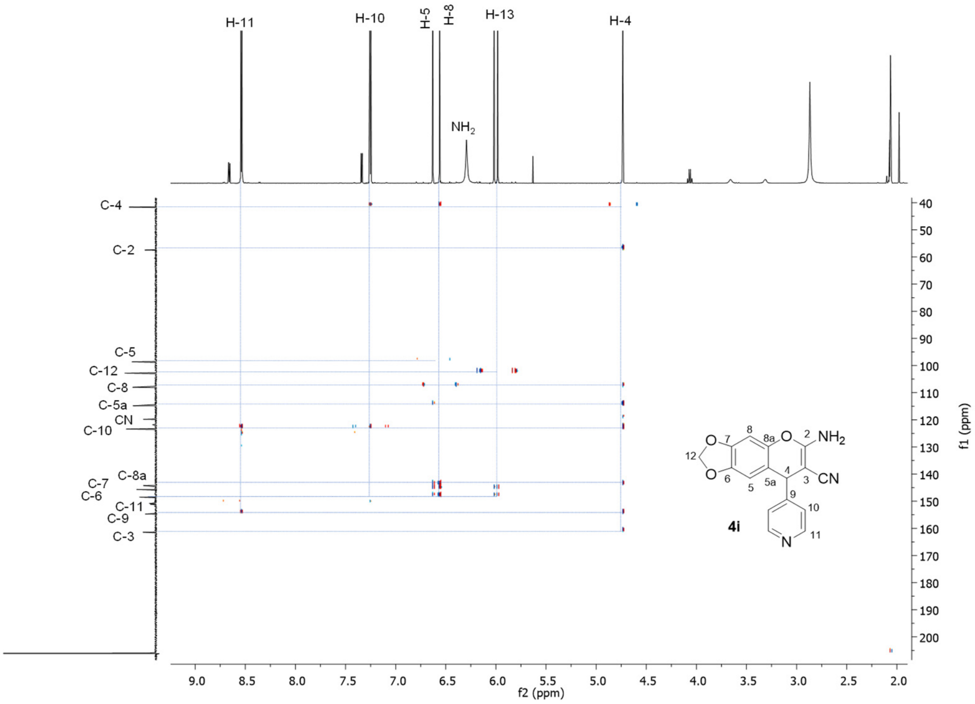

Figure S31. HMBC experiment of 2-Amino-3-cyano-6,7-methylendioxy-4-(pyridin-4-yl)-4*H*-chromene **4i**.

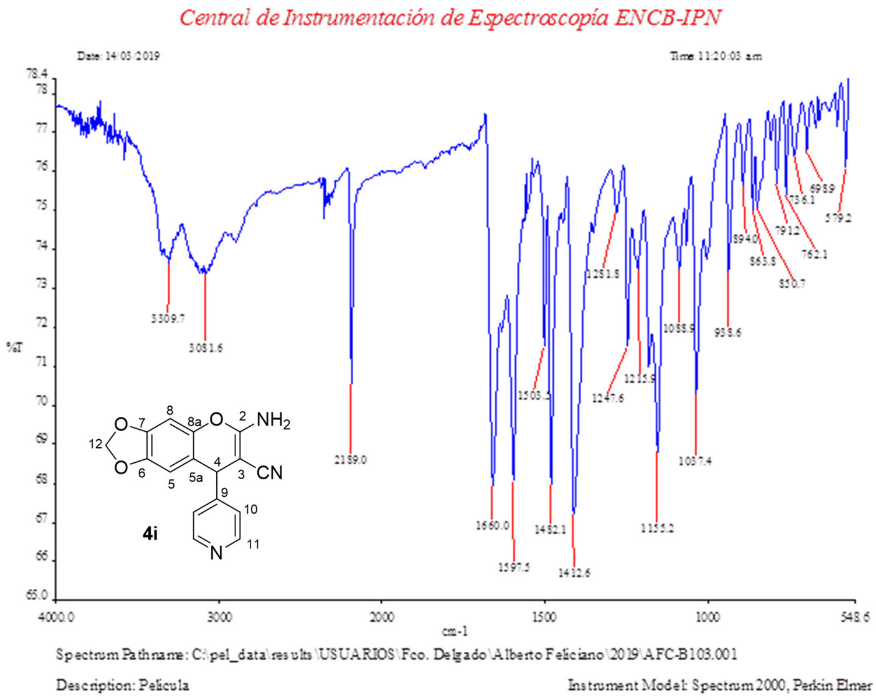

Figure S32. FT-IR of 2-Amino-3-cyano-6,7-methylendioxy-4-(pyridin-4-yl)-4*H*-chromene **4i**.

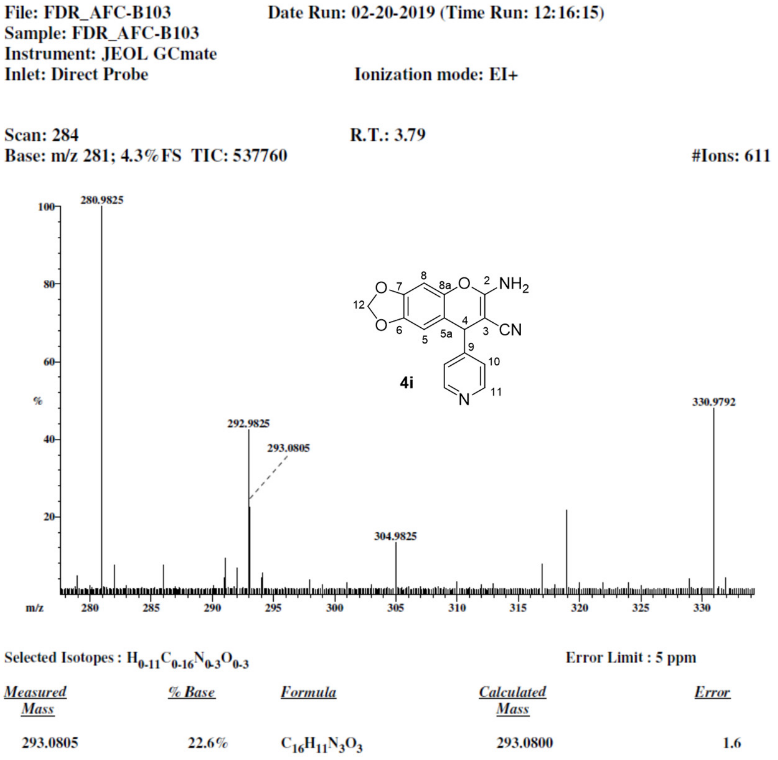

Figure S33. HRMS (EI) [M]$^{+}$ of 2-Amino-3-cyano-6,7-methylendioxy-4-(pyridin-4-yl)-4*H*-chromene **4i**.

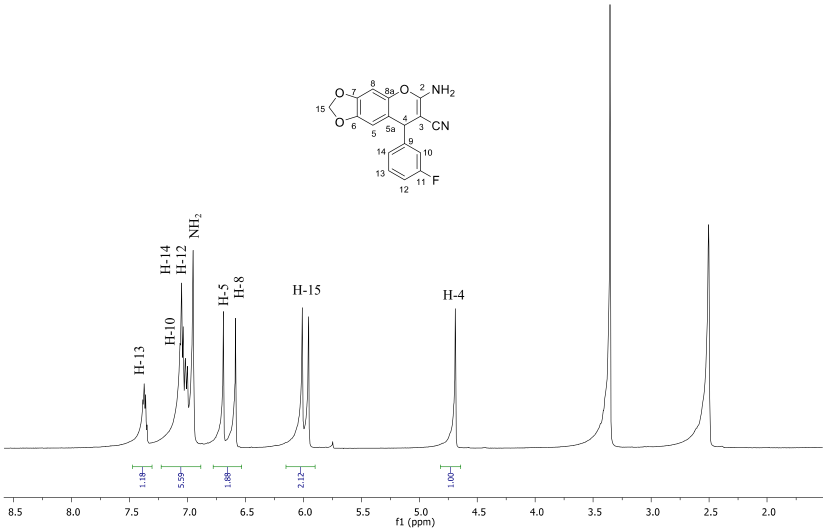

Figure S34. ^{1}H NMR (600 MHz, DMSO d_6) of 2-amino-3-cyano-4-(3-fluorophenyl)-6,7-methylendioxy-4*H*- chromene **4j**.

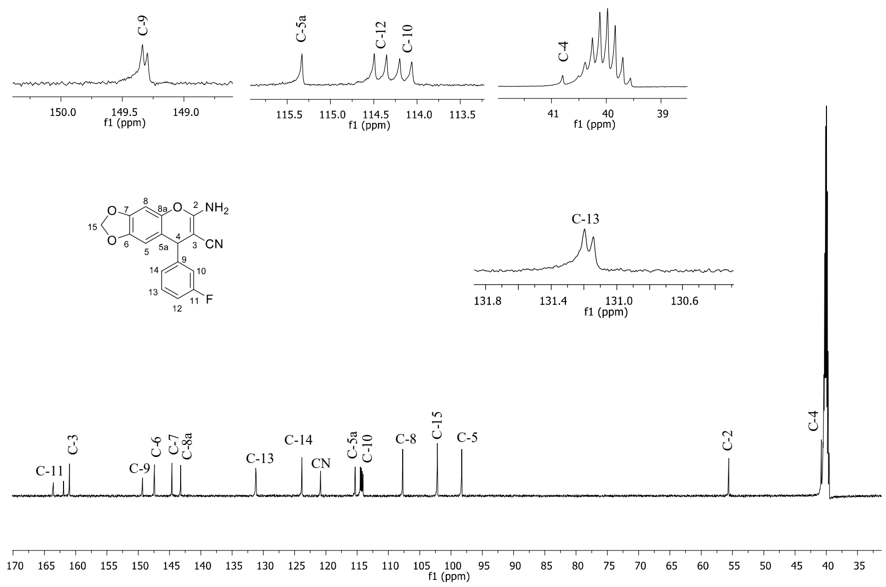

Figure S35. ^{13}C NMR (150 MHz, DMSO d_6) of 2-amino-3-cyano-4-(3-fluorophenyl)-6,7-methylendioxy-4*H*- chromene **4j**.

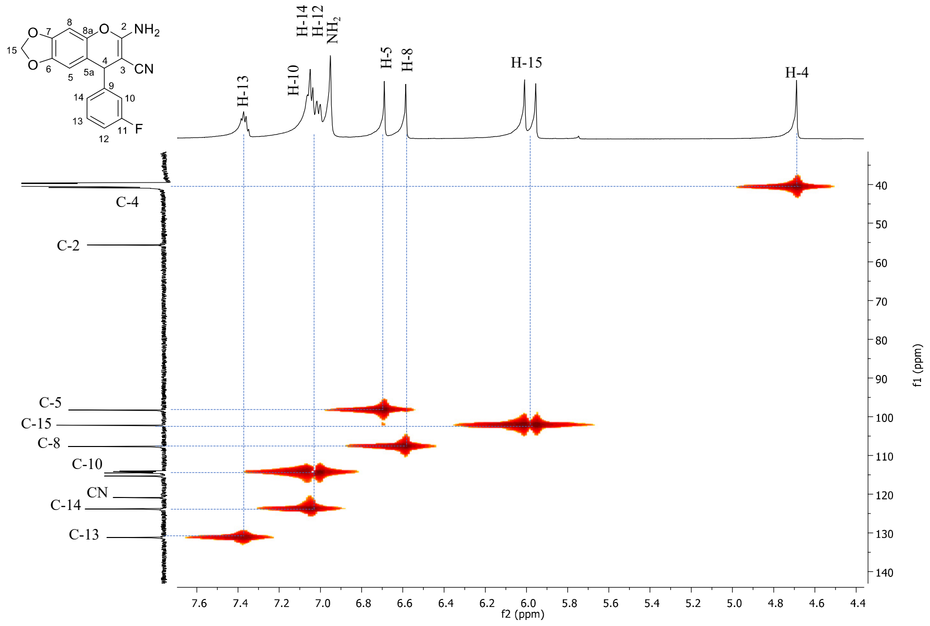

Figure S36. HMQC experiment of 2-amino-3-cyano-4-(3-fluorophenyl)-6,7-methylendioxy-4*H*- chromene **4j**.

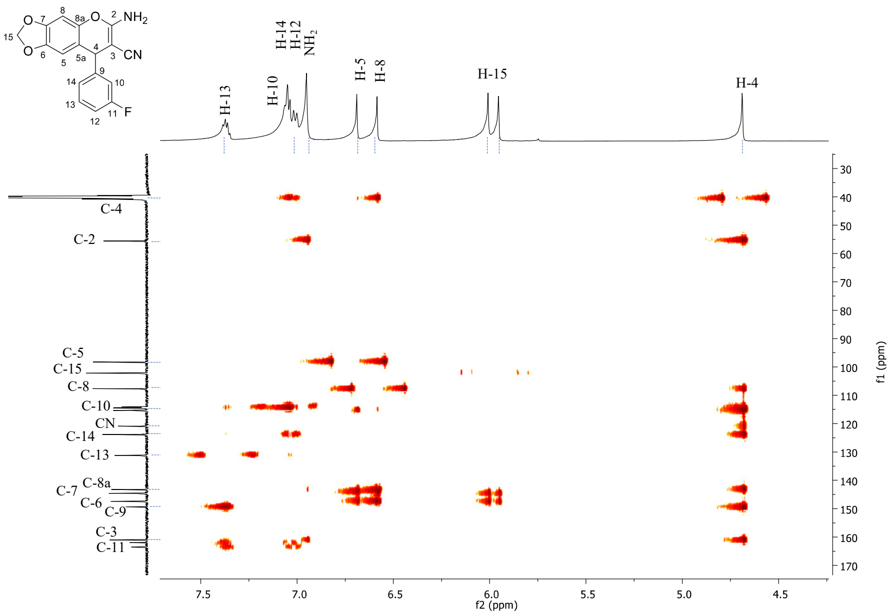

Figure S37. HMBC experiment of 2-amino-3-cyano-4-(3-fluorophenyl)-6,7-methylendioxy-4*H*- chromene **4j**.

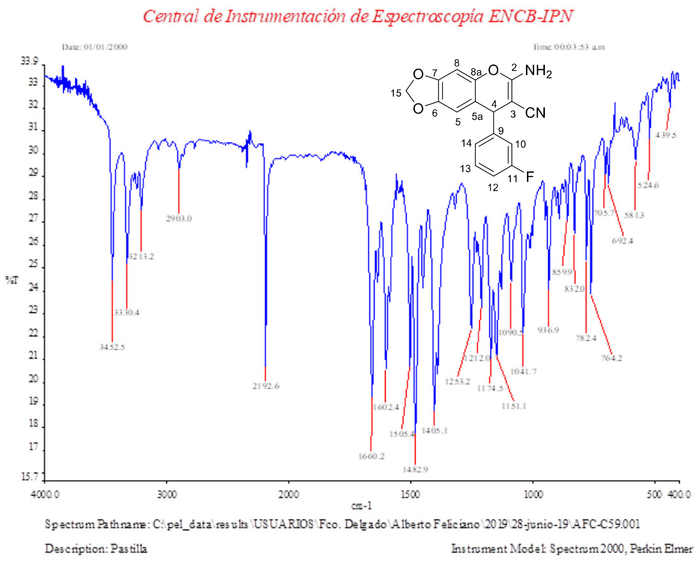

Figure S38. FT-IR of 2-amino-3-cyano-4-(3-fluorophenyl)-6,7-methylendioxy-4*H*-chromene **4j**.

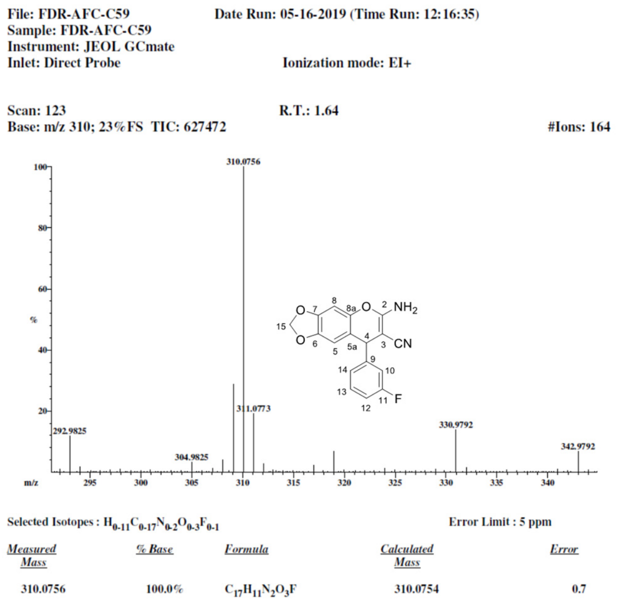

Figure S39. HRMS (EI) $[M]^+$ of 2-amino-3-cyano-4-(3-fluorophenyl)-6,7-methylendioxy-4*H*- chromene **4j**.

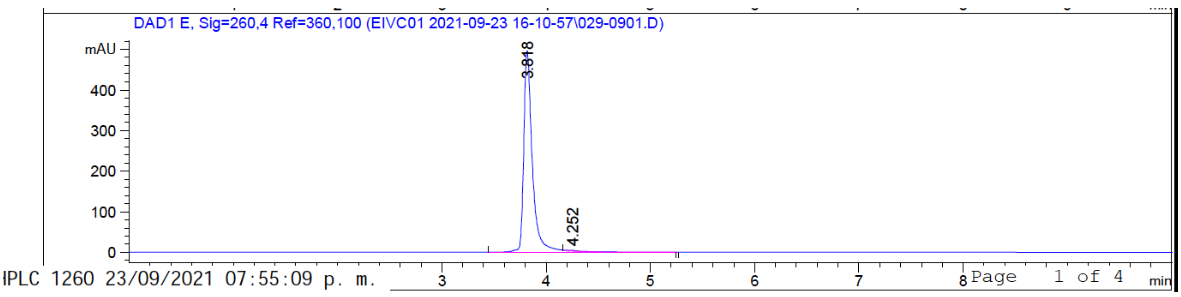

Signal 5: DAD1 E, Sig=260,4 Ref=360,100

Peak #	RetTime [min]	Type	Width [min]	Area [mAU*s]	Height [mAU]	Area %
1	3.818	BV R	0.0847	2885.13257	496.98206	99.1569
2	4.252	VB E	0.2195	24.53098	1.40626	0.8431

Totals :				2909.66355	498.38831	

Figure S-0. **HPLC** of 2-amino-3-cyano-4-(3-fluorophenyl)-6,7-methylendioxy-4*H*-chromene **4j**. 0.1 mg/ml, MeCN, 0.4 ml/min, 260 nm, purity = 99.15%.

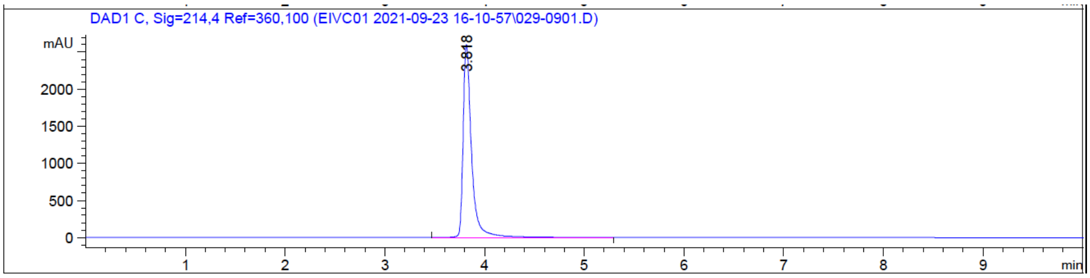

Signal 3: DAD1 C, Sig=214,4 Ref=360,100

Peak #	RetTime [min]	Type	Width [min]	Area [mAU*s]	Height [mAU]	Area %
1	3.818	BB	0.0877	1.53237e4	2601.53564	100.0000
Totals :				1.53237e4	2601.53564	

Figure S41. **HPLC** of 2-amino-3-cyano-4-(3-fluorophenyl)-6,7-methylendioxy-4*H*-chromene **4j**. 0.1 mg/ml, MeCN, 0.4 ml/min, 214 nm, purity = 100%.

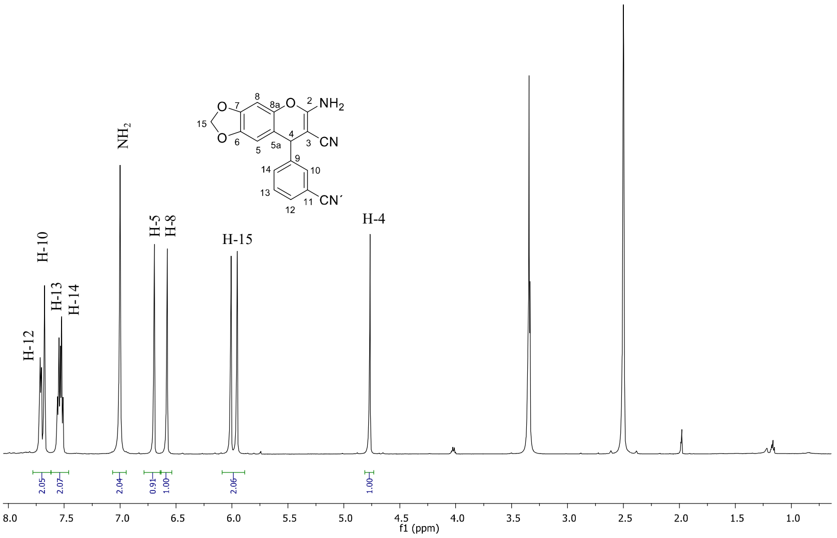

Figure S42. ^{1}H NMR (600 MHz, DMSO d$_6$) of 2-amino-3-cyano-4-(3-cyanophenyl)-6,7-methylendioxy-4*H*- chromene **4k**.

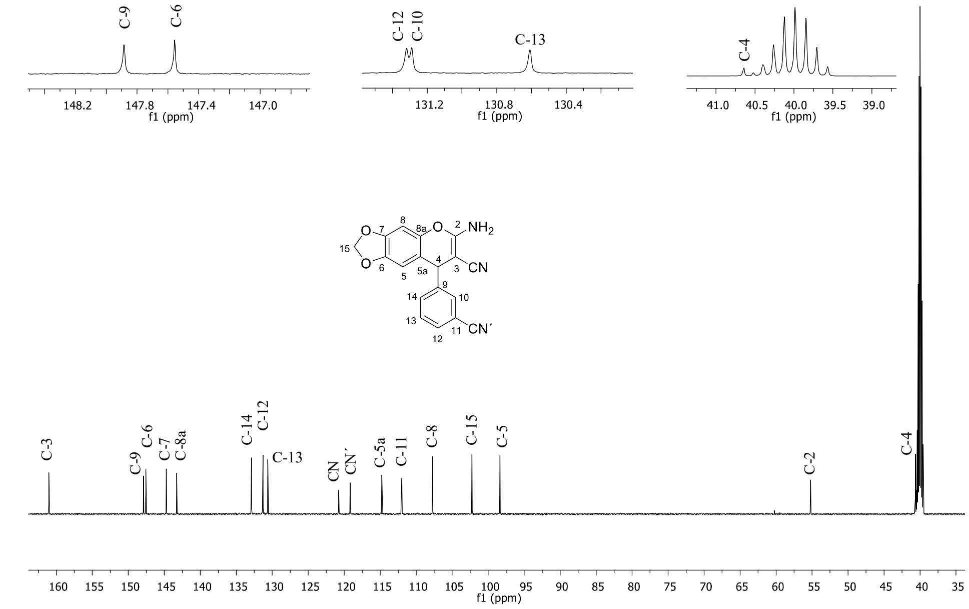

Figure S43. ^{13}C NMR (150 MHz, DMSO d_6) of 2-amino-3-cyano-4-(3-cyanophenyl)-6,7-methylendioxy-4*H*- chromene **4k**.

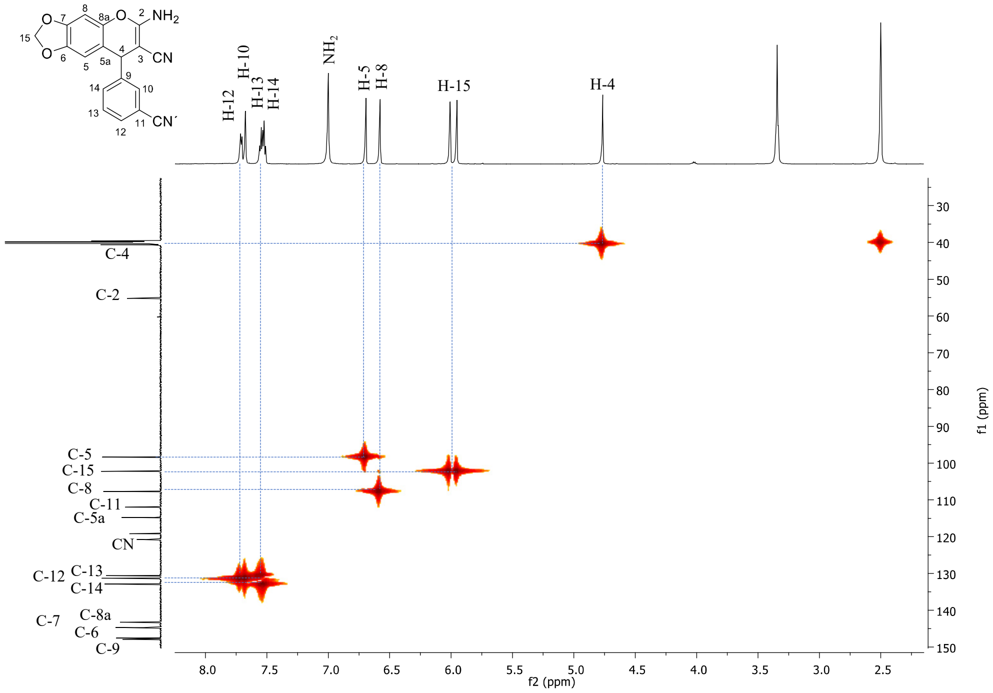

Figure S44. HMQC experiment of 2-amino-3-cyano-4-(3-cyanophenyl)-6,7-methylendioxy-4*H*- chromene **4k**.

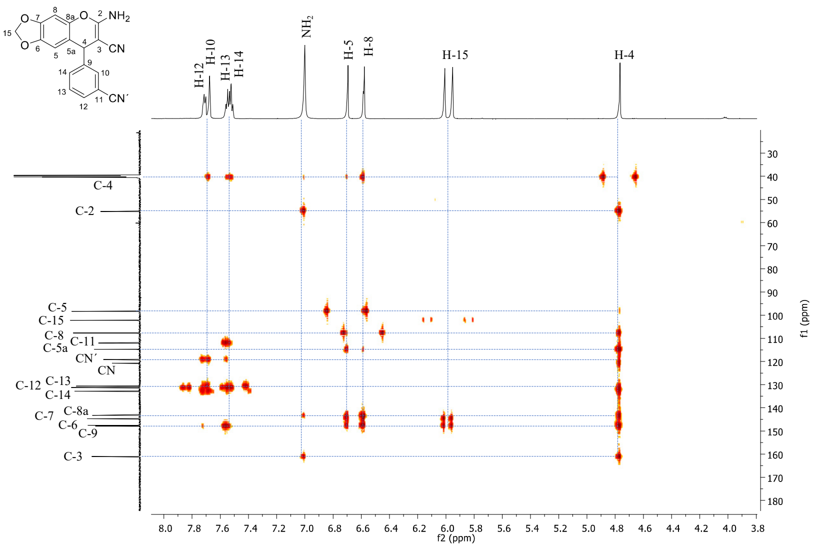

Figure S45. HMBC experiment of 2-amino-3-cyano-4-(3-cyanophenyl)-6,7-methylendioxy-4*H*- chromene **4k**.

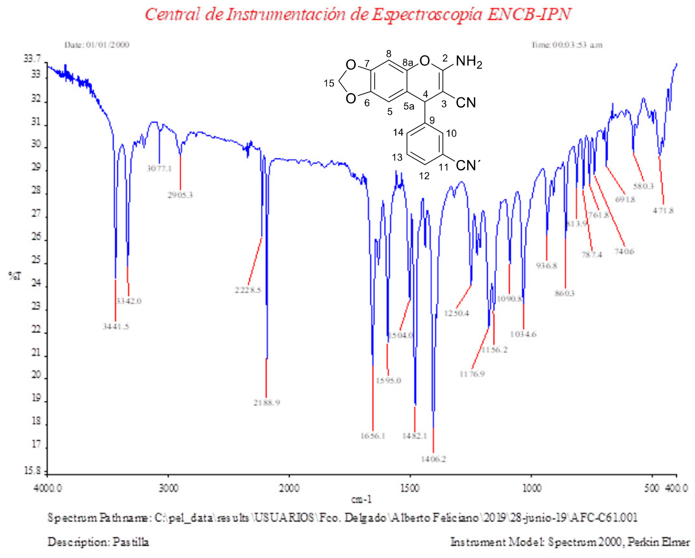

Figure S46. FT-IR of 2-amino-3-cyano-4-(3-cyanophenyl)-6,7-methylendioxy-4*H*-chromene **4k**.

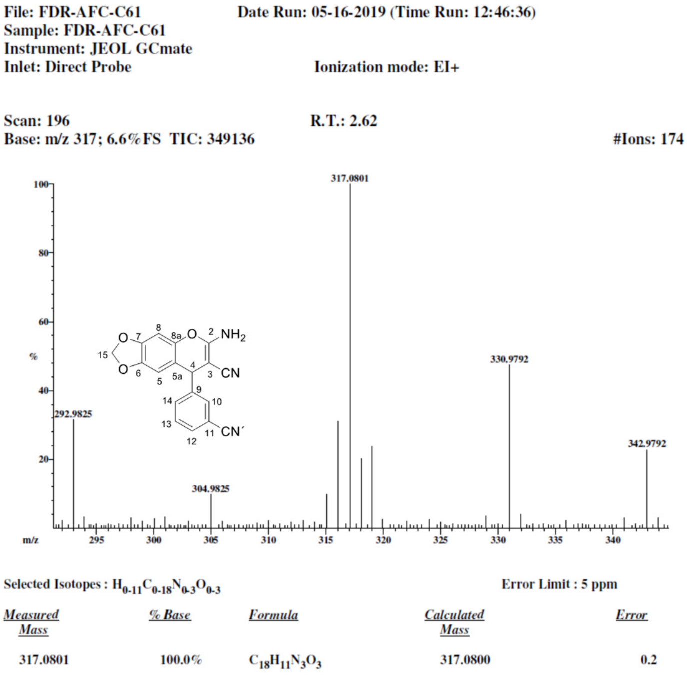

Figure S47. HRMS (EI) $[M]^+$ of 2-amino-3-cyano-4-(3-cyanophenyl)-6,7-methylendioxy-4*H*- chromene **4k**.

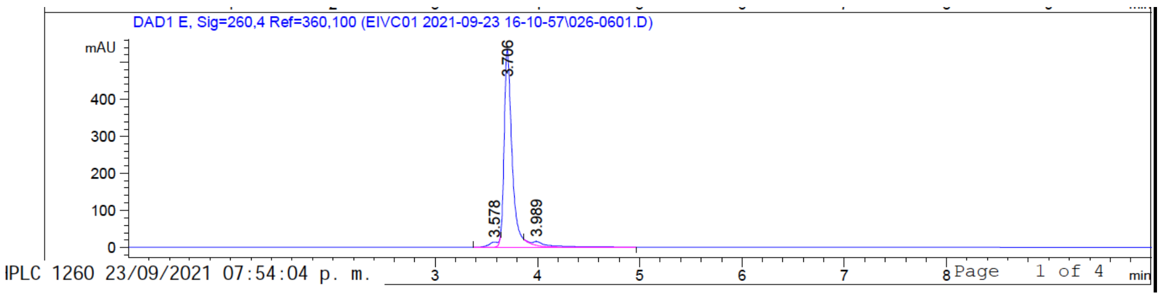

Signal 5: DAD1 E, Sig=260,4 Ref=360,100

Peak #	RetTime [min]	Type	Width [min]	Area [mAU*s]	Height [mAU]	Area %
1	3.578	BV E	0.0853	84.95459	14.29279	2.6819
2	3.706	VV R	0.0801	2943.35767	535.48724	92.9190
3	3.989	VB E	0.1709	139.34714	10.49443	4.3991
Totals :				3167.65939	560.27446	

Figure S48. **HPLC** of 2-amino-3-cyano-4-(3-cyanophenyl)-6,7-methylendioxy-4*H*-chromene **4k**. 0.1 mg/ml, MeCN, 0.4 ml/min, 260 nm, purity = 92.92%.

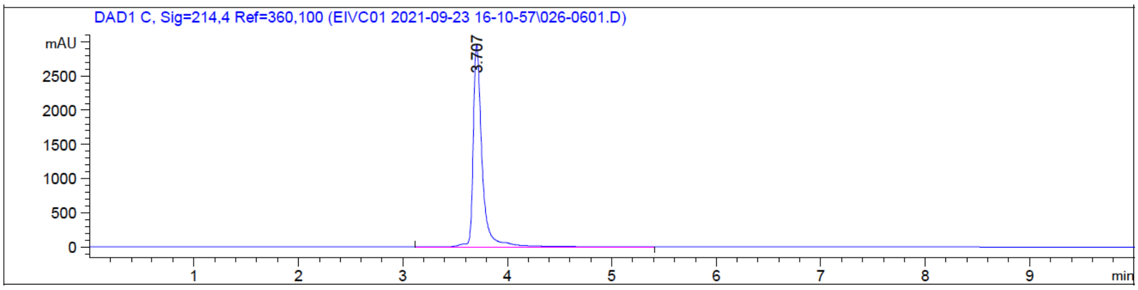

Signal 3: DAD1 C, Sig=214,4 Ref=360,100

Peak #	RetTime [min]	Type	Width [min]	Area [mAU*s]	Height [mAU]	Area %
1	3.707	BV R	0.0889	1.77300e4	2959.16895	100.0000

Totals : 1.77300e4 2959.16895

Figure S49. **HPLC** of 2-amino-3-cyano-4-(3-cyanophenyl)-6,7-methylendioxy-4*H*-chromene **4k**. 0.1 mg/ml, MeCN, 0.4 ml/min, 214 nm, purity = 100 %.

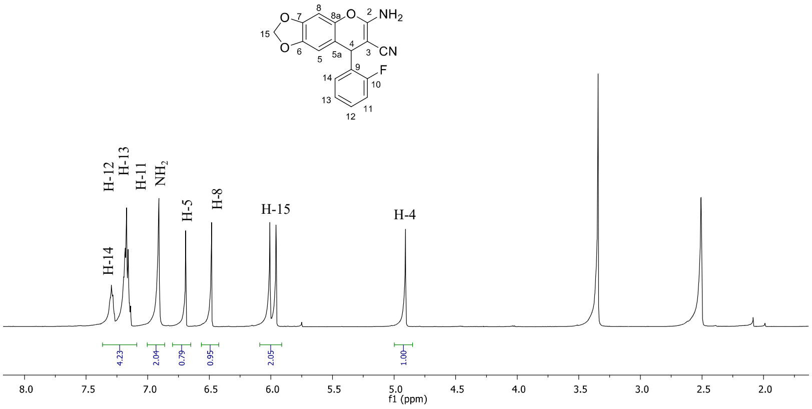

Figure S50. ^{1}H NMR (600 MHz, DMSO d_6) of 2-amino-3-cyano-4-(2-fluorophenyl)-6,7-methylendioxy-4*H*-chromene **4l**.

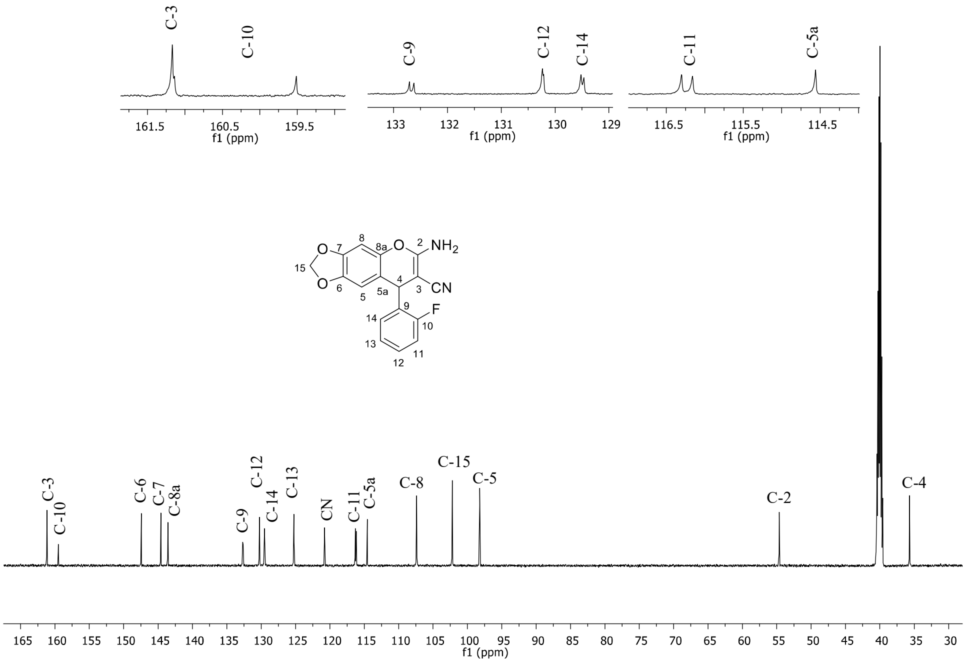

Figure S51. ^{13}C NMR (150 MHz, DMSO d_6) of 2-amino-3-cyano-4-(2-fluorophenyl)-6,7-methylendioxy-4*H*-chromene **4l**.

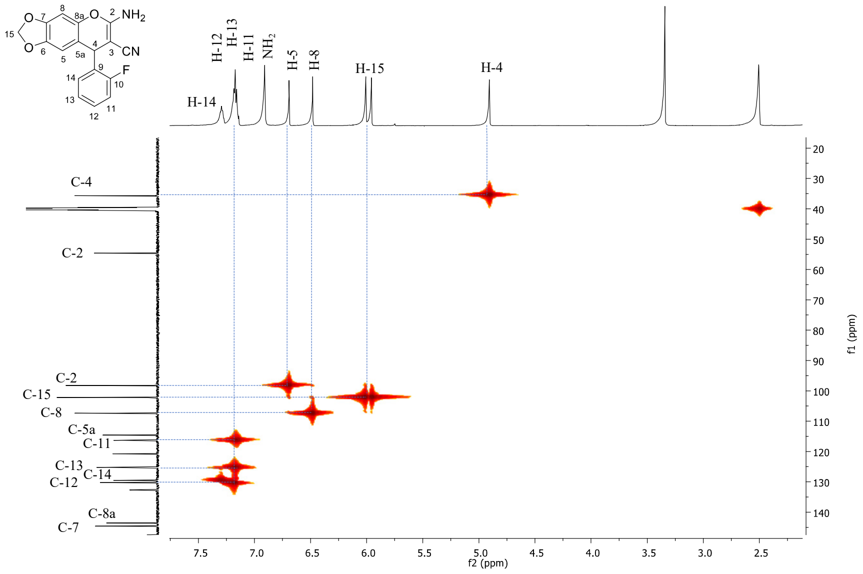

Figure S52. HMQC experiment of 2-amino-3-cyano-4-(2-fluorophenyl)-6,7-methylendioxy-4*H*-chromene **4l**.

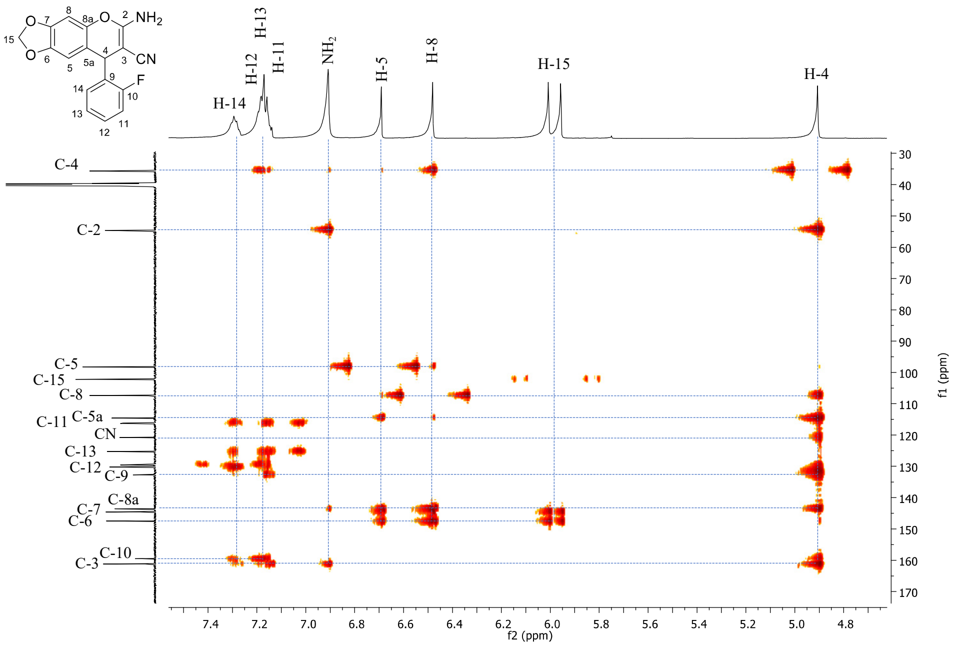

Figure S53. HMBC experiment of 2-amino-3-cyano-4-(2-fluorophenyl)-6,7-methylendioxy-4*H*-chromene **4l**.

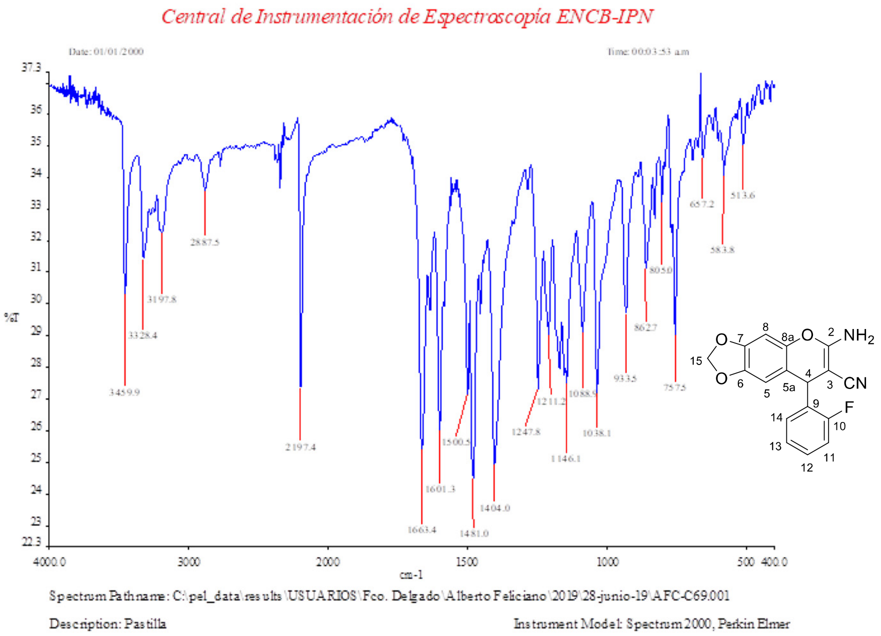

Figure S54. FT-IR of 2-amino-3-cyano-4-(2-fluorophenyl)-6,7-methylendioxy-4*H*-chromene **4l**.

Scan: 137 R.T.: 1.83
Base: m/z 310; 10%FS TIC: 411104 #Ions: 168

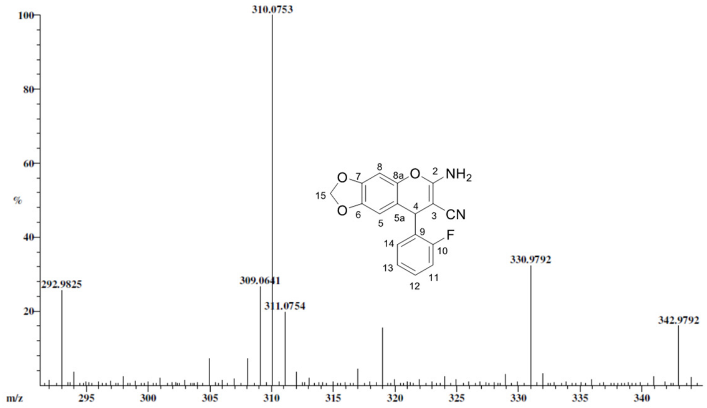

Selected Isotopes : $H_{0-11}C_{0-17}N_{0-2}O_{0-3}F_{0-1}$ Error Limit : 5 ppm

Measured Mass	% Base	Formula	Calculated Mass	Error
310.0753	100.0%	$C_{17}H_{11}N_2O_3F$	310.0754	-0.2

Figure S55. HRMS (EI) $[M]^+$ of 2-amino-3-cyano-4-(2-fluorophenyl)-6,7-methylendioxy-4*H*-chromene **4l**.

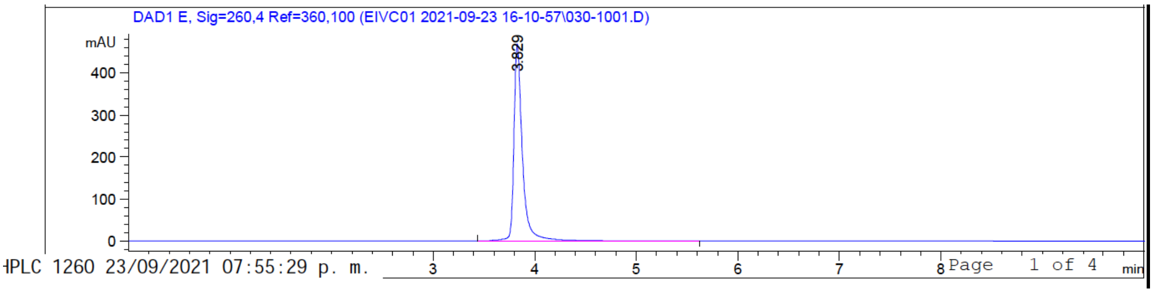

Signal 5: DAD1 E, Sig=260,4 Ref=360,100

Peak #	RetTime [min]	Type	Width [min]	Area [mAU*s]	Height [mAU]	Area %
1	3.829	BB	0.0858	2764.80737	469.16028	100.0000
Totals :				2764.80737	469.16028	

Figure S56. **HPLC** of 2-amino-3-cyano-4-(2-fluorophenyl)-6,7-methylendioxy-4*H*-chromene **4l**. 0.1 mg/ml, MeCN, 0.4 ml/min, 260 nm, purity = 100 %.

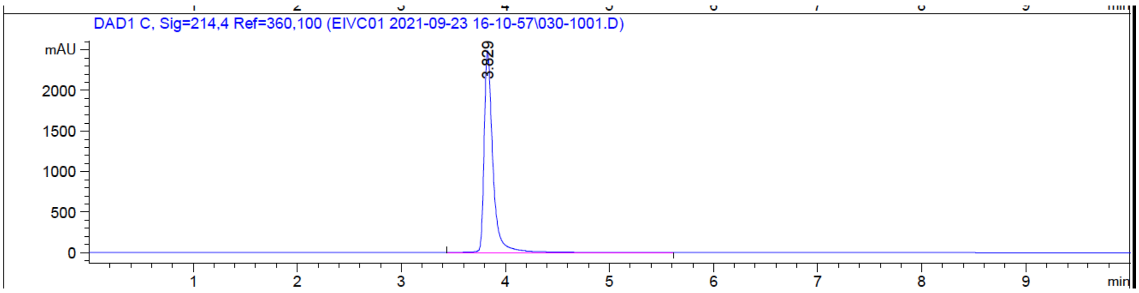

Signal 3: DAD1 C, Sig=214,4 Ref=360,100

Peak #	RetTime [min]	Type	Width [min]	Area [mAU*s]	Height [mAU]	Area %
1	3.829	BB	0.0877	1.46678e4	2491.67651	100.0000
Totals :				1.46678e4	2491.67651	

Figure S57. **HPLC** of 2-amino-3-cyano-4-(2-fluorophenyl)-6,7-methylendioxy-4*H*-chromene **4l**. 0.1 mg/ml, MeCN, 0.4 ml/min, 214 nm, purity = 100 %.

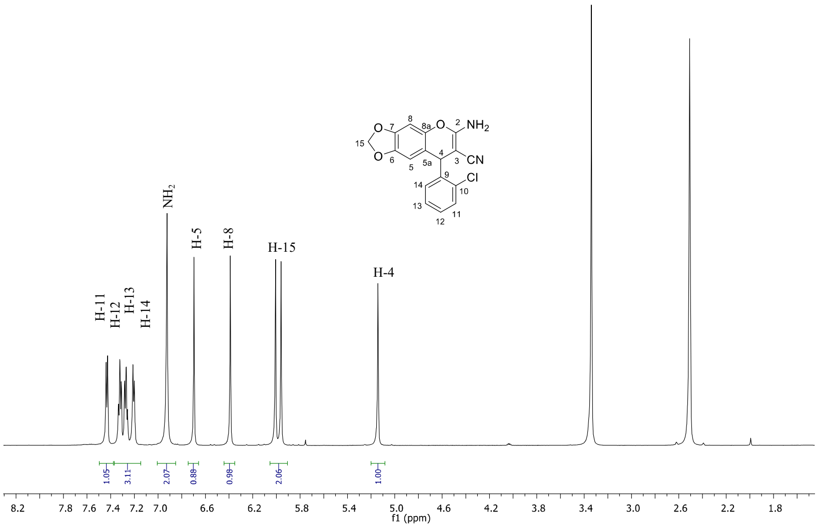

Figure S58. ^{1}H NMR (600 MHz, DMSO d$_6$) of 2-amino-3-cyano-4-(2-chlorophenyl)-6,7-methylendioxy-4*H*- chromene **4m**.

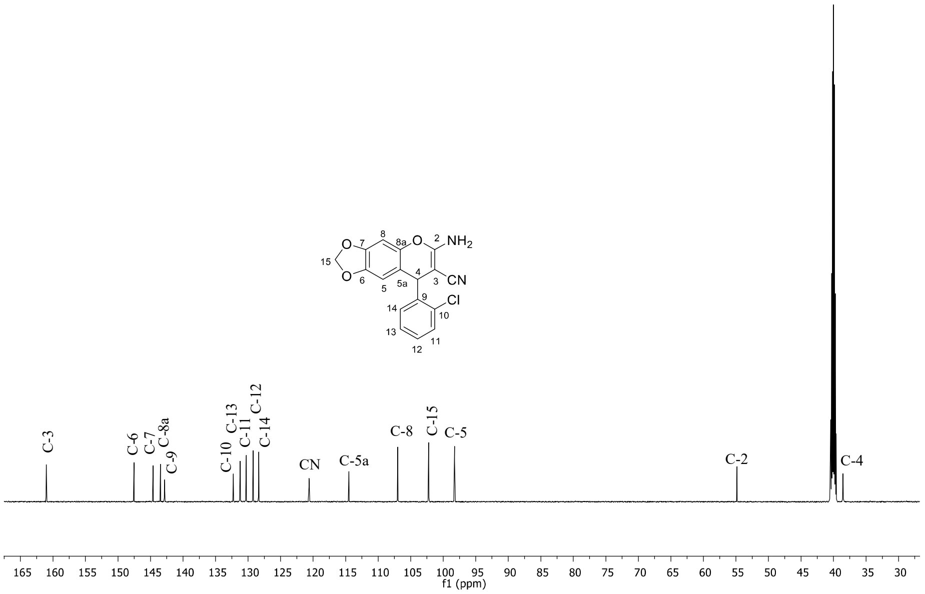

Figure S59. ^{13}C NMR (150 MHz, DMSO d_6) of 2-amino-3-cyano-4-(2-chlorophenyl)-6,7-methylendioxy-4*H*- chromene **4m**.

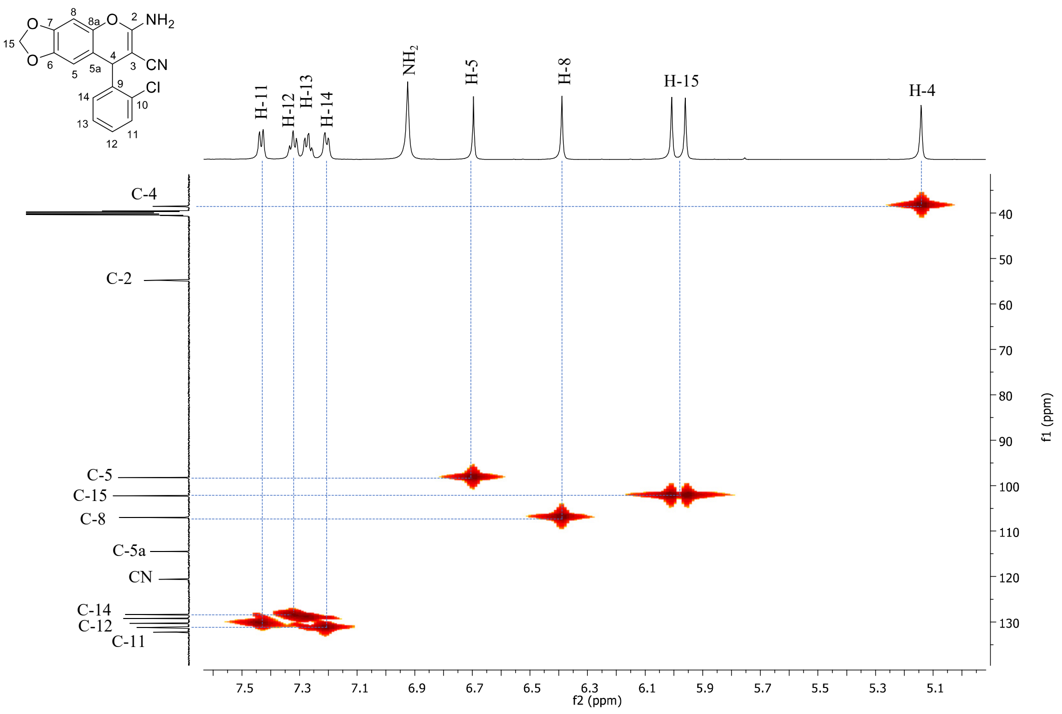

Figure S60. HMQC experiment of 2-amino-3-cyano-4-(2-chlorophenyl)-6,7-methylendioxy-4*H*- chromene **4m**.

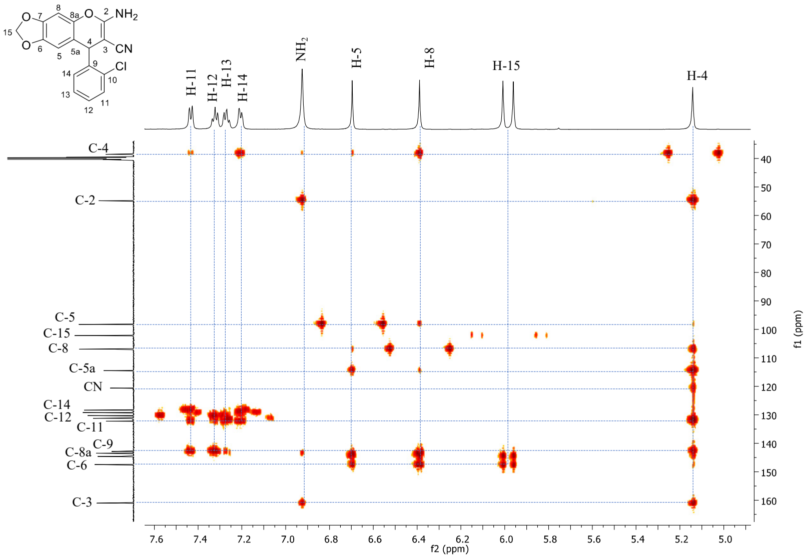

Figure S61. HMBC experiment of 2-amino-3-cyano-4-(2-chlorophenyl)-6,7-methylendioxy-4*H*- chromene **4m**.

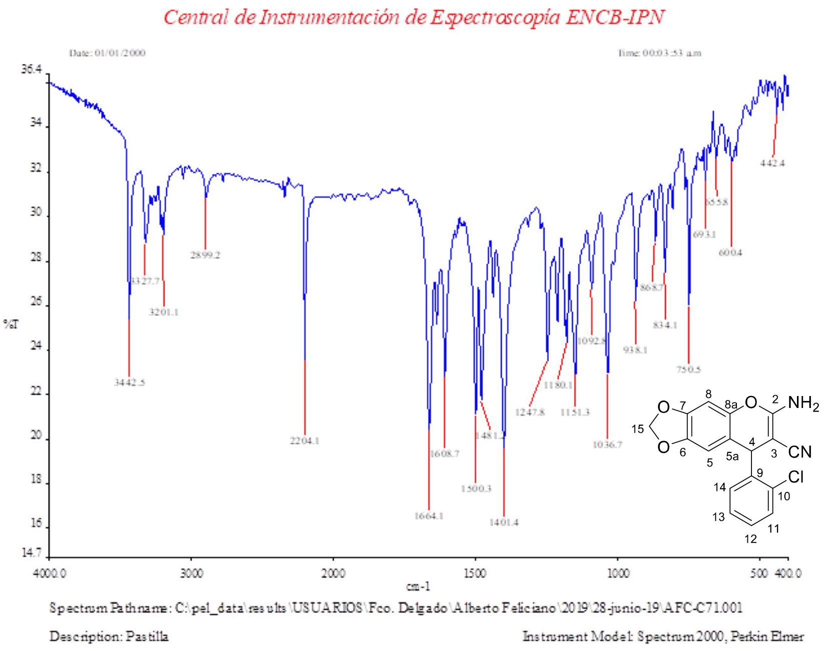

Figure S62. FT-IR of 2-amino-3-cyano-4-(2-chlorophenyl)-6,7-methylendioxy-4*H*-chromene **4m**.

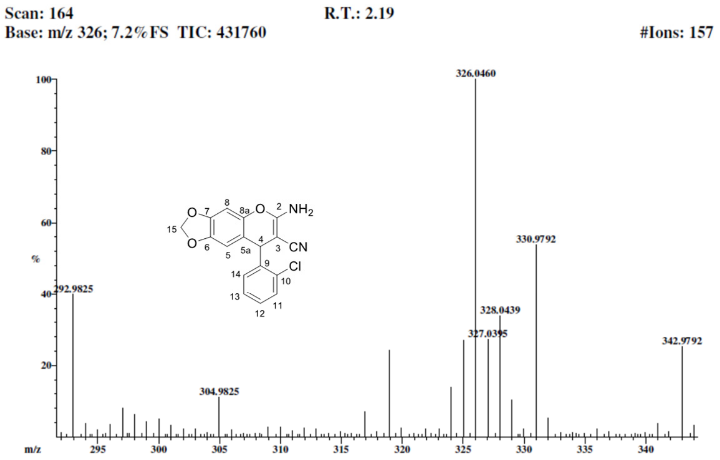

Selected Isotopes : $H_{0-11}C_{0-17}N_{0-2}O_{0-3}Cl_{0-1}$ Error Limit : 5 ppm

Measured Mass	% Base	Formula	Calculated Mass	Error
326.0460	100.0%	$C_{17}H_{11}N_2O_3Cl$	326.0458	0.5

Figure S63. HRMS (EI) $[M]^+$ of 2-amino-3-cyano-4-(2-chlorophenyl)-6,7-methylendioxy-4*H*- chromene **4m**.

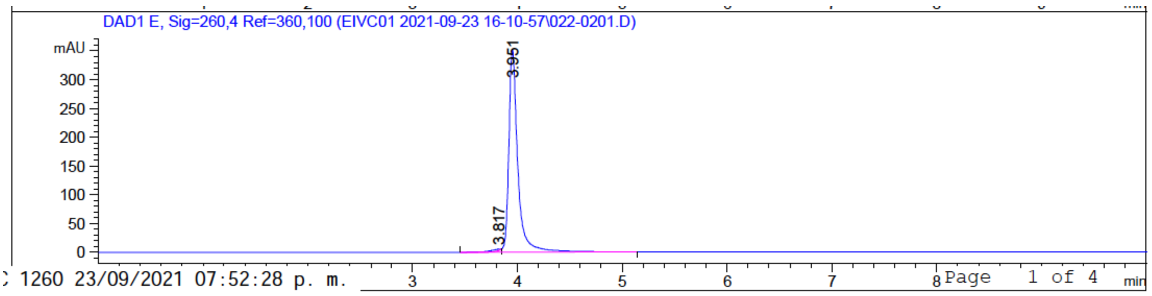

Signal 5: DAD1 E, Sig=260,4 Ref=360,100

Peak #	RetTime [min]	Type	Width [min]	Area [mAU*s]	Height [mAU]	Area %
1	3.817	BV E	0.1333	27.52771	2.71499	1.2859
2	3.951	VB R	0.0879	2113.27246	352.61096	98.7141
Totals :				2140.80017	355.32595	

Figure S64. **HPLC** of 2-amino-3-cyano-4-(2-chlorophenyl)-6,7-methylendioxy-4*H*-chromene **4m**. 0.1 mg/ml, MeCN, 0.4 ml/min, 260 nm, purity = 98.71%.

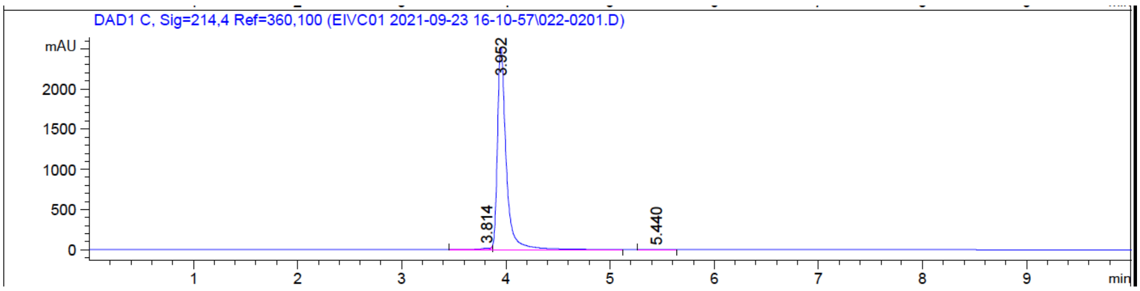

Signal 3: DAD1 C, Sig=214,4 Ref=360,100

Peak #	RetTime [min]	Type	Width [min]	Area [mAU*s]	Height [mAU]	Area %
1	3.814	BV E	0.0949	132.41058	19.32473	0.8905
2	3.952	VB R	0.0873	1.47258e4	2516.07080	99.0300
3	5.440	BB	0.1476	11.82748	1.11625	0.0795
Totals :				1.48700e4	2536.51178	

Figure S65. **HPLC** of 2-amino-3-cyano-4-(2-chlorophenyl)-6,7-methylendioxy-4*H*-chromene **4m**. 0.1 mg/ml, MeCN, 0.4 ml/min, 214 nm, purity = 99.03%.

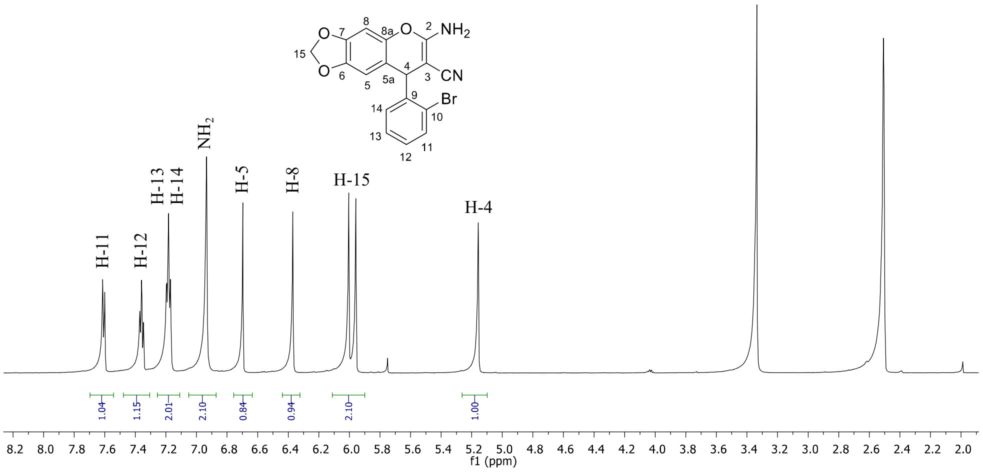

Figure S66. ^{1}H NMR (600 MHz, DMSO d_6) of 2-amino-3-cyano-4-(2-Bromophenyl)-6,7-methylendioxy-4*H*- chromene **4n**.

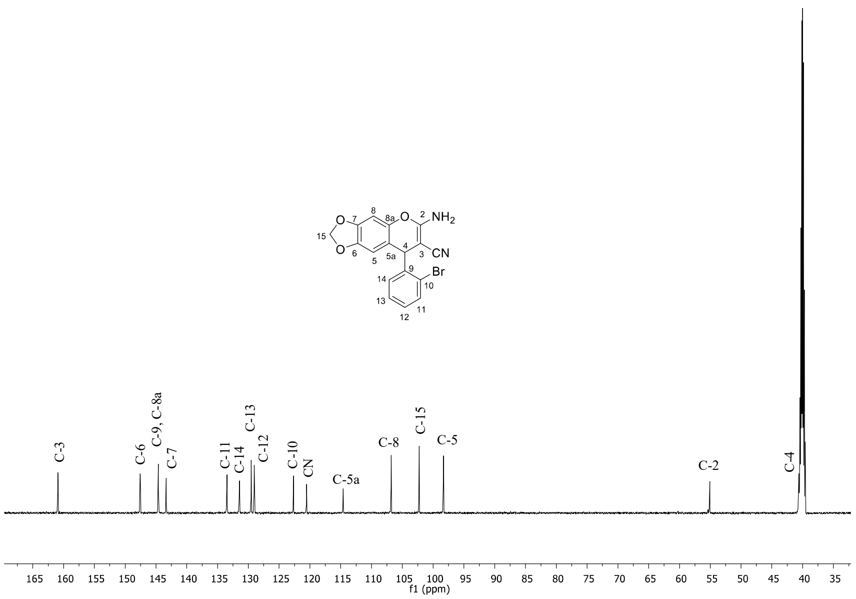

Figure S67. ^{13}C NMR (150 MHz, DMSO d_6) of 2-amino-3-cyano-4-(2-Bromophenyl)-6,7-methylendioxy-4*H*- chromene **4n**.

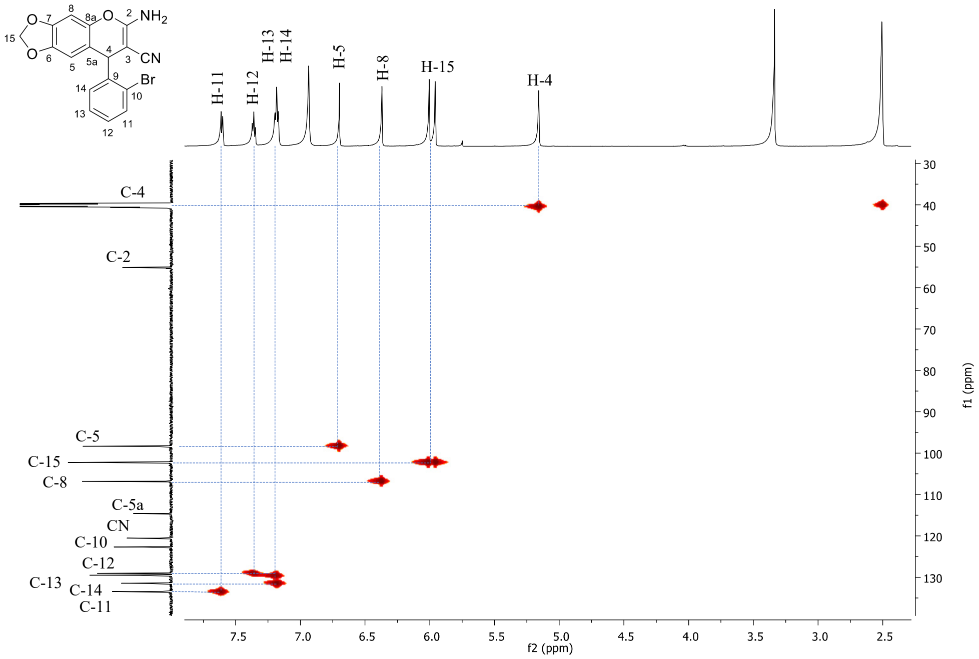

Figure S68. HMQC experiment of 2-amino-3-cyano-4-(2-Bromophenyl)-6,7-methylendioxy-4*H*- chromene **4n**.

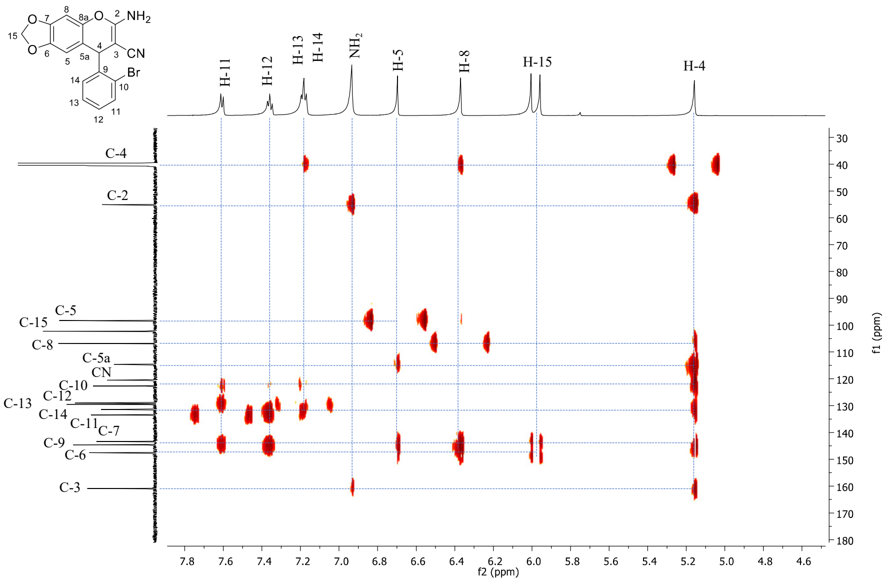

Figure S69. HMBC experiment of 2-amino-3-cyano-4-(2-Bromophenyl)-6,7-methylendioxy-4*H*- chromene **4n**.

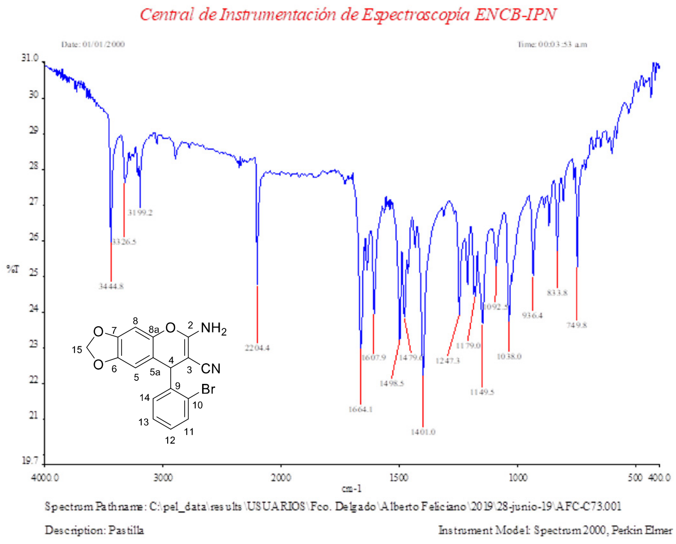

Figure S70. FT-IR of 2-amino-3-cyano-4-(2-Bromophenyl)-6,7-methylendioxy-4*H*-chromene **4n**.

Scan: 305 R.T.: 4.08
Base: m/z 370; 18.2%FS TIC: 836880 #Ions: 137

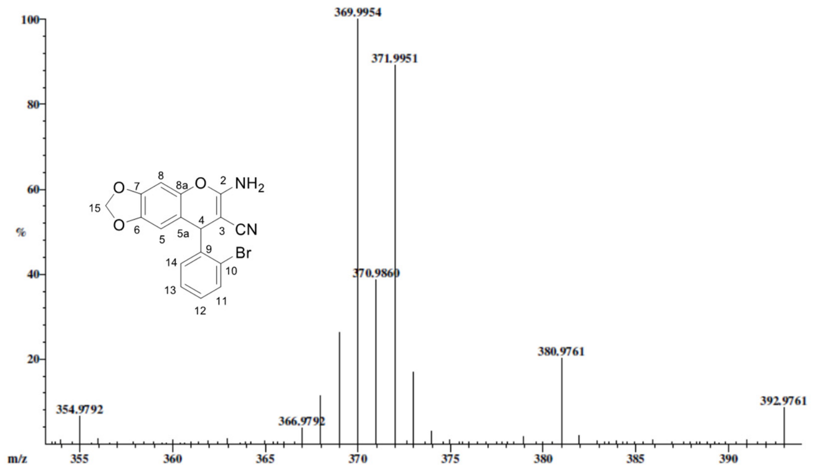

Selected Isotopes : $H_{0-11}C_{0-17}N_{0-2}O_{0-3}Br_{0-1}$ Error Limit : 5 ppm

Measured Mass	% Base	Formula	Calculated Mass	Error
369.9954	100.0%	$C_{17}H_{11}N_2O_3Br$	369.9953	0.3

Figure S71. HRMS (EI) $[M]^+$ of 2-amino-3-cyano-4-(2-Bromophenyl)-6,7-methylendioxy-4*H*- chromene **4n**.

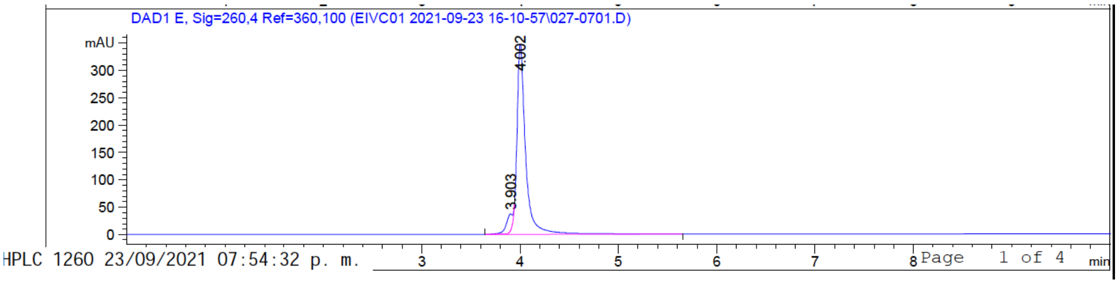

Signal 5: DAD1 E, Sig=260,4 Ref=360,100

Peak #	RetTime [min]	Type	Width [min]	Area [mAU*s]	Height [mAU]	Area %
1	3.903	BV E	0.0703	145.62874	30.16978	6.2830
2	4.002	VB R	0.0908	2172.20850	347.93692	93.7170
Totals :				2317.83723	378.10670	

Figure S72. **HPLC** of 2-amino-3-cyano-4-(2-Bromophenyl)-6,7-methylendioxy-4*H*-chromene **4n**. 0.1 mg/ml, MeCN, 0.4 ml/min, 260 nm, purity = 93.71 %

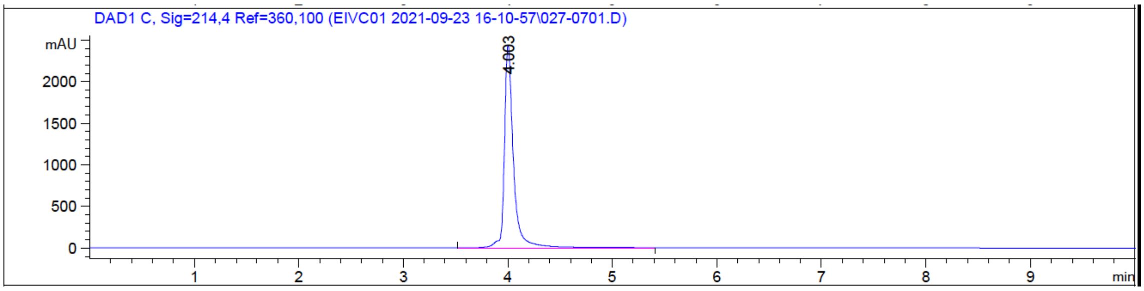

Signal 3: DAD1 C, Sig=214,4 Ref=360,100

Peak #	RetTime [min]	Type	Width [min]	Area [mAU*s]	Height [mAU]	Area %
1	4.003	BB	0.0883	1.46719e4	2434.05811	100.0000
Totals :				1.46719e4	2434.05811	

Figure S73. **HPLC** of 2-amino-3-cyano-4-(2-Bromophenyl)-6,7-methylendioxy-4*H*-chromene **4n**. 0.1 mg/ml, MeCN, 0.4 ml/min, 214 nm, purity = 100 %

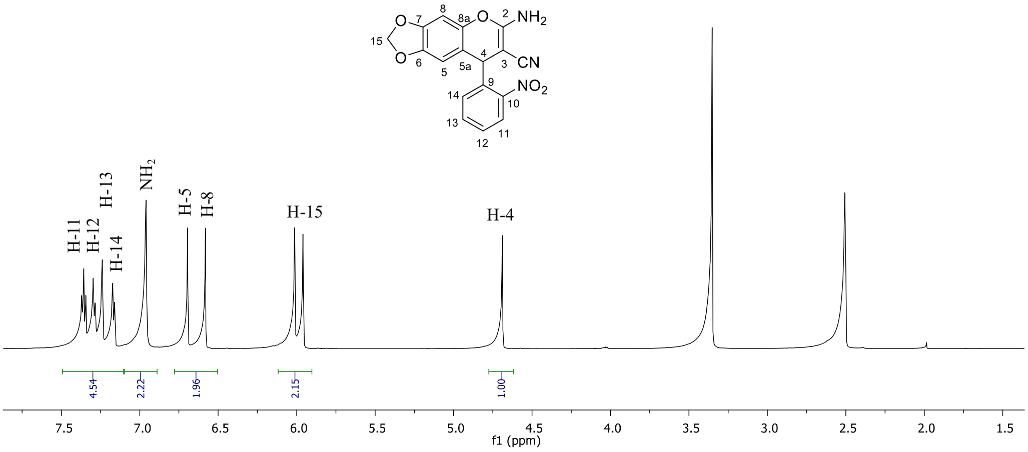

Figure S74. ^{1}H NMR (600 MHz, DMSO d6) of 2-amino-3-cyano-4-(2-nitrophenyl)-6,7-methylendioxy-4*H*- chromene **4o**.

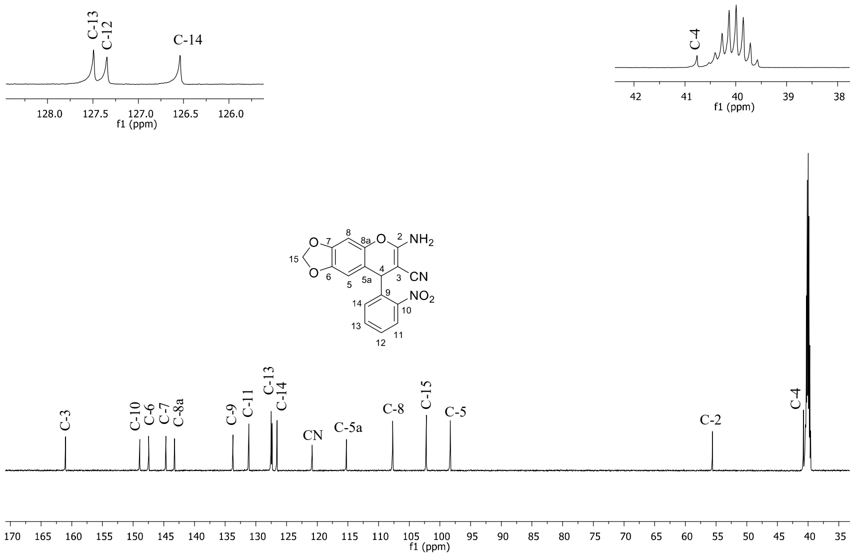

Figure S75. ^{13}C NMR (150 MHz, DMSO d$_6$) of 2-amino-3-cyano-4-(2-nitrophenyl)-6,7-methylendioxy-4*H*- chromene **4o**.

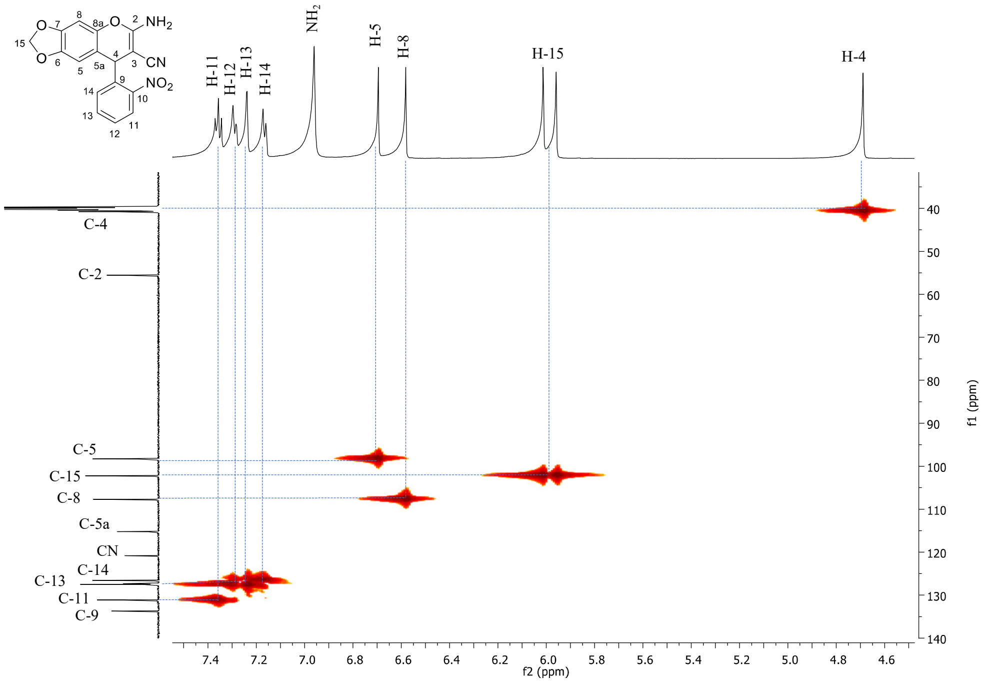

Figure S76. HMQC experiment of 2-amino-3-cyano-4-(2-nitrophenyl)-6,7-methylendioxy-4*H*- chromene **4o**.

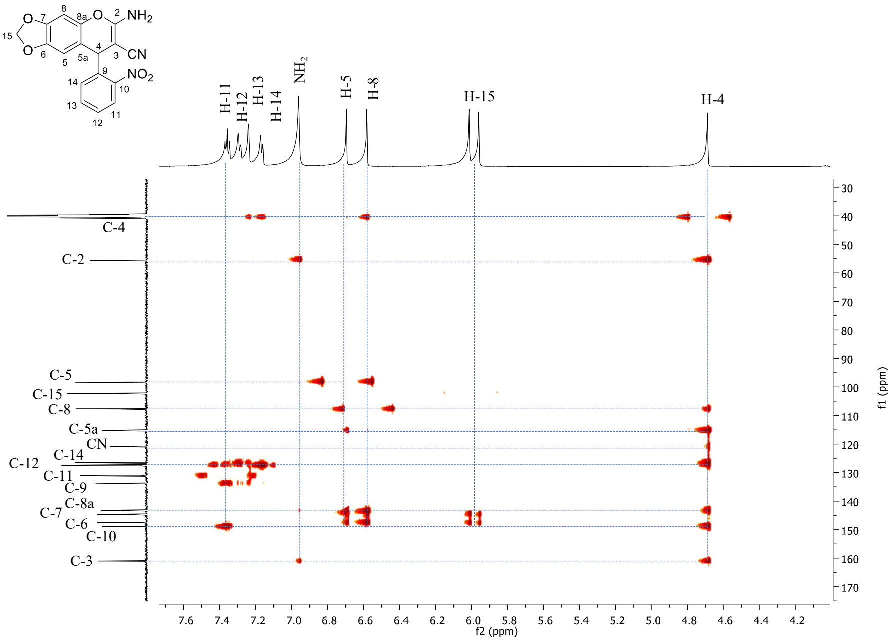

Figure S77. HMBC experiment of 2-amino-3-cyano-4-(2-nitrophenyl)-6,7-methylendioxy-4*H*- chromene **4o**.

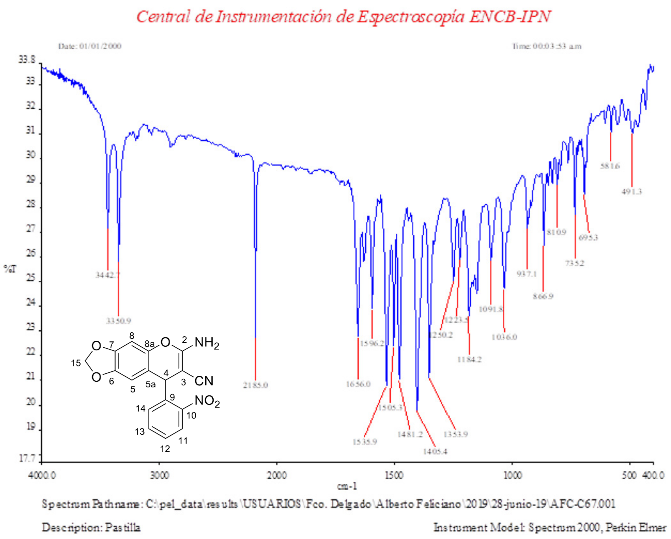

Figure S78. FT-IR of 2-amino-3-cyano-4-(2-nitrophenyl)-6,7-methylendioxy-4*H*-chromene **4o**.

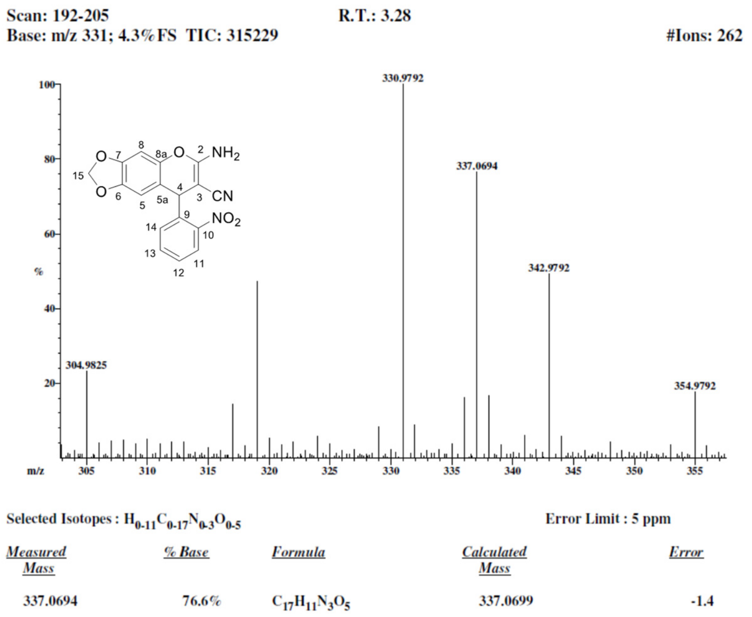

Figure S79. HRMS (EI) $[M]^+$ of 2-amino-3-cyano-4-(2-nitrophenyl)-6,7-methylendioxy-4*H*- chromene **4o**.

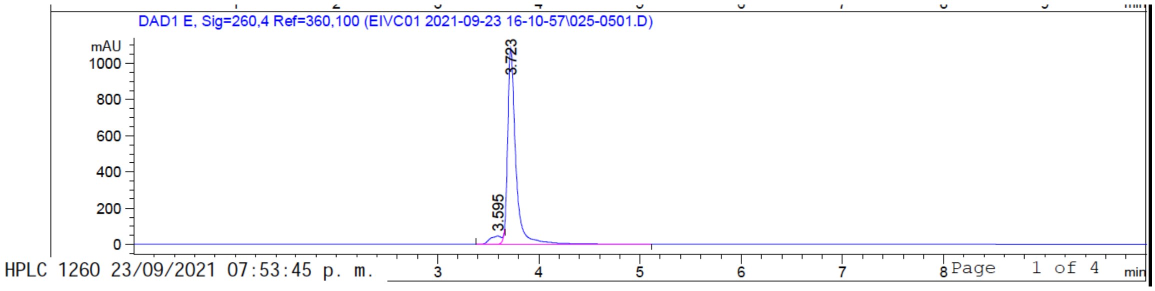

Signal 5: DAD1 E, Sig=260,4 Ref=360,100

Peak #	RetTime [min]	Type	Width [min]	Area [mAU*s]	Height [mAU]	Area %
1	3.595	BV E	0.1043	343.26083	44.86161	5.2996
2	3.723	VB R	0.0819	6133.83594	1085.74829	94.7004
Totals :				6477.09677	1130.60991	

Figure S80. **HPLC** of 2-amino-3-cyano-4-(2-nitrophenyl)-6,7-methylendioxy-4*H*-chromene **4o**. 0.1 mg/ml, MeCN, 0.4 ml/min, 260 nm, purity = 94.7 %

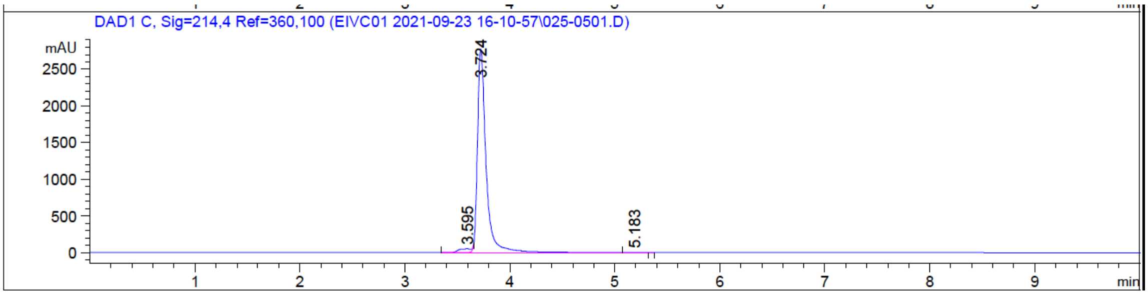

Signal 3: DAD1 C, Sig=214,4 Ref=360,100

Peak #	RetTime [min]	Type	Width [min]	Area [mAU*s]	Height [mAU]	Area %
1	3.595	BV E	0.1067	410.60367	51.10603	2.5138
2	3.724	VV R	0.0850	1.59171e4	2771.15576	97.4493
3	5.183	VB E	0.0887	6.02624	1.03874	0.0369
Totals :				1.63337e4	2823.30053	

Figure S81. **HPLC** of 2-amino-3-cyano-4-(2-nitrophenyl)-6,7-methylendioxy-4*H*-chromene **4o**. 0.1 mg/ml, MeCN, 0.4 ml/min, 214 nm, purity = 97.45 %

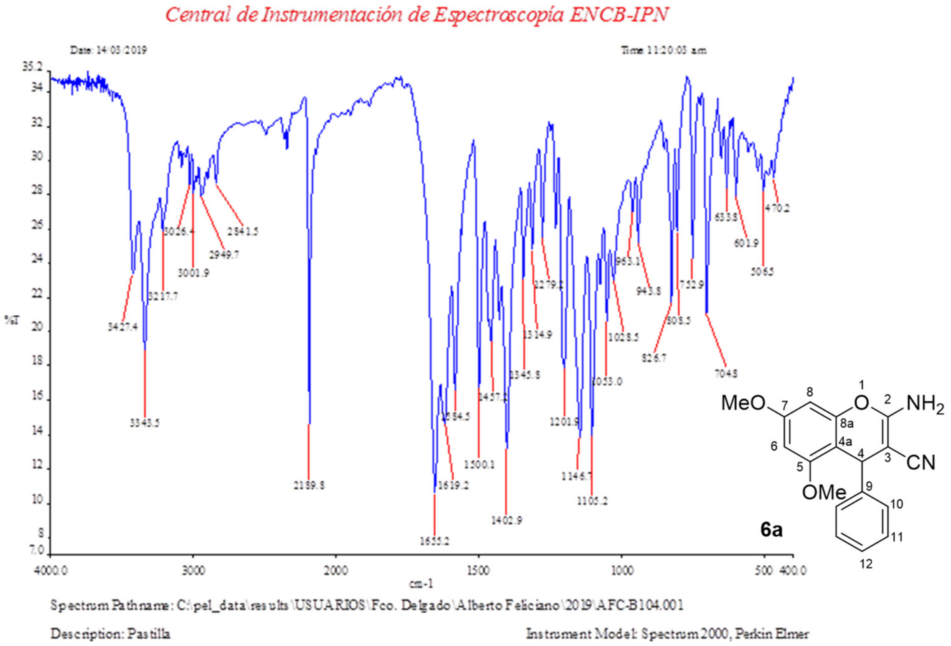

Figure S82. FT-IR of 2-Amino-3-cyano-5,7-dimethoxy-4-phenyl-4*H*-chromene **6a**.

File: FDR-AFC-B104 Date Run: 02-13-2019 (Time Run: 15:47:40)
Sample: FDR-AFC-B104
Instrument: JEOL GCmate
Inlet: Direct Probe Ionization mode: EI+

Scan: 280 R.T.: 3.71
Base: m/z 331; 2.1%FS TIC: 229152 #Ions: 179

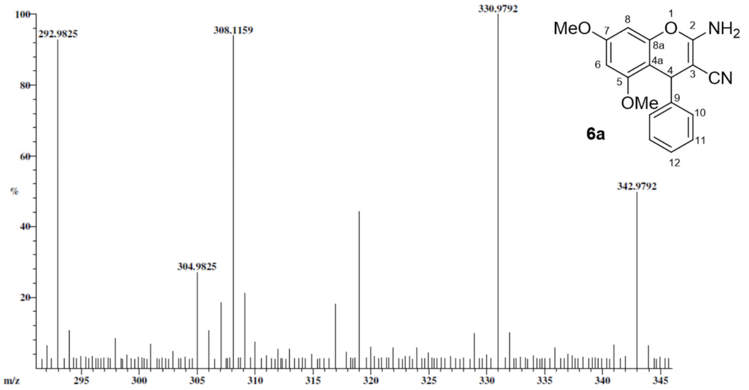

Selected Isotopes : $H_{0-16}C_{0-18}N_{0-2}O_{0-3}$ Error Limit : 5 ppm

Measured Mass	% Base	Formula	Calculated Mass	Error
308.1159	93.9%	$C_{18}H_{16}N_2O_3$	308.1161	-0.6

Figure S83. HRMS (EI) [M]$^+$ of 2-Amino-3-cyano-5,7-dimethoxy-4-phenyl-4*H*-chromene **6a**.

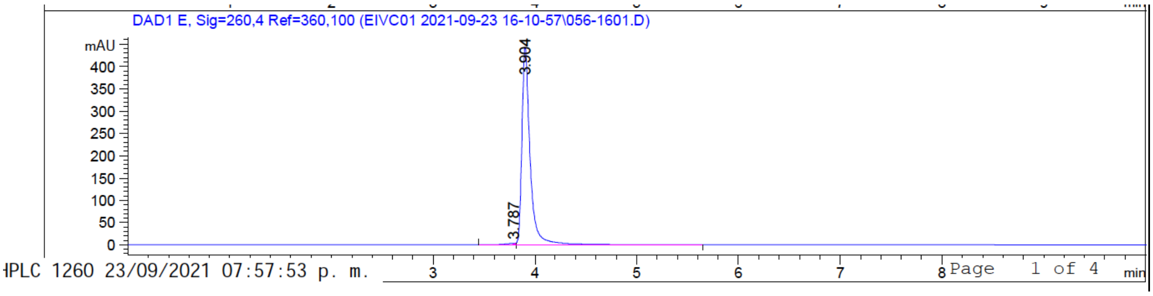

Signal 5: DAD1 E, Sig=260,4 Ref=360,100

Peak #	RetTime [min]	Type	Width [min]	Area [mAU*s]	Height [mAU]	Area %
1	3.787	BV E	0.1171	23.35457	2.77857	0.9210
2	3.904	VB R	0.0831	2512.35229	443.26486	99.0790

Totals : 2535.70687 446.04343

Figure S84. **HPLC** of 2-Amino-3-cyano-5,7-dimethoxy-4-phenyl-4*H*-chromene **6a**. 0.1 mg/ml, MeCN, 0.4 ml/min, 260 nm, purity = 99.08%

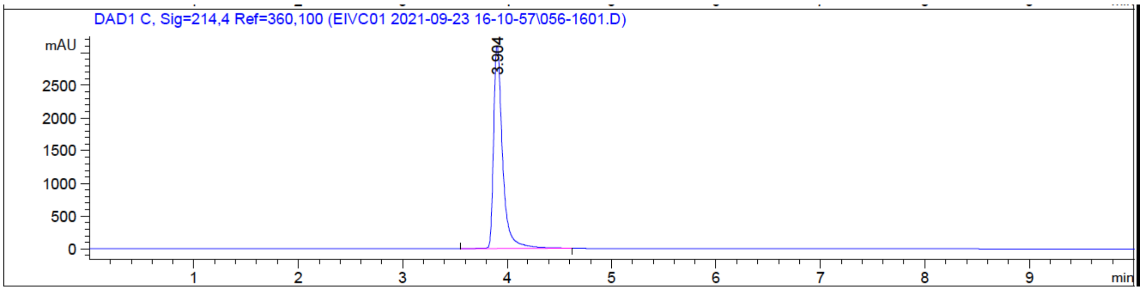

Signal 3: DAD1 C, Sig=214,4 Ref=360,100

Peak #	RetTime [min]	Type	Width [min]	Area [mAU*s]	Height [mAU]	Area %
1	3.904	BB	0.0903	1.86459e4	3092.75073	100.0000
Totals :				1.86459e4	3092.75073	

Figure S85. **HPLC** of 2-Amino-3-cyano-5,7-dimethoxy-4-phenyl-4*H*-chromene **6a**. 0.1 mg/ml, MeCN, 0.4 ml/min, 214 nm, purity = 100%

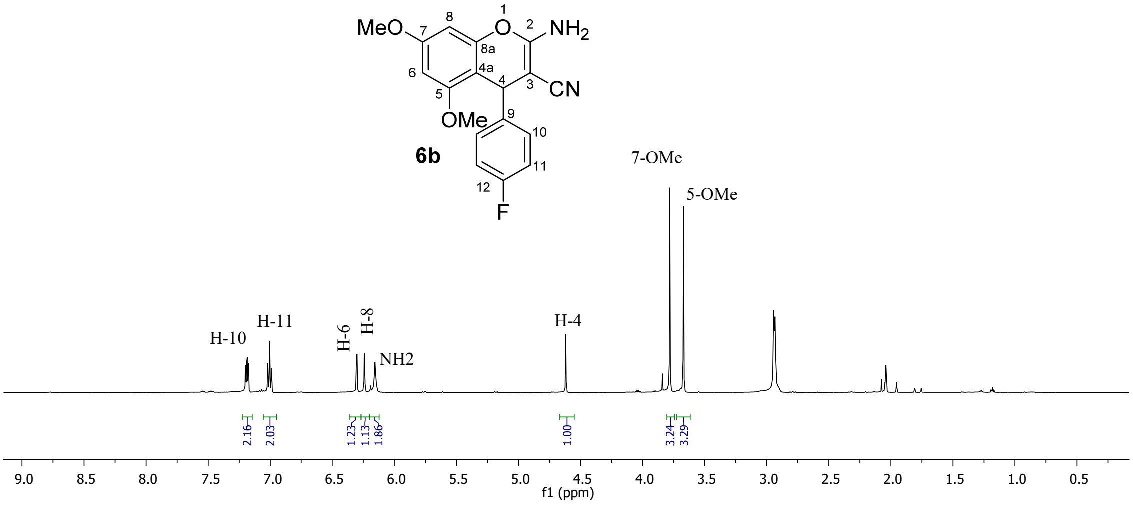

Figure S86. ^{1}H NMR (600 MHz, acetone d$_6$) of 2-Amino-3-cyano-5,7-dimethoxy-4-(4-fluorophenyl)-4*H*-chromene **6b**.

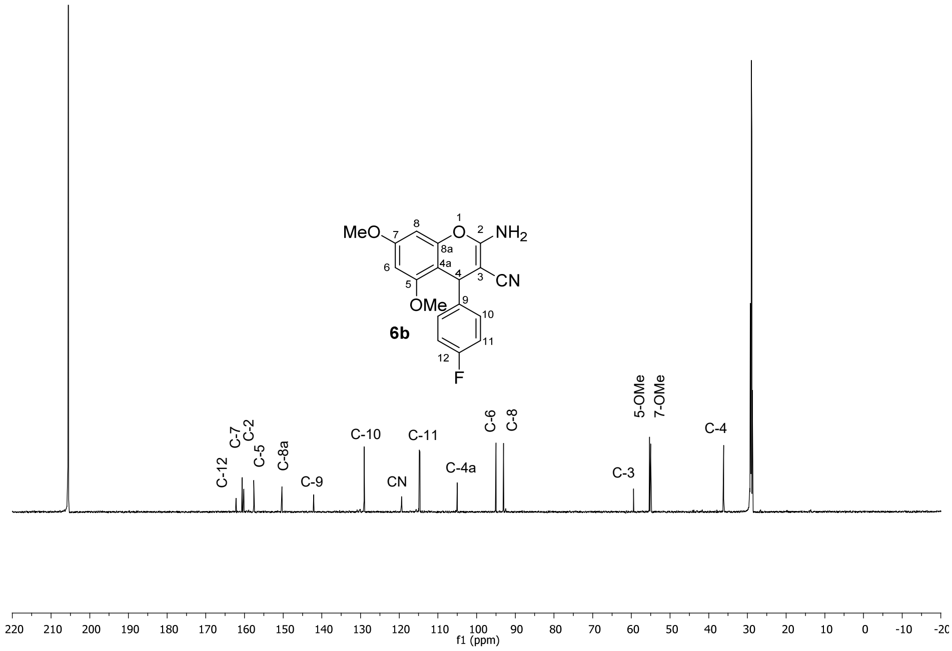

Figure S87. ^{13}C NMR (150 MHz, acetone d$_6$) of 2-Amino-3-cyano-5,7-dimethoxy-4-(4-fluorophenyl)-4*H*-chromene **6b**.

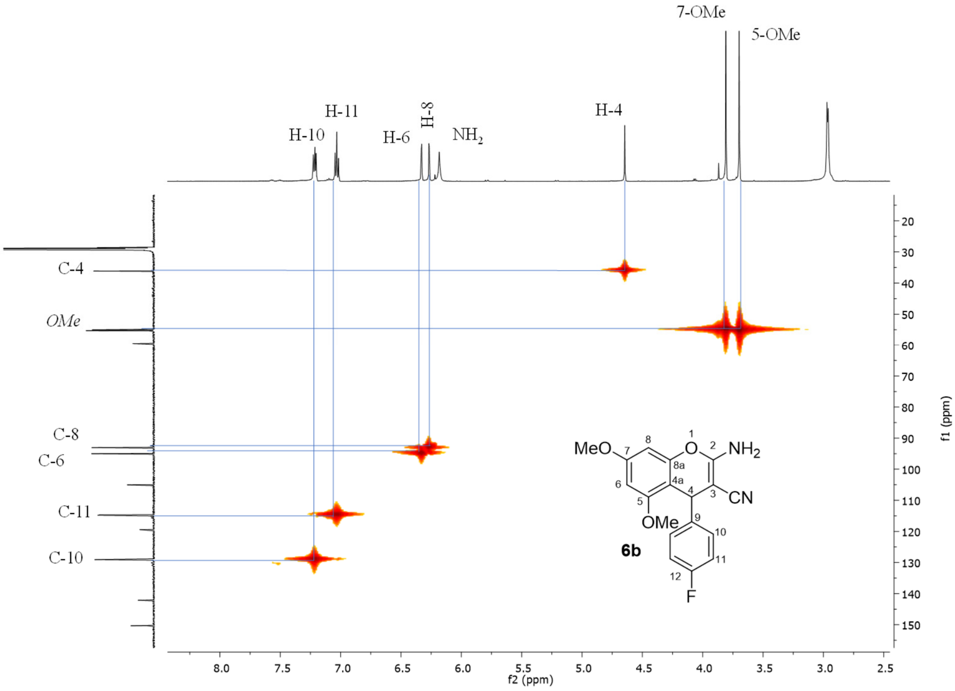

Figure S88. HMQC experiment of 2-Amino-3-cyano-5,7-dimethoxy-4-(4-fluorophenyl)-4*H*-chromene **6b**.

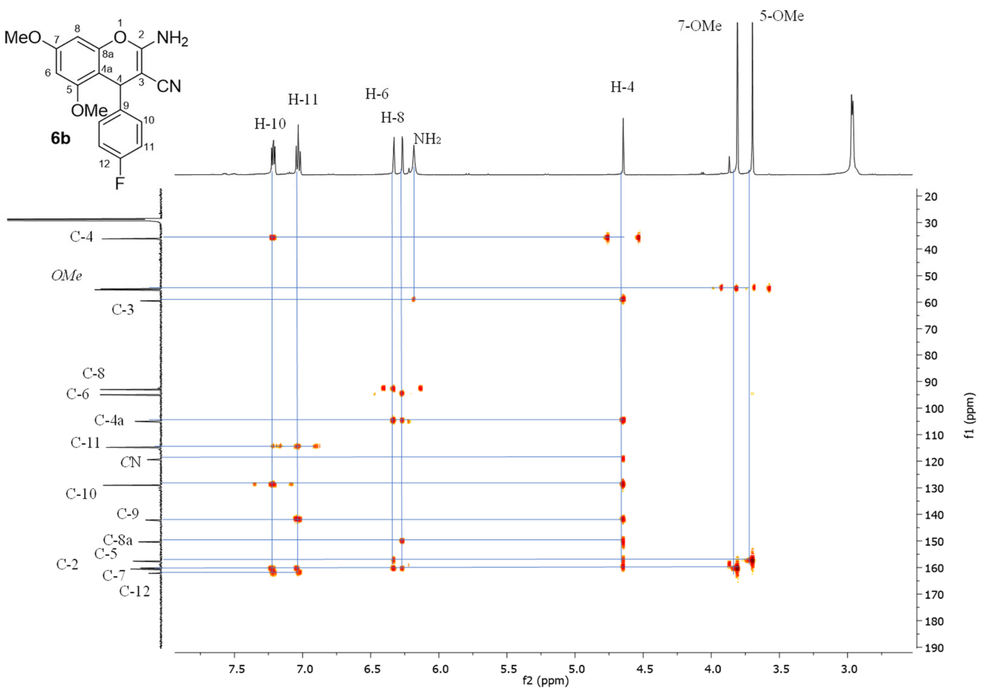

Figure S89. HMBC experiment of 2-Amino-3-cyano-5,7-dimethoxy-4-(4-fluorophenyl)-4*H*-chromene **6b**.

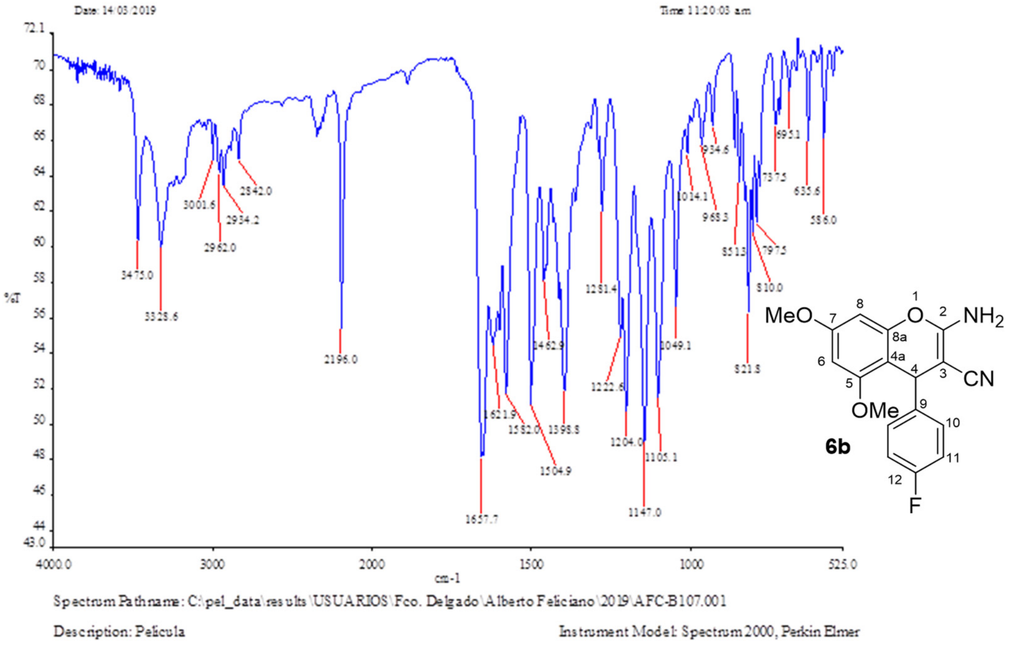

Figure S90. FT-IR of 2-Amino-3-cyano-5,7-dimethoxy-4-(4-fluorophenyl)-4*H*-chromene **6b**.

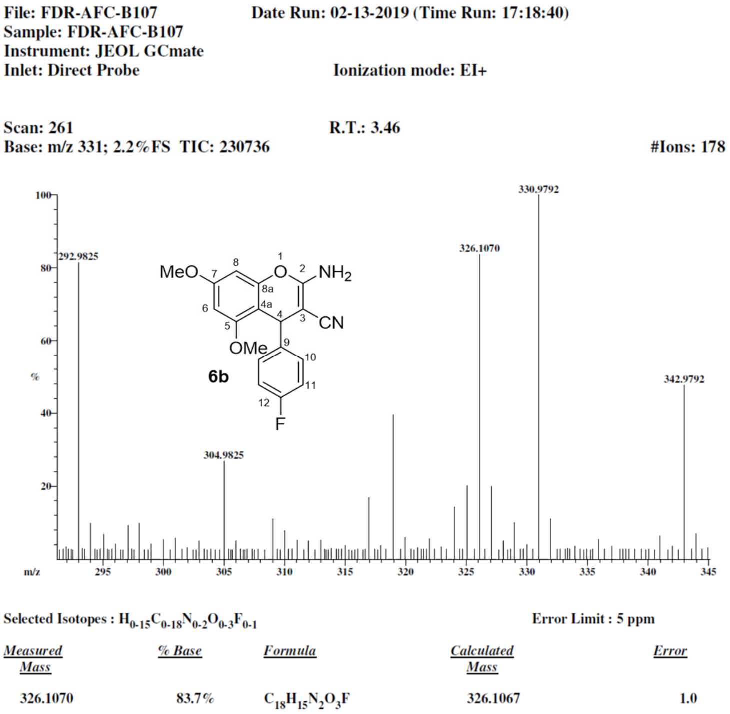

Figure S91. HRMS (EI) $[M]^+$ of 2-Amino-3-cyano-5,7-dimethoxy-4-(4-fluorophenyl)-4*H*-chromene **6b**.

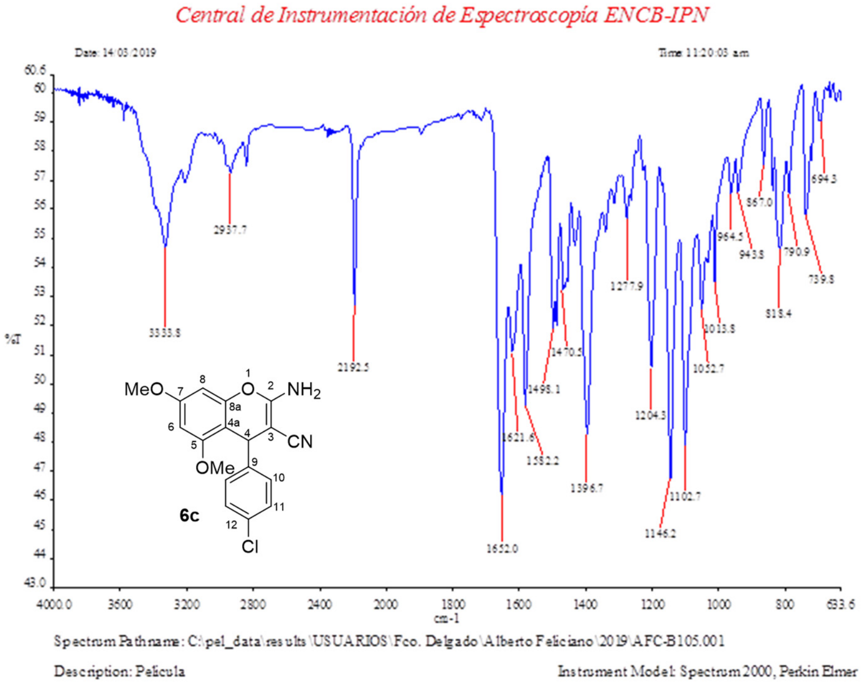

Figure S92. FT-IR of 2-Amino-3-cyano-5,7-dimethoxy-4-(4-chlorophenyl)-4*H*-chromene **6c**.

File: FDR-AFC-B105 Date Run: 02-20-2019 (Time Run: 17:14:45)
Sample: FDR-AFC-B105
Instrument: JEOL GCmate
Inlet: Direct Probe Ionization mode: EI+

Scan: 400 R.T.: 6.6
Base: m/z 331; 2.5%FS TIC: 222880 #Ions: 189

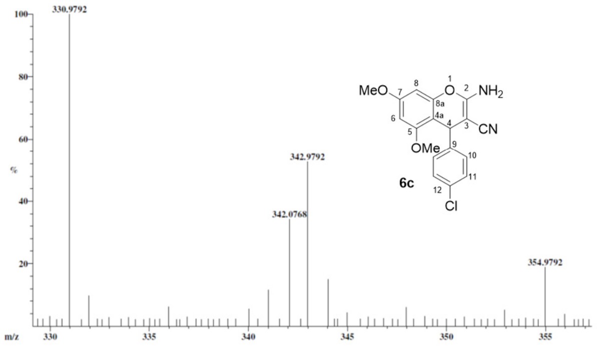

Selected Isotopes : $H_{0-15}C_{0-18}N_{0-2}O_{0-3}Cl_{0-1}$ Error Limit : 5 ppm

Measured Mass	% Base	Formula	Calculated Mass	Error
342.0768	34.2%	$C_{18}H_{15}N_2O_3Cl$	342.0771	-0.9

Figure S93. HRMS (EI) $[M]^+$ of 2-Amino-3-cyano-5,7-dimethoxy-4-(4-chlorophenyl)-4*H*-chromene **6c**.

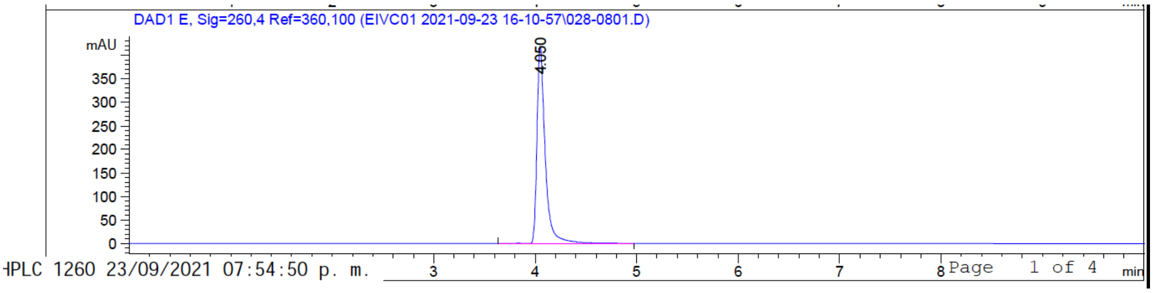

Signal 5: DAD1 E, Sig=260,4 Ref=360,100

Peak #	RetTime [min]	Type	Width [min]	Area [mAU*s]	Height [mAU]	Area %
1	4.050	VB R	0.0842	2376.26392	418.70908	100.0000
Totals :				2376.26392	418.70908	

Figure S94. **HPLC** of 2-Amino-3-cyano-5,7-dimethoxy-4-(4-chlorophenyl)-4*H*-chromene **6c**. 0.1 mg/ml, MeCN, 0.4 ml/min, 260 nm, purity = 100 %

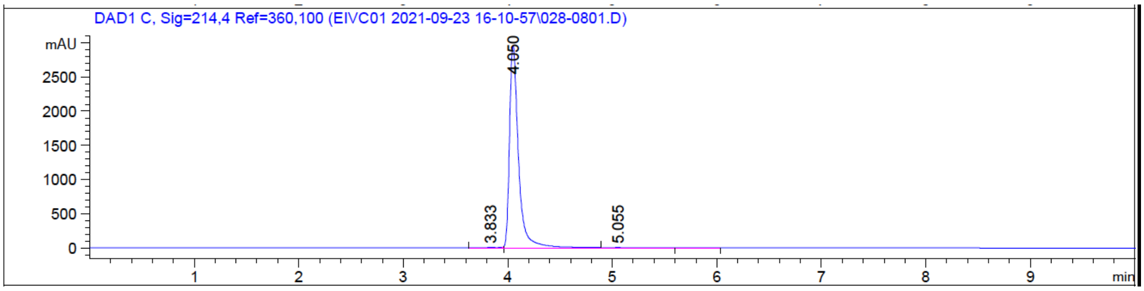

Signal 3: DAD1 C, Sig=214,4 Ref=360,100

Peak #	RetTime [min]	Type	Width [min]	Area [mAU*s]	Height [mAU]	Area %
1	3.833	BV E	0.1225	56.52501	6.31154	0.3152
2	4.050	VV R	0.0904	1.78567e4	2957.13623	99.5889
3	5.055	VB E	0.1302	17.18803	1.96165	0.0959
Totals :				1.79304e4	2965.40942	

Figure S95. **HPLC** of 2-Amino-3-cyano-5,7-dimethoxy-4-(4-chlorophenyl)-4*H*-chromene **6c**. 0.1 mg/ml, MeCN, 0.4 ml/min, 214 nm, purity = 99.6 %

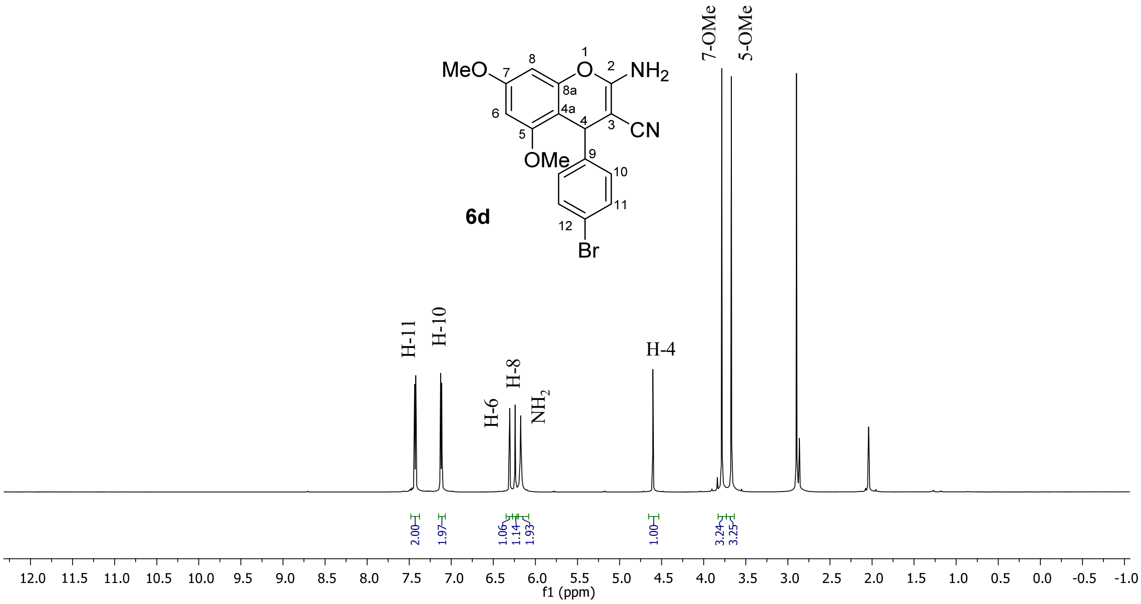

Figure S96. ^{1}H NMR (600 MHz, acetone d$_6$) of 2-Amino-4-(4-bromophenyl)-3-cyano-5,7-dimethoxy-4*H*-chromene **6d**.

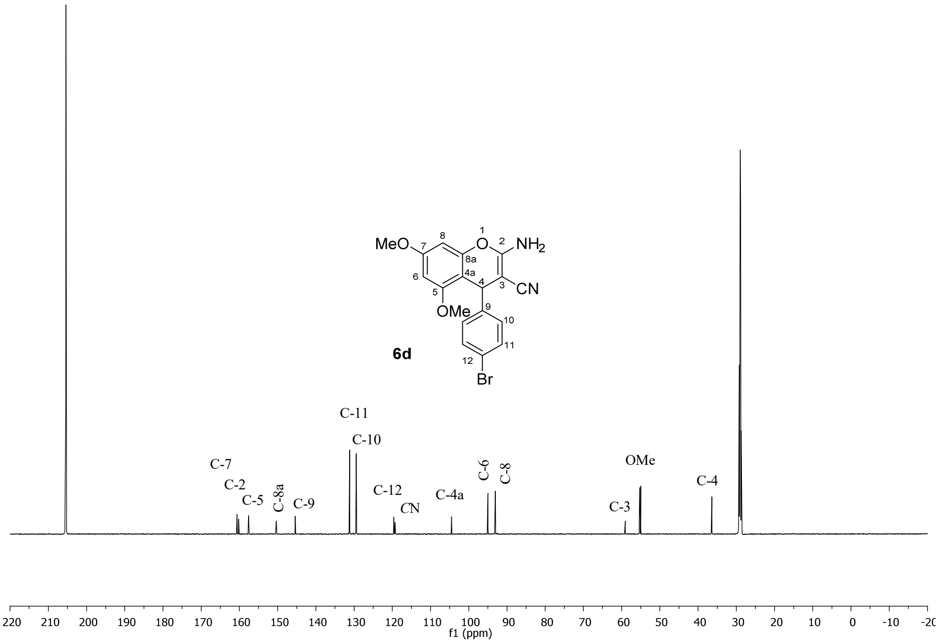

Figure S97. ^{13}C NMR (150 MHz, acetone d$_6$) of 2-Amino-4-(4-bromophenyl)-3-cyano-5,7-dimethoxy-4*H*-chromene **6d**.

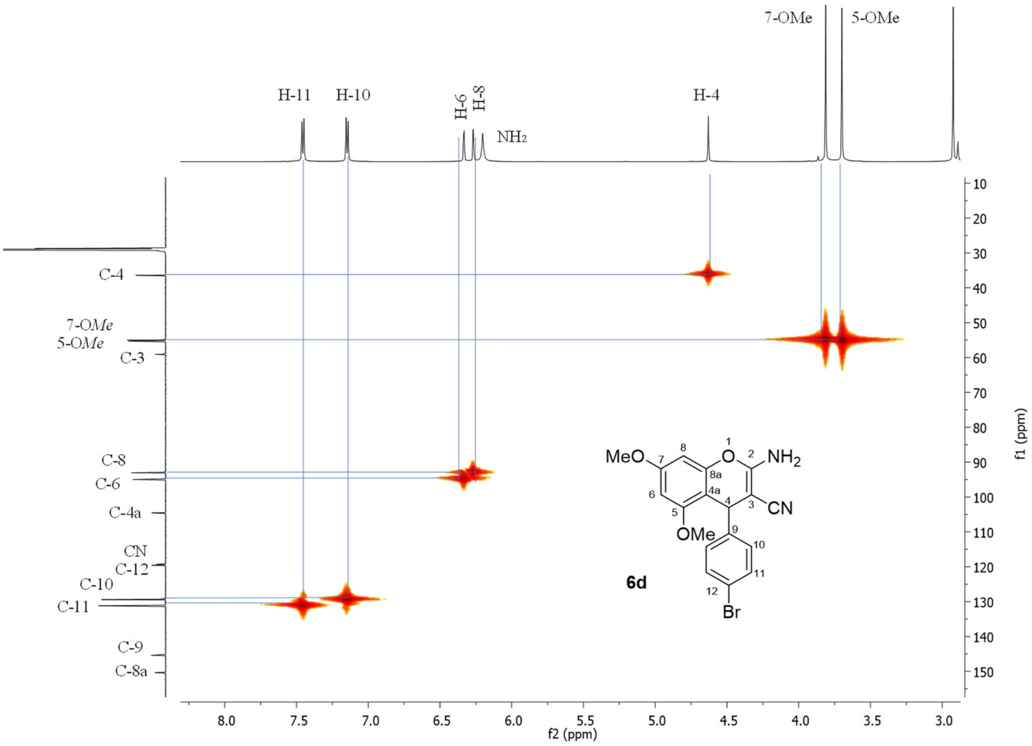

Figure S98. HMQC experiment of 2-Amino-4-(4-bromophenyl)-3-cyano-5,7-dimethoxy-4*H*-chromene **6d.**

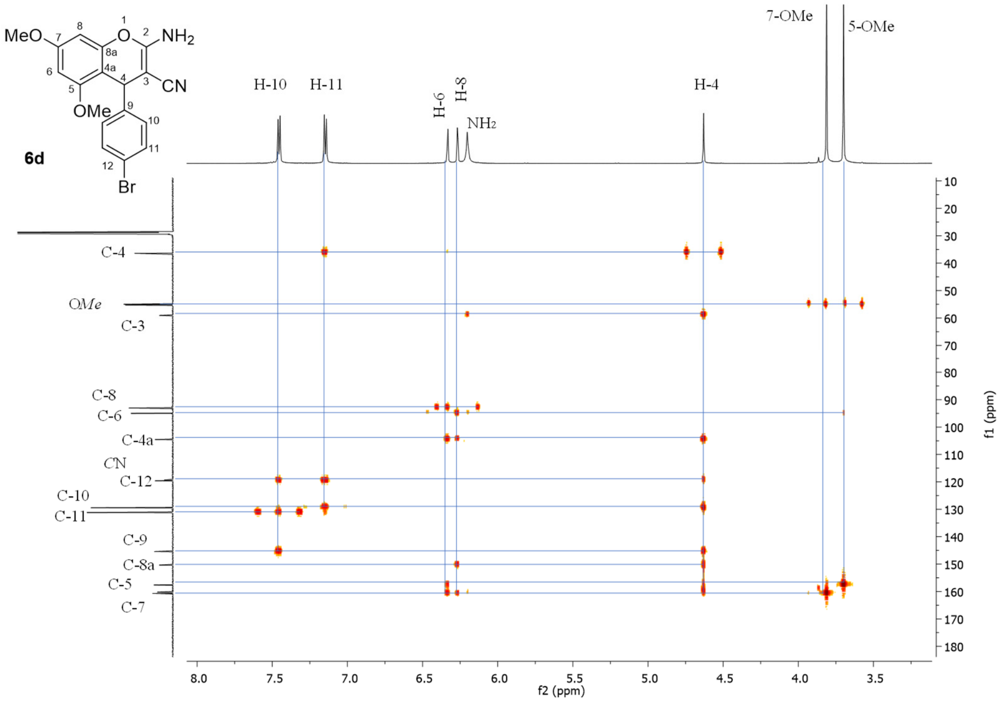

Figure S99. HMBC experiment of 2-Amino-4-(4-bromophenyl)-3-cyano-5,7-dimethoxy-4*H*-chromene **6d**.

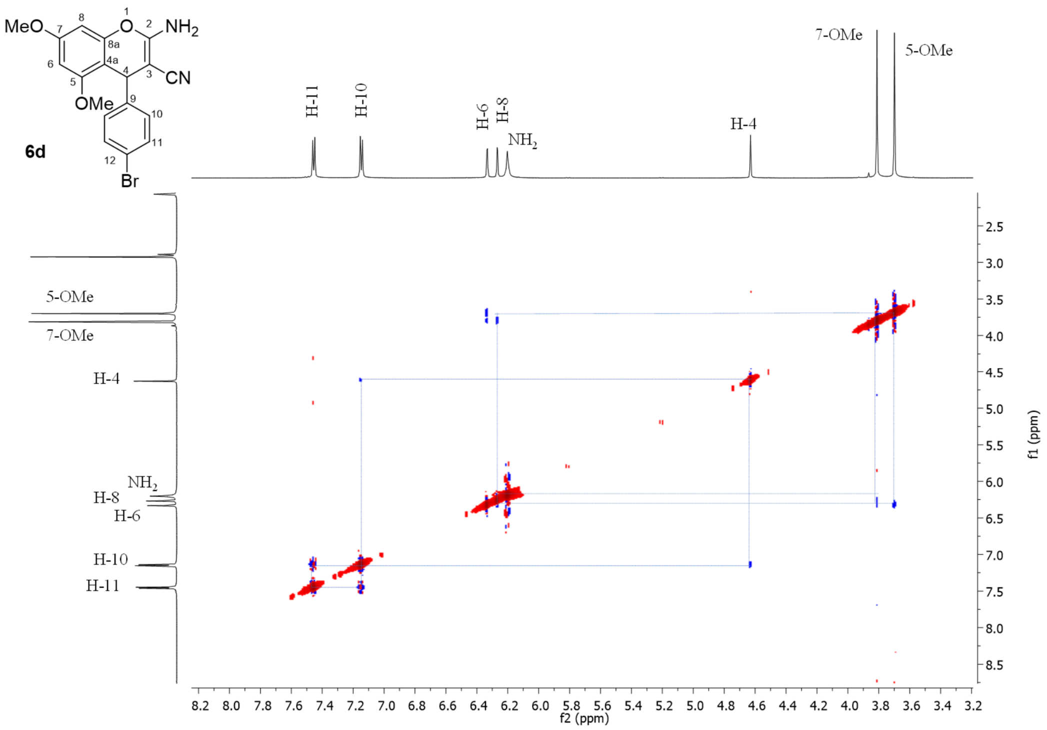

Figure S100. NOESY experiment of 2-Amino-4-(4-bromophenyl)-3-cyano-5,7-dimethoxy-4*H*-chromene **6d**.

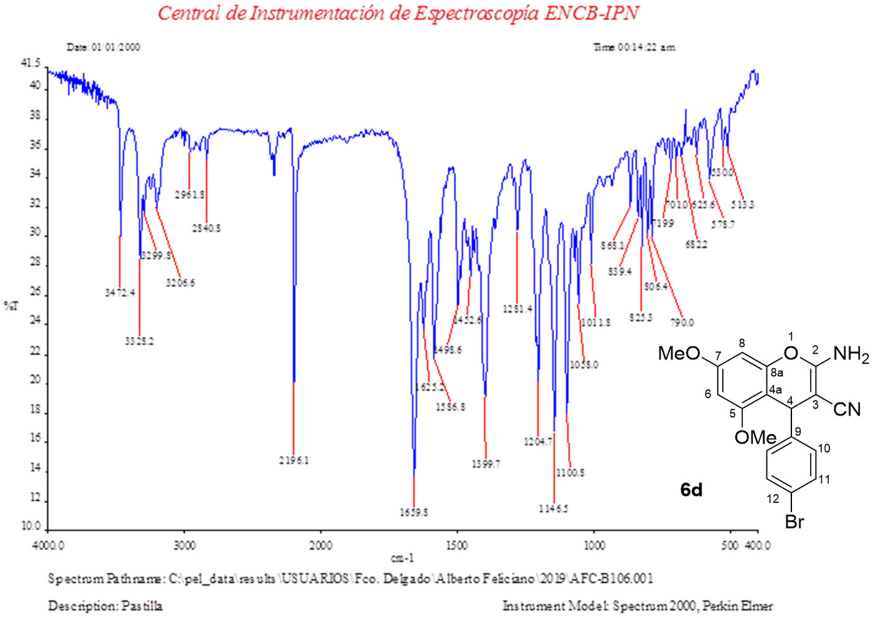

Figure S101. FT-IR of 2-Amino-4-(4-bromophenyl)-3-cyano-5,7-dimethoxy-4*H*-chromene **6d**.

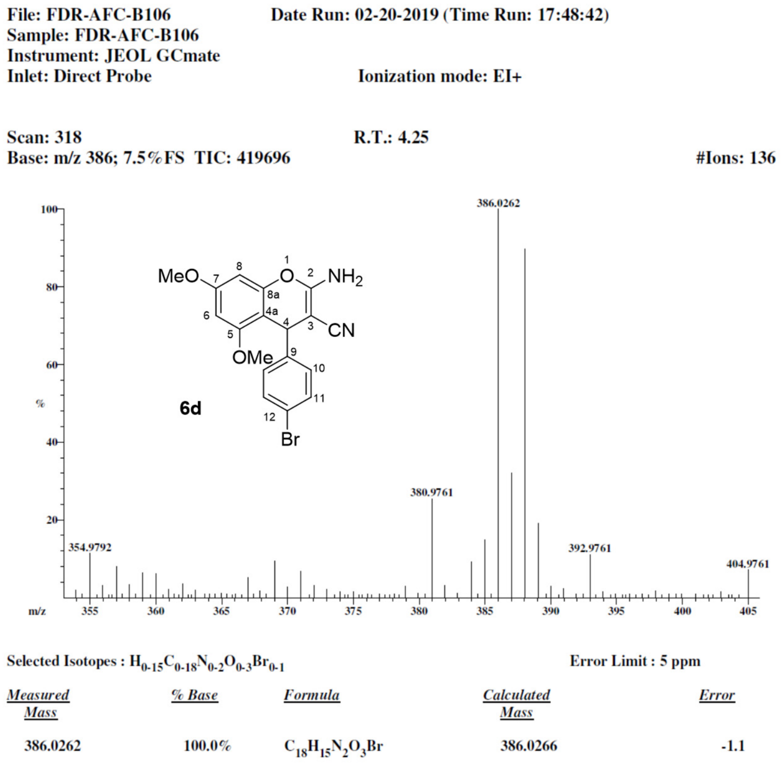

Figure S102. HRMS (EI) $[M]^+$ of 2-Amino-4-(4-bromophenyl)-3-cyano-5,7-dimethoxy-4*H*-chromene **6d**.

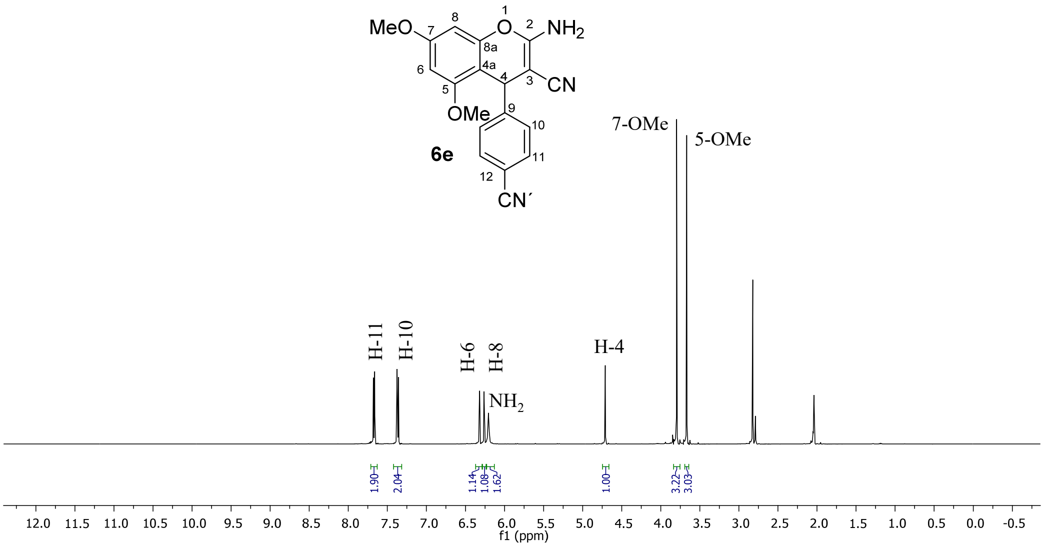

Figure S103. ^{1}H NMR (600 MHz, acetone d$_6$) of 2-Amino-3-cyano-4-(4-cyanophenyl)-5,7-dimethoxy-4*H*-chromene **6e**.

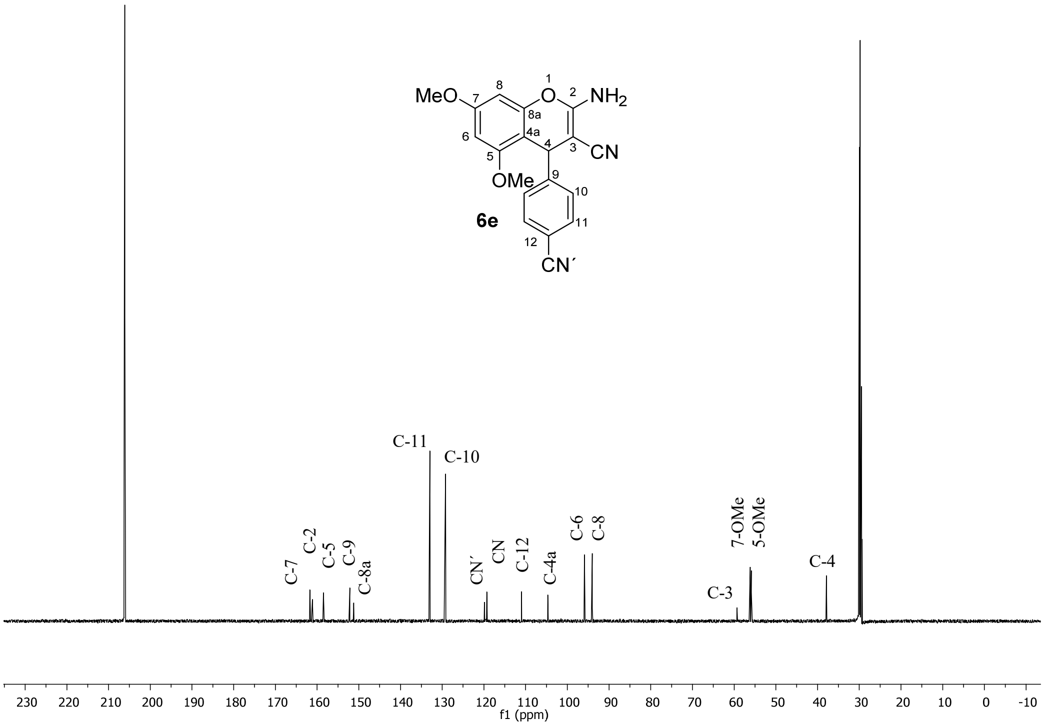

Figure S104. ^{13}C NMR (150 MHz, acetone d$_6$) of 2-Amino-3-cyano-4-(4-cyanophenyl)-5,7-dimethoxy-4*H*-chromene **6e**.

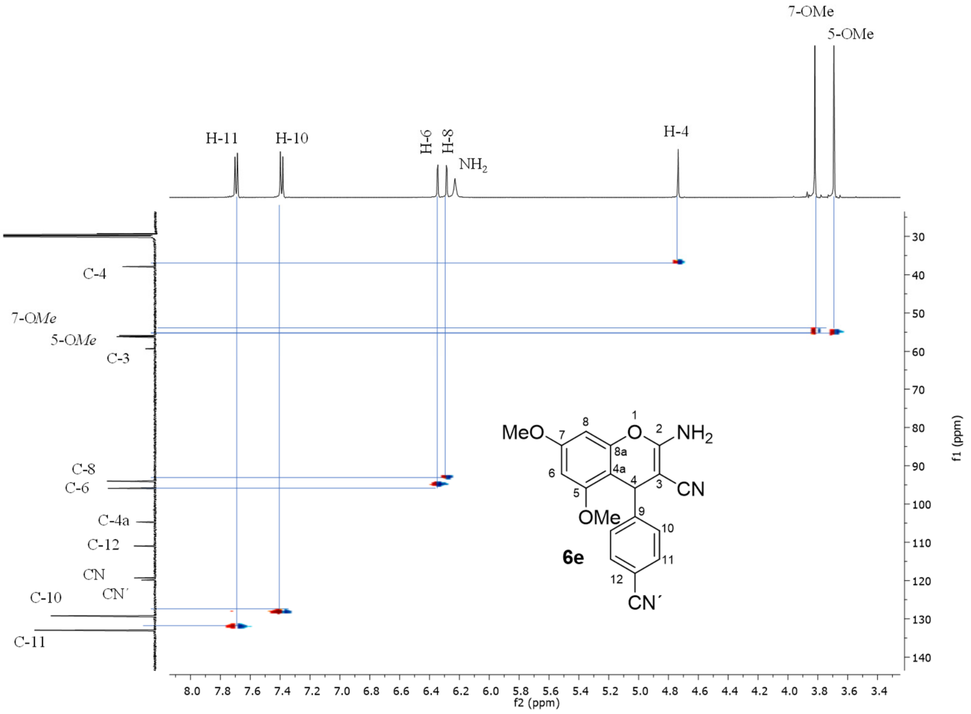

Figure S105. HMQC experiment of 2-Amino-3-cyano-4-(4-cyanophenyl)-5,7-dimethoxy-4*H*-chromene **6e**.

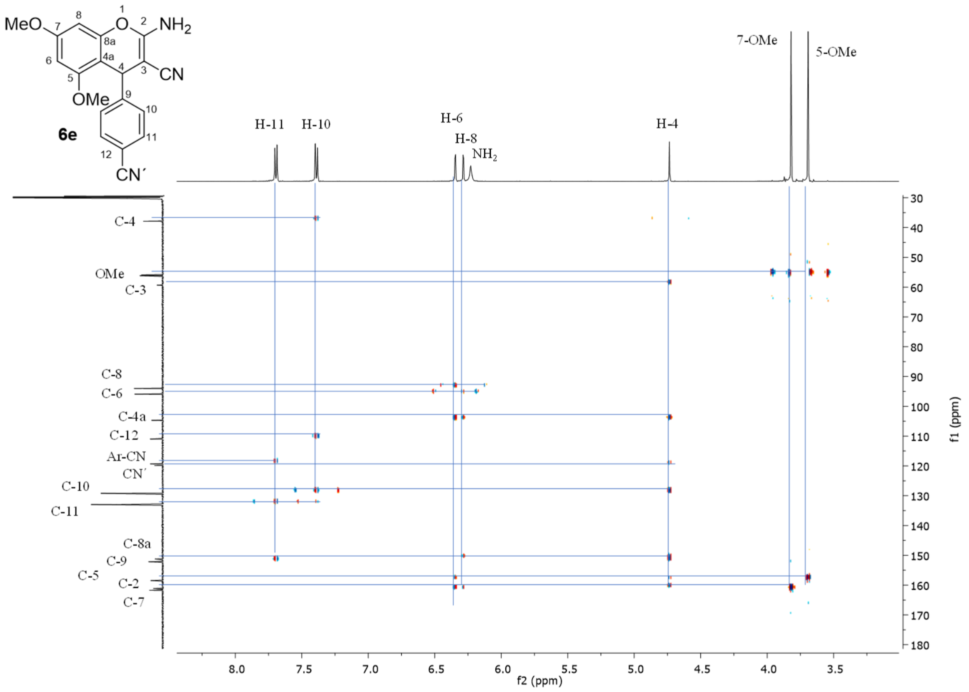

Figure S106. HMBC experiment of 2-Amino-3-cyano-4-(4-cyanophenyl)-5,7-dimethoxy-4*H*-chromene **6e**.

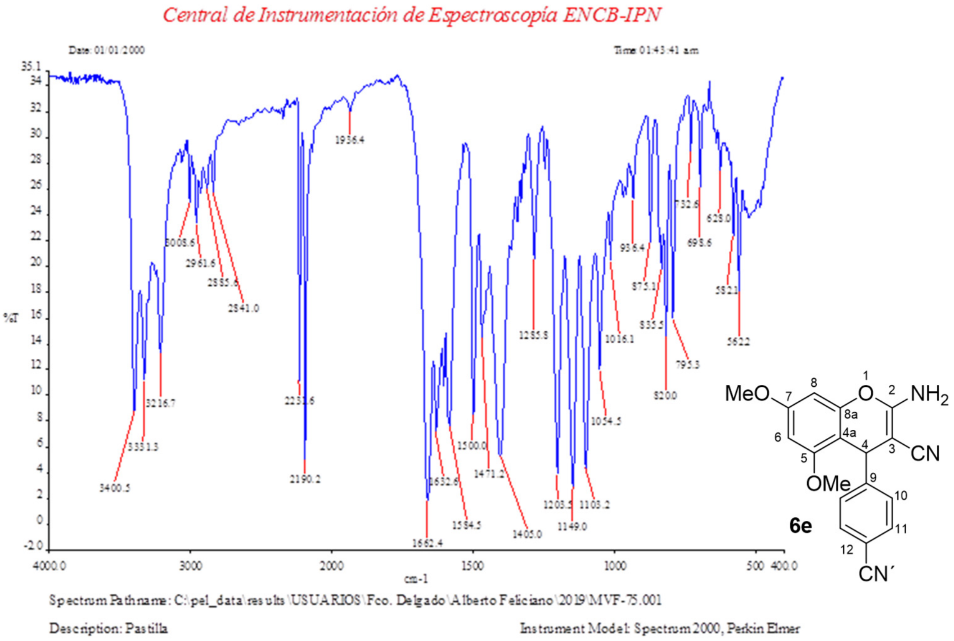

Figure S107. FT-IR of 2-Amino-3-cyano-4-(4-cyanophenyl)-5,7-dimethoxy-4*H*-chromene **6e**.

File: FDR-MVF-75 **Date Run: 02-20-2019 (Time Run: 16:49:56)**
Sample: FDR-MVF-75
Instrument: JEOL GCmate
Inlet: Direct Probe **Ionization mode: EI+**

Scan: 636 **R.T.: 8.62**
Base: m/z 331; 2.6%FS TIC: 207792 **#Ions: 175**

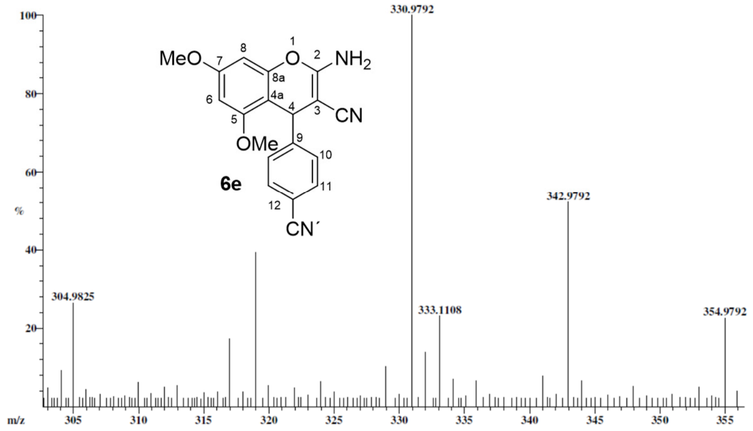

Selected Isotopes : $H_{0-15}C_{0-19}N_{0-3}O_{0-3}$ Error Limit : 5 ppm

Measured Mass	% Base	Formula	Calculated Mass	Error
333.1108	23.1%	$C_{19}H_{15}N_3O_3$	333.1113	-1.6

Figure S108. HRMS (EI) $[M]^+$ of 2-Amino-3-cyano-4-(4-cyanophenyl)-5,7-dimethoxy-4*H*-chromene **6e**.

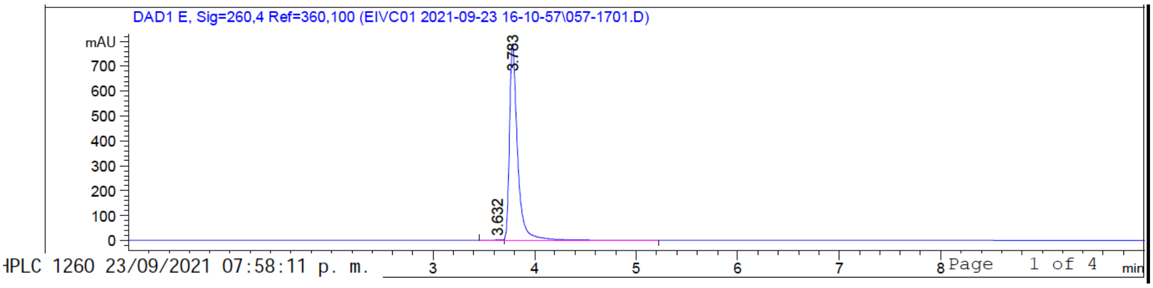

Signal 5: DAD1 E, Sig=260,4 Ref=360,100

Peak #	RetTime [min]	Type	Width [min]	Area [mAU*s]	Height [mAU]	Area %
1	3.632	BV E	0.0793	9.40926	1.68142	0.2123
2	3.783	VB R	0.0822	4421.86133	791.16357	99.7877
Totals :				4431.27059	792.84500	

Figure S109. **HPLC** of 2-Amino-3-cyano-4-(4-cyanophenyl)-5,7-dimethoxy-4*H*-chromene **6e**. 0.1 mg/ml, MeCN, 0.4 ml/min, 260 nm, purity = 99.78%.

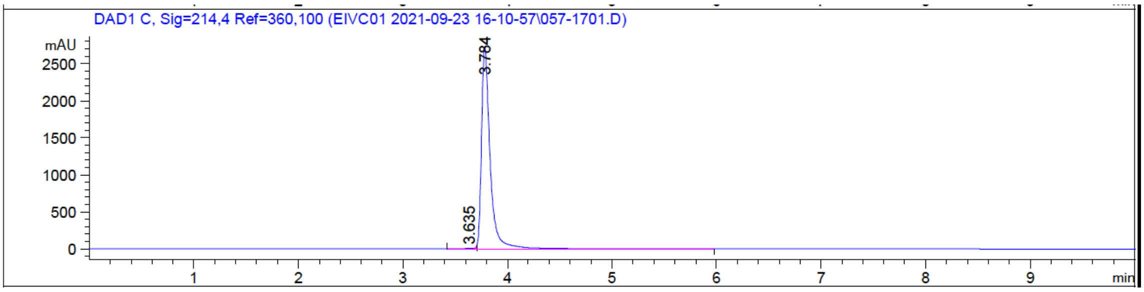

Signal 3: DAD1 C, Sig=214,4 Ref=360,100

Peak #	RetTime [min]	Type	Width [min]	Area [mAU*s]	Height [mAU]	Area %
1	3.635	BV E	0.0768	53.91659	10.17904	0.3404
2	3.784	VB R	0.0853	1.57837e4	2735.40405	99.6596
Totals :				1.58376e4	2745.58310	

Figure S110. **HPLC** of 2-Amino-3-cyano-4-(4-cyanophenyl)-5,7-dimethoxy-4*H*-chromene **6e**. 0.1 mg/ml, MeCN, 0.4 ml/min, 214 nm, purity = 99.65%.

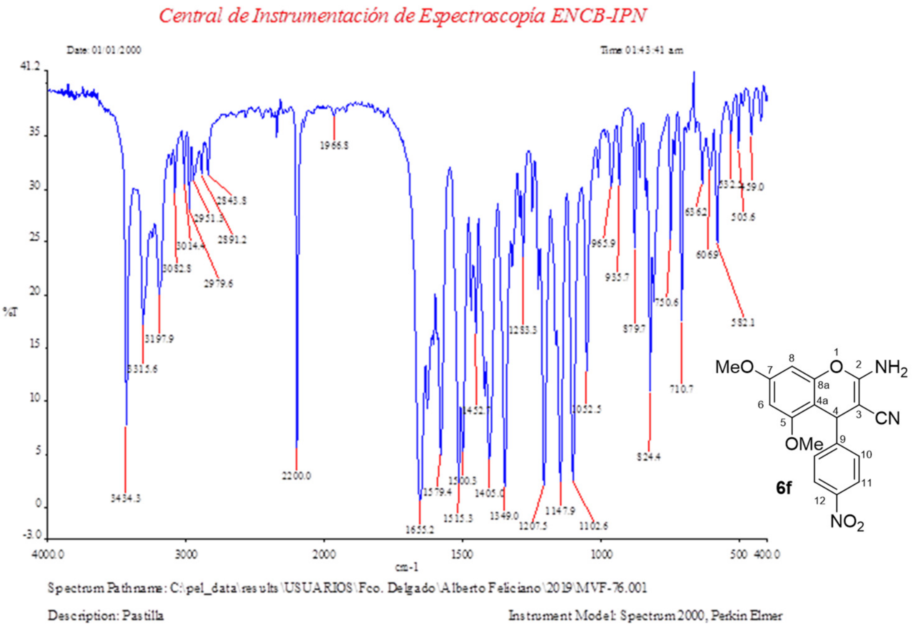

Figure S111. FT-IR of 2-Amino-3-cyano-5,7-dimethoxy-4-(4-nitrophenyl)-4H-chromene **6f**.

File: FDR-MVF-76 Date Run: 02-26-2019 (Time Run: 13:38:11)
Sample: FDR-MVF-76
Instrument: JEOL GCmate
Inlet: Direct Probe Ionization mode: EI+

Scan: 466 R.T.: 6.22
Base: m/z 353; 8.1%FS TIC: 357104 #Ions: 178

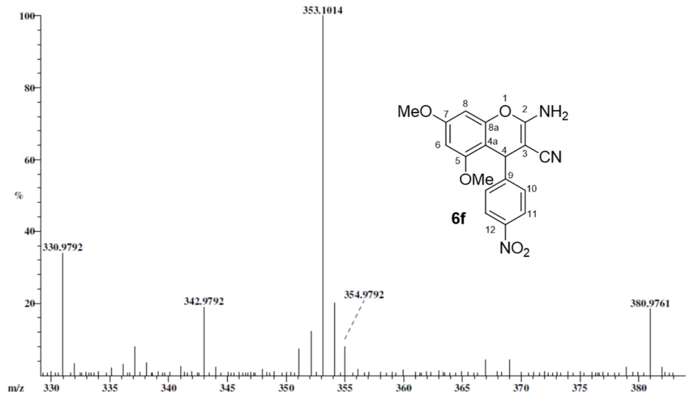

Selected Isotopes : $H_{0-15}C_{0-18}N_{0-3}O_{0-5}$ Error Limit : 5 ppm

Measured Mass	% Base	Formula	Calculated Mass	Error
353.1014	100.0%	$C_{18}H_{15}N_3O_5$	353.1012	0.7

Figure S112. HRMS (EI) $[M]^+$ of 2-Amino-3-cyano-5,7-dimethoxy-4-(4-nitrophenyl)-4H-chromene **6f**.

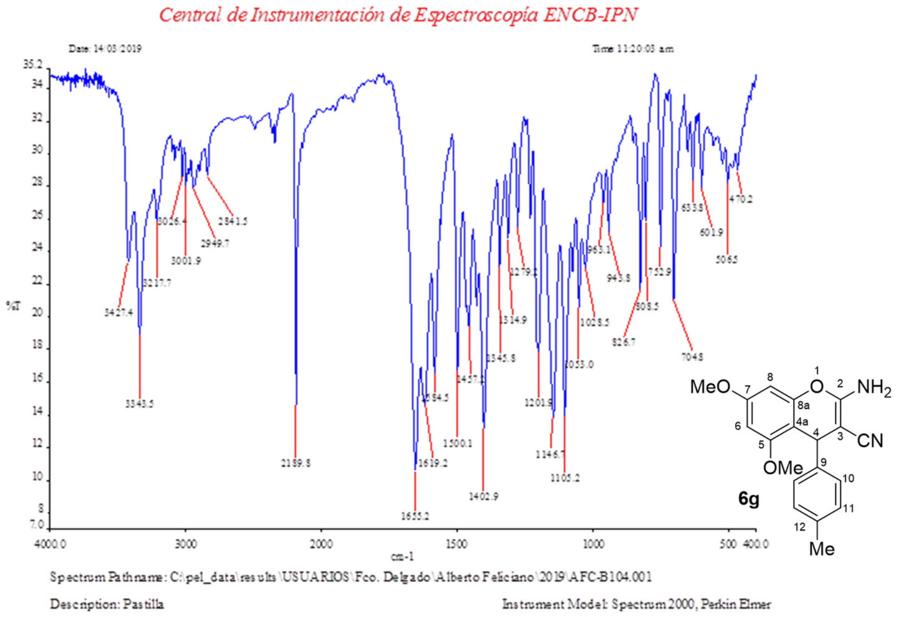

Figure S113. FT-IR of 2-Amino-3-cyano-5,7-dimethoxy-4-(*p*-tolyl)-4*H*-chromene **6g**.

File: FDR-AFC-B108 **Date Run: 02-13-2019 (Time Run: 16:47:10)**
Sample: FDR-AFC-B108
Instrument: JEOL GCmate
Inlet: Direct Probe **Ionization mode: EI+**

Scan: 272 **R.T.: 3.61**
Base: m/z 322; 2.2%FS TIC: 230384 **#Ions: 166**

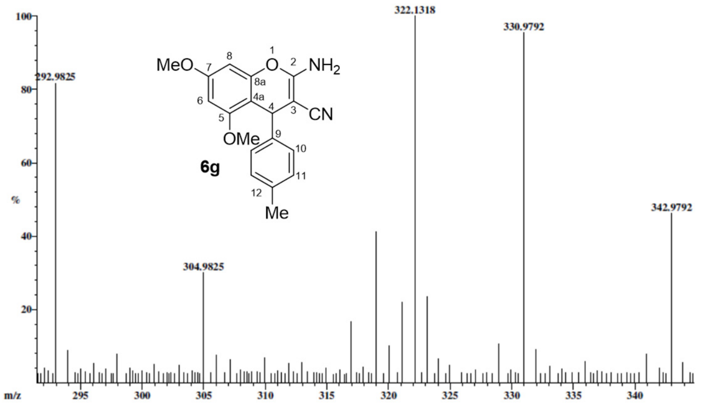

Selected Isotopes : $H_{0-18}C_{0-19}N_{0-2}O_{0-3}$ **Error Limit : 5 ppm**

Measured Mass	% Base	Formula	Calculated Mass	Error
322.1318	100.0%	$C_{19}H_{18}N_2O_3$	322.1318	0.2

Figure S114. HRMS (EI) $[M]^+$ of 2-Amino-3-cyano-5,7-dimethoxy-4-(*p*-tolyl)-4*H*-chromene **6g**.

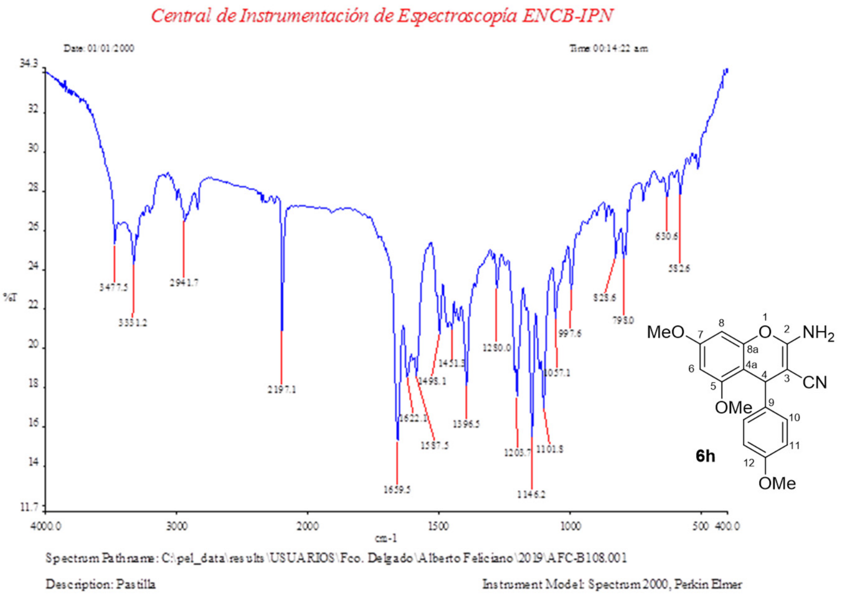

Figure S115. FT-IR of 2-Amino-3-cyano-5,7-dimethoxy-4-(4-methoxyphenyl)-4*H*-chromene **6h**.

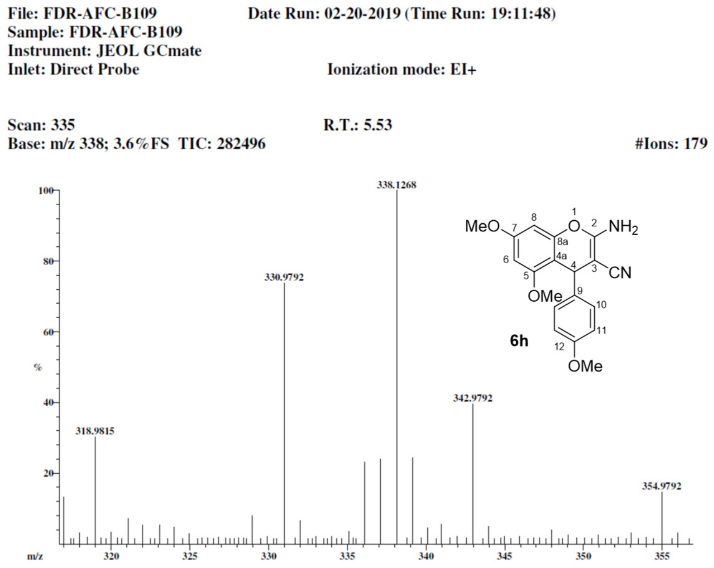

Selected Isotopes : $H_{0-18}C_{0-19}N_{0-2}O_{0-4}$ Error Limit : 5 ppm

Measured Mass	% Base	Formula	Calculated Mass	Error
338.1268	100.0%	$C_{19}H_{18}N_2O_4$	338.1267	0.4

Figure S116. HRMS (EI) $[M]^+$ of 2-Amino-3-cyano-5,7-dimethoxy-4-(4-methoxyphenyl)-4*H*-chromene **6h**.

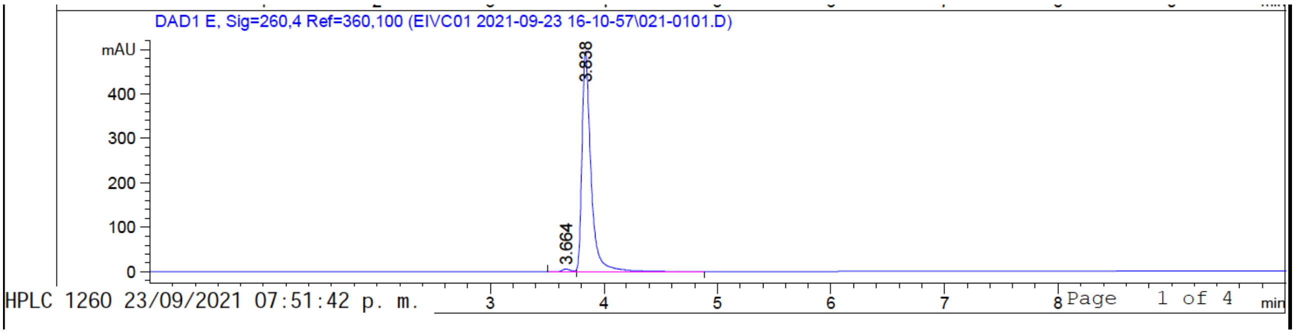

Signal 5: DAD1 E, Sig=260,4 Ref=360,100

Peak #	RetTime [min]	Type	Width [min]	Area [mAU*s]	Height [mAU]	Area %
1	3.664	BV E	0.0742	30.79066	6.17711	1.1066
2	3.838	VB R	0.0817	2751.67749	496.36740	98.8934
Totals :				2782.46815	502.54451	

Figure S117. **HPLC** of 2-Amino-3-cyano-5,7-dimethoxy-4-(4-methoxyphenyl)-4*H*-chromene **6h**. 0.1 mg/ml, MeCN, 0.4 ml/min, 260 nm, purity = 98.89%.

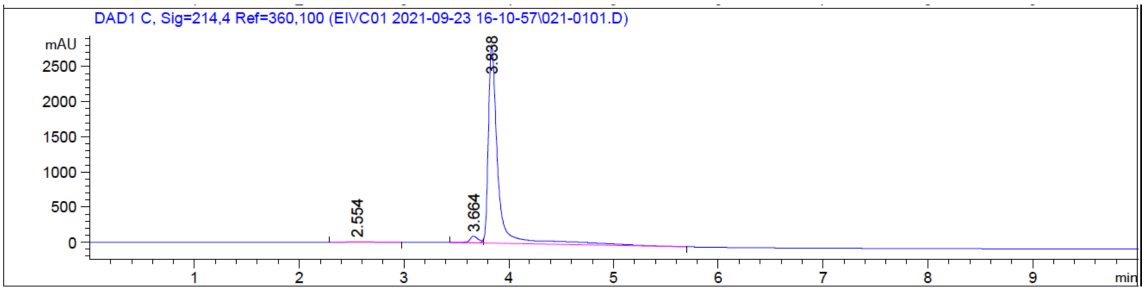

Signal 3: DAD1 C, Sig=214,4 Ref=360,100

Peak #	RetTime [min]	Type	Width [min]	Area [mAU*s]	Height [mAU]	Area %
1	2.554	BB	0.2128	45.70712	3.21559	0.2420
2	3.664	BV E	0.0801	504.85776	93.33439	2.6733
3	3.838	VB R	0.0942	1.83348e4	2805.51050	97.0847
Totals :				1.88854e4	2902.06047	

Figure S118. **HPLC** of 2-Amino-3-cyano-5,7-dimethoxy-4-(4-methoxyphenyl)-4*H*-chromene **6h**. 0.1 mg/ml, MeCN, 0.4 ml/min, 214 nm, purity = 97.08%.

```
CYP51CK       -MSVIKAIAADVQRYALLAYSHFQTFSLLQQTLLVISIPFLYSALWQLLYSFRKDRVPMV  59
CYP51CKE      -MSTSESFVGKLFEVLQGLLLQFWALTITQRVSIVILLPFVYNIVWQLLYSMRKDRVPLV  59
CYP51CG       MSTENTSLVVELLEYVKLGLSYFQALPLAQRVSIMVALPFVYTITWQLLYSLRKDRPPLV  60
CYP51SC       -MSATKSIVGEALEYVNIGLSHFLALPLAQRISLIIIIPFIYNIVWQLLYSLRKDRPPLV  59
CYP51CP       ---------MALVDLALHGYNYFMTLSTLQQFGLLVFAPFIYNIIWQLLYSLRKDRVPLV  51
CYP51CA       ---------MAIVETVIDGINYFLSLSVTQQISILLGVPFVYNLVWQYLYSLRKDRAPLV  51
CYP51CD       ---------MAIVETAIDGINYFLSLSVTQQITILLGVPFVYNLIWQYLYSLRKDRAPLV  51
                                    * ::   *:  :::  **:*.  ** ***:**** *:*

CYP51CK       HYWIPWVGSAVVYGMQPYEFFENCRKQHGDVFSFLLLGKVMTVYLGPKGHEFVLNAKLSD  119
CYP51CKE      FYWIPWVGSAVTYGMRPYEFFEECRQKYGDVFSFVLLGRVMTVYLGPKGHEFVLNARLAD  119
CYP51CG       FYWIPWVGSAIPYGTKPYEFFEDCQKKYGDIFSFMLLGRIMTVYLGPKGHEFIFNAKLAD  120
CYP51SC       FYWIPWVGSAVVYGMKPYEFFEECQKKYGDIFSFVLLGRVMTVYLGPKGHEFVFNAKLAD  119
CYP51CP       FYWIPWVGSAVSYGQDPYGFFEQCREKYGDLFSFVMLGRVMTVYLGPKGHEFVFNAKLSD  111
CYP51CA       FYWIPWFGSAASYGQQPYEFFESCRQKYGDVFSFMLLGKIMTVYLGPKGHEFVFNAKLSD  111
CYP51CD       FYWIPWFGSAASFGQKPYEFFESCRQKHGDIFSFMLLGKIMTVYLGPKGHEFIFNAKLSD  111
              .*****.***  :*  ** ***.*::::**:***::**::************::**:*:*
                    118
CYP51CK       VSAEDAYTHLTTPVFGKGVIYDCPNWKLMEQKKFAKVALTKESFIRYVPLIKDEMLKYFN  179
CYP51CKE      VSAEAAYTHLTTPVFGEGVIYDCSNSRLMDQKKFVKGALTKDAFRKYVPLVTEEVQKYFK  179
CYP51CG       VSAEAAYSHLTTPVFGKGVIYDCPNHRLMEQKKFVKGALTKEAFVRYVPLIAEEIYKYFR  180
CYP51SC       VSAEAAYAHLTTPVFGKGVIYDCPNSRLMEQKKFVKGALTKEAFKSYVPLIAEEVYKYFR  179
CYP51CP       VSAEDAYQHLTTPVFGKGVIYDCPNARLMEQKKFAKTALTTDSFRRYVPLIRGEILDYFT  171
CYP51CA       VSAEDAYKHLTTPVFGKGVIYDCPNSRLMEQKKFAKFALTTDSFKRYVPKIREEILNYFV  171
CYP51CD       VSAEDAYKHLTTPVFGKGVIYDCPNSRLMEQKKFAKFALTTESFKRYVPKIREEILDYFV  171
              **** ** ********:****** * :**:****.* ***.::*  *** :  *: .**

CYP51CK       ANF-----RGDSGKTDVLKSQSEMTLFTASRSLFGDALRNRLDASYAEMYSDLDKGFTPL  234
CYP51CKE      NSANFKIGEKDHGKINVMVTQPEMTIFTASRTLLGKEMREKLDTGFAYLYSDLDKGFTPL  239
CYP51CG       NSKNFKINENNSGIVDVMVSQPEMTIFTASRSLLGKEMRDKLDTDFAYLYSDLDKGFTPI  240
CYP51SC       DSKNFRLNERTTGTIDVMVTQPEMTIFTASRSLLGKEMRAKLDTDFAYLYSDLDKGFTPI  239
CYP51CP       KSKVFNMKKQKSGVVDVLQSQPEITIFTASRSLLGEAMRKRFDASFAQLYADLDKGFTPI  231
CYP51CA       TDESFKLKEKTHGVANVMKTQPEITIFTASRSLFGDEMRRIFDRSFAQLYSDLDKGFTPI  231
CYP51CD       NDESFKLKEKNHGVANIMKTQPEITIFTASRSLFGDDVRKLFDRSFAQLYSDLDKGFTPI  231
               .      .   *  ::: :* *:*:*****:*:*. :*  :* .:* :*:********:

CYP51CK       NFVFSYLPLPNYWKRDAAHKNISNTYLDLINTKRAGGEIK-NEDLVDALLKNSVYKDGTR  293
CYP51CKE      NFVFSHLPLDNYRKRDHAQRTISATYMSLIKERRANNDIQ-DRDLIDTLMKSSTYKDGTK  298
CYP51CG       NFVFPNLPLEHYRKRDHAQQAISGTYMSLIKERREKNDIQ-NRDLIDELMKNSTYKDGTK  299
CYP51SC       NFVFPNLPLEHYRKRDHAQKAISGTYMSLIKERRKNNDIQ-DRDLIDSLMKNSTYKDGVK  298
CYP51CP       NFVFPHLPLPHYWKRDAAQQKISETYMTEIARRRETGDIDENRDLIDSLLVNSTYKDGVK  291
CYP51CA       NFVFPNLPLPHYWRRDAAQKKISATYMKEIKSRRERGDIDPNRDLIDSLLIHSTYKDGVK  291
CYP51CD       NFVFPNLPLPHYWRRDAAQKKISATYMKEIKSRRERGDIDPSRDLIDSLLTHSTYKDGVK  291
              ****  ***  :* :** *:: ** **:  *  :*  .:*. ..**:* *:  *.****.:
                                 311
CYP51CK       MTDEELAHLMIGVLMGGQHTSSATSAWFLLHLGEKPQLQEEIYREIQSVLGEN--FEREL  351
CYP51CKE      MTDKEIANLLIGVLMGGQHTSAATSAWAILHLAERPDVQQELYEEQMRVLDN---GKKEL  355
CYP51CG       MTDQEIANLLIGVLMGGQHTSAATSAWCLLHLAERPDVQEELYQEQMRVLNN---DTKEL  356
CYP51SC       MTDQEIANLLIGVLMGGQHTSAATSAWILLHLAERPDVQQELYEEQMRVLDG---GKKEL  355
CYP51CP       MTDQEIANLLIGVLMGGQHTSATTSAWFLLHLAEKPQLQDELYQEVLNALSGKGGNLDDL  351
CYP51CA       MTDQEIANLLIGILMGGQHTSASTSAWFLLHLGEKPHLQDVIYQEVVELLKEKGGDLNDL  351
CYP51CD       MTDQEIANLLIGILMGGQHTSASTSAWFLLHLGEKPHLQDAIYQEVVELLKEKGGDLNDL  351
              ***:*:*:*:**:********::**** :***.*:*.:*: :*.*    *        :*

CYP51CK       TYDDLQKLDLVNATIKETLRLHMPLHSIFRKVTRDLPVPNTSYIVPKGHYVLISPGYTML  411
CYP51CKE      TYDLLQEMPFLNQTIKETLRLHHPLHSLFRKVMNDMPVPNTSYVVPKGHYVLVSPGYCHL  415
CYP51CG       TYDDLQNMPLLNQMIKETLRLHHPLHSLFRKVMRDVAIPNTSYVVPRDYHVLVSPGYTHL  416
CYP51SC       TYDLLQEMPLLNQTIKETLRMHHPLHSLFRKVMKDMHVPNTSYVIPAGYHVLVSPGYTHL  415
CYP51CP       SYEDLQQMPLVNNTIKETLRLHMPLHSIFRKVVSPLVVPNTKYIVPRGHHVLVSPGYAHT  411
CYP51CA       TYEDLQKLPSVNNTIKETLRMHMPLHSIFRKVTNPLRIPETNYIVPKGHYVLVSPGYAHT  411
CYP51CD       TYEDLQKLPSVTNTIKETLRMHMPLHSIFRKVKNPLRIPETNYVVPRGHYVLVSPGYAHT  411
```

```
              :*: **::  :.  ******:* ****:****   : :*:*.*::* .::**:****

CYP51CK       SERYFPNASEFQPHRWDEIKSIDGGISLPAEGENAKETVDYGFGKISKGVASPYLPFGGG   471
CYP51CKE      QDRYFPNAKEFNPHRWDNDAASS---Y-----A-SGEQVDYGFGAISKGVSSPYLPFGGG   466
CYP51CG       QEEFFPKPNEFNIHRWDGDAASS---S-----AAGGDEVDYGFGAISKGVSSPYLPFGGG   468
CYP51SC       RDEYFPNAHQFNIHRWNNDSASS---Y------SVGEEVDYGFGAISKGVSSPYLPFGGG   466
CYP51CP       NERFYKDASDFNPHRWDESAS-----------TNDAGEVDYGFGKVSKGVSSSYLPFGGG   460
CYP51CA       SERYFDNPEDFDPTRWDTAAAKANSVS-----FNSSDEVDYGFGKVSKGVSSPYLPFGGG   466
CYP51CD       SERYFDNPNNFDPTRWDTAAAKANSVS-----FNSSDEVDYGFGKISKGVSSPYLPFGGG   466
               :.:: .  :*:  **:   :                 ****** :****:* *******

CYP51CK       RHRCIGEPFAYTQLGTLLVHYIQNFKWTA-----KVPPIDYTSMVTLPTQPAEIKWERRQ   526
CYP51CKE      RHRCVGEHFAYMQLGTLLSNFVRTLTWKYANDKDTVPVPDFQSMVTLPLAPGEIEWTLRK   526
CYP51CG       RHRCIGELFAYCQLGVLMSIFIRTMKWRYPTEGETVPPSDFTSMVTLPTAPAKIYWEKRH   528
CYP51SC       RHRCIGEHFAYCQLGVLMSIFIRTLKWHYP-EGKTVPPPDFTSMVTLPTGPAKIIWEKRN   525
CYP51CP       RHRCIGEQFAYVQLGTILTTFVYNLKWKLA--NGKVPDVDYTSMVTLPQHPAEIVWEKRD   518
CYP51CA       RHRCIGEQFAYVQLGTILTTFVYNLRWTID--GYKVPDPDYSSMVVLPTEPAEIIWEKRE   524
CYP51CD       RHRCIGEQFAYVQLGTILTTFVYNLRWTVE--GNRVPDPDYSSMVVLPTEPARIIWEKRE   524
              ****:** *** ***.::  :: .: *        **  *: ***.**  *..* *  *.

CYP51CK       KN------------      528
CYP51CKE      --------------      526
CYP51CG       PEQKY---------      533
CYP51SC       PEQKIGGRHHHHHH      539
CYP51CP       TCVI----------      522
CYP51CA       TCMF----------      528
CYP51CD       TCMF----------      528
```

Figure S119. Multiple sequence alignment de CYP51 *Candida spp*., with CYP51SC template sequence. Asterisks show identical amino acids and dots similar amino acids. In cyan color shows the CYP51 motif sites, cysteine residue (red), two arginine residues (green) and histidine residue (fuchsia), Tyr118 and Thr311 (yellow).

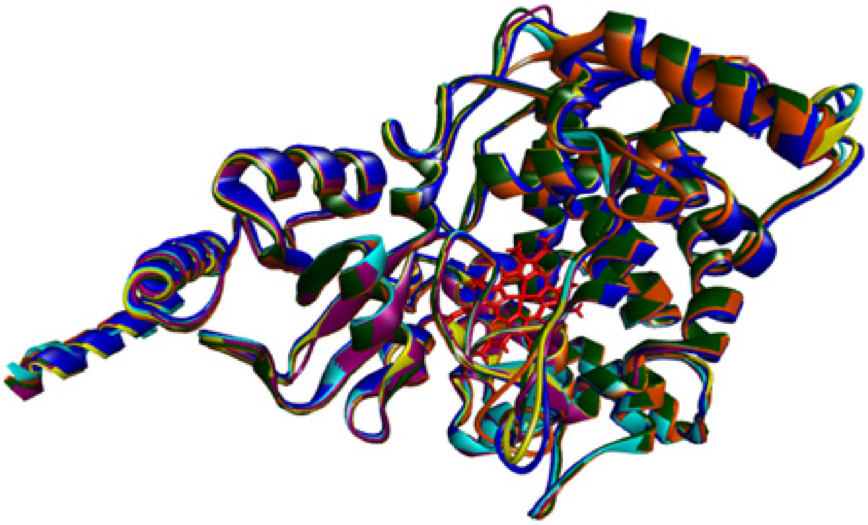

Yeast	Model	% Identity
***S. cerevisiae* (PDB: 4WMZ)**	*C. albicans* (CYP51CA)	65.00
	C. dubliniensis (CYP51CD)	63.76
	C. glabrata (CYP51CG)	83.00
	C. kefyr (CYP51CKE)	78.48
	C. krusei (CYP51CK)	61.50
	C. parapsilosis CYP51CP)	66.80

Figure S120. Overlap can be appreciated between models, portrayed with different colored ribbons. The best model was taken from each CYP51 enzyme pertaining to a particular *Candida* species. The models are depicted as follows: CYP51CA (yellow), CYP51CD (blue), CYP51CG (purple), CYP51CKE (green), CYP51CK (orange) and CYP51CP (cyan). The heme group in each CYP51 is in stick representation (red). The percentage of identity of the CYP51 enzymes with the *S. cerevisiae* template is shown.

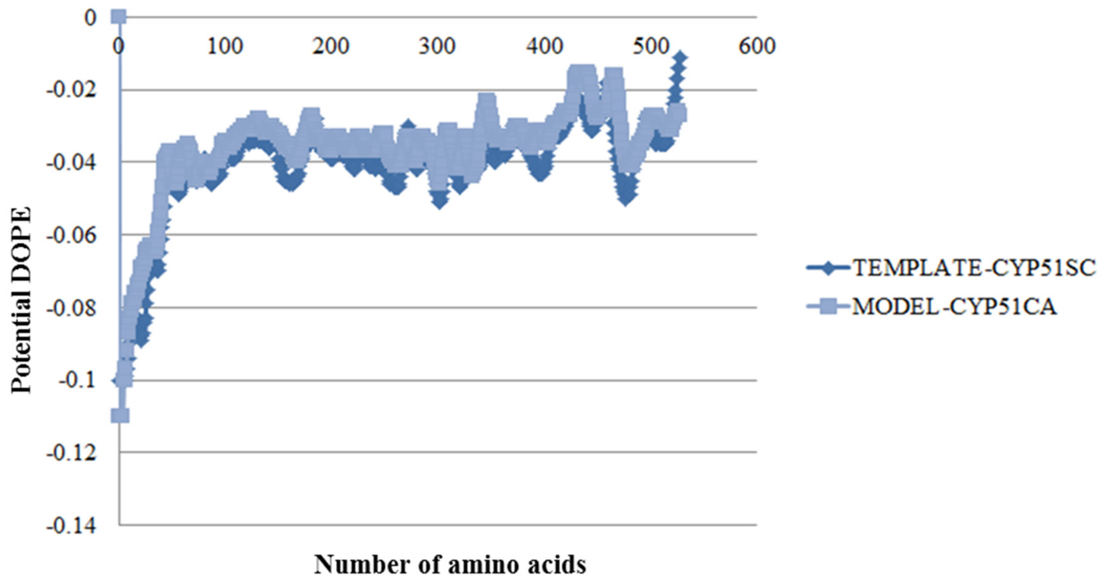

Figure S121. DOPE potential of CYP51CA model with CYP51SC template (pdb: 4wmz).

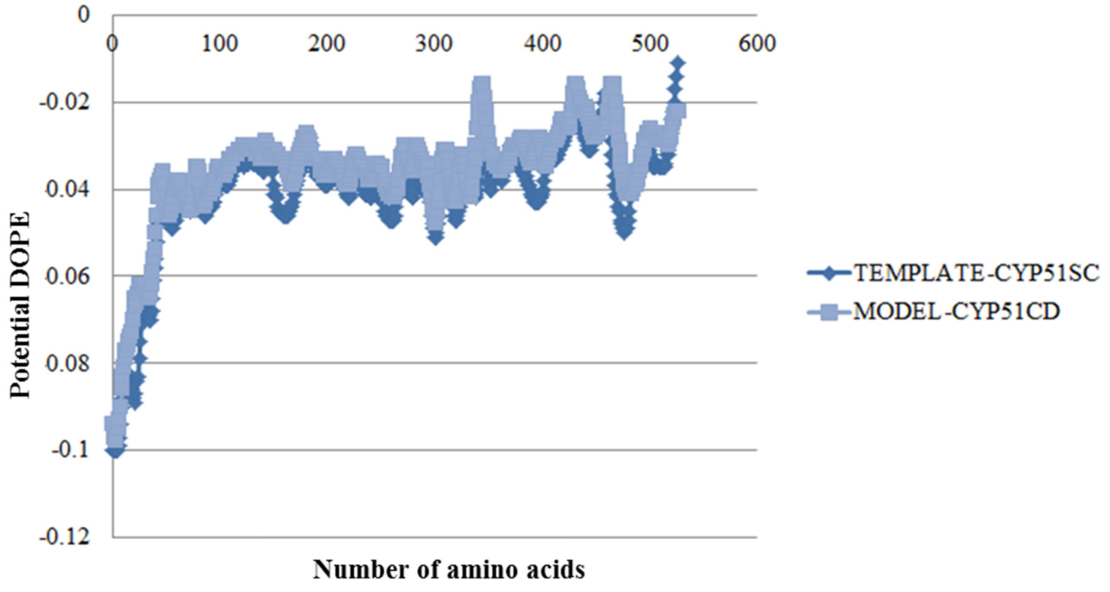

Figure S122. DOPE potential of CYP51CD model with CYP51SC template (pdb: 4wmz).

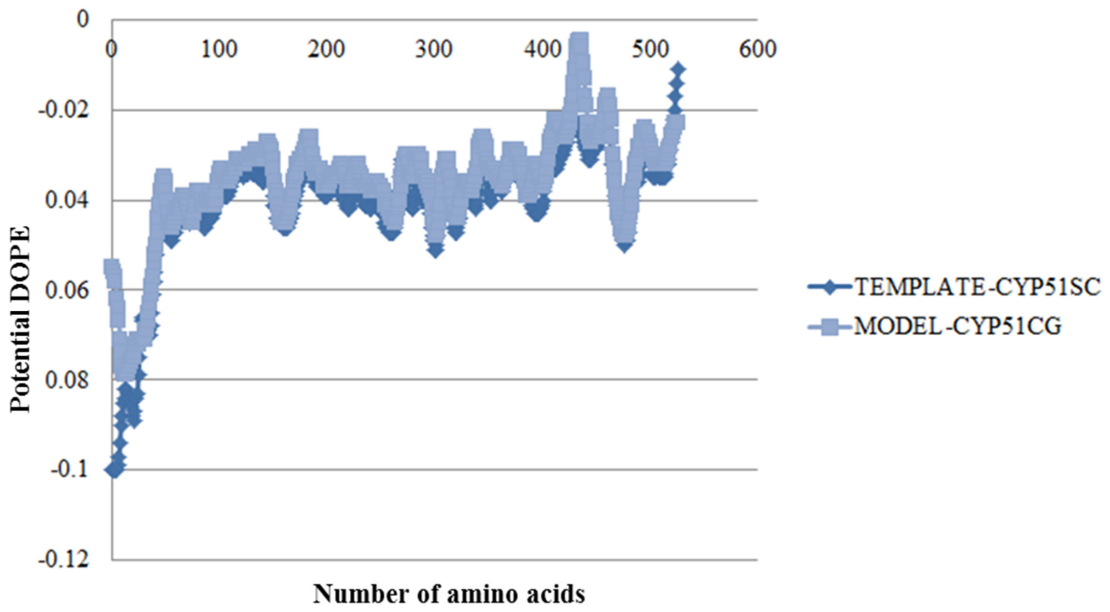

Figure S123. DOPE potential of CYP51CG model with CYP51SC template (pdb: 4wmz).

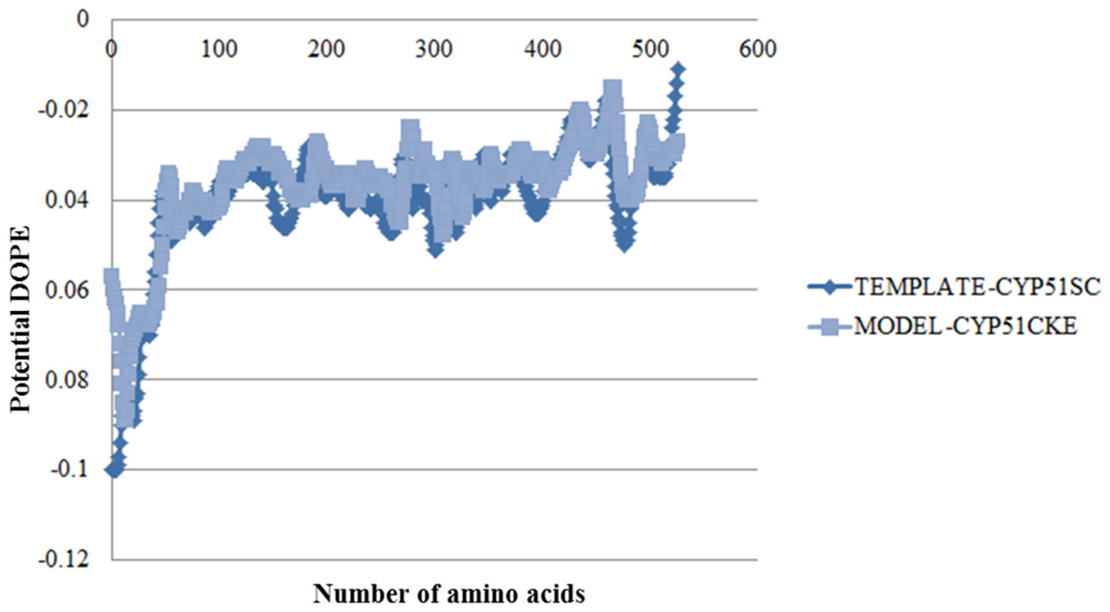

Figure S124. DOPE potential of CYP51CKE model with CYP51SC template (pdb: 4wmz).

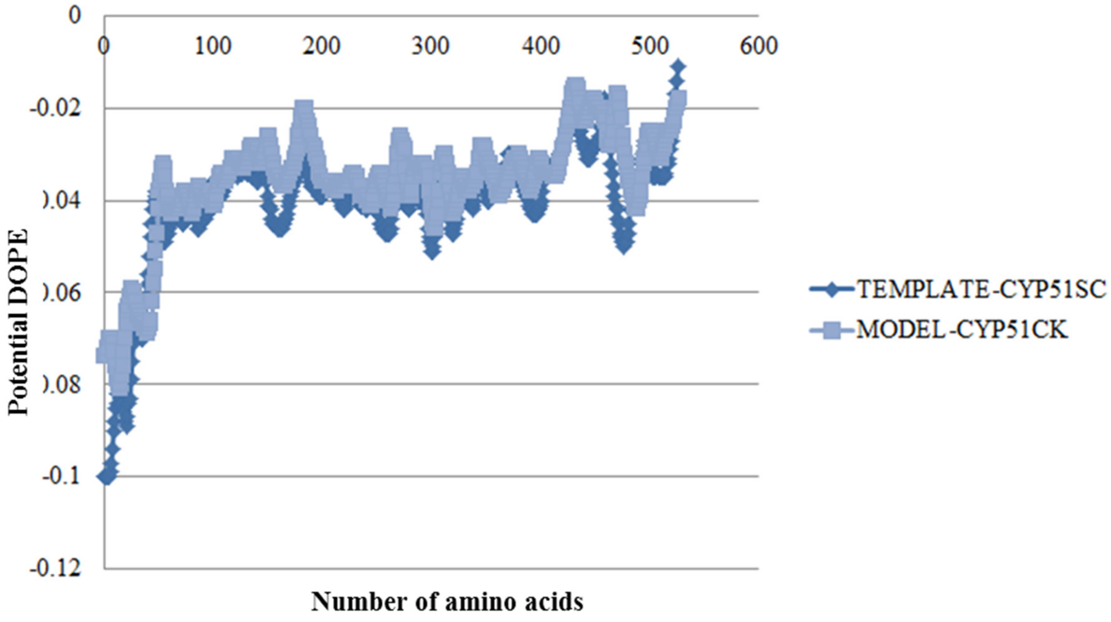

Figure S125. DOPE potential of CYP51CK model with CYP51SC template (pdb: 4wmz).

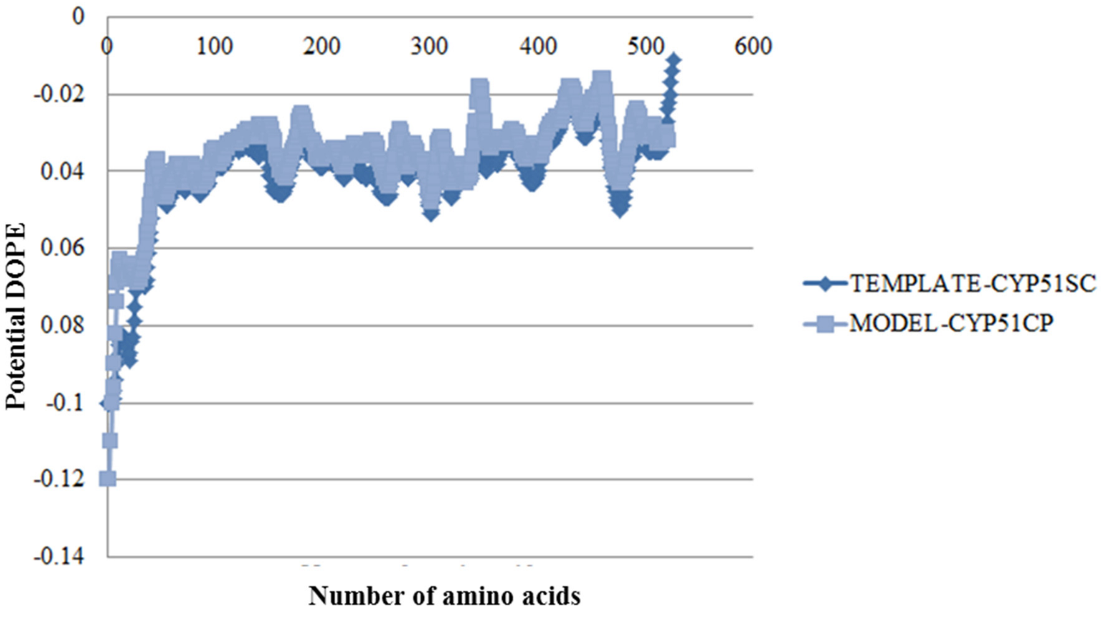

Figure S126. DOPE potential of CYP51CP model with CYP51SC template (pdb: 4wmz).

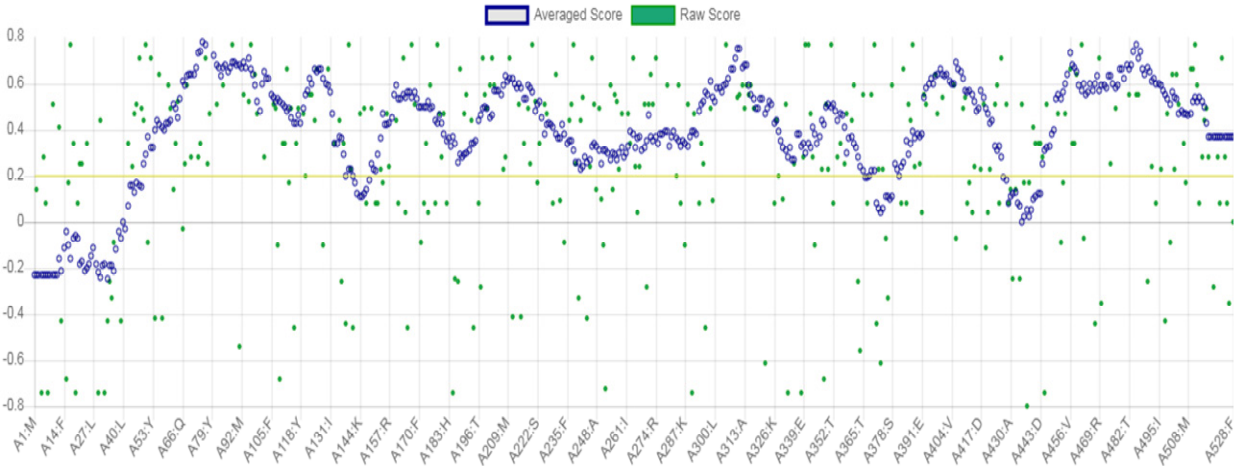

Figure S127. Compatibility chart of an atomic model 3D of CYP51CA with its own amino acid sequence 1D. 84.47% of the residues have averaged 3D-1D score >= 0.2.

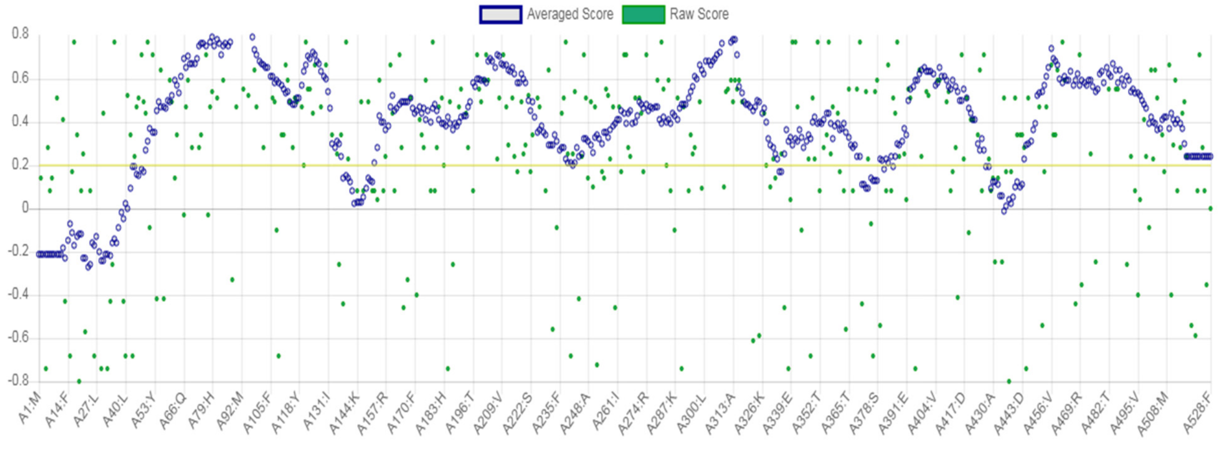

Figure S128. Compatibility chart of an atomic model 3D of CYP51CD with its own amino acid sequence 1D. 82.77% of the residues have averaged 3D-1D score >= 0.2.

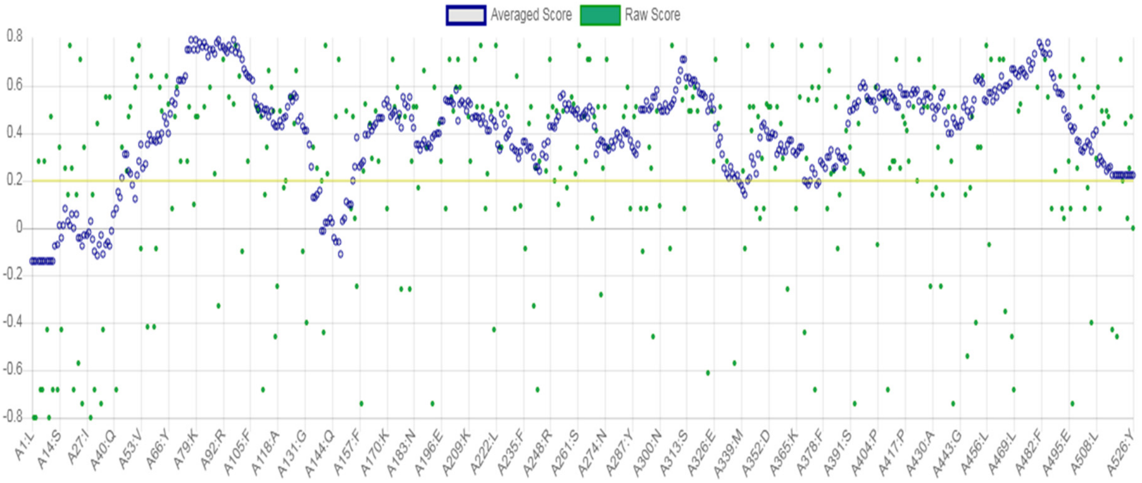

Figure S129. Compatibility chart of an atomic model 3D of CYP51CG with its own amino acid sequence 1D. 86.31 % of the residues have averaged 3D-1D score >= 0.2.

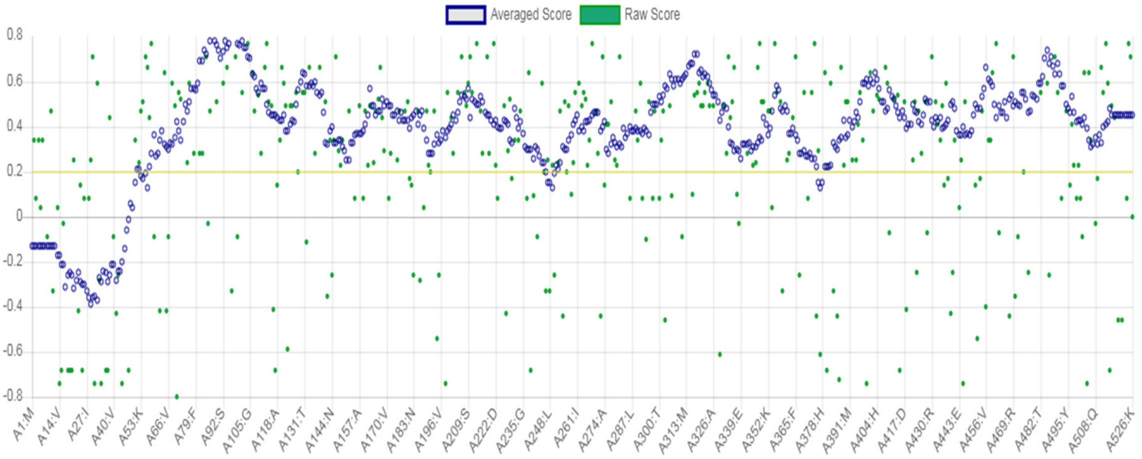

Figure S130. Compatibility chart of an atomic model 3D of CYP51CKE with its own amino acid sequence 1D. 88.40 % of the residues have averaged 3D-1D score >= 0.2.

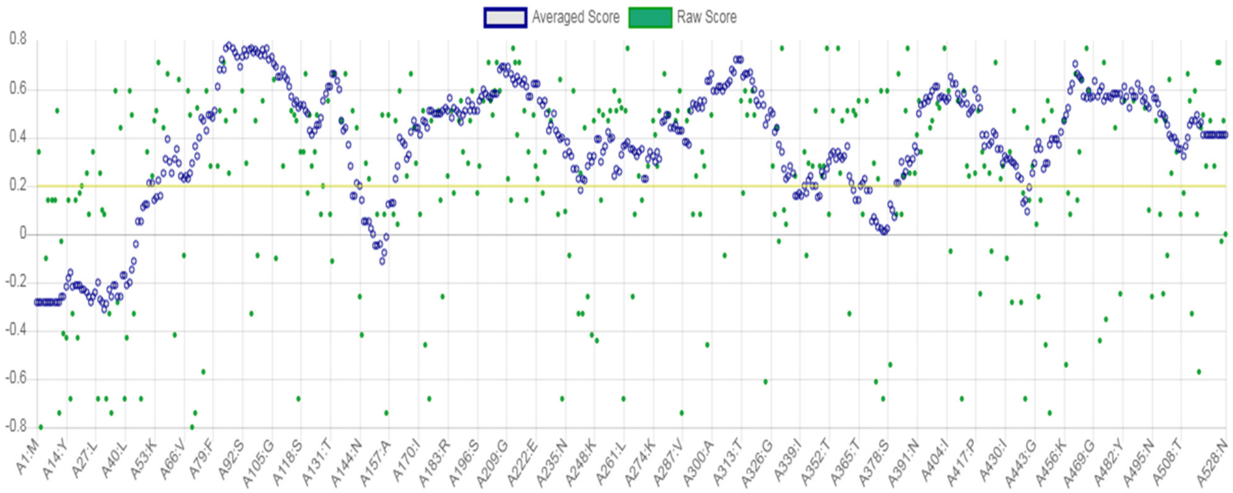

Figure S131. Compatibility chart of an atomic model 3D of CYP51CK with its own amino acid sequence 1D. 81.44 % of the residues have averaged 3D-1D score >= 0.2.

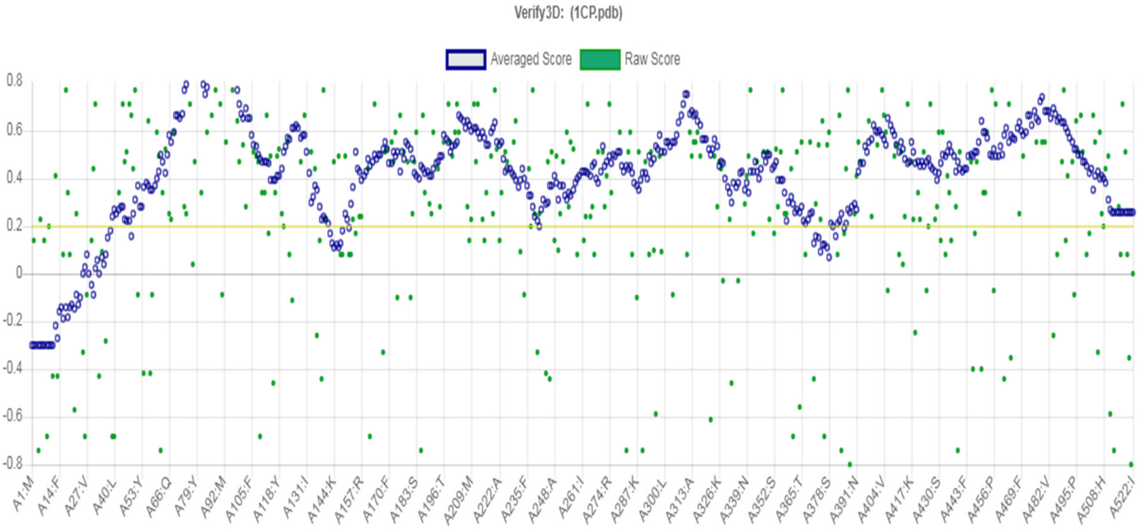

Figure S132. Compatibility chart of an atomic model 3D of CYP51CP with its own amino acid sequence 1D. 89.08 % of the residues have averaged 3D-1D score >= 0.2.

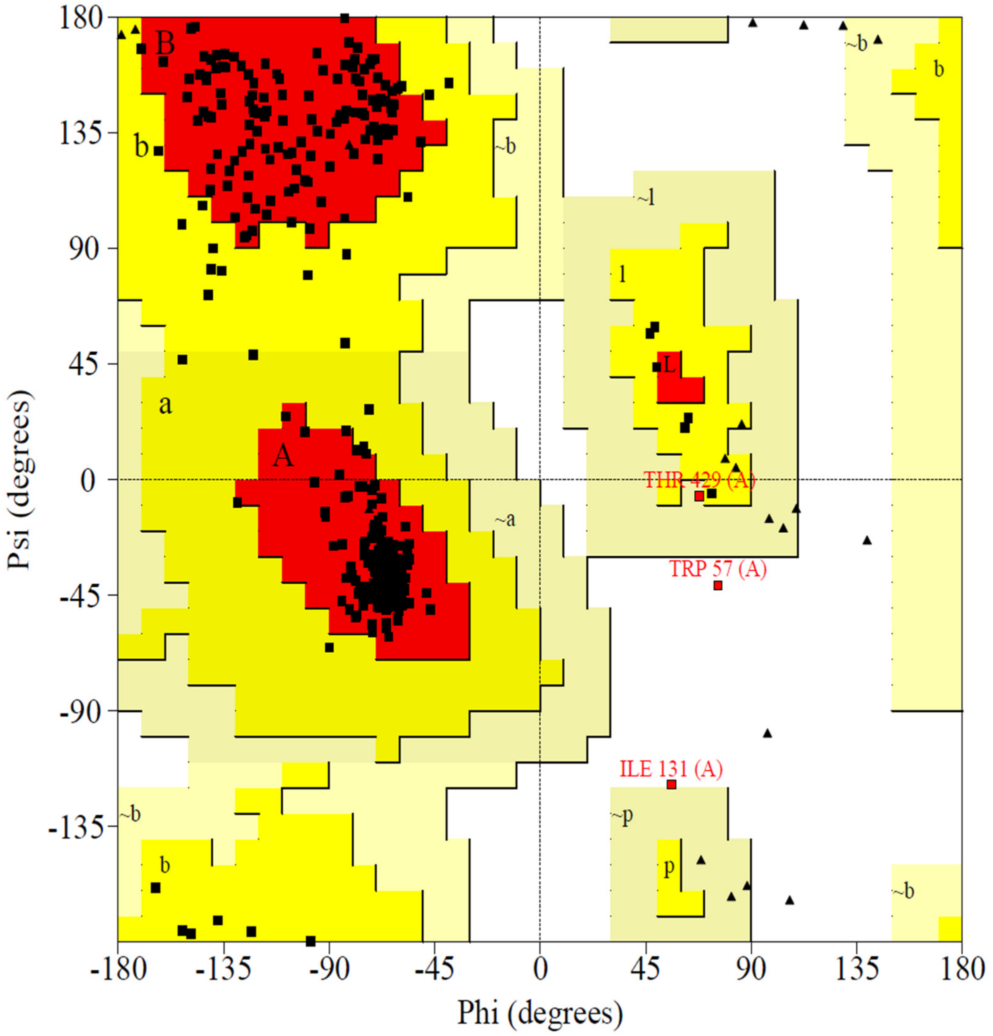

Plot statistics

Residues in most favoured regions [A,B,L]	428	92.0%
Residues in additional allowed regions [a,b,l,p]	34	7.3%
Residues in generously allowed regions [~a,~b,~l,~p]	1	0.2%
Residues in disallowed regions	2	0.4%
	----	------
Number of non-glycine and non-proline residues	465	100.0%
Number of end-residues (excl. Gly and Pro)	3	
Number of glycine residues (shown as triangles)	32	
Number of proline residues	29	

Total number of residues	529	

Figure S133. Plot Ramachandran of CYP51CA model. The favorable residues [A, B, L] remain in the red zone, additional allowed residues [a, b, l, p] remain in the yellow zones, generously permitted residues [~a,~b,~l,~p] remain in beige zone and not allowed regions remain in white zone.

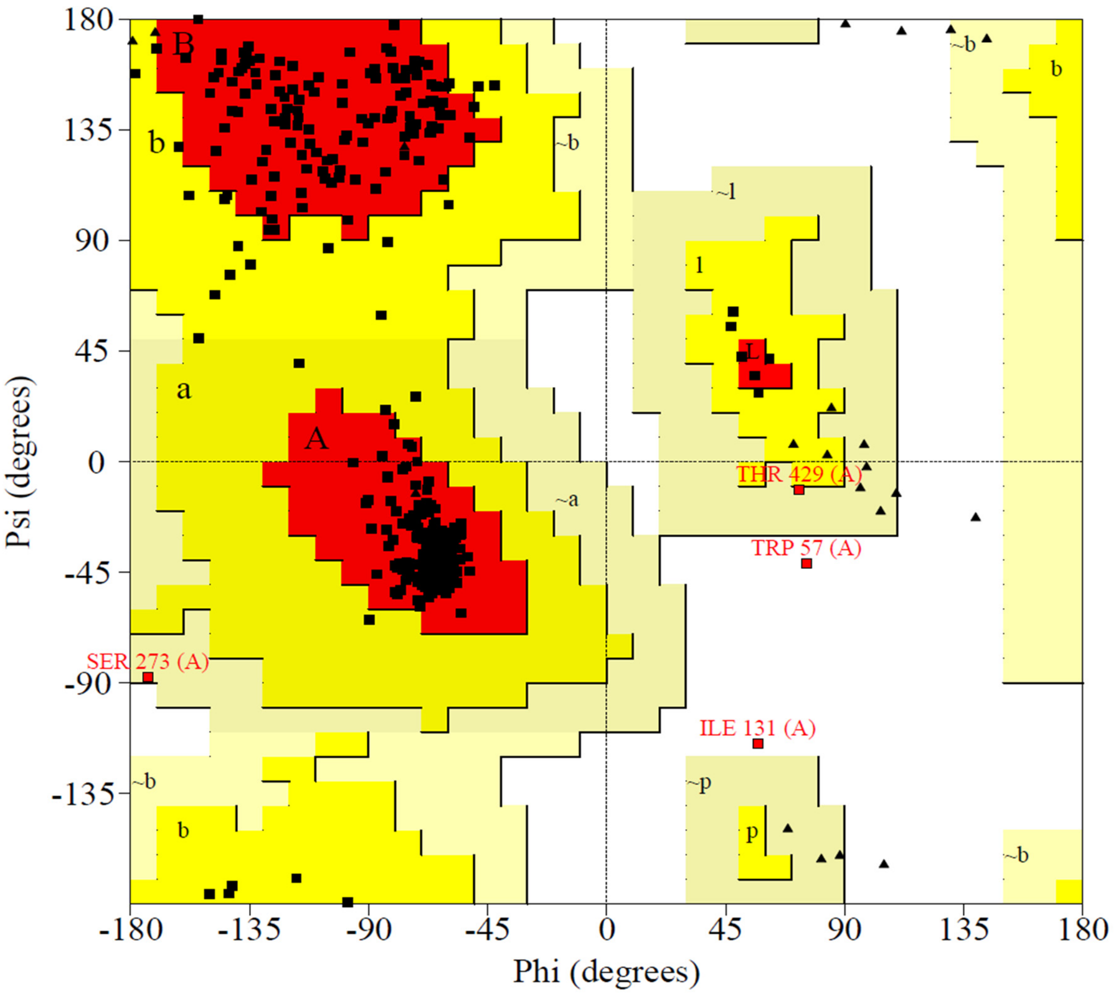

Plot statistics

Residues in most favoured regions [A,B,L]	431	92.7%
Residues in additional allowed regions [a,b,l,p]	30	6.5%
Residues in generously allowed regions [~a,~b,~l,~p]	2	0.4%
Residues in disallowed regions	2	0.4%
	----	------
Number of non-glycine and non-proline residues	465	100.0%
Number of end-residues (excl. Gly and Pro)	3	
Number of glycine residues (shown as triangles)	32	
Number of proline residues	29	

Total number of residues	529	

Figure S134. Plot Ramachandran of CYP51CD model. The favorable residues [A, B, L] remain in the red zone, additional allowed residues [a, b, l, p] remain in the yellow zones, generously permitted residues [~a,~b,~l,~p] remain in beige zone and not allowed regions remain in white zone.

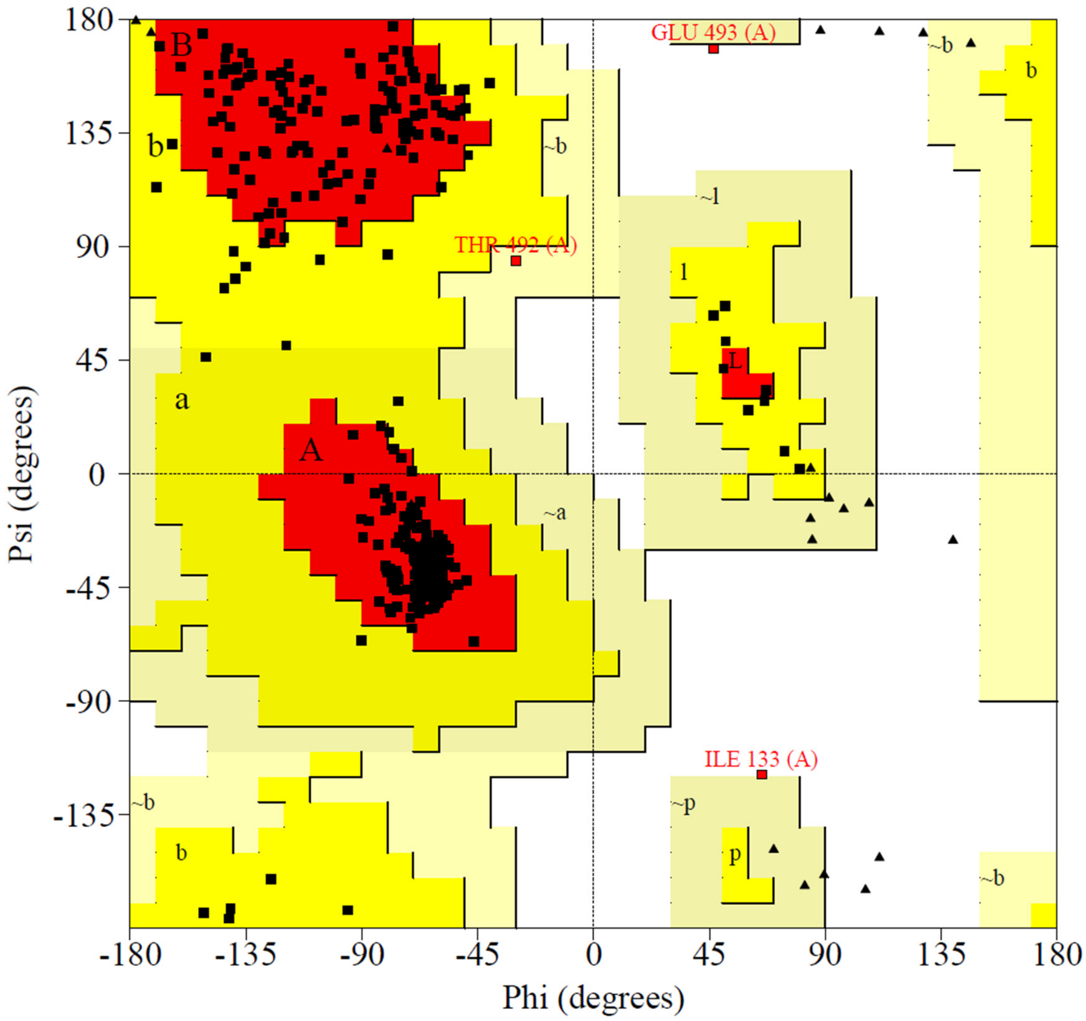

Plot statistics

Residues in most favoured regions [A,B,L]	426	92.2%
Residues in additional allowed regions [a,b,l,p]	33	7.1%
Residues in generously allowed regions [~a,~b,~l,~p]	1	0.2%
Residues in disallowed regions	2	0.4%
	----	------
Number of non-glycine and non-proline residues	462	100.0%
Number of end-residues (excl. Gly and Pro)	3	
Number of glycine residues (shown as triangles)	31	
Number of proline residues	31	

Total number of residues	527	

Figure S135. Plot Ramachandran of CYP51CG model. The favorable residues [A, B, L] remain in the red zone, additional allowed residues [a, b, l, p] remain in the yellow zones, generously permitted residues [~a,~b,~l,~p] remain in beige zone and not allowed regions remain in white zone.

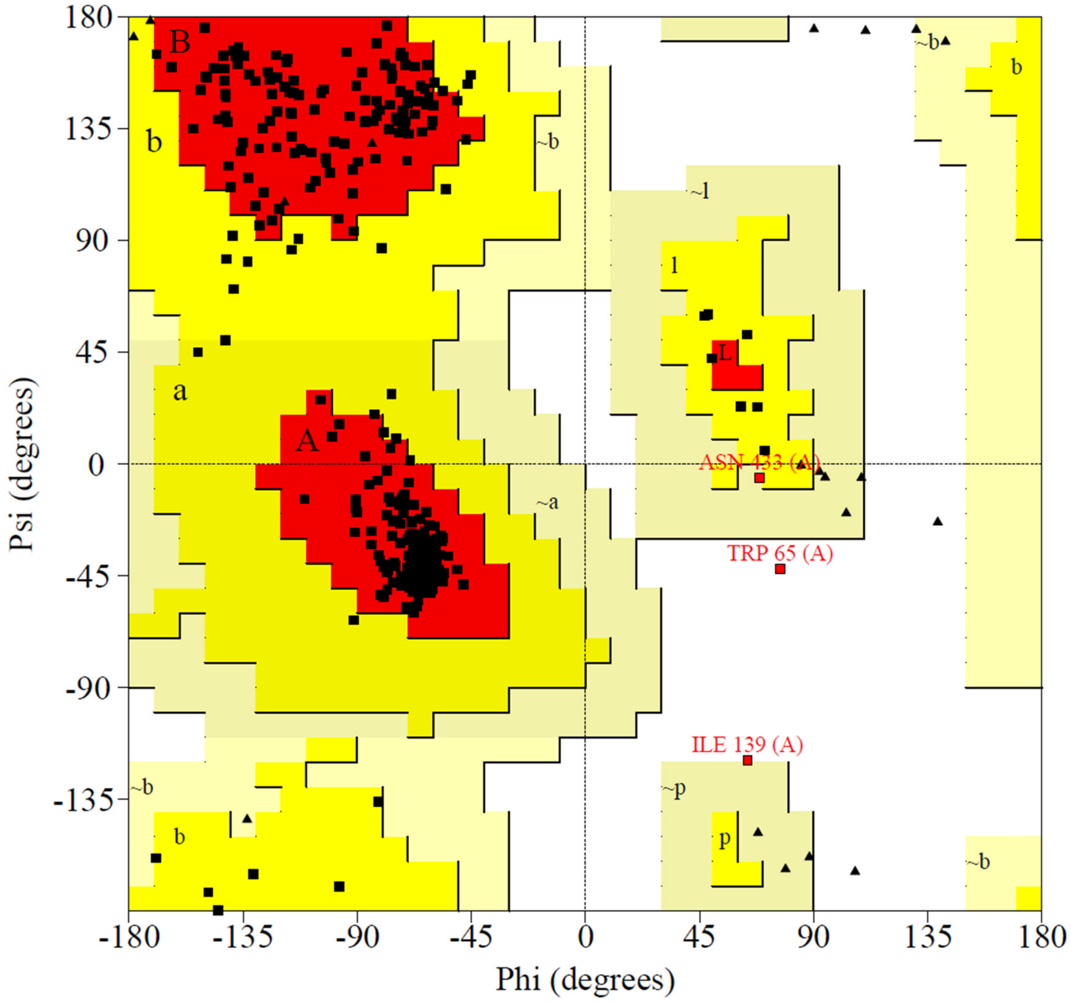

Plot statistics

Residues in most favoured regions [A,B,L]	431	92.5%
Residues in additional allowed regions [a,b,l,p]	32	6.9%
Residues in generously allowed regions [~a,~b,~l,~p]	1	0.2%
Residues in disallowed regions	2	0.4%
	----	------
Number of non-glycine and non-proline residues	466	100.0%
Number of end-residues (excl. Gly and Pro)	3	
Number of glycine residues (shown as triangles)	33	
Number of proline residues	25	

Total number of residues	527	

Figure S136. Plot Ramachandran of CYP51CKE model. The favorable residues [A, B, L] remain in the red zone, additional allowed residues [a, b, l, p] remain in the yellow zones, generously permitted residues [~a,~b,~l,~p] remain in beige zone and not allowed regions remain in white zone.

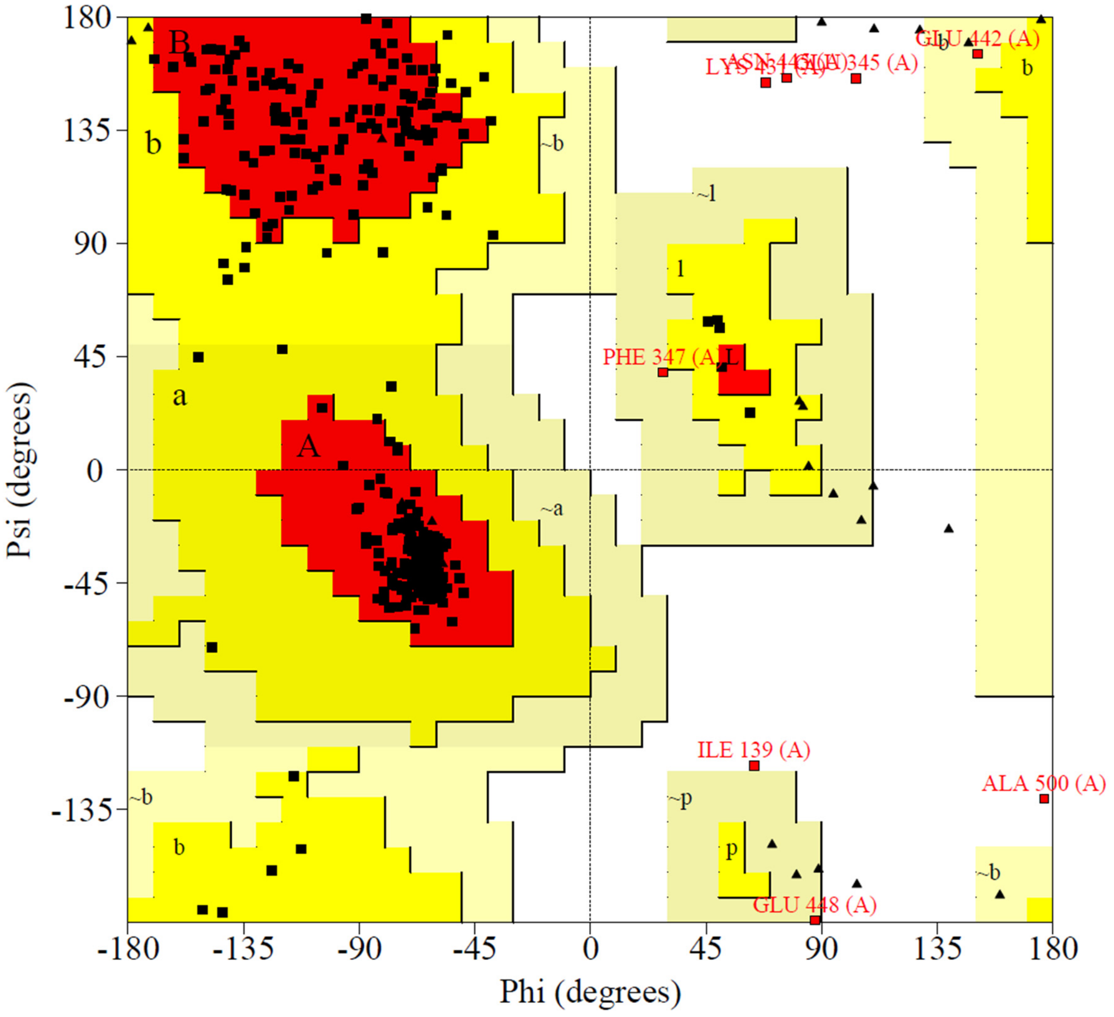

Plot statistics

Residues in most favoured regions [A,B,L]	429	92.1%
Residues in additional allowed regions [a,b,l,p]	29	6.2%
Residues in generously allowed regions [~a,~b,~l,~p]	3	0.6%
Residues in disallowed regions	5	1.1%
	----	------
Number of non-glycine and non-proline residues	466	100.0%
Number of end-residues (excl. Gly and Pro)	3	
Number of glycine residues (shown as triangles)	33	
Number of proline residues	27	

Total number of residues	529	

Figure S137. Plot Ramachandran of CYP51CK model. The favorable residues [A, B, L] remain in the red zone, additional allowed residues [a, b, l, p] remain in the yellow zones, generously permitted residues [~a,~b,~l,~p] remain in beige zone and not allowed regions remain in white zone.

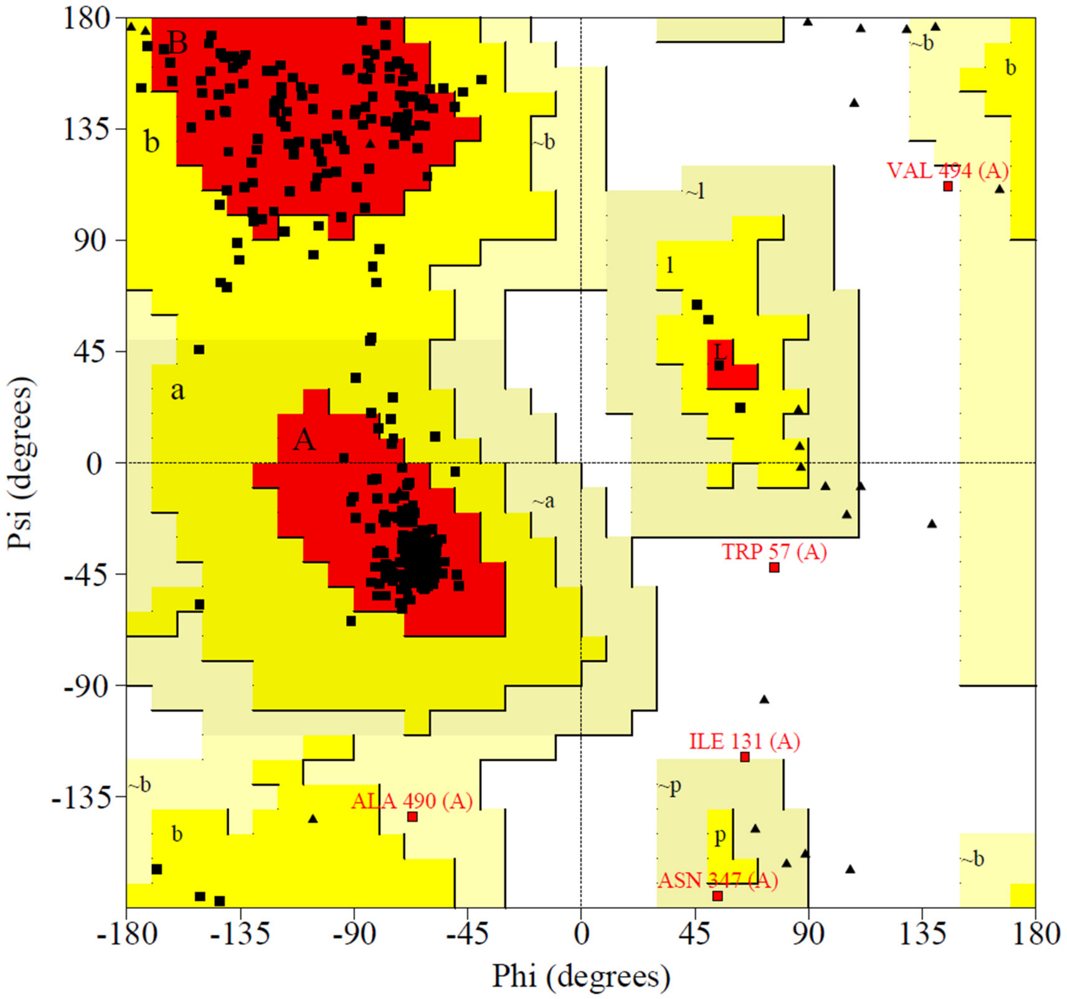

Plot statistics

Residues in most favoured regions [A,B,L]	421	91.5%
Residues in additional allowed regions [a,b,l,p]	34	7.4%
Residues in generously allowed regions [~a,~b,~l,~p]	2	0.4%
Residues in disallowed regions	3	0.7%
	----	------
Number of non-glycine and non-proline residues	460	100.0%
Number of end-residues (excl. Gly and Pro)	3	
Number of glycine residues (shown as triangles)	35	
Number of proline residues	25	

Total number of residues	523	

Figure S138. Plot Ramachandran of CYP51CP model. The favorable residues [A, B, L] remain in the red zone, additional allowed residues [a, b, l, p] remain in the yellow zones, generously permitted residues [~a,~b,~l,~p] remain in beige zone and not allowed regions remain in white zone.

Fluconazole **8**

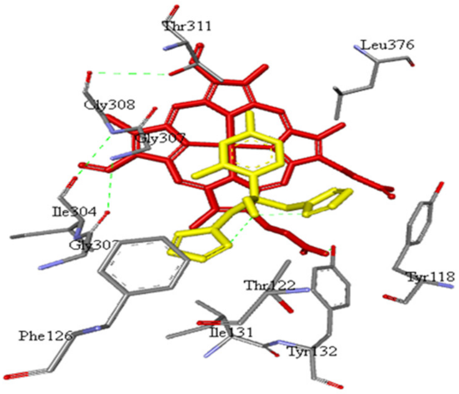

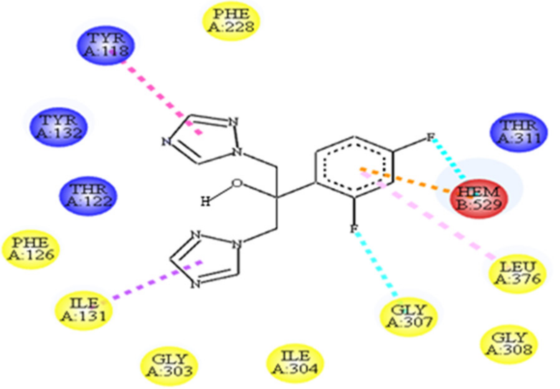

4a

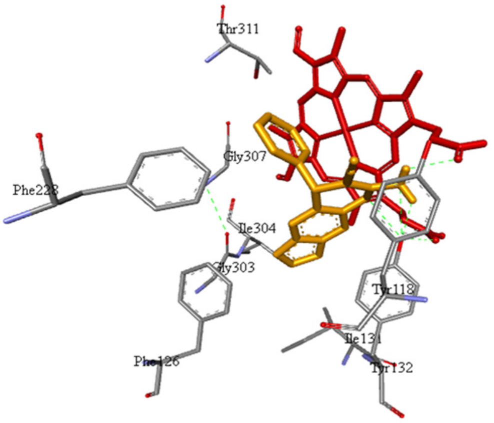

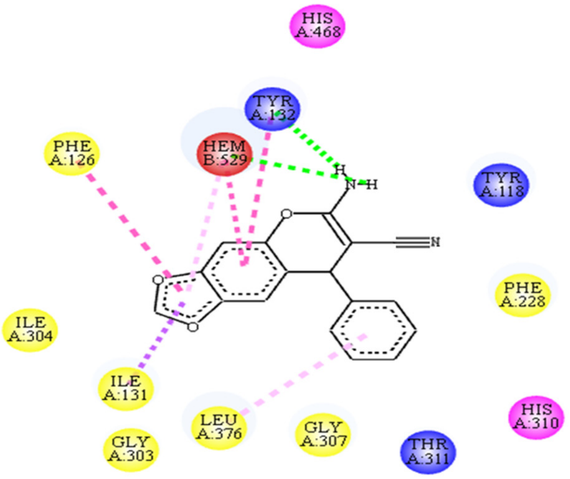

4b

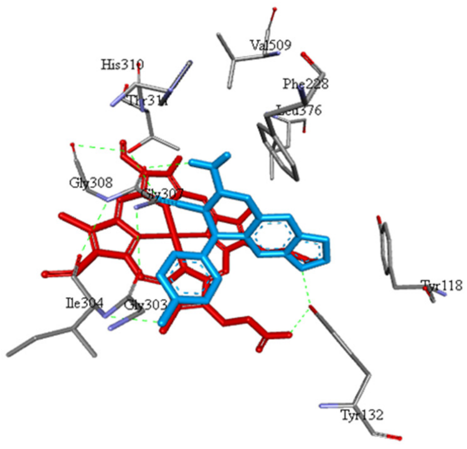

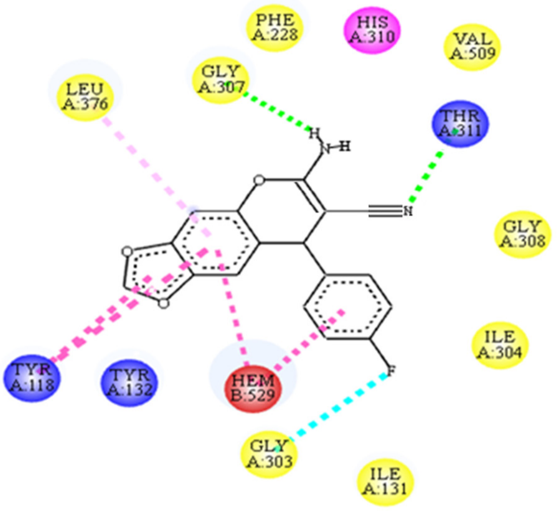

4c

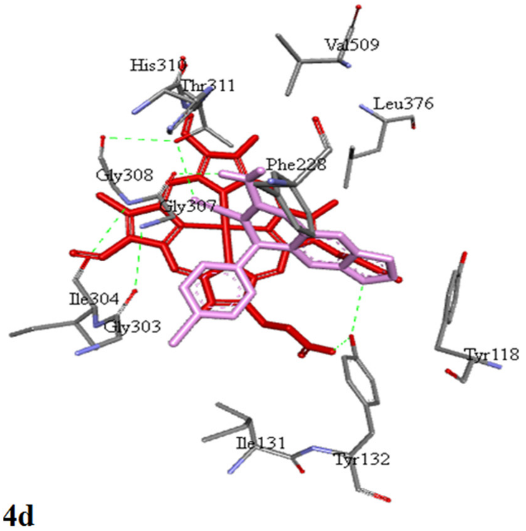

Val509
His310
Thr311
Leu376
Gly308
Phe228
Gly307
Ile304
Gly303
Tyr118
Ile131
Tyr132

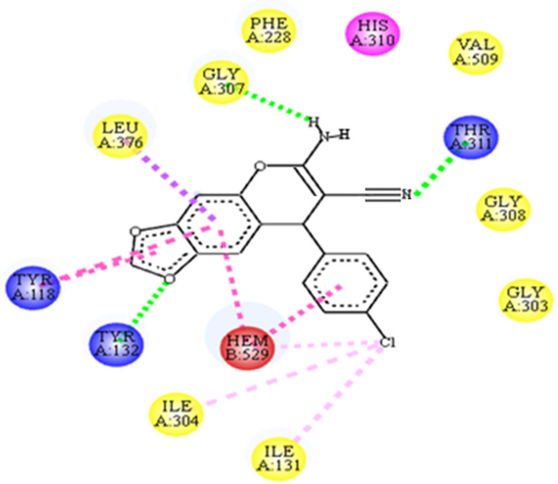

PHE A:228
HIS A:310
VAL A:509
GLY A:307
LEU A:376
THR A:311
GLY A:308
TYR A:118
GLY A:303
TYR A:132
HEM B:529
ILE A:304
ILE A:131

4d

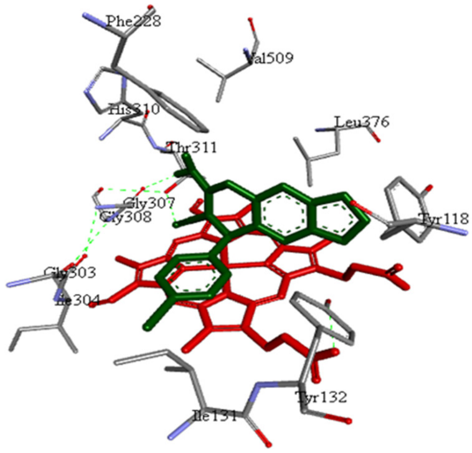

Phe228
Val509
His310
Leu376
Thr311
Gly307
Gly308
Tyr118
Gly303
Ile304
Tyr132
Ile131

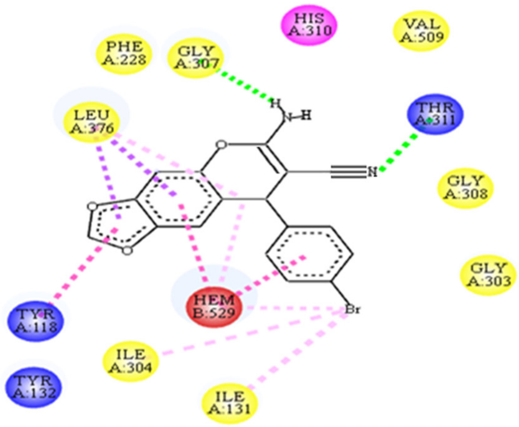

HIS A:310
VAL A:509
PHE A:228
GLY A:307
LEU A:376
THR A:311
GLY A:308
GLY A:303
TYR A:118
HEM B:529
ILE A:304
TYR A:132
ILE A:131

4e

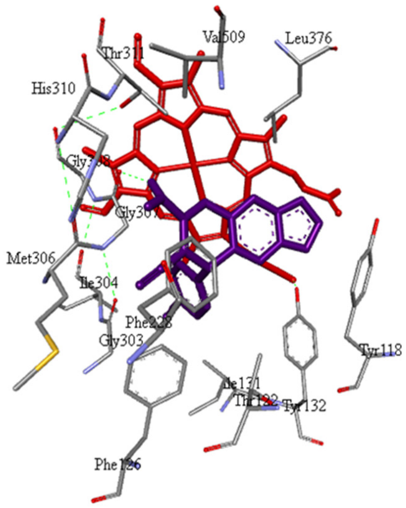

Val509
Leu376
Thr311
His310
Met306
Ile304
Phe228
Gly303
Tyr118
Tyr132
Phe126

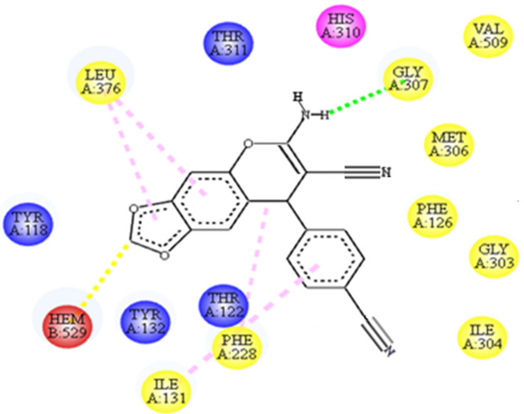

THR A:311
HIS A:310
VAL A:509
LEU A:376
GLY A:307
MET A:306
TYR A:118
PHE A:126
GLY A:303
HEM B:529
TYR A:132
THR A:122
PHE A:228
ILE A:304
ILE A:131

4i

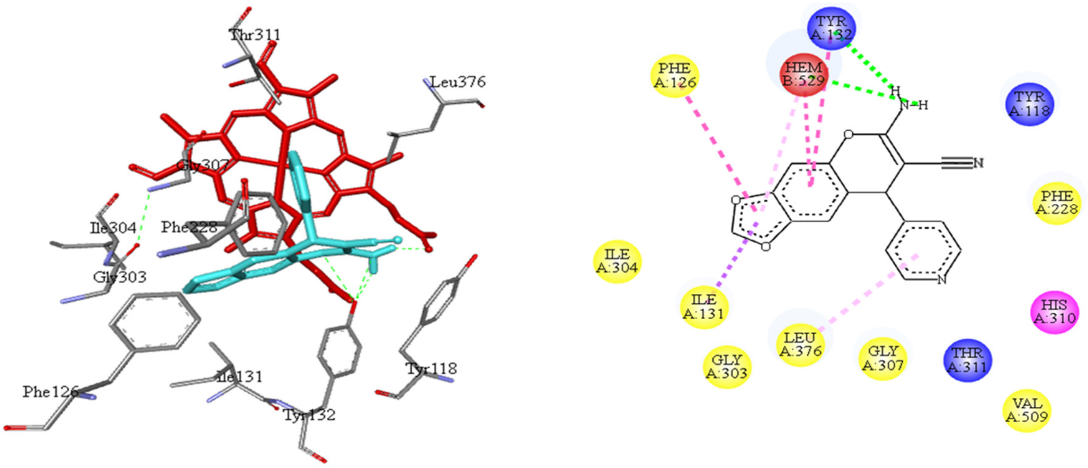

Figure S139. Schematic representation of binding mode between 2-amino-3-cyano-4-aryl-6,7-methylendioxy-4*H*-chromenes **4a-i** and the reference compound fluconazole **8** at the active site of CYP51CA (*C. albicans*). Models 3D and 2D are shown. In 3D model hydrophilic bonds and amino acid residues that are part of the active site of CYP51 are represented. In the 2D model, the following interactions are portrayed with dotted lines: conventional hydrogen bonds (green), carbon-hydrogen (yellow), π-cation (orange), π-sigma (purple), π-alkyl (pink), T-shaped π-π (fushia), and halogen (cyan). Heme group is in red. The solvent accessible surface is illustrated for the amino acid residues and ligands. The amino acids are denoted in pink (basic), yellow (hydrophobic) and blue (hydrophilic).

6a

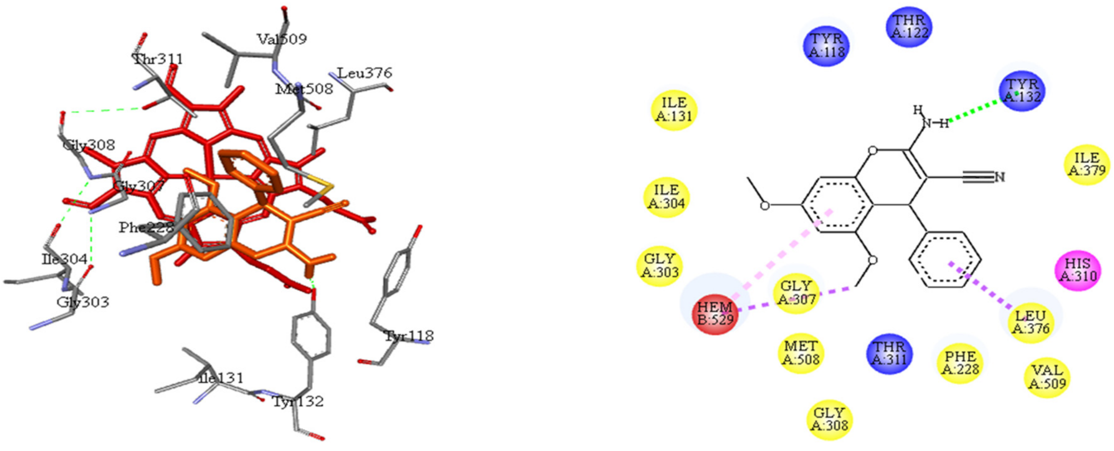

6b

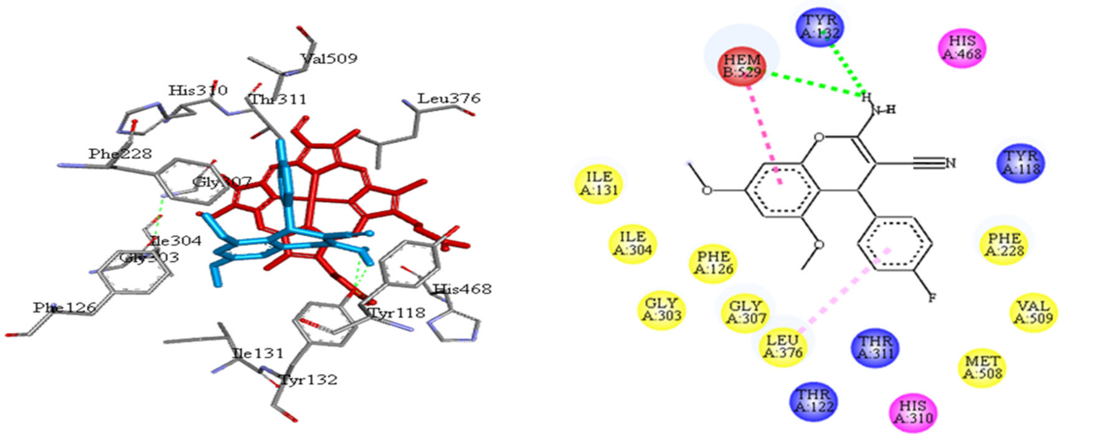
Val509
His310
Thr311
Leu376
Phe228
Gly307
Ile304
Gly303
Phe126
His468
Tyr118
Ile131
Tyr132
TYR A:132
HIS A:468
HEM B:529
N-H
N
TYR A:118
ILE A:131
ILE A:304
PHE A:126
PHE A:228
F
GLY A:303
GLY A:307
VAL A:509
LEU A:376
THR A:311
MET A:508
THR A:122
HIS A:310

6c

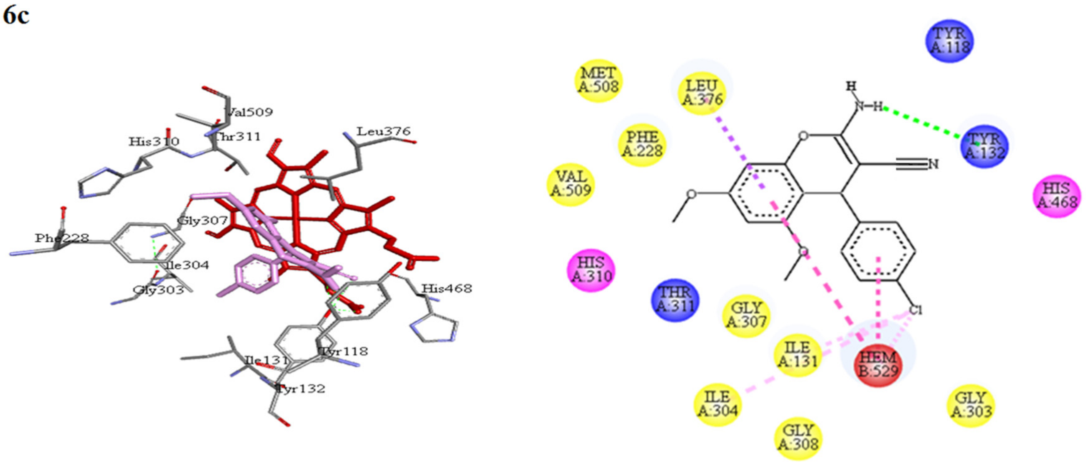
Val509
His310
Thr311
Leu376
Gly307
Phe228
Ile304
Gly303
His468
Tyr118
Ile131
Tyr132
TYR A:118
MET A:508
LEU A:376
N-H
TYR A:132
PHE A:228
N
VAL A:509
HIS A:468
HIS A:310
THR A:311
GLY A:307
Cl
ILE A:131
HEM B:529
ILE A:304
GLY A:303
GLY A:308

6d

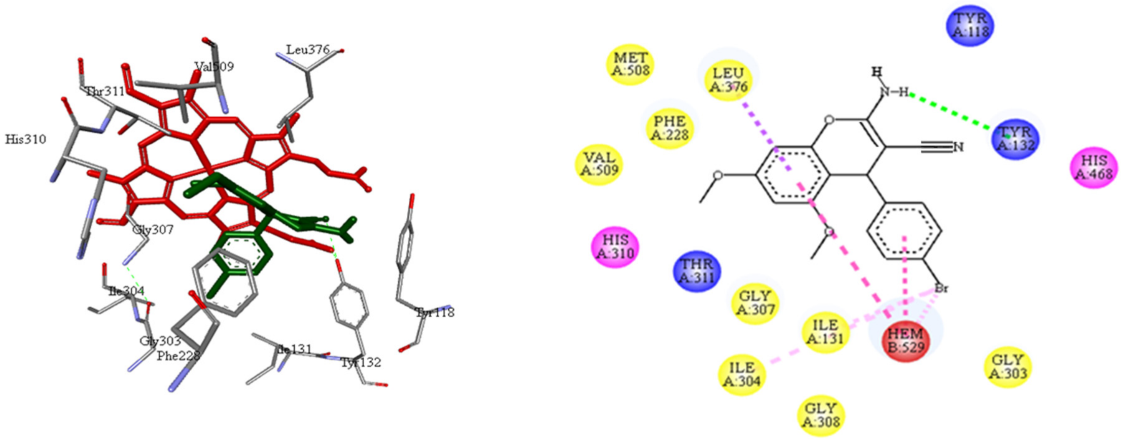
Leu376
Val509
Thr311
His310
Gly307
Ile304
Gly303
Phe228
Tyr118
Ile131
Tyr132
TYR A:118
MET A:508
LEU A:376
N-H
TYR A:132
PHE A:228
N
VAL A:509
HIS A:468
HIS A:310
THR A:311
Br
GLY A:307
ILE A:131
HEM B:529
ILE A:304
GLY A:303
GLY A:308

6e

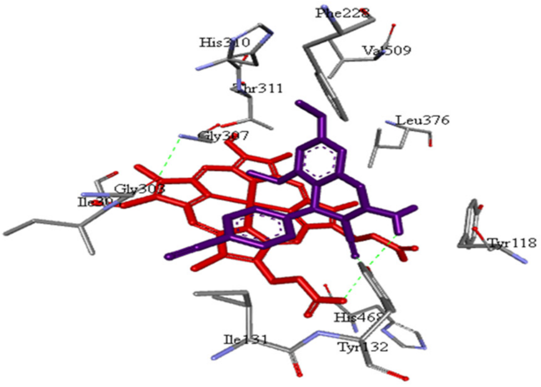
Phe228
His310
Val509
Thr311
Leu376
Gly307
Gly303
Tyr118
His468
Ile131
Tyr132

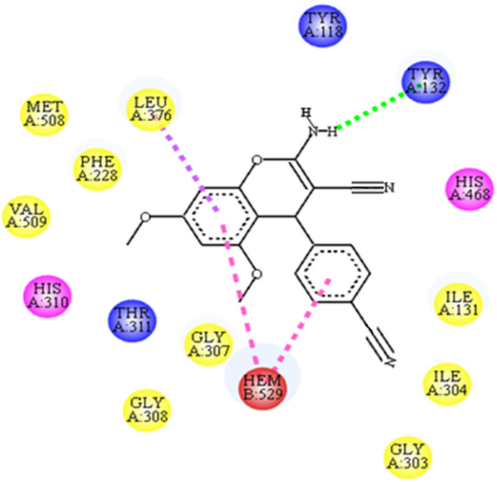
TYR A:118
TYR A:132
MET A:508
LEU A:376
PHE A:228
HIS A:468
VAL A:509
HIS A:310
THR A:311
GLY A:307
ILE A:131
HEM B:529
ILE A:304
GLY A:308
GLY A:303

6f

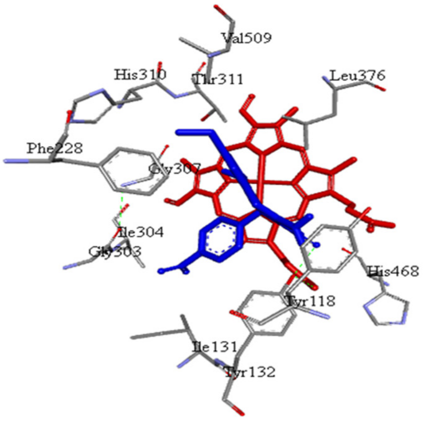
Val509
His310
Thr311
Leu376
Phe228
Gly307
Ile304
Gly303
His468
Tyr118
Ile131
Tyr132

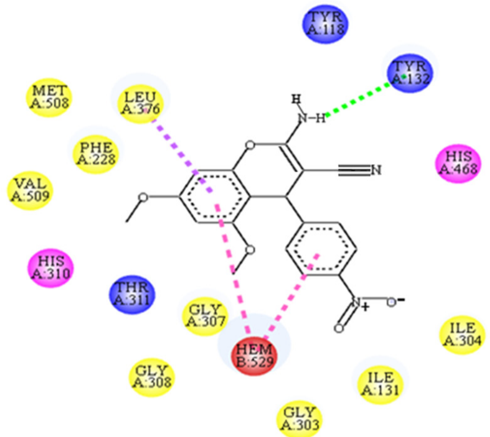
TYR A:118
TYR A:132
MET A:508
LEU A:376
PHE A:228
HIS A:468
VAL A:509
HIS A:310
THR A:311
GLY A:307
HEM B:529
ILE A:304
GLY A:308
ILE A:131
GLY A:303

6g

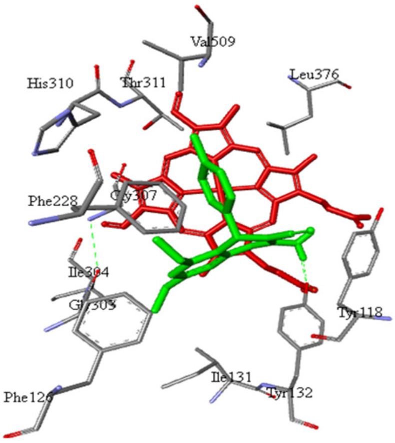
Val509
His310
Thr311
Leu376
Phe228
Gly307
Ile304
Gly303
Tyr118
Ile131
Tyr132

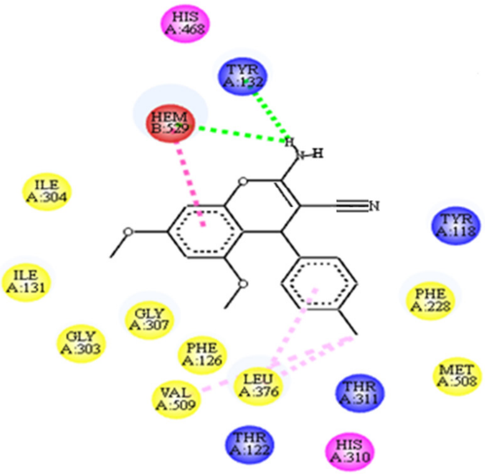
HIS A:468
TYR A:132
HEM B:529
ILE A:304
TYR A:118
ILE A:131
PHE A:228
GLY A:307
GLY A:303
PHE A:126
MET A:508
VAL A:509
LEU A:376
THR A:311
THR A:122
HIS A:310

6h

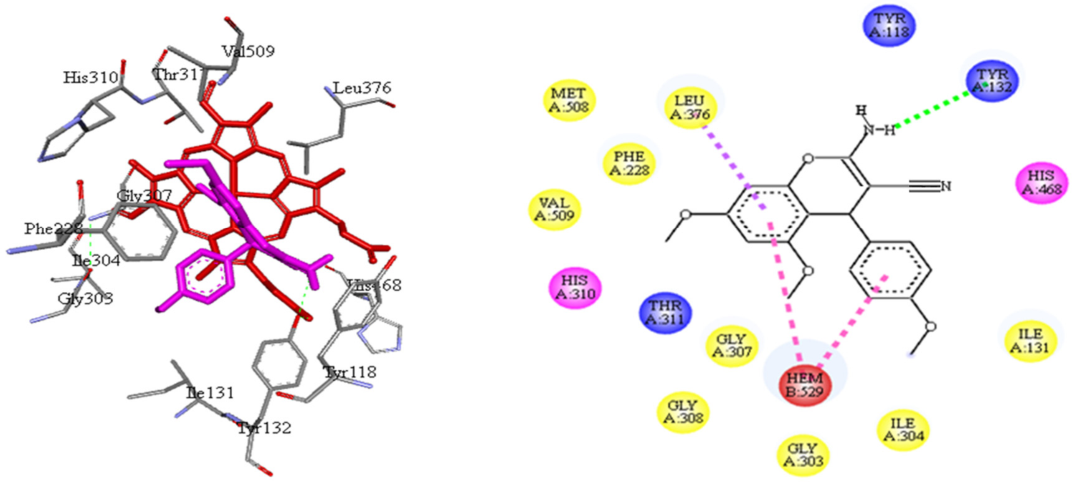

Figure S140. Schematic representation of binding mode between 2-amino-3-cyano-5,7-dimethoxy-4-aryl-4*H*-chromenes **6a-h** at the active site of CYP51CA (*C. albicans*). Models 3D and 2D are shown. In 3D model hydrophilic bonds and amino acid residues that are part of the active site of CYP51 are represented. In the 2D model, the following interactions are portrayed with dotted lines: conventional hydrogen bonds (green), carbon-hydrogen (yellow), π-cation (orange), π-sigma (purple), π-alkyl (pink), T-shaped π-π (fushia), and halogen (cyan). Heme group is in red. The solvent accessible surface is illustrated for the amino acid residues and ligands. The amino acids are denoted in pink (basic), yellow (hydrophobic) and blue (hydrophilic).

Fluconazole **8**

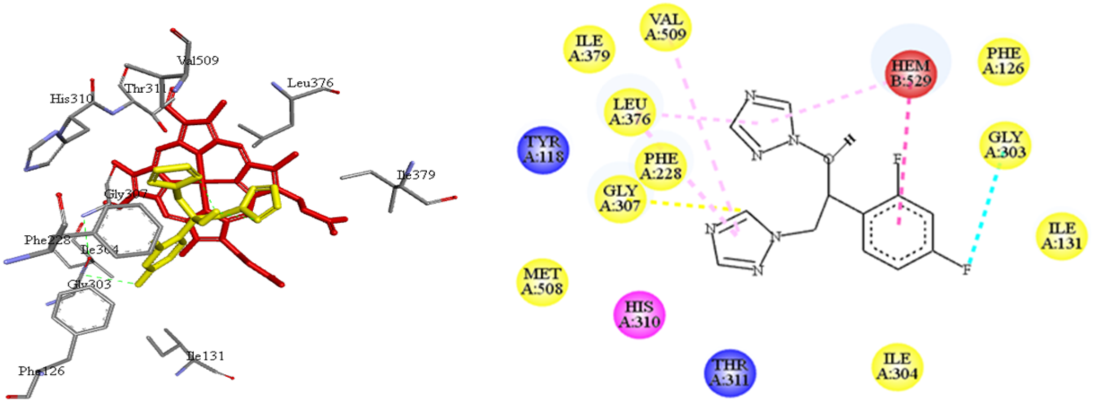

4a

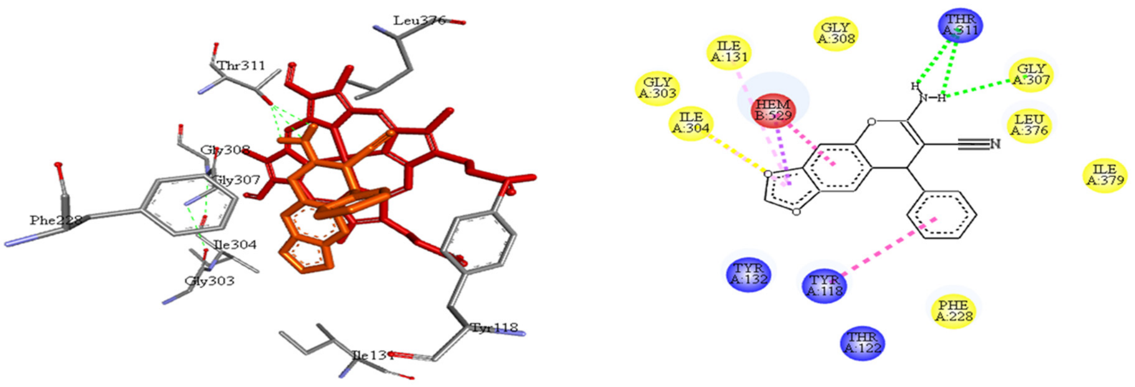
Leu376
Thr311
Gly308
Gly307
Phe228
Ile304
Gly303
Tyr118
ILE A:131
GLY A:308
THR A:311
GLY A:303
HEM B:529
GLY A:307
ILE A:304
LEU A:376
ILE A:379
TYR A:132
TYR A:118
PHE A:228
THR A:122

4b

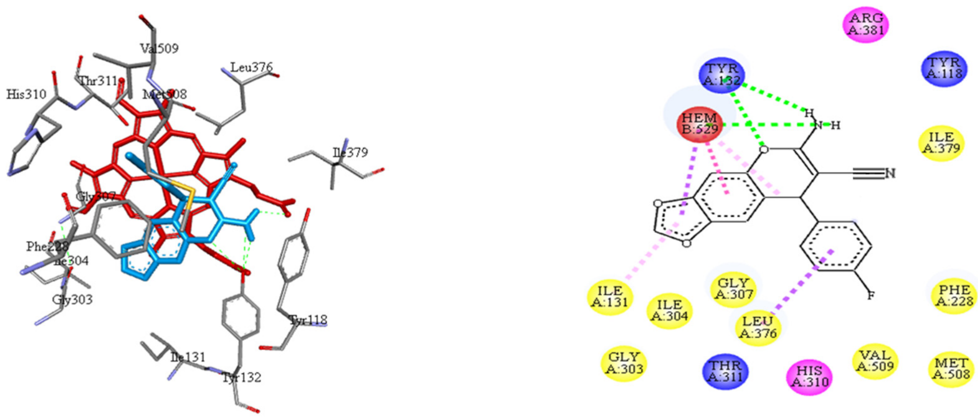
Val509
Leu376
His310
Thr311
Ile379
Gly307
Ile304
Gly303
Tyr118
Ile131
Tyr132
ARG A:381
TYR A:132
TYR A:118
HEM B:529
ILE A:379
ILE A:131
ILE A:304
GLY A:307
LEU A:376
PHE A:228
GLY A:303
THR A:311
HIS A:310
VAL A:509
MET A:508

4c

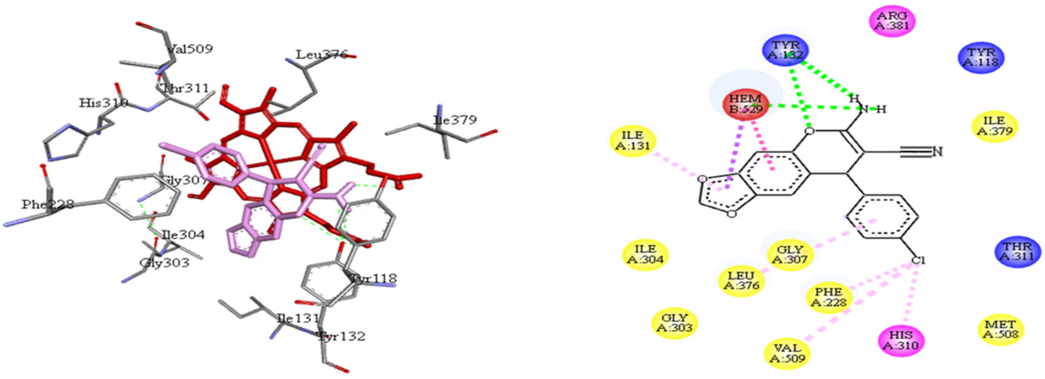
Val509
Leu376
Thr311
His310
Ile379
Phe228
Ile304
Gly303
Tyr118
Ile131
Tyr132
ARG A:381
TYR A:132
TYR A:118
HEM B:529
ILE A:379
ILE A:131
ILE A:304
GLY A:307
THR A:311
LEU A:376
PHE A:228
GLY A:303
HIS A:310
MET A:508
VAL A:509

4e

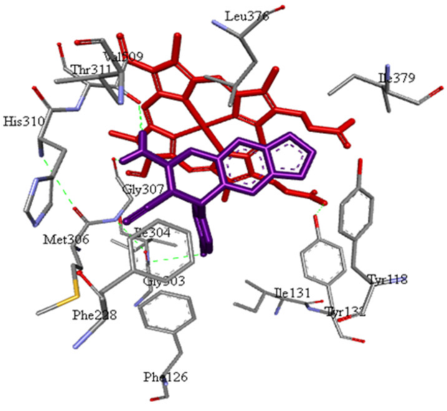
Leu376
Val509
Thr311
Ile379
His310
Gly307
Ile304
Met306
Gly303
Tyr118
Ile131
Tyr132
Phe228
Phe126

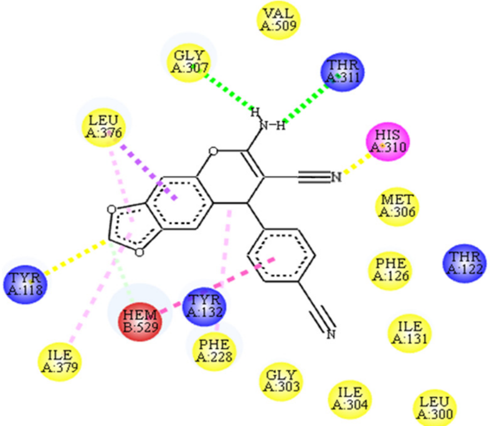
VAL A:509
GLY A:307
THR A:311
LEU A:376
HIS A:310
MET A:306
PHE A:126
THR A:122
TYR A:118
TYR A:132
HEM B:529
ILE A:131
PHE A:228
ILE A:379
GLY A:303
ILE A:304
LEU A:300

4f

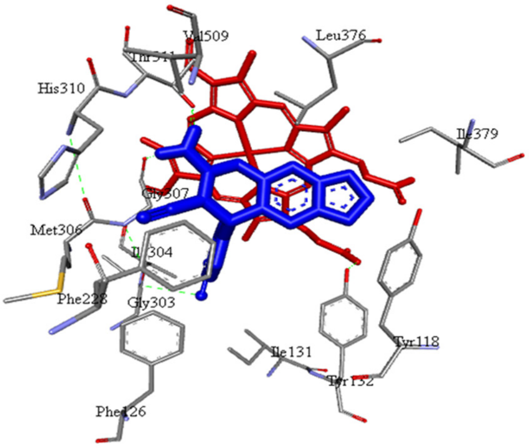
Val509
Leu376
Thr311
His310
Ile379
Gly307
Met306
Ile304
Phe228
Gly303
Tyr118
Ile131
Tyr132
Phe126

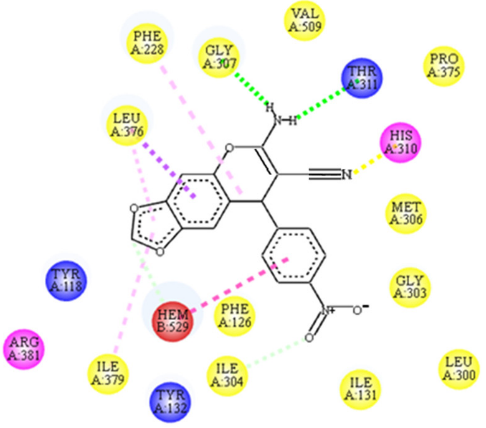
VAL A:509
PHE A:228
GLY A:307
PRO A:375
THR A:311
LEU A:376
HIS A:310
MET A:306
TYR A:118
GLY A:303
HEM B:529
PHE A:126
ARG A:381
ILE A:379
ILE A:304
LEU A:300
ILE A:131
TYR A:132

4g

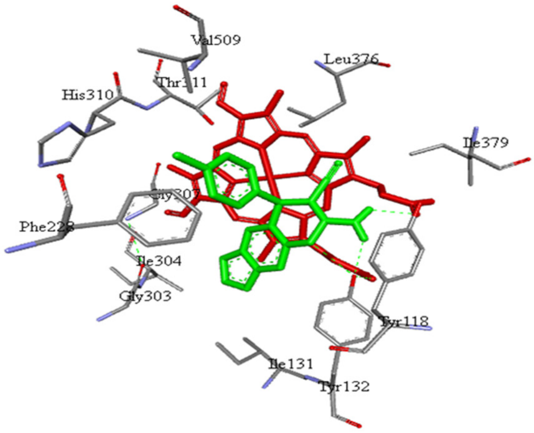
Val509
Leu376
His310
Thr311
Ile379
Gly307
Phe228
Ile304
Gly303
Tyr118
Ile131
Tyr132

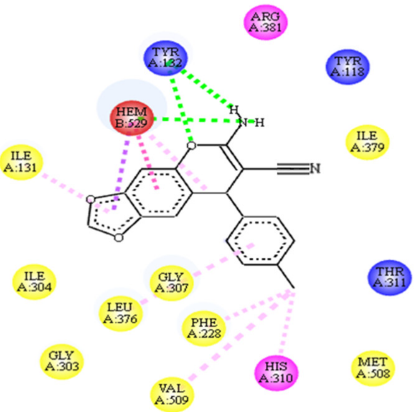
ARG A:381
TYR A:132
TYR A:118
HEM B:529
ILE A:379
ILE A:131
ILE A:304
GLY A:307
THR A:311
LEU A:376
PHE A:228
GLY A:303
HIS A:310
MET A:508
VAL A:509

4h

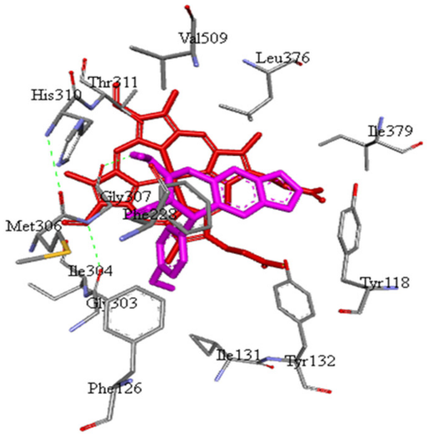

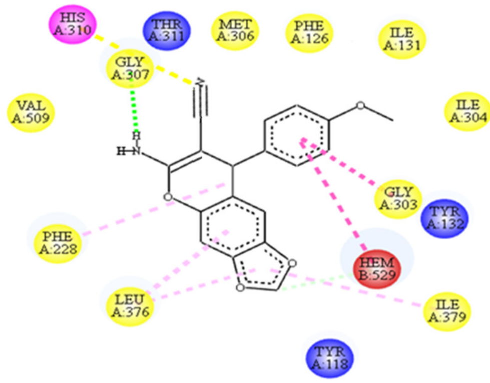

4i

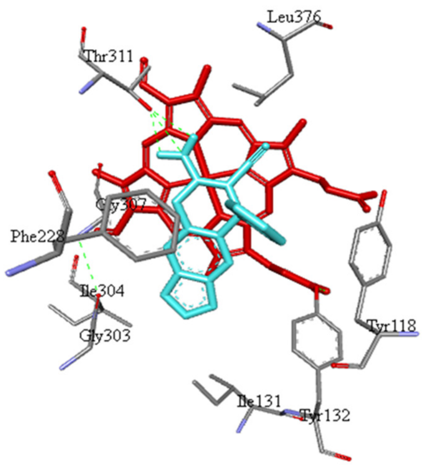

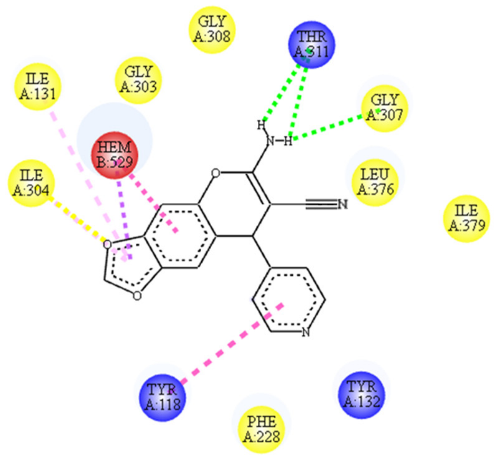

Figure S141. Schematic representation of binding mode between 2-amino-3-cyano-4-aryl-6,7-methylendioxy-4*H*-chromenes **4a-c**, **4e-i** and the reference compound fluconazole **8** at the active site of CYP51CD (*C. dubliniensis*). Models 3D and 2D are shown. In 3D model hydrophilic bonds and amino acid residues that are part of the active site of CYP51 are represented. In the 2D model, the following interactions are portrayed with dotted lines: conventional hydrogen bonds (green), carbon-hydrogen (yellow), π-cation (orange), π-sigma (purple), π-alkyl (pink), T-shaped π-π (fushia), and halogen (cyan). Heme group is in red. The solvent accessible surface is illustrated for the amino acid residues and ligands. The amino acids are denoted in pink (basic), yellow (hydrophobic) and blue (hydrophilic).

6b

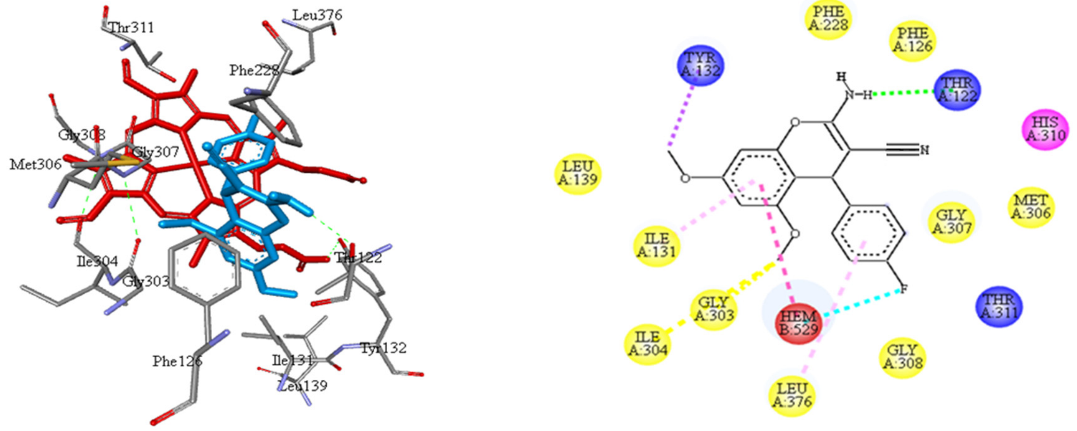
Thr311
Leu376
Phe228
Gly308
Gly307
Met306
Ile304
Gly303
Thr122
Tyr132
Phe126
Ile131
Leu139
PHE A:228
PHE A:126
TYR A:132
THR A:122
HIS A:310
LEU A:139
GLY A:307
MET A:306
ILE A:131
GLY A:303
HEM B:529
THR A:311
ILE A:304
GLY A:308
LEU A:376

6c

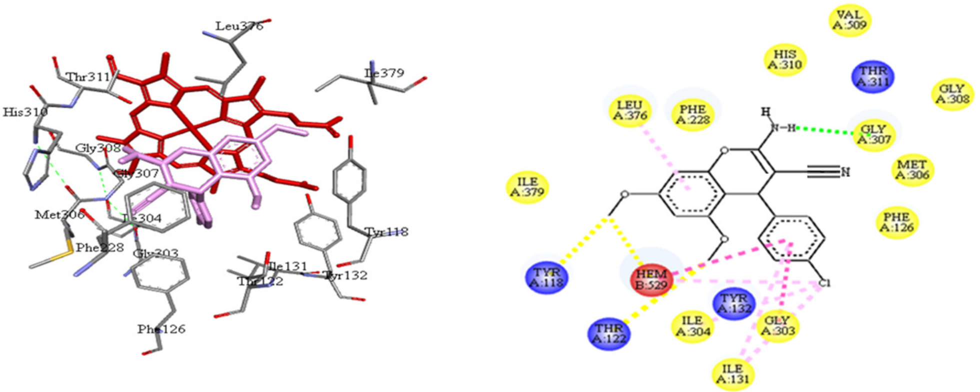
Leu376
Thr311
Ile379
His310
Gly308
Gly307
Met306
Ile304
Phe228
Gly303
Tyr118
Ile131
Thr122
Tyr132
Phe126
VAL A:509
HIS A:310
THR A:311
GLY A:308
LEU A:376
PHE A:228
GLY A:307
MET A:306
ILE A:379
PHE A:126
TYR A:118
HEM B:529
TYR A:132
THR A:122
ILE A:304
GLY A:303
ILE A:131

6d

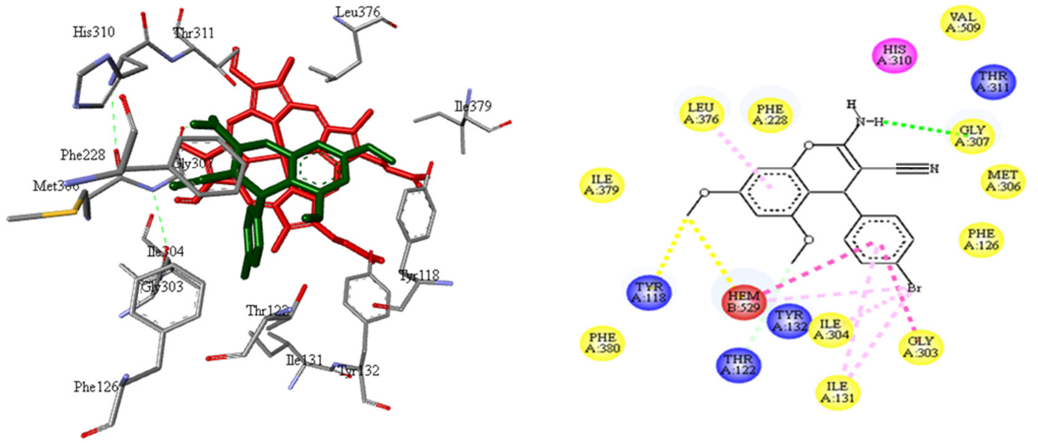
His310
Thr311
Leu376
Ile379
Phe228
Met306
Ile304
Gly303
Tyr118
Thr122
Ile131
Tyr132
Phe126
VAL A:509
HIS A:310
THR A:311
LEU A:376
PHE A:228
GLY A:307
MET A:306
ILE A:379
PHE A:126
TYR A:118
HEM B:529
TYR A:132
ILE A:304
GLY A:303
PHE A:380
THR A:122
ILE A:131

6e

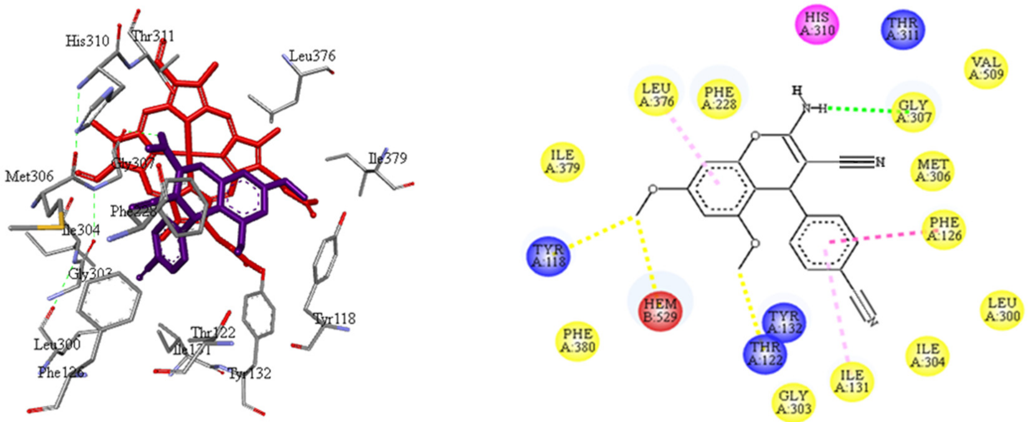

His310
Thr311
Leu376
Ile379
Met306
Gly307
Phe228
Ile304
Gly303
Leu300
Phe126
Thr122
Ile131
Tyr132
Tyr118
HIS A:310
THR A:311
VAL A:509
LEU A:376
PHE A:228
GLY A:307
ILE A:379
MET A:306
PHE A:126
TYR A:118
HEM B:529
TYR A:132
THR A:122
LEU A:300
ILE A:304
PHE A:380
GLY A:303
ILE A:131

6f

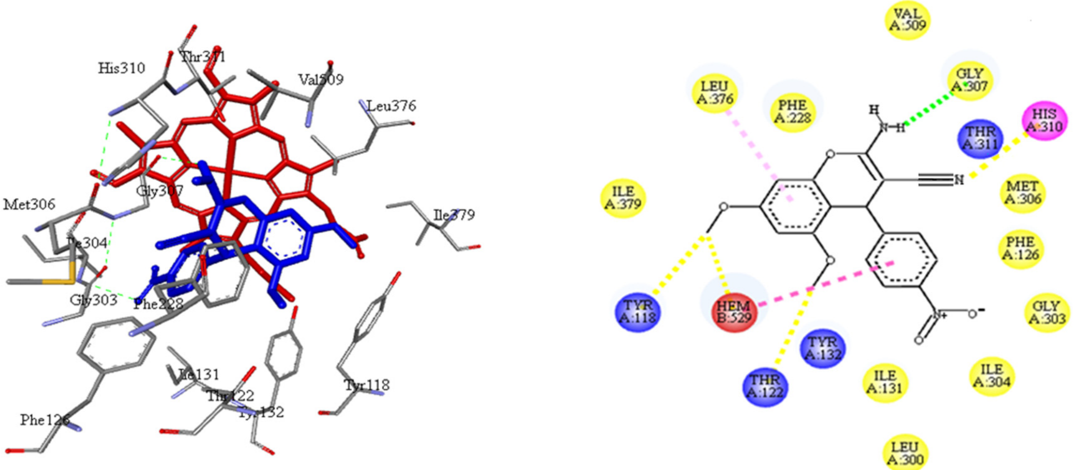

His310
Thr311
Val509
Leu376
Met306
Gly307
Ile379
Ile304
Gly303
Phe228
Ile131
Thr122
Tyr132
Tyr118
Phe126
VAL A:509
GLY A:307
LEU A:376
PHE A:228
HIS A:310
THR A:311
ILE A:379
MET A:306
PHE A:126
TYR A:118
HEM B:529
GLY A:303
TYR A:132
THR A:122
ILE A:131
ILE A:304
LEU A:300

6g

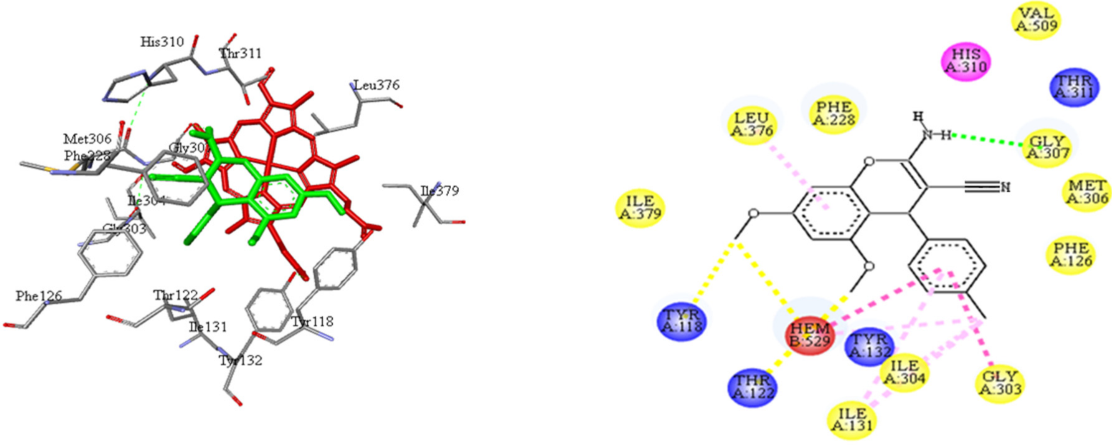

His310
Thr311
Leu376
Met306
Phe228
Gly307
Ile379
Ile304
Gly303
Phe126
Thr122
Ile131
Tyr118
Tyr132
VAL A:509
HIS A:310
THR A:311
LEU A:376
PHE A:228
GLY A:307
MET A:306
ILE A:379
PHE A:126
TYR A:118
HEM B:529
TYR A:132
ILE A:304
GLY A:303
THR A:122
ILE A:131

6h

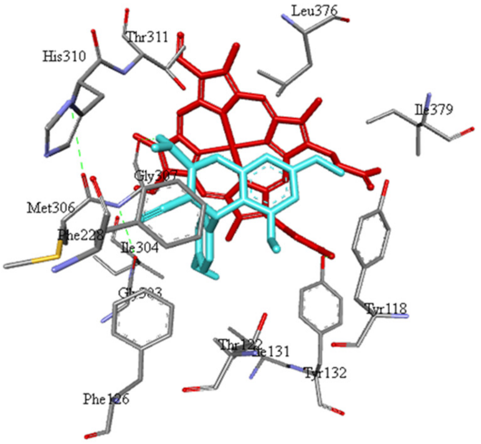

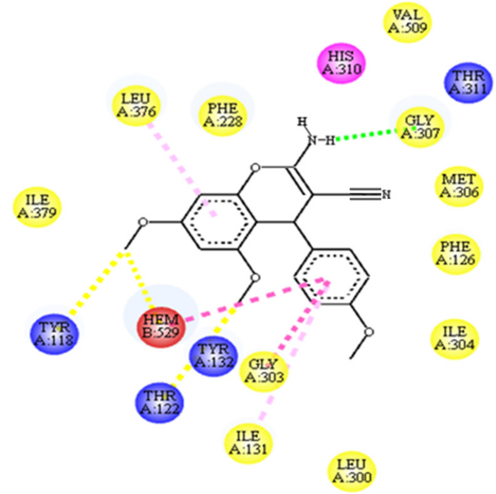

Figure S142. Schematic representation of binding mode between 2-amino-3-cyano-5,7-dimethoxy-4-aryl-4*H*-chromenes **6b-h** at the active site of CYP51CD (*C. dubliniensis*). Models 3D and 2D are shown. In 3D model hydrophilic bonds and amino acid residues that are part of the active site of CYP51 are represented. In the 2D model, the following interactions are portrayed with dotted lines: conventional hydrogen bonds (green), carbon-hydrogen (yellow), π-cation (orange), π-sigma (purple), π-alkyl (pink), T-shaped π-π (fushia), and halogen (cyan). Heme group is in red. The solvent accessible surface is illustrated for the amino acid residues and ligands. The amino acids are denoted in pink (basic), yellow (hydrophobic) and blue (hydrophilic).

Fluconazole **8**

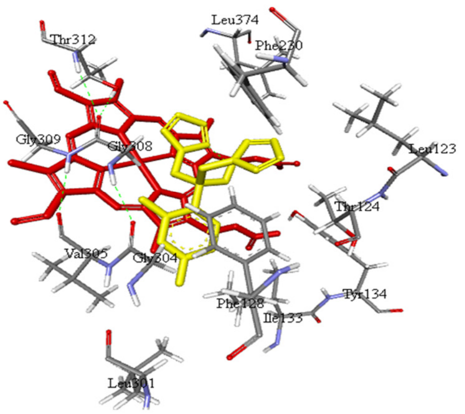

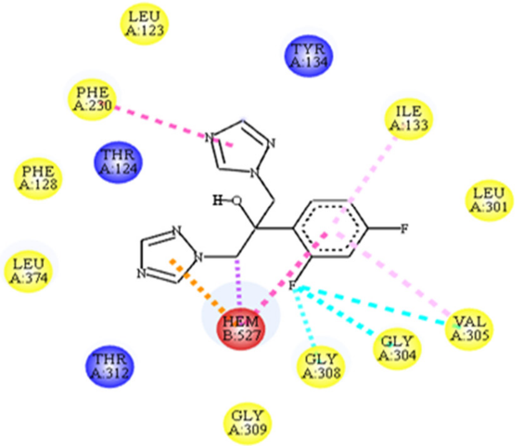

4a

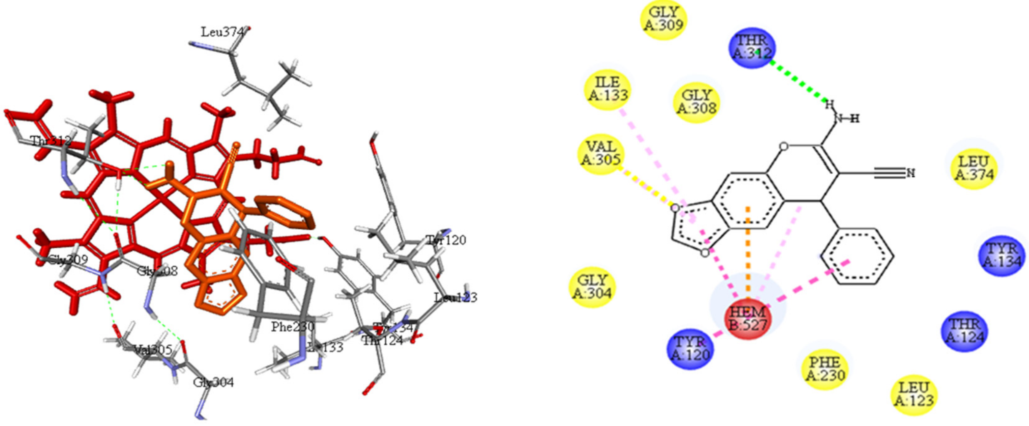
Leu374
Phe230
Gly309
Gly308
Val305
Gly304
Tyr120
GLY A:309
THR A:312
ILE A:133
GLY A:308
VAL A:305
LEU A:374
TYR A:134
GLY A:304
HEM B:527
THR A:124
TYR A:120
PHE A:230
LEU A:123

4b

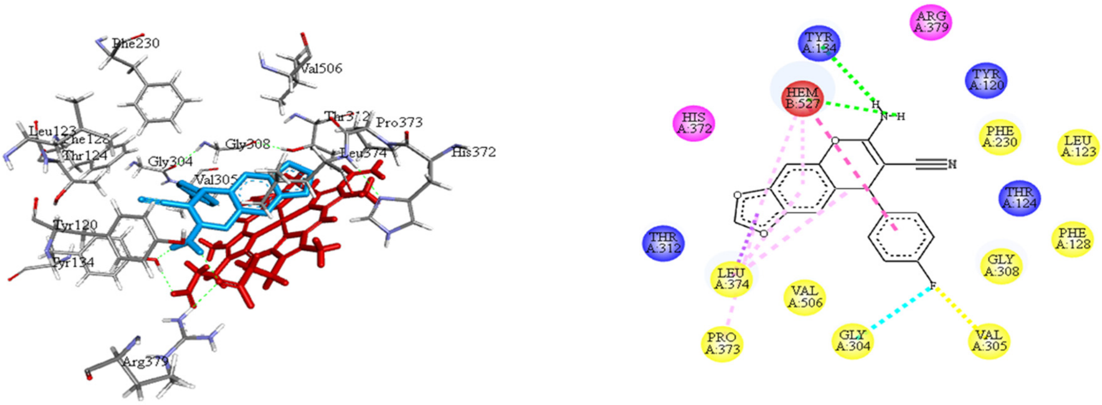
Phe230
Val506
Thr312
Pro373
Gly308
His372
Leu123
Thr124
Gly304
Leu374
Val305
Tyr120
Tyr134
Arg379
TYR A:134
ARG A:379
HEM B:527
TYR A:120
HIS A:372
PHE A:230
LEU A:123
THR A:124
THR A:312
PHE A:128
LEU A:374
VAL A:506
GLY A:308
PRO A:373
GLY A:304
VAL A:305

4c

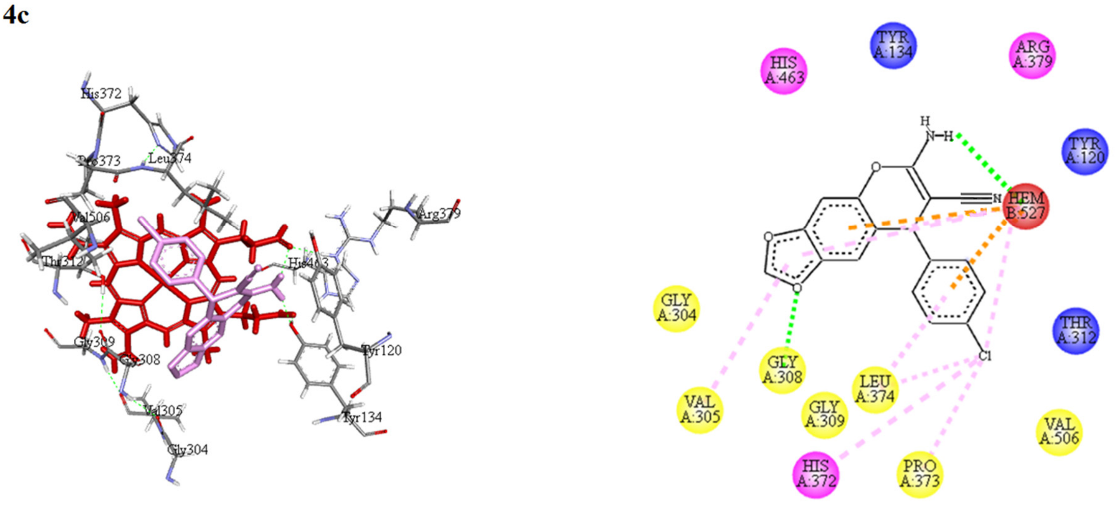
His372
Pro373
Leu374
Val506
Arg379
Thr312
His463
Gly309
Gly308
Tyr120
Val305
Tyr134
Gly304
HIS A:463
TYR A:134
ARG A:379
TYR A:120
HEM B:527
GLY A:304
THR A:312
GLY A:308
LEU A:374
VAL A:305
GLY A:309
VAL A:506
HIS A:372
PRO A:373

4d

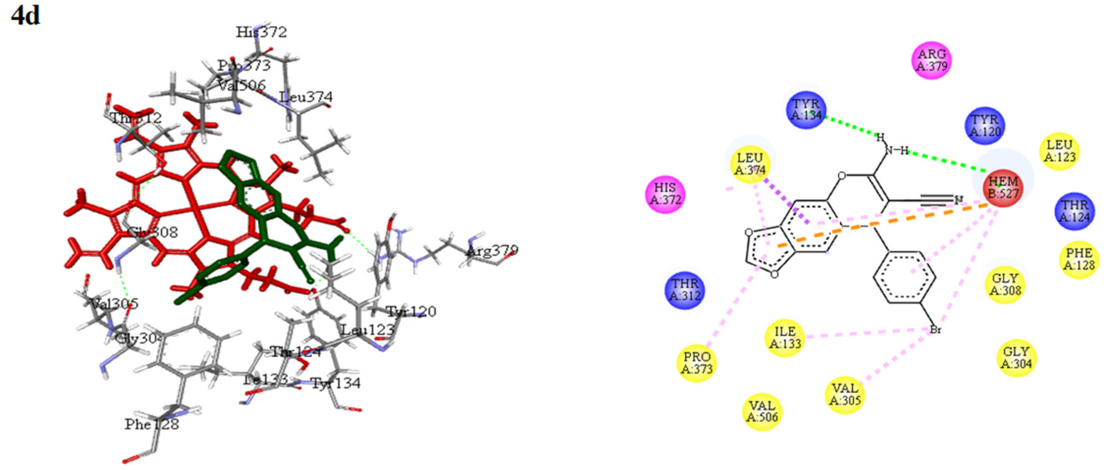

His372
Pro373
Val506
Leu374
Thr312
Gly308
Arg379
Val305
Gly304
Tyr120
Leu123
Thr124
Ile133
Tyr134
Phe128
ARG A:379
TYR A:134
TYR A:120
LEU A:123
LEU A:374
HEM B:527
HIS A:372
THR A:124
PHE A:128
THR A:312
GLY A:308
ILE A:133
PRO A:373
GLY A:304
VAL A:305
VAL A:506
Br

4e

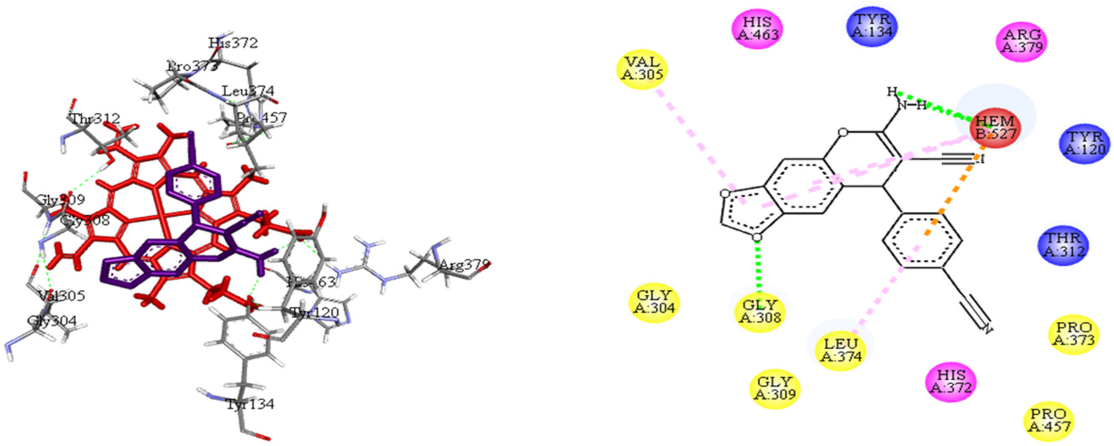

His372
Pro373
Leu374
Pro457
Thr312
Gly309
Gly308
Arg379
Val305
Gly304
His463
Tyr120
Tyr134
HIS A:463
TYR A:134
ARG A:379
VAL A:305
HEM B:527
TYR A:120
THR A:312
GLY A:304
GLY A:308
PRO A:373
LEU A:374
GLY A:309
HIS A:372
PRO A:457

4f

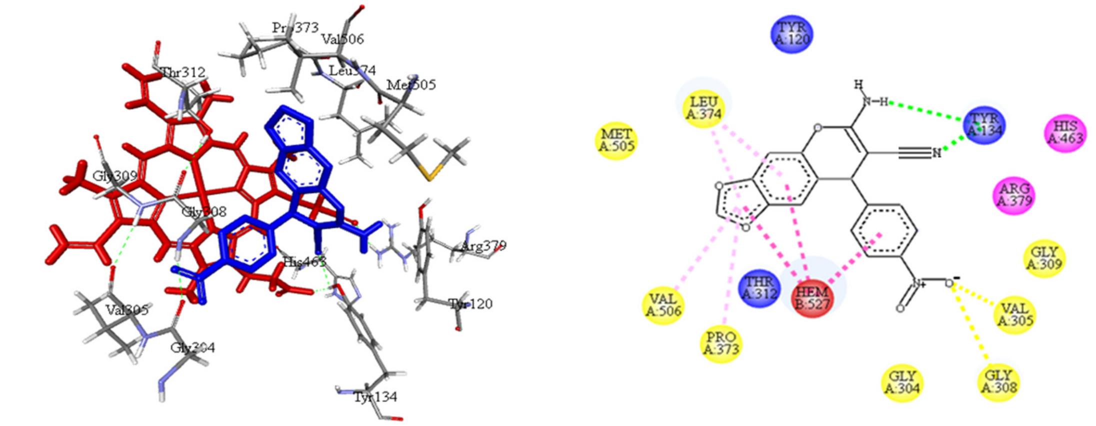

Pro373
Val506
Leu374
Met505
Thr312
Gly309
Gly308
His463
Arg379
Val305
Gly304
Tyr120
Tyr134
TYR A:120
LEU A:374
TYR A:134
HIS A:463
MET A:505
ARG A:379
GLY A:309
VAL A:506
THR A:312
HEM B:527
VAL A:305
PRO A:373
GLY A:304
GLY A:308

4g

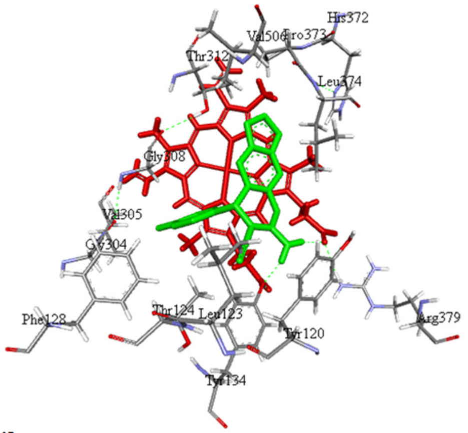

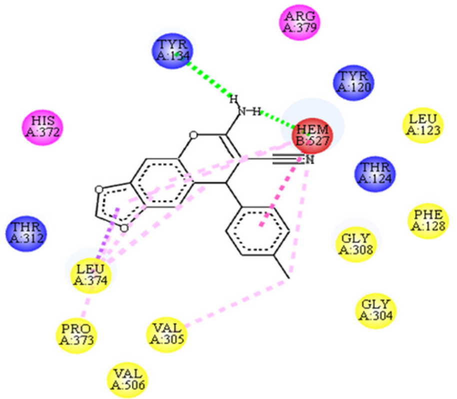
ARG A:379
TYR A:134
TYR A:120
HIS A:372
HEM B:527
LEU A:123
THR A:124
PHE A:128
THR A:312
GLY A:308
LEU A:374
GLY A:304
PRO A:373
VAL A:305
VAL A:506

4h

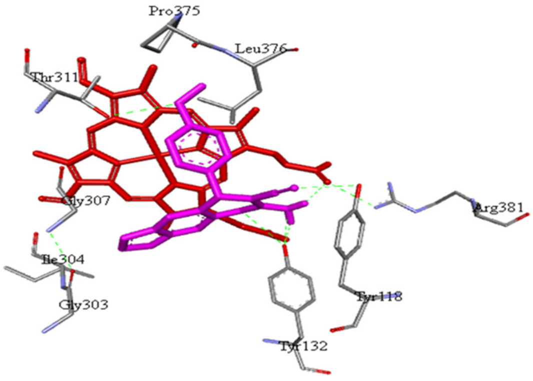

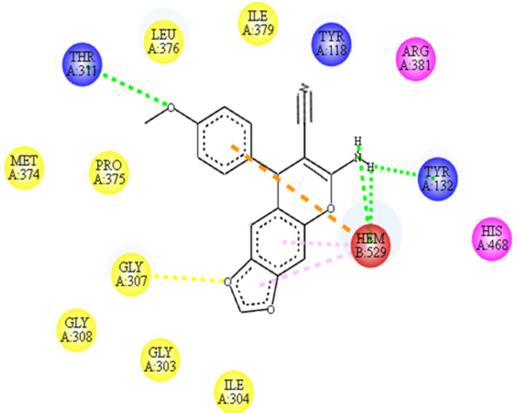
ILE A:379
LEU A:376
TYR A:118
THR A:311
ARG A:381
MET A:374
PRO A:375
TYR A:132
HIS A:468
HEM B:529
GLY A:307
GLY A:308
GLY A:303
ILE A:304

4i

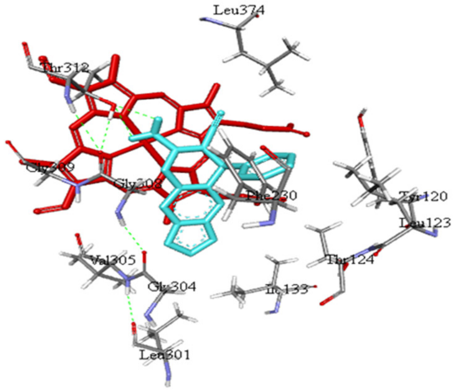

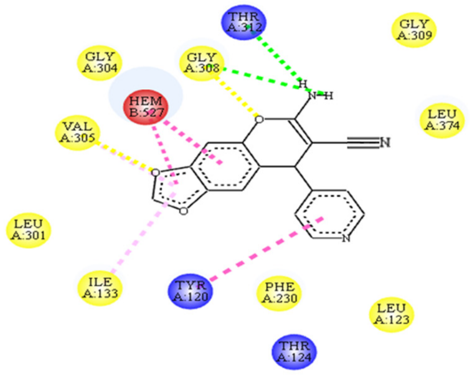
THR A:312
GLY A:309
GLY A:304
GLY A:308
HEM B:527
LEU A:374
VAL A:305
LEU A:301
ILE A:133
TYR A:120
PHE A:230
LEU A:123
THR A:124

Figure S143. Schematic representation of binding mode between 2-amino-3-cyano-4-aryl-6,7-methylendioxy-4*H*-chromenes **4a-i** and the reference compound fluconazole **8** at the active site of CYP51CG (*C. glabrata*). Models 3D and 2D are shown. In 3D model hydrophilic bonds and amino acid residues that are part of the active site of CYP51 are represented. In the 2D model, the following interactions are portrayed with dotted lines: conventional hydrogen bonds (green), carbon-hydrogen (yellow), π-cation (orange), π-sigma (purple), π-alkyl (pink), T-shaped π-π (fushia), and halogen (cyan). Heme group is in red. The solvent accessible surface is illustrated for the amino acid residues and ligands. The amino acids are denoted in pink (basic), yellow (hydrophobic) and blue (hydrophilic).

6a

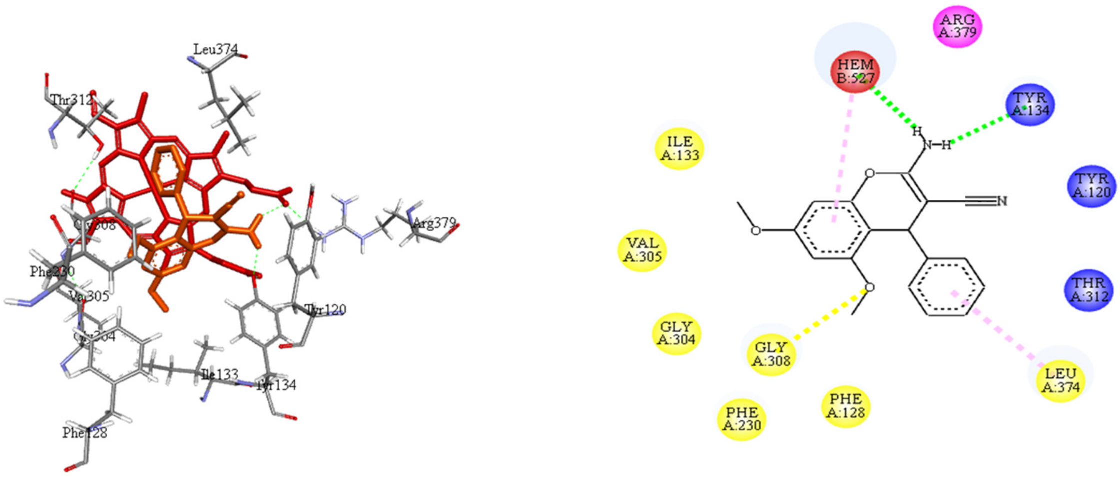

6b

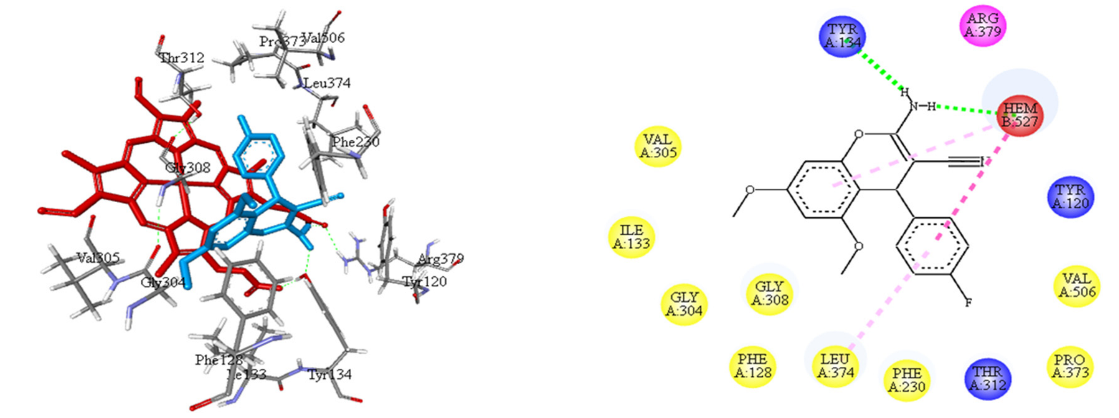

6c

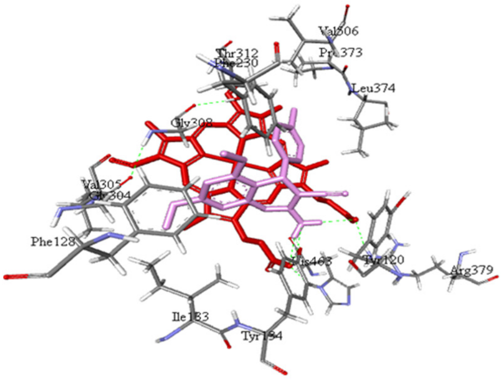

Val306
Thr312
Phe230
Pro373
Leu374
Gly308
Val305
Glu304
Phe128
His463
Tyr120
Arg379
Ile133
Tyr134

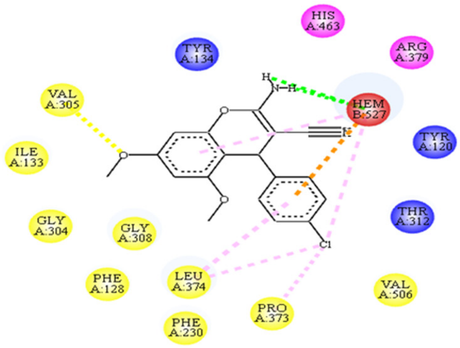

HIS A:463
TYR A:134
ARG A:379
VAL A:305
HEM B:527
ILE A:133
TYR A:120
GLY A:304
GLY A:308
THR A:312
PHE A:128
LEU A:374
VAL A:506
PRO A:373
PHE A:230
Cl

6d

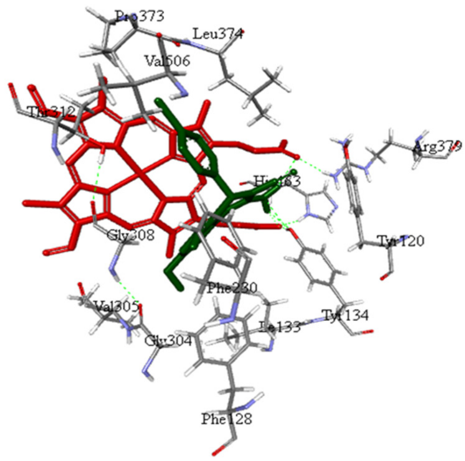

Pro373
Leu374
Val506
Thr312
Arg379
His463
Gly308
Tyr120
Phe230
Val305
Leu133
Tyr134
Glu304
Phe128

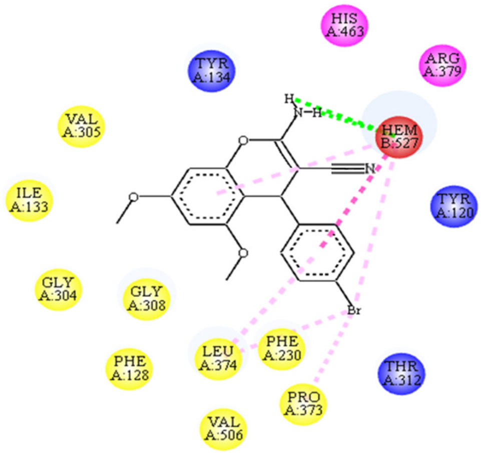

HIS A:463
TYR A:134
ARG A:379
VAL A:305
HEM B:527
ILE A:133
TYR A:120
GLY A:304
GLY A:308
Br
PHE A:128
LEU A:374
PHE A:230
THR A:312
PRO A:373
VAL A:506

6e

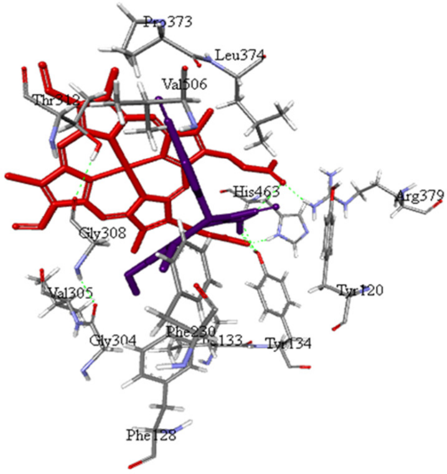

Pro373
Leu374
Val506
Thr312
His463
Arg379
Gly308
Tyr120
Val305
Gly304
Phe230
Tyr134
Phe128

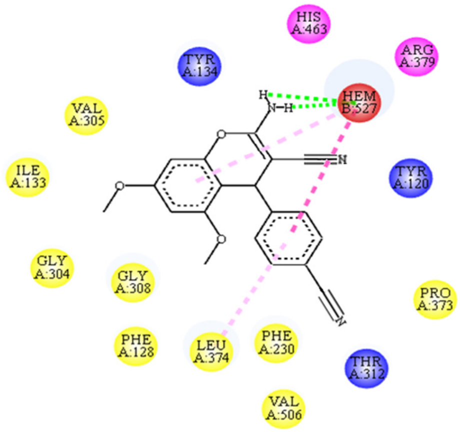

HIS A:463
TYR A:134
ARG A:379
VAL A:305
HEM B:527
ILE A:133
TYR A:120
GLY A:304
GLY A:308
PRO A:373
PHE A:128
LEU A:374
PHE A:230
THR A:312
VAL A:506

6f

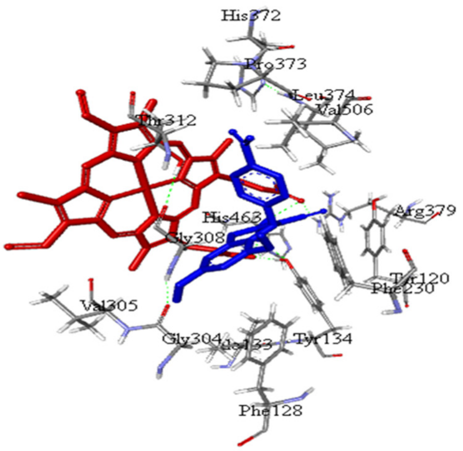
His372
Pro373
Leu374
Val506
Thr312
His463
Arg379
Gly308
Tyr120
Phe230
Val305
Gly304
Tyr134
Phe128

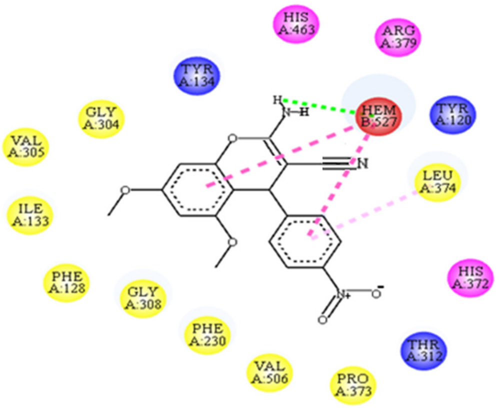
HIS A:463
ARG A:379
TYR A:134
HEM B:527
TYR A:120
GLY A:304
VAL A:305
LEU A:374
ILE A:133
PHE A:128
GLY A:308
HIS A:372
PHE A:230
THR A:312
VAL A:506
PRO A:373

6g

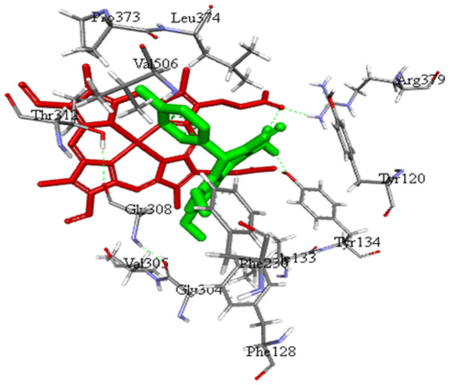
Pro373
Leu374
Val506
Arg379
Tyr120
Gly308
Tyr134
Val305
Gly304
Phe128

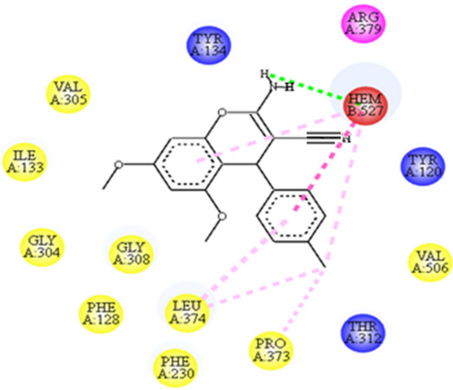
ARG A:379
TYR A:134
HEM B:527
VAL A:305
ILE A:133
TYR A:120
GLY A:304
GLY A:308
VAL A:506
PHE A:128
LEU A:374
THR A:312
PRO A:373
PHE A:230

6h

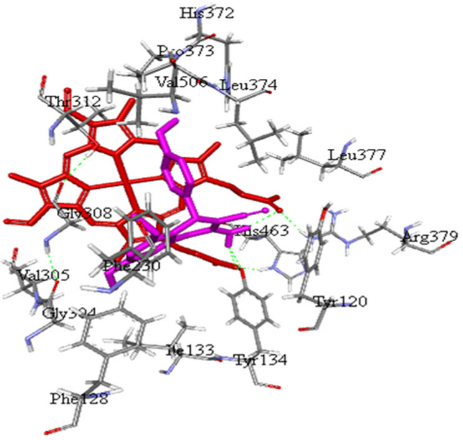
His372
Pro373
Thr312
Val506
Leu374
Leu377
Gly308
Arg379
Phe230
Val305
Tyr120
Tyr134
Phe128

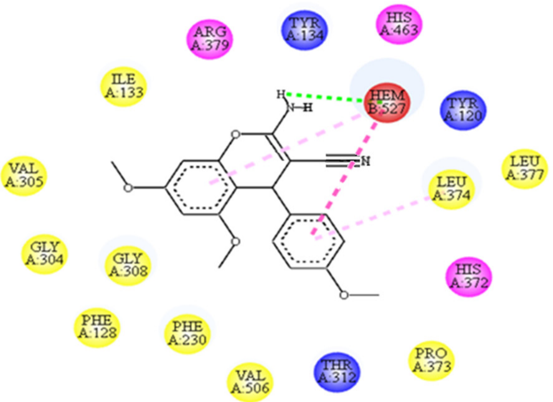
ARG A:379
TYR A:134
HIS A:463
ILE A:133
HEM B:527
TYR A:120
LEU A:377
VAL A:305
LEU A:374
GLY A:304
GLY A:308
HIS A:372
PHE A:128
PHE A:230
PRO A:373
VAL A:506
THR A:312

Figure S144. Schematic representation of binding mode between 2-amino-3-cyano-5,7-dimethoxy-4-aryl-4*H*-chromenes **6a-h** at the active site of CYP51CG (*C. glabrata*). Models 3D and 2D are shown. In 3D model hydrophilic bonds and amino acid residues that are part of the active site of CYP51 are represented. In the 2D model, the following interactions are portrayed with dotted lines: conventional hydrogen bonds (green), carbon-hydrogen (yellow), π-cation (orange), π-sigma (purple), π-alkyl (pink), T-shaped π-π (fushia), and halogen (cyan). Heme group is in red. The solvent accessible surface is illustrated for the amino acid residues and ligands. The amino acids are denoted in pink (basic), yellow (hydrophobic) and blue (hydrophilic).

Fluconazole **8**

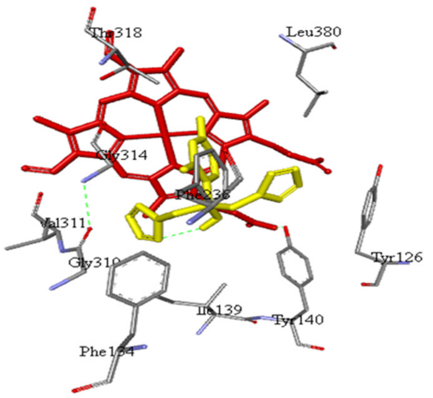

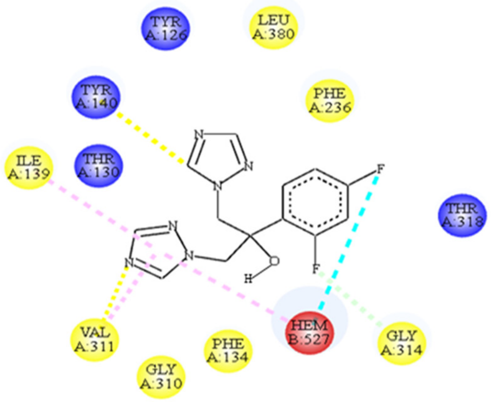

4a

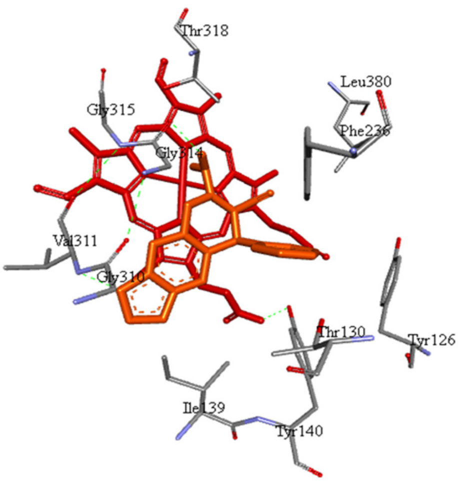

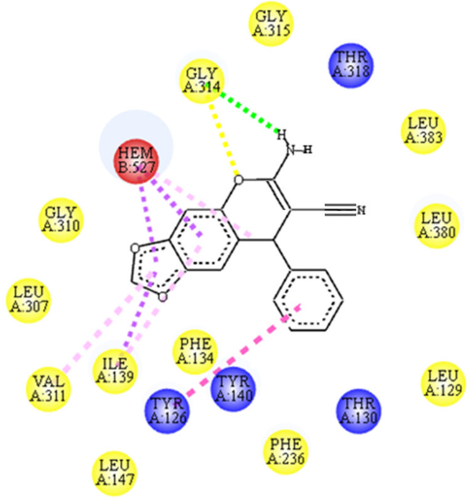

4c

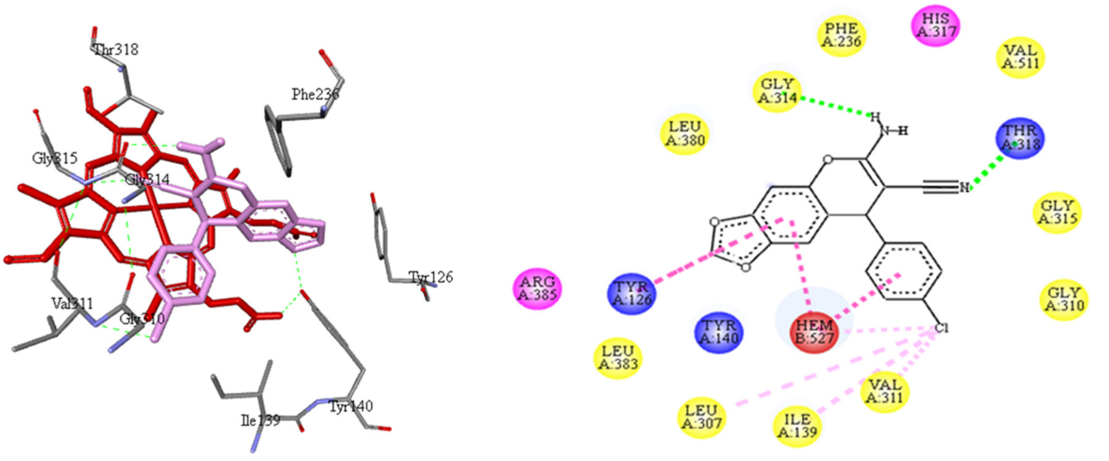
Thr318
Phe236
Gly315
Gly314
Tyr126
Val311
Gly310
Tyr140
Ile139
PHE A:236
HIS A:317
VAL A:511
GLY A:314
LEU A:380
THR A:318
GLY A:315
ARG A:385
TYR A:126
GLY A:310
TYR A:140
HEM B:527
LEU A:383
VAL A:311
LEU A:307
ILE A:139
Cl

4d

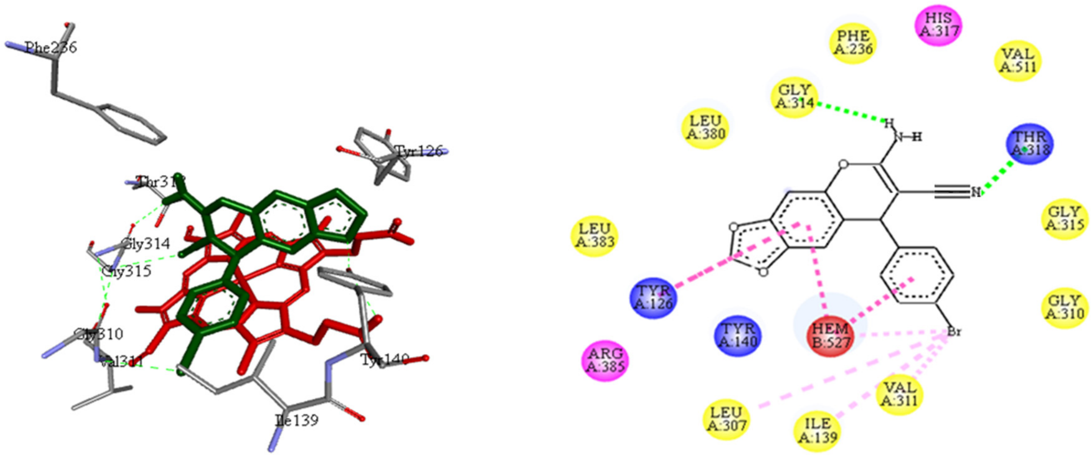
Phe236
Tyr126
Gly314
Gly315
Ile139
PHE A:236
HIS A:317
VAL A:511
GLY A:314
LEU A:380
THR A:318
LEU A:383
GLY A:315
TYR A:126
GLY A:310
TYR A:140
HEM B:527
ARG A:385
VAL A:311
LEU A:307
ILE A:139
Br

4e

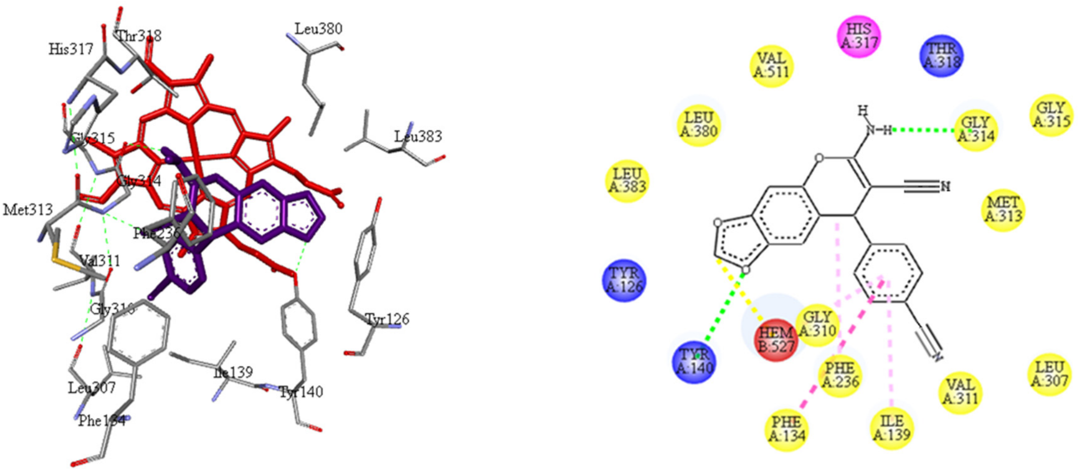
His317
Thr318
Leu380
Leu383
Gly315
Met313
Tyr126
Ile139
Tyr140
Leu307
HIS A:317
VAL A:511
THR A:318
LEU A:380
GLY A:314
GLY A:315
LEU A:383
MET A:313
TYR A:126
GLY A:310
HEM B:527
TYR A:140
PHE A:236
VAL A:311
LEU A:307
PHE A:134
ILE A:139

4f

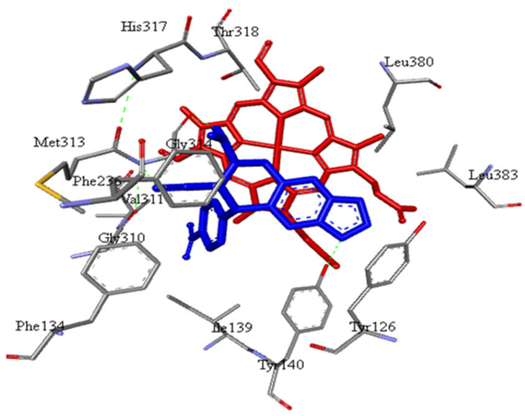
His317
Thr318
Leu380
Met313
Gly314
Leu383
Phe236
Val311
Gly310
Phe134
Ile139
Tyr126
Tyr140

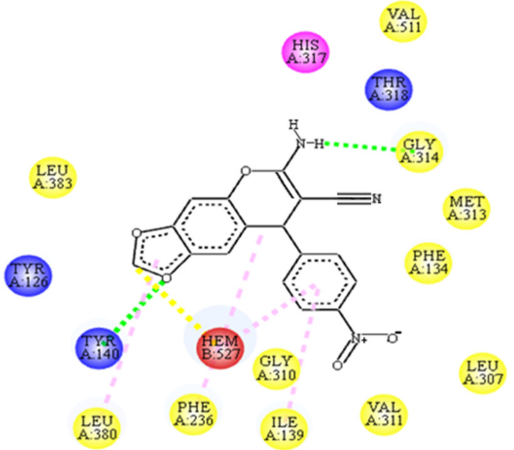
VAL A:511
HIS A:317
THR A:318
GLY A:314
LEU A:383
MET A:313
TYR A:126
PHE A:134
TYR A:140
HEM B:527
GLY A:310
LEU A:307
VAL A:311
LEU A:380
PHE A:236
ILE A:139

4g

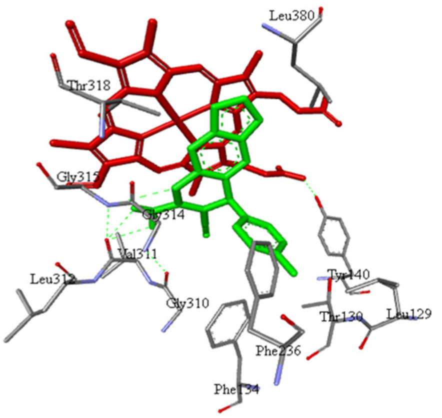
Leu380
Thr318
Gly315
Gly314
Val311
Leu312
Tyr140
Gly310
Thr130
Leu129
Phe236
Phe134

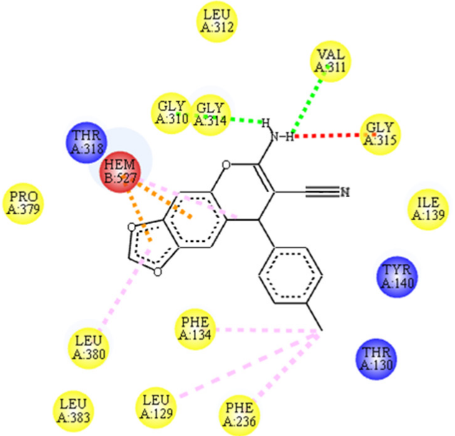
LEU A:312
VAL A:311
GLY A:310
GLY A:314
GLY A:315
THR A:318
HEM B:527
PRO A:379
ILE A:139
TYR A:140
PHE A:134
LEU A:380
THR A:130
LEU A:383
LEU A:129
PHE A:236

4h

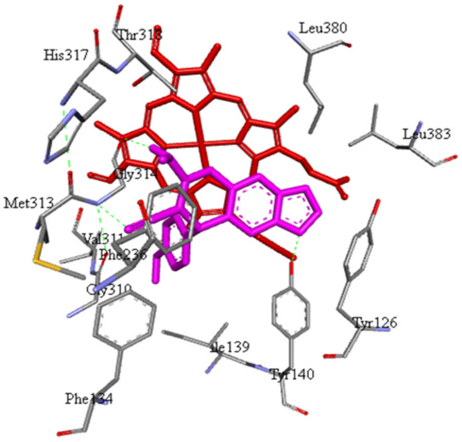
Thr318
Leu380
His317
Leu383
Gly314
Met313
Val311
Phe236
Gly310
Tyr126
Ile139
Tyr140
Phe134

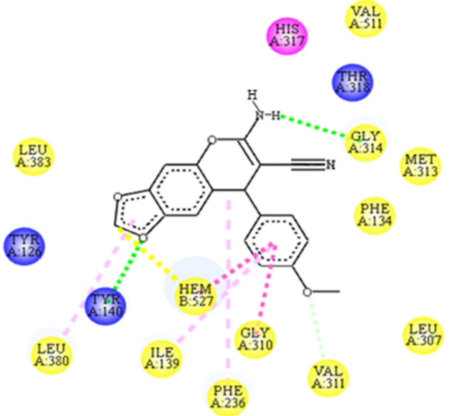
VAL A:511
HIS A:317
THR A:318
GLY A:314
LEU A:383
MET A:313
PHE A:134
TYR A:126
TYR A:140
HEM B:527
LEU A:380
ILE A:139
GLY A:310
LEU A:307
VAL A:311
PHE A:236

4i

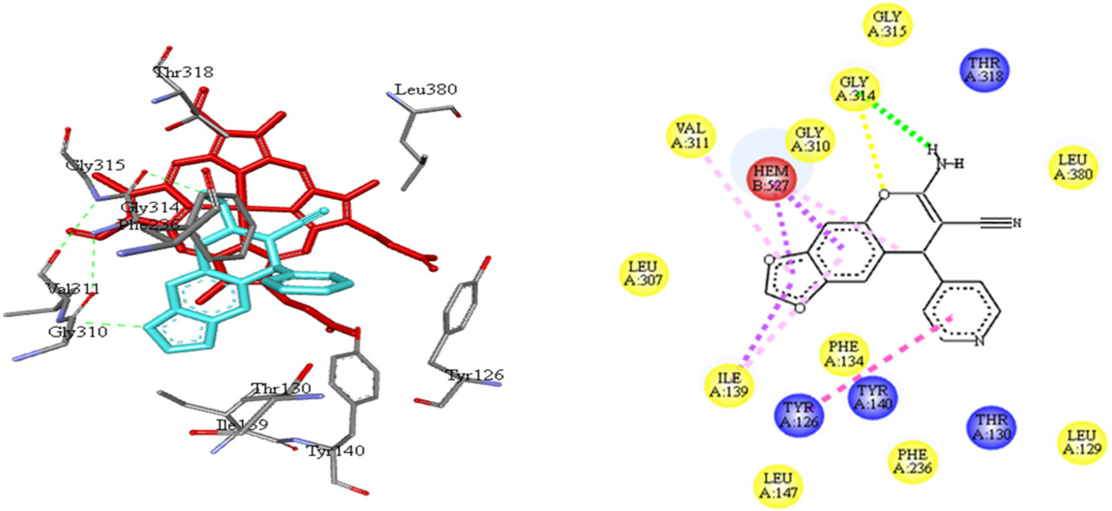

Figure S145. Schematic representation of binding mode between 2-amino-3-cyano-4-aryl-6,7-methylendioxy-4*H*-chromenes **4a**, **4c-i** and the reference compound fluconazole **8** at the active site of CYP51CKE (*C. kefyr*). Models 3D and 2D are shown. In 3D model hydrophilic bonds and amino acid residues that are part of the active site of CYP51 are represented. In the 2D model, the following interactions are portrayed with dotted lines: conventional hydrogen bonds (green), carbon-hydrogen (yellow), π-cation (orange), π-sigma (purple), π-alkyl (pink), T-shaped π-π (fushia), and halogen (cyan). Heme group is in red. The solvent accessible surface is illustrated for the amino acid residues and ligands. The amino acids are denoted in pink (basic), yellow (hydrophobic) and blue (hydrophilic).

6b

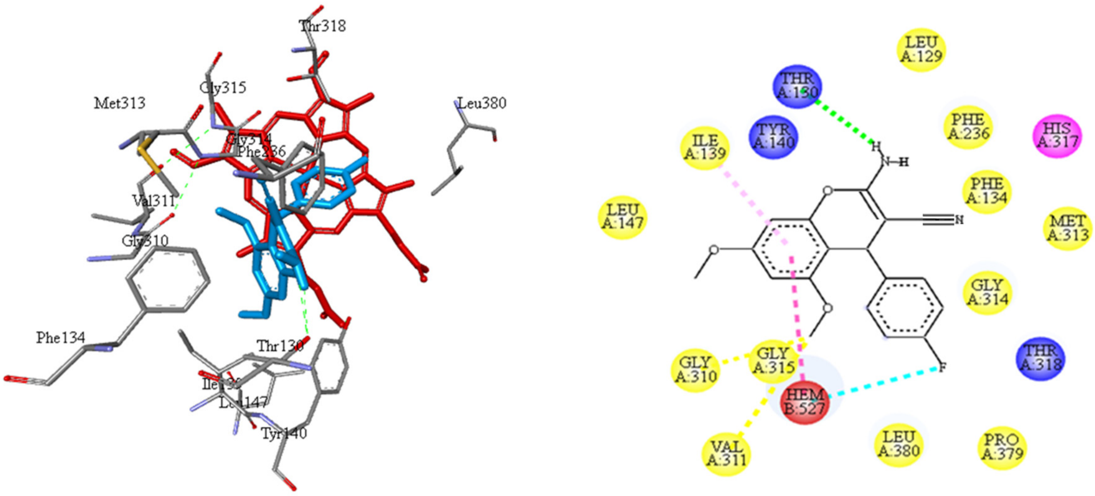

6c

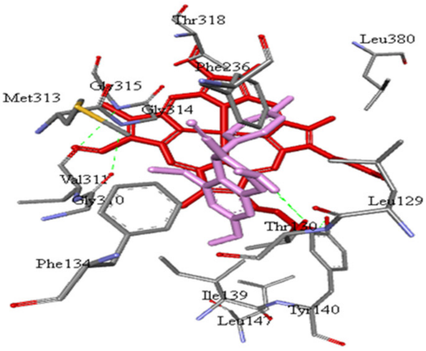
Thr318
Leu380
Phe236
Gly315
Met313
Gly314
Val311
Leu129
Phe134
Ile139
Tyr140
Leu147

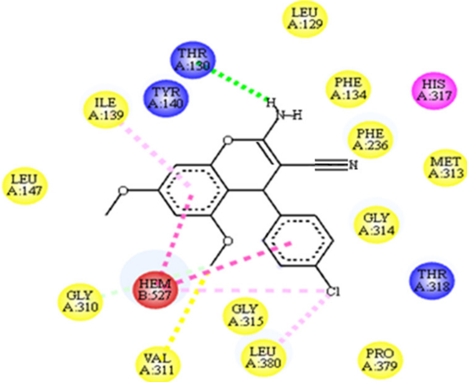
LEU A:129
THR A:130
TYR A:140
ILE A:139
PHE A:134
HIS A:317
PHE A:236
MET A:313
LEU A:147
GLY A:314
HEM B:527
GLY A:310
THR A:318
GLY A:315
VAL A:311
LEU A:380
PRO A:379
Cl

6d

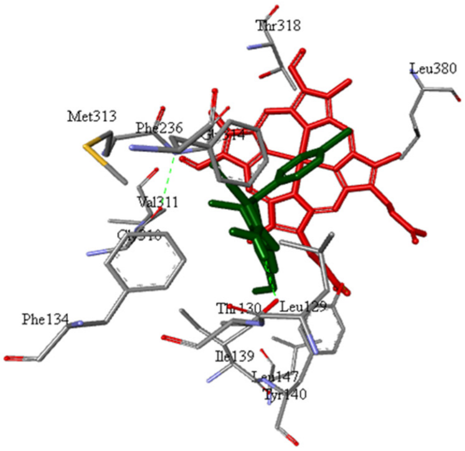
Thr318
Leu380
Met313
Phe236
Val311
Phe134
Thr130
Leu129
Ile139
Leu147
Tyr140

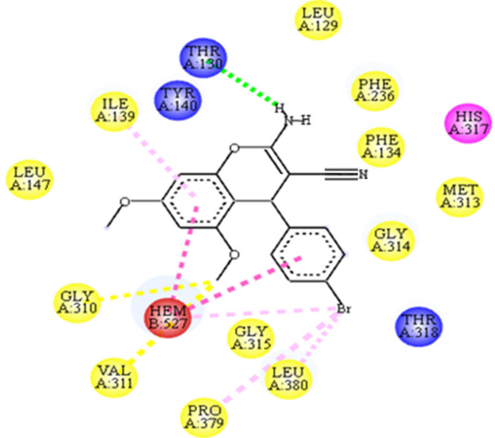
LEU A:129
THR A:130
TYR A:140
ILE A:139
PHE A:236
HIS A:317
PHE A:134
LEU A:147
MET A:313
GLY A:314
GLY A:310
HEM B:527
Br
THR A:318
GLY A:315
VAL A:311
LEU A:380
PRO A:379

6e

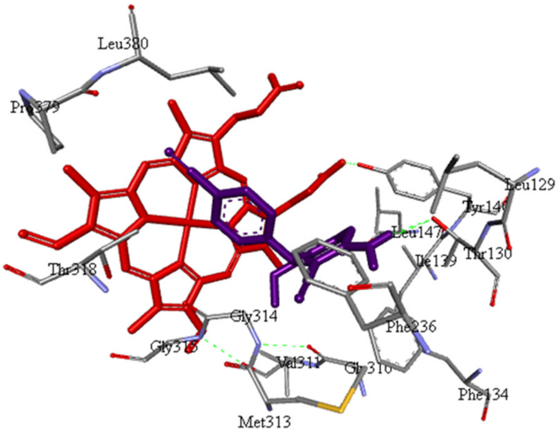
Leu380
Leu129
Thr318
Thr130
Gly314
Phe236
Val311
Phe134
Met313

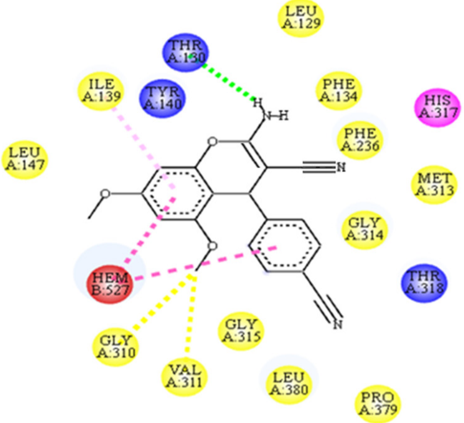
LEU A:129
THR A:130
ILE A:139
TYR A:140
PHE A:134
HIS A:317
PHE A:236
LEU A:147
MET A:313
GLY A:314
HEM B:527
THR A:318
GLY A:315
GLY A:310
VAL A:311
LEU A:380
PRO A:379

6f

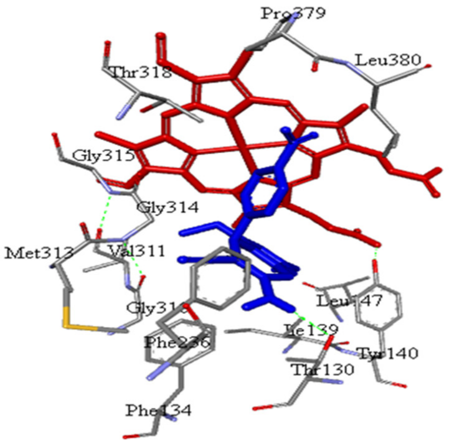

Pro379
Leu380
Thr318
Gly315
Gly314
Met313
Val311
Gly310
Leu147
Phe236
Ile139
Tyr140
Thr130
Phe134

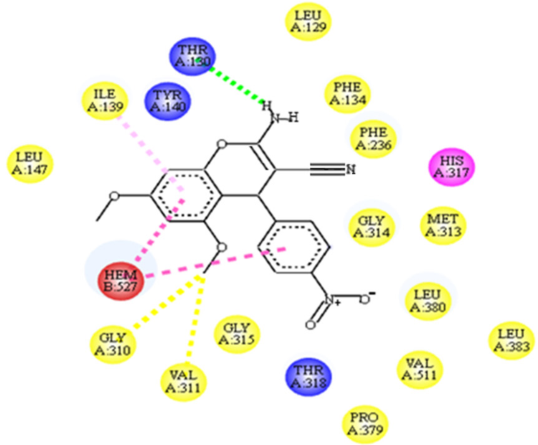

LEU A:129
THR A:130
PHE A:134
ILE A:139
TYR A:140
PHE A:236
HIS A:317
LEU A:147
GLY A:314
MET A:313
HEM B:527
LEU A:380
LEU A:383
GLY A:310
GLY A:315
VAL A:511
VAL A:311
THR A:318
PRO A:379

6g

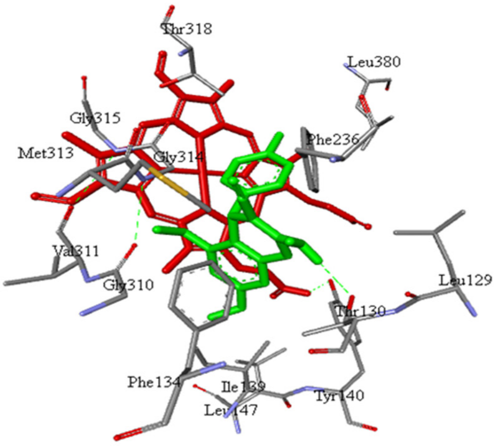

Thr318
Leu380
Gly315
Met313
Gly314
Phe236
Val311
Gly310
Leu129
Thr130
Phe134
Ile139
Leu147
Tyr140

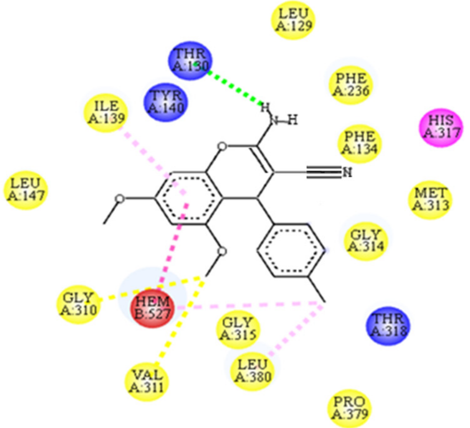

LEU A:129
THR A:130
PHE A:236
ILE A:139
TYR A:140
HIS A:317
PHE A:134
LEU A:147
MET A:313
GLY A:314
GLY A:310
HEM B:527
THR A:318
GLY A:315
VAL A:311
LEU A:380
PRO A:379

6h

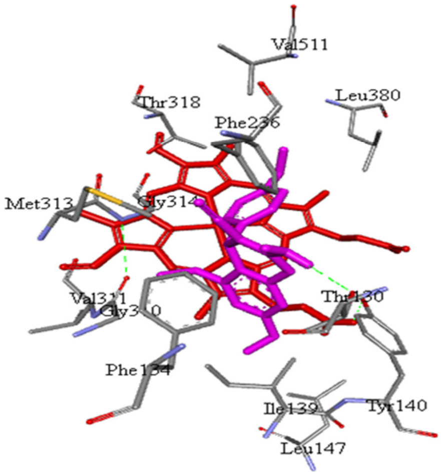

Val511
Leu380
Thr318
Phe236
Met313
Gly314
Val311
Thr130
Phe134
Ile139
Tyr140
Leu147

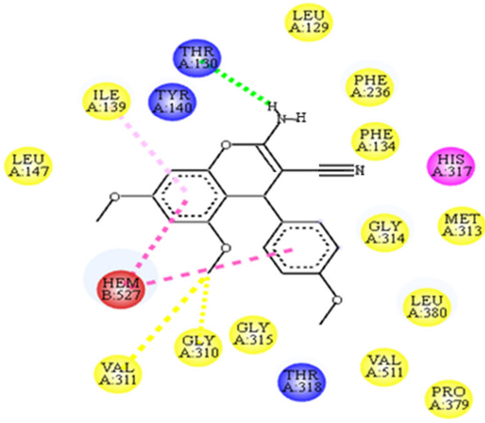

LEU A:129
THR A:130
PHE A:236
ILE A:139
TYR A:140
PHE A:134
HIS A:317
LEU A:147
GLY A:314
MET A:313
HEM B:527
LEU A:380
GLY A:310
GLY A:315
VAL A:311
VAL A:511
THR A:318
PRO A:379

Figure S146. Schematic representation of binding mode between 2-amino-3-cyano-5,7-dimethoxy-4-aryl-4*H*-chromenes **6b-h** at the active site of CYP51CKE (*C. kefyr*). Models 3D and 2D are shown. In 3D model hydrophilic bonds and amino acid residues that are part of the active site of CYP51 are represented. In the 2D model, the following interactions are portrayed with dotted lines: conventional hydrogen bonds (green), carbon-hydrogen (yellow), π-cation (orange), π-sigma (purple), π-alkyl (pink), T-shaped π-π (fushia), and halogen (cyan). Heme group is in red. The solvent accessible surface is illustrated for the amino acid residues and ligands. The amino acids are denoted in pink (basic), yellow (hydrophobic) and blue (hydrophilic).

Fluconazole **8**

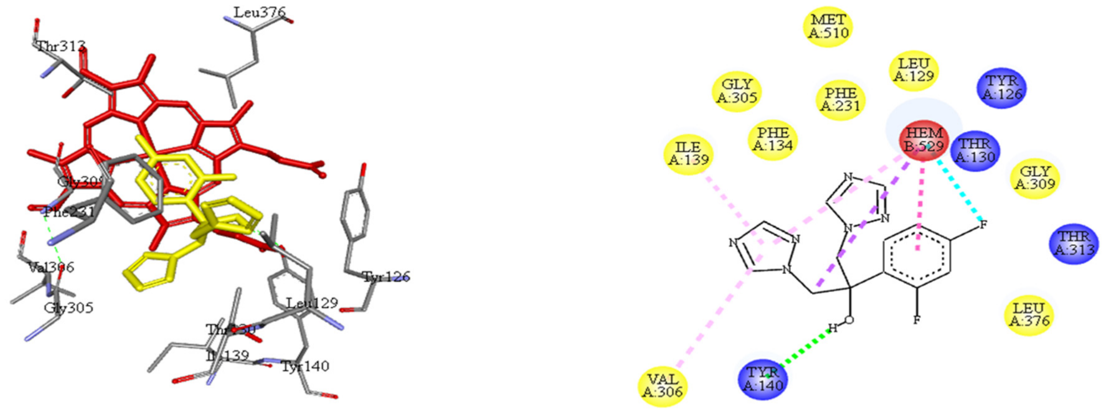

4a

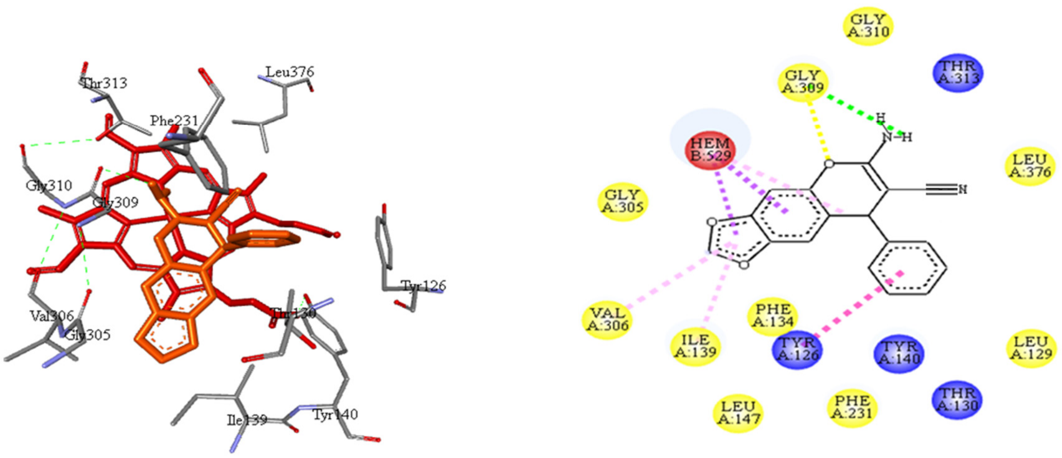

4b

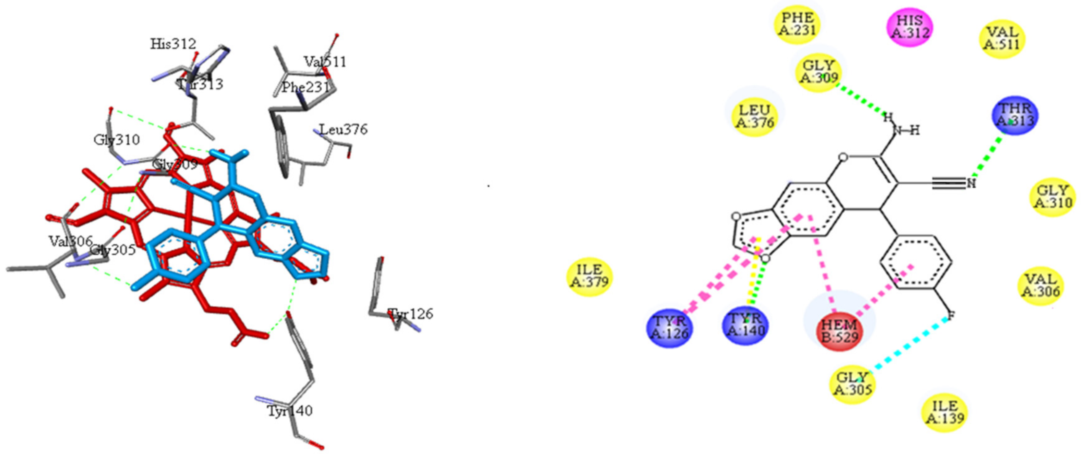
His312
Val511
Thr313
Phe231
Leu376
Gly310
Gly309
Val306
Gly305
Tyr126
Tyr140
PHE A:231
HIS A:312
VAL A:511
GLY A:309
LEU A:376
THR A:313
GLY A:310
ILE A:379
VAL A:306
TYR A:126
TYR A:140
HEM B:529
GLY A:305
ILE A:139
N–H
N
F

4c

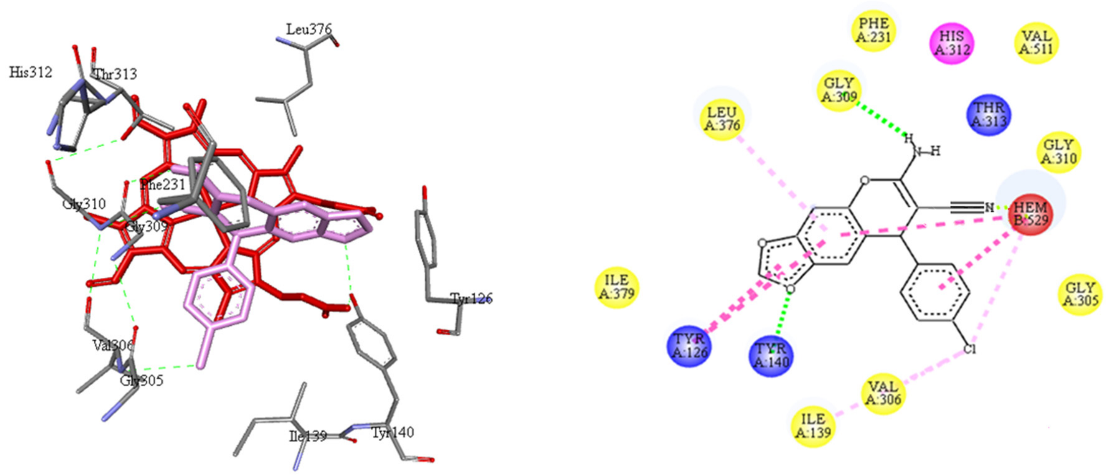
Leu376
His312
Thr313
Phe231
Gly310
Gly309
Tyr126
Val306
Gly305
Ile139
Tyr140
PHE A:231
HIS A:312
VAL A:511
GLY A:309
LEU A:376
THR A:313
GLY A:310
HEM B:529
ILE A:379
GLY A:305
TYR A:126
TYR A:140
VAL A:306
ILE A:139
N–H
N
Cl

4d

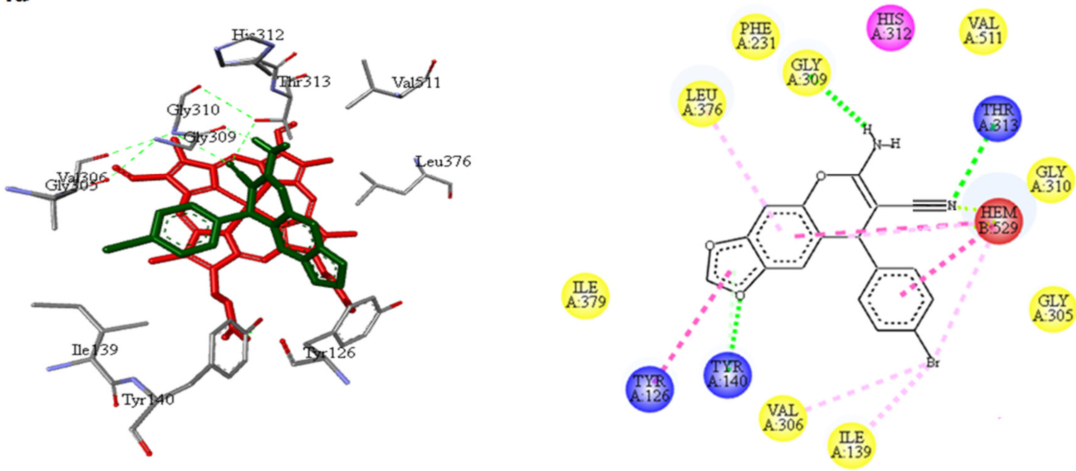
His312
Thr313
Val511
Gly310
Gly309
Val306
Gly305
Leu376
Ile139
Tyr126
Tyr140
PHE A:231
HIS A:312
VAL A:511
GLY A:309
LEU A:376
THR A:313
GLY A:310
HEM B:529
ILE A:379
GLY A:305
TYR A:126
TYR A:140
VAL A:306
ILE A:139
N–H
N
Br

4e

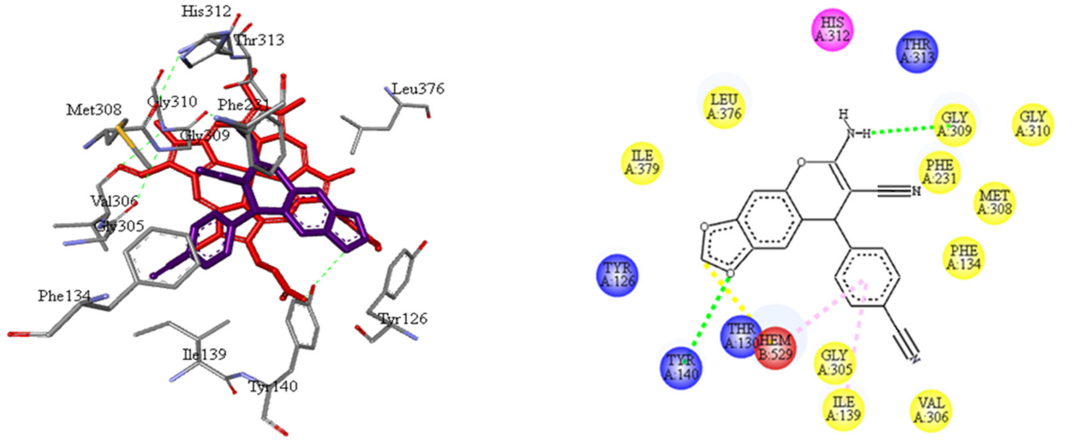
His312
Thr313
Leu376
Met308
Gly310
Phe231
Gly309
Val306
Gly305
Phe134
Tyr126
Ile139
Tyr140
HIS A:312
THR A:313
LEU A:376
GLY A:309
GLY A:310
ILE A:379
PHE A:231
MET A:308
TYR A:126
PHE A:134
THR A:130
HEM B:529
TYR A:140
GLY A:305
ILE A:139
VAL A:306

4f

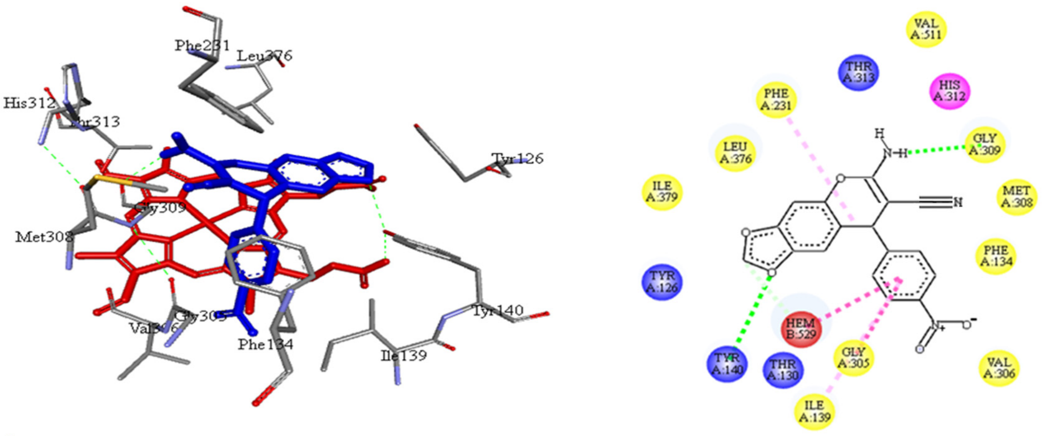
Phe231
Leu376
His312
Thr313
Tyr126
Gly309
Met308
Tyr140
Val306
Gly305
Phe134
Ile139
VAL A:511
THR A:313
HIS A:312
PHE A:231
GLY A:309
LEU A:376
ILE A:379
MET A:308
PHE A:134
TYR A:126
HEM B:529
TYR A:140
THR A:130
GLY A:305
VAL A:306
ILE A:139

4g

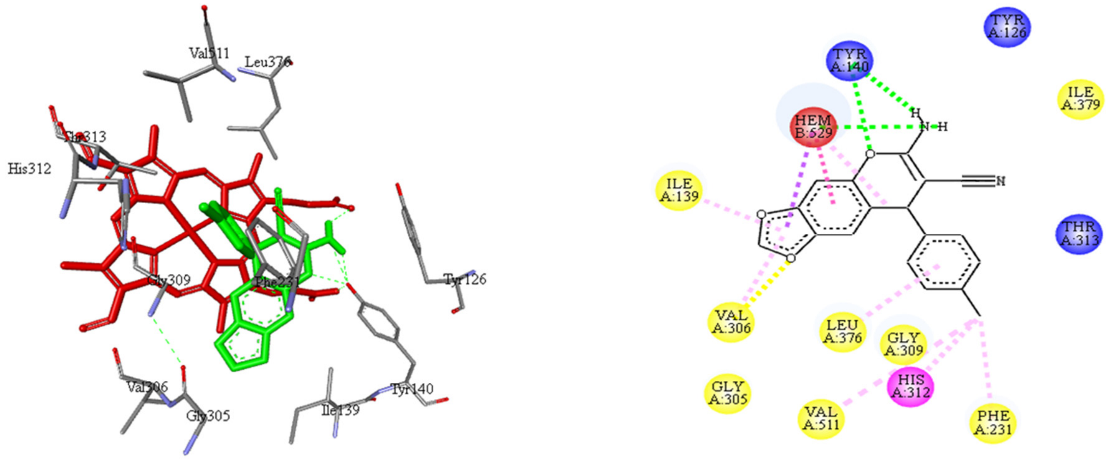
Val511
Leu376
Thr313
His312
Gly309
Phe231
Tyr126
Val306
Gly305
Ile139
Tyr140
TYR A:126
TYR A:140
ILE A:379
HEM B:529
ILE A:139
THR A:313
VAL A:306
LEU A:376
GLY A:309
GLY A:305
HIS A:312
VAL A:511
PHE A:231

4h

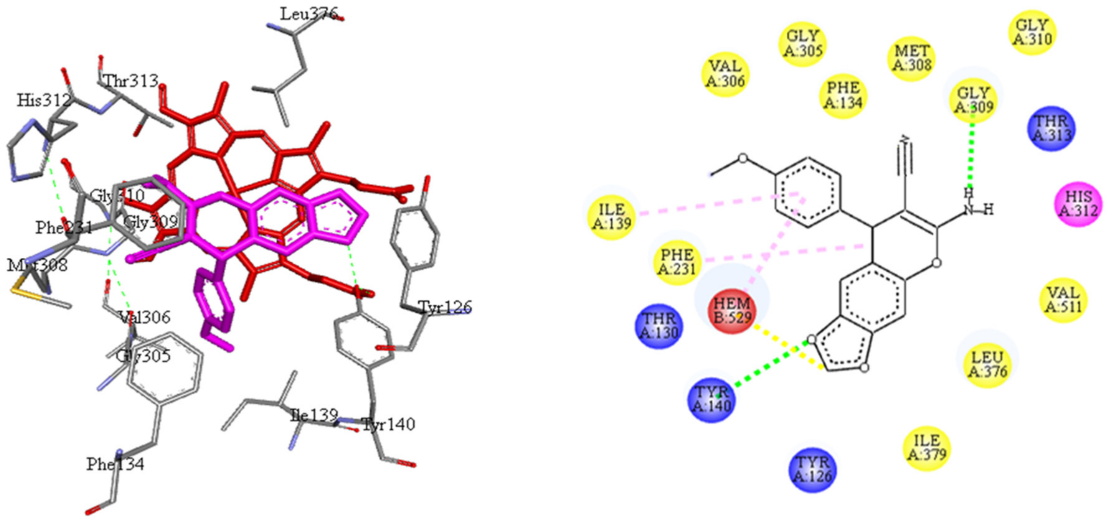

4i

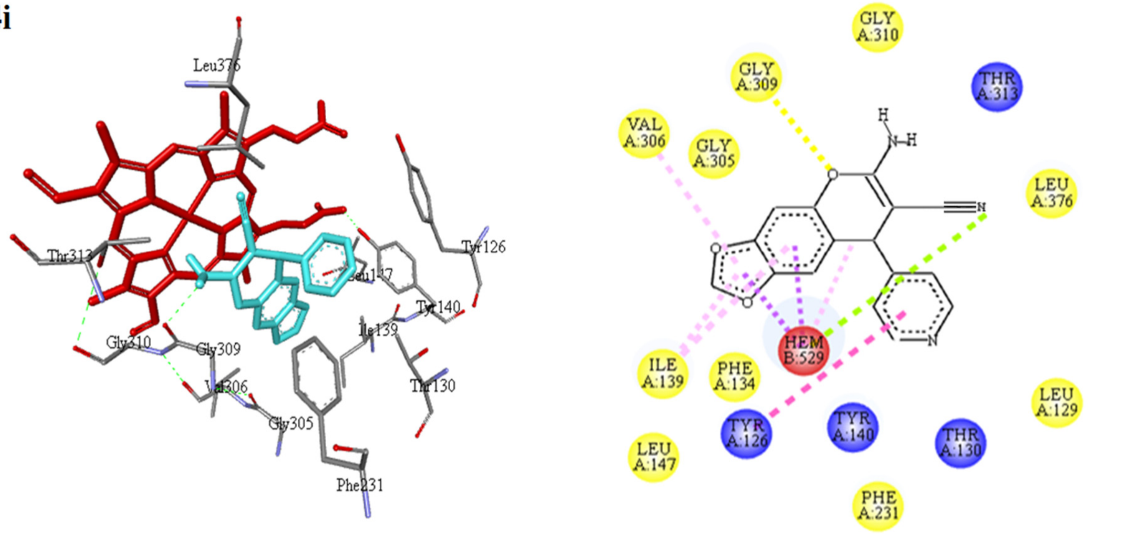

Figure S147. Schematic representation of mode of binding between 2-amino-3-cyano-4-aryl-6,7-methylendioxy-4*H*-chromenes **4a-i** and reference compound fluconazole **8** at the active site of CYP51CK (*C. krusei*). Models 3D and 2D are shown. In 3D model hydrophilic bonds and amino acid residues that are part of the active site of CYP51 are represented. In the 2D model, the following interactions are portrayed with dotted lines: conventional hydrogen bonds (green), carbon-hydrogen (yellow), π-cation (orange), π-sigma (purple), π-alkyl (pink), T-shaped π-π (fushia), and halogen (cyan). Heme group is in red. The solvent accessible surface is illustrated for the amino acid residues and ligands. The amino acids are

denoted in pink (basic), yellow (hydrophobic) and blue (hydrophilic).

6a

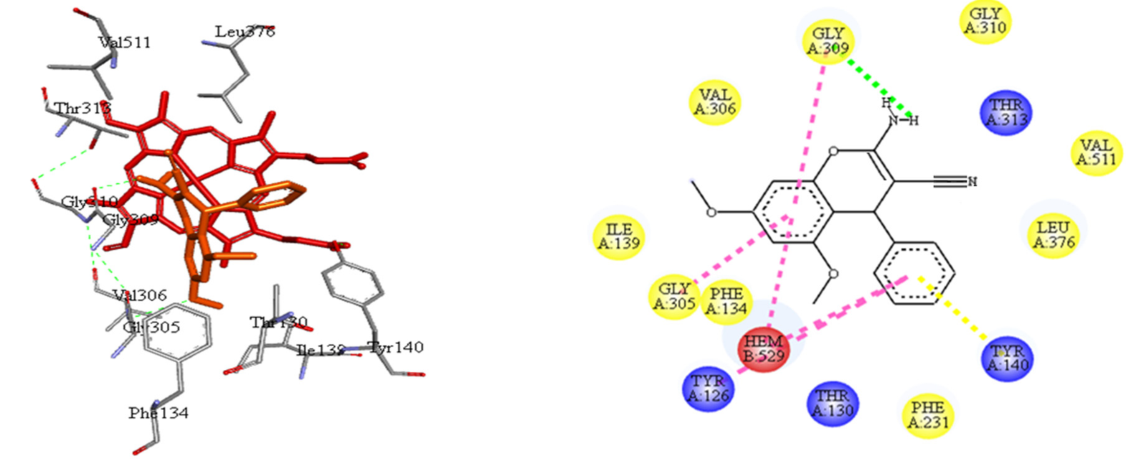

6b

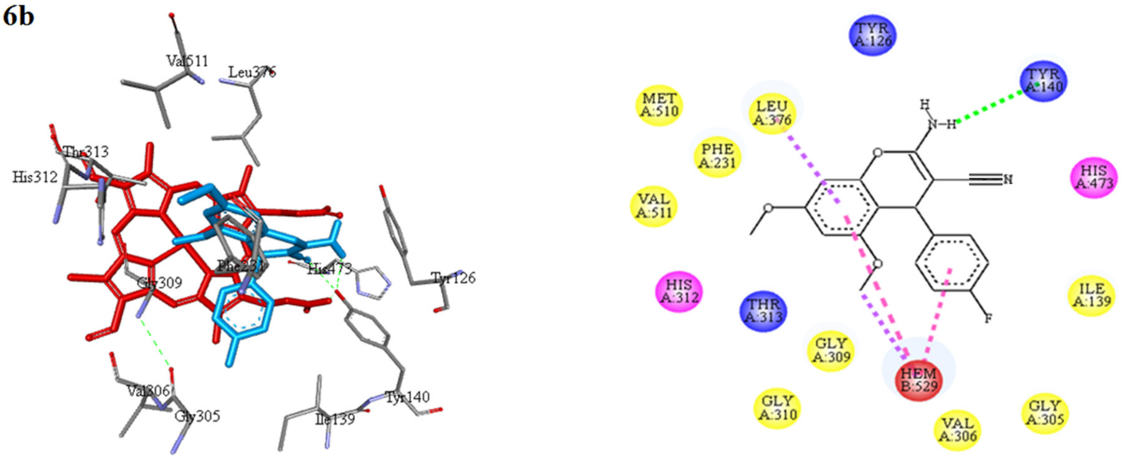

6c

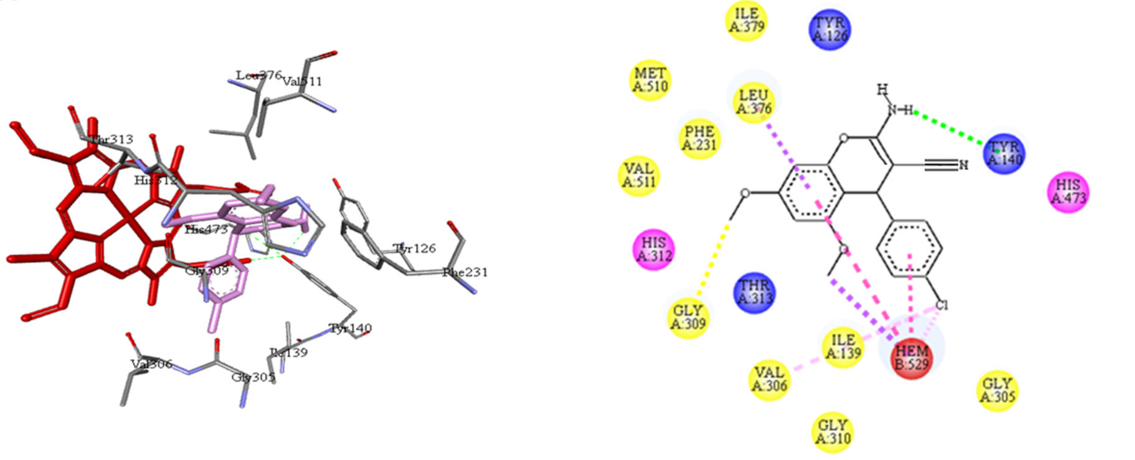

6d

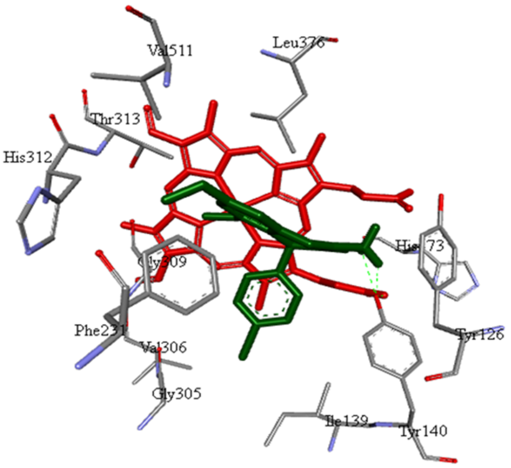
Val511
Leu376
Thr313
His312
Gly309
His473
Phe231
Val306
Tyr126
Gly305
Ile139
Tyr140

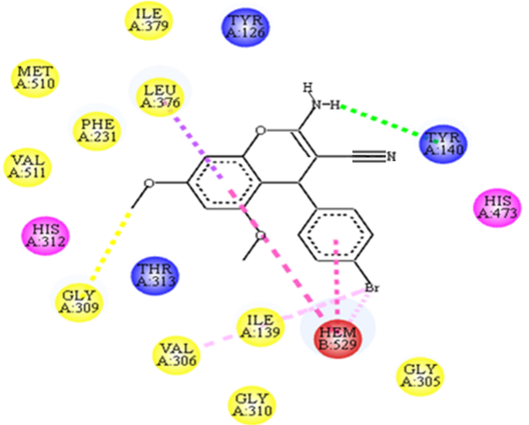
ILE A:379
TYR A:126
MET A:510
LEU A:376
PHE A:231
TYR A:140
VAL A:511
HIS A:473
HIS A:312
THR A:313
GLY A:309
ILE A:139
HEM B:529
VAL A:306
GLY A:305
GLY A:310

6e

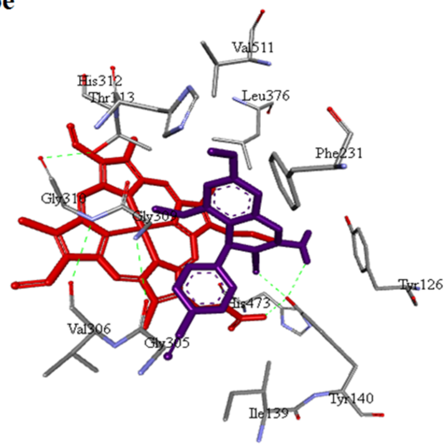
Val511
His312
Thr313
Leu376
Phe231
Tyr126
His473
Val306
Gly305
Ile139
Tyr140

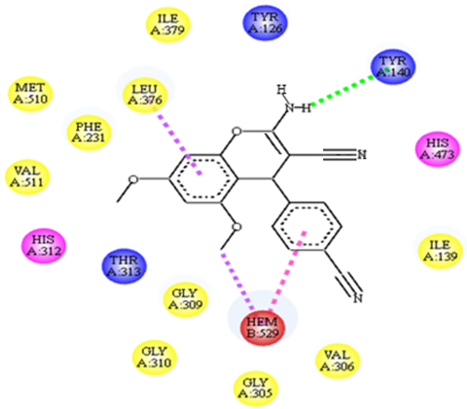
ILE A:379
TYR A:126
TYR A:140
MET A:510
LEU A:376
PHE A:231
HIS A:473
VAL A:511
HIS A:312
THR A:313
ILE A:139
GLY A:309
HEM B:529
GLY A:310
VAL A:306
GLY A:305

6f

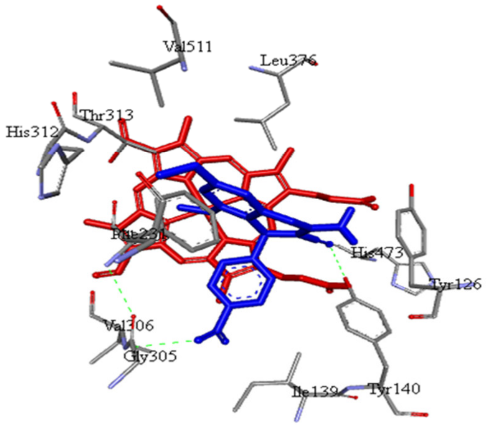
Val511
Leu376
Thr313
His312
His473
Tyr126
Val306
Gly305
Ile139
Tyr140

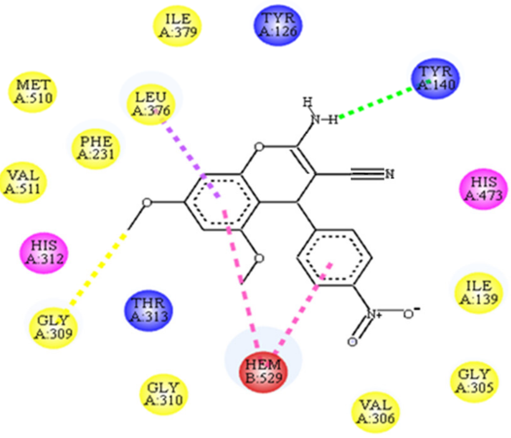
ILE A:379
TYR A:126
TYR A:140
MET A:510
LEU A:376
PHE A:231
VAL A:511
HIS A:473
HIS A:312
ILE A:139
GLY A:309
THR A:313
HEM B:529
GLY A:305
GLY A:310
VAL A:306

6g

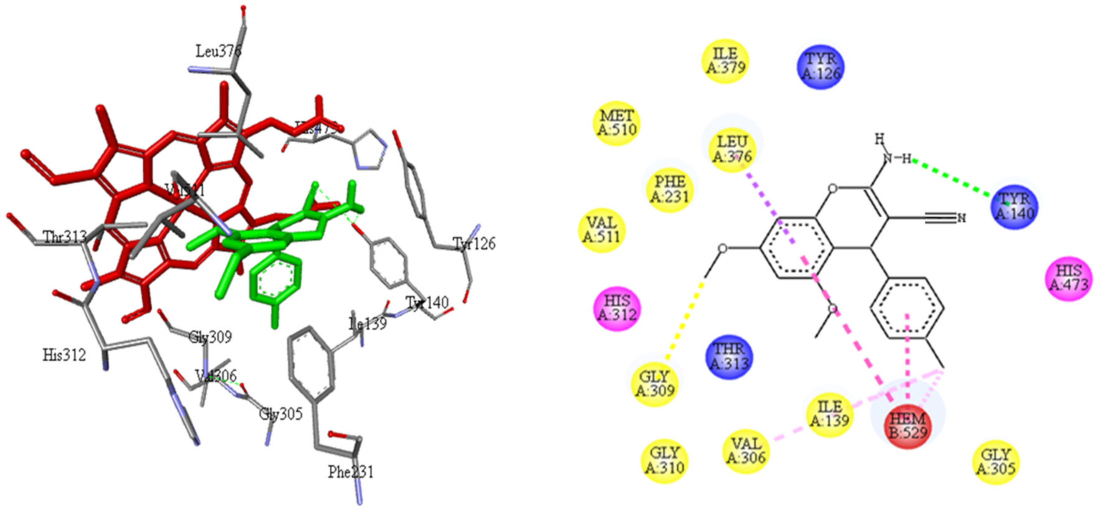

6h

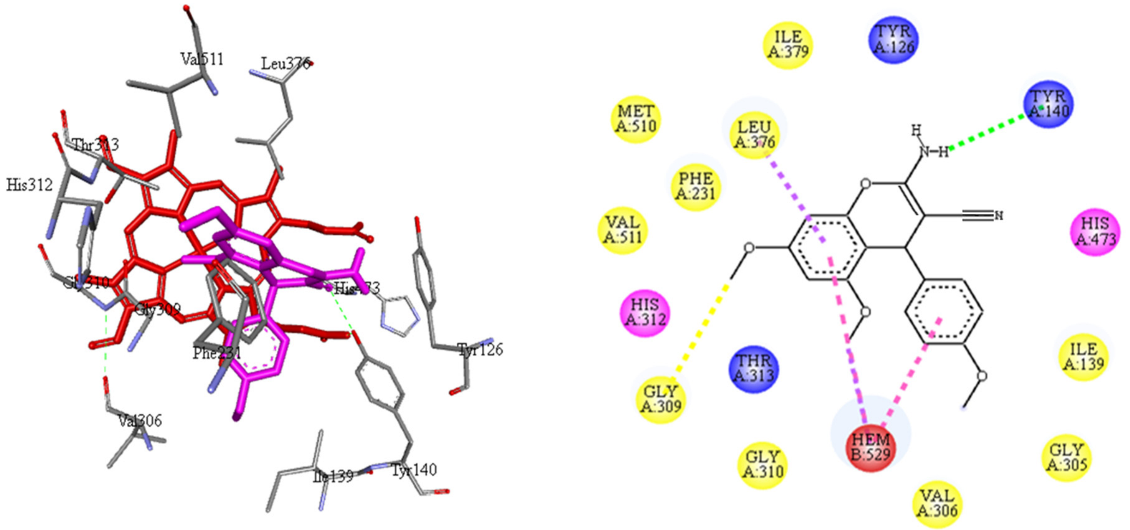

Figure S148. Schematic representation of mode of binding between 2-amino-3-cyano-5,7-dimethoxy-4-aryl-4*H*-chromenes **6a-h** at the active site of CYP51CK (*C. krusei*). Models 3D and 2D are shown. In 3D model hydrophilic bonds and amino acid residues that are part of the active site of CYP51 are represented. In the 2D model, the following interactions are portrayed with dotted lines: conventional hydrogen bonds (green), carbon-hydrogen (yellow), π-cation (orange), π-sigma (purple), π-alkyl (pink), T-shaped π-π (fushia), and halogen (cyan). Heme group is in red. The solvent accessible surface is illustrated for the

amino acid residues and ligands. The amino acids are denoted in pink (basic), yellow (hydrophobic) and blue (hydrophilic).

Fluconazole **8**

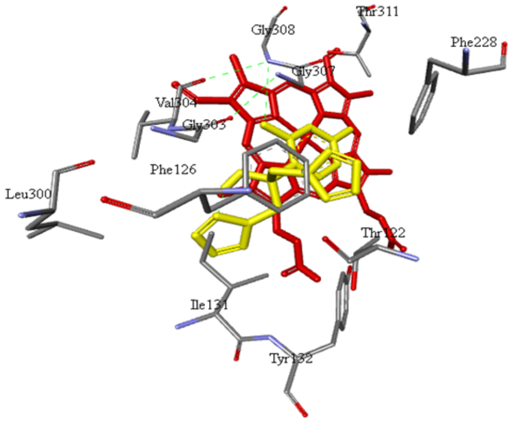

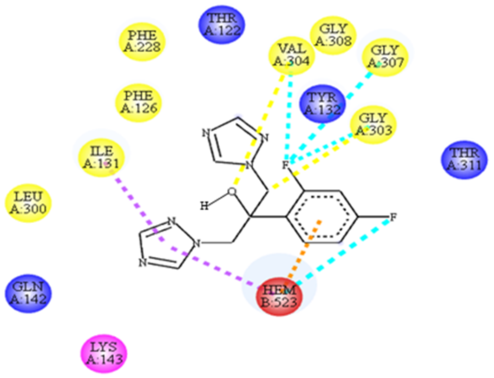

4a

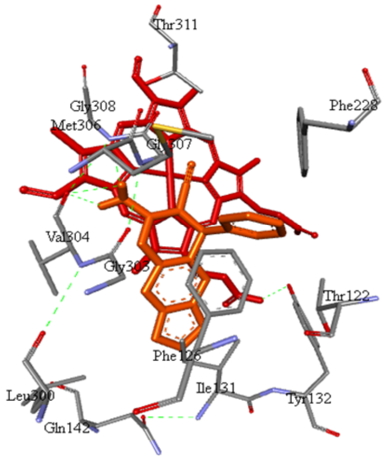

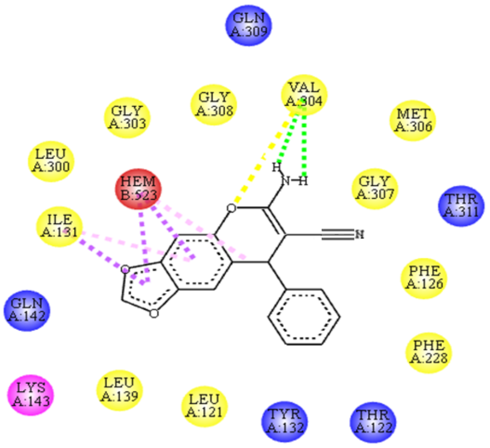

4b

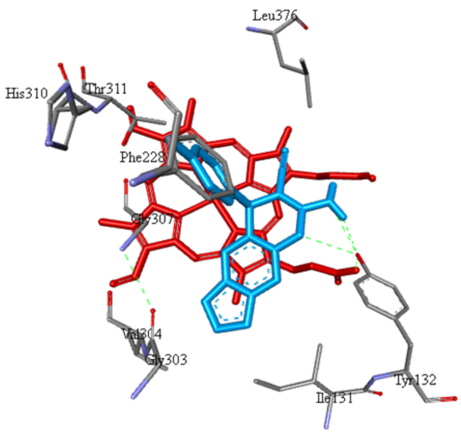

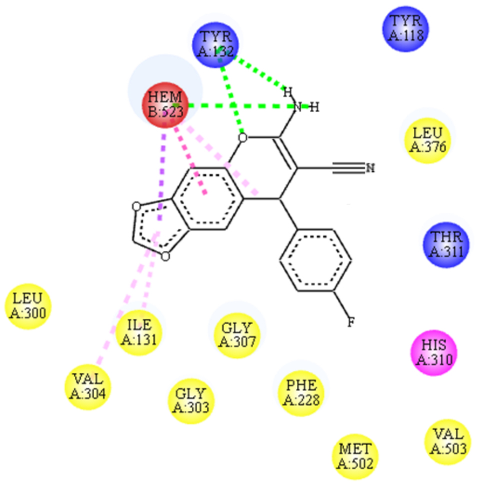

4c

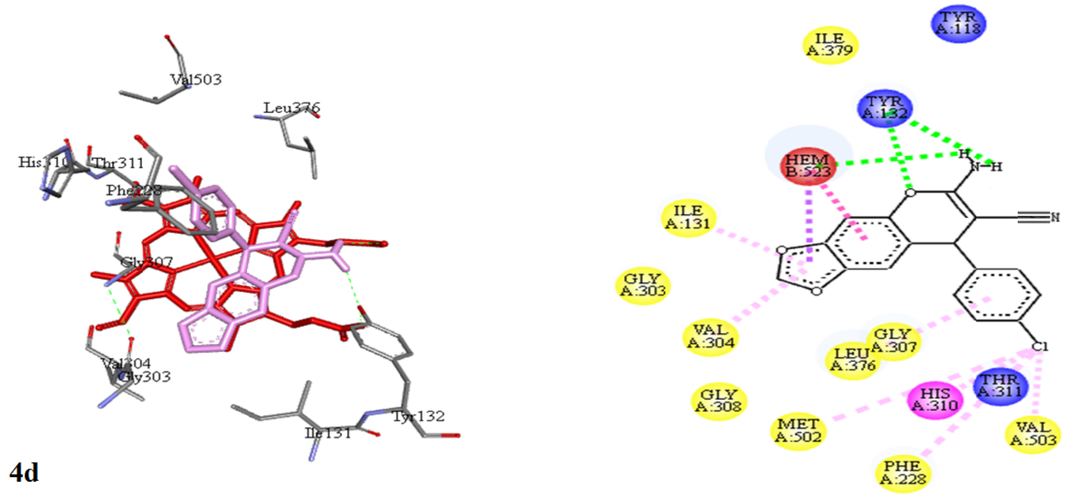

Val503
Leu376
His310
Thr311
Phe228
Gly307
Val304
Gly303
Ile131
Tyr132
ILE A:379
TYR A:118
TYR A:132
HEM B:523
ILE A:131
GLY A:303
VAL A:304
GLY A:307
LEU A:376
HIS A:310
THR A:311
GLY A:308
MET A:502
VAL A:503
PHE A:228

4d

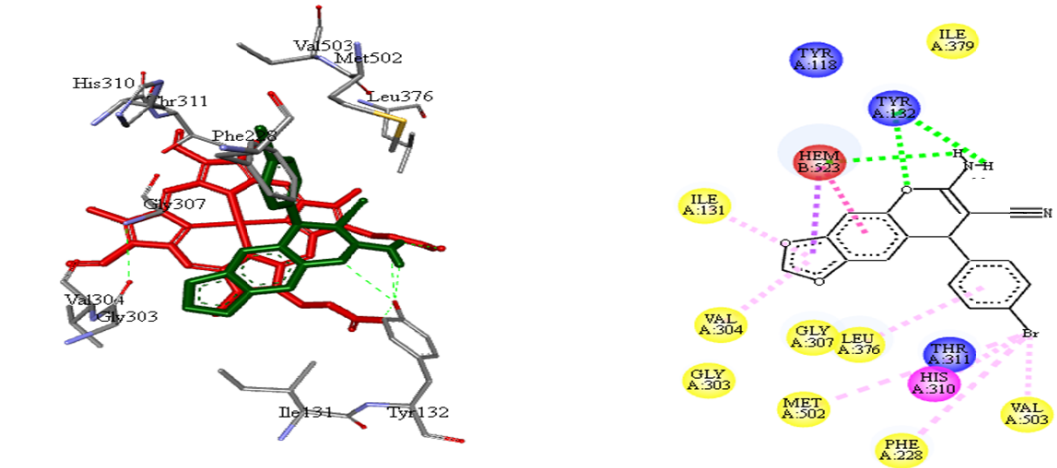

Val503
Met502
His310
Thr311
Leu376
Phe228
Gly307
Val304
Gly303
Ile131
Tyr132
TYR A:118
ILE A:379
TYR A:132
HEM B:523
ILE A:131
VAL A:304
GLY A:307
LEU A:376
THR A:311
HIS A:310
GLY A:303
MET A:502
VAL A:503
PHE A:228

4e

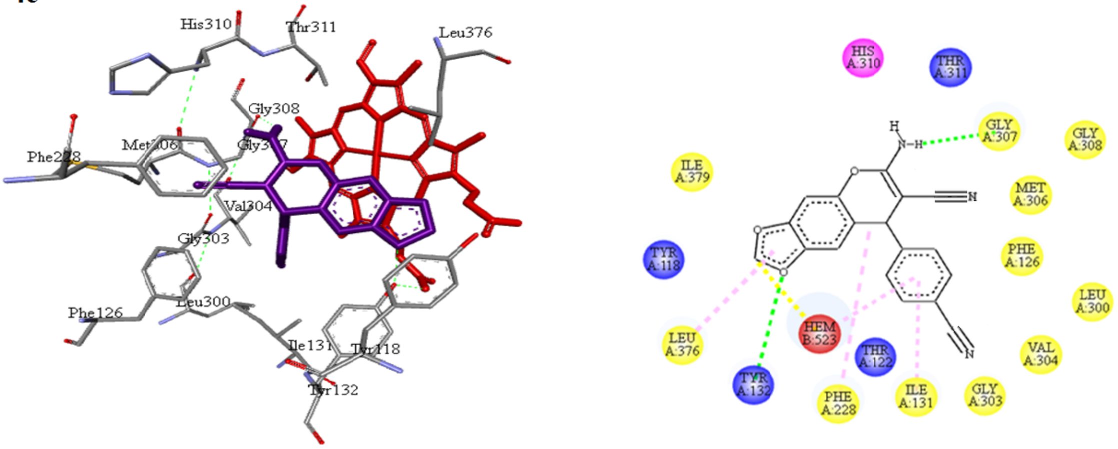

His310
Thr311
Leu376
Gly308
Phe228
Val304
Gly303
Phe126
Leu300
Ile131
Tyr118
Tyr132
HIS A:310
THR A:311
GLY A:307
GLY A:308
ILE A:379
MET A:306
TYR A:118
PHE A:126
LEU A:300
HEM B:523
LEU A:376
THR A:122
VAL A:304
TYR A:132
PHE A:228
ILE A:131
GLY A:303

4f

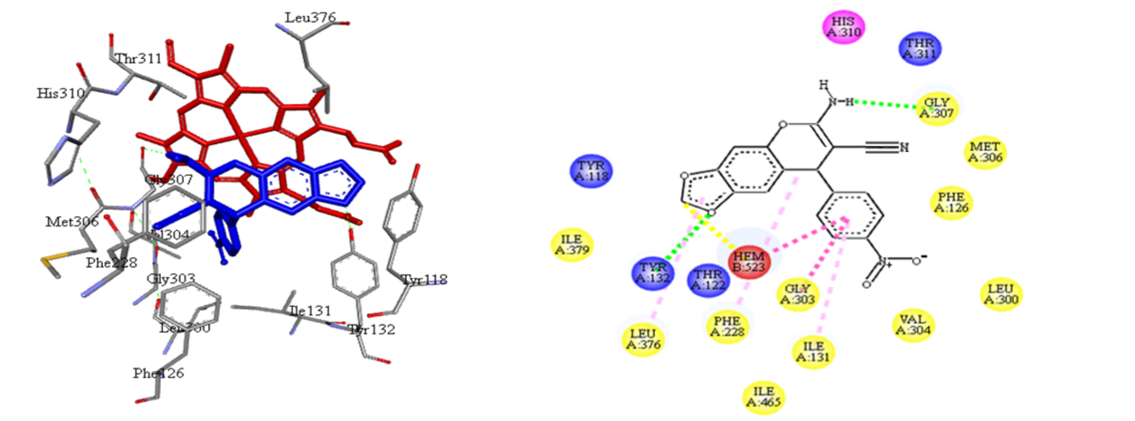
Leu376
Thr311
His310
Gly307
Met306
Val304
Phe228
Gly303
Tyr118
Ile131
Tyr132
HIS A:310
THR A:311
GLY A:307
MET A:306
TYR A:118
PHE A:126
ILE A:379
TYR A:132
THR A:122
HEM B:523
GLY A:303
LEU A:300
LEU A:376
PHE A:228
VAL A:304
ILE A:131
ILE A:465

4g

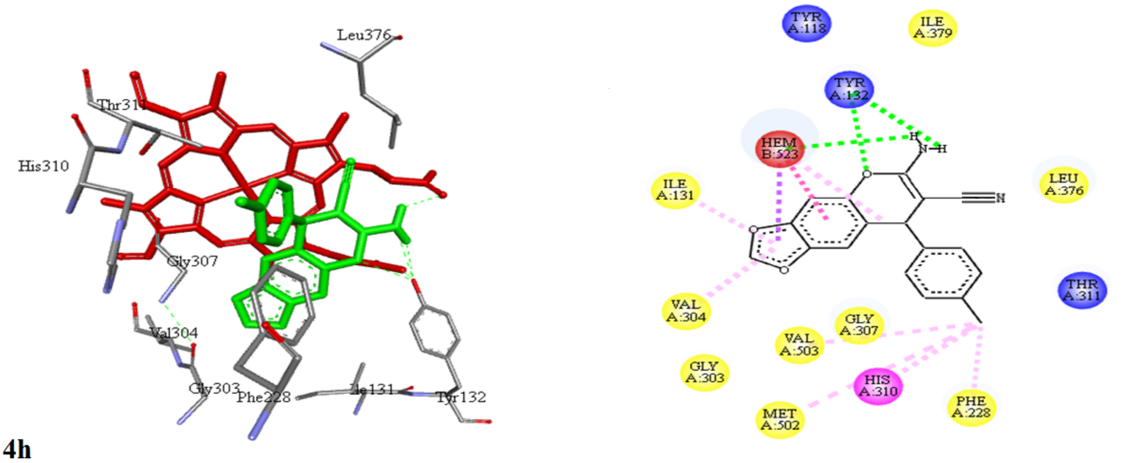
Leu376
His310
Gly307
Val304
Gly303
Phe228
Ile131
Tyr132
TYR A:118
ILE A:379
TYR A:132
HEM B:523
LEU A:376
ILE A:131
THR A:311
VAL A:304
GLY A:307
VAL A:503
GLY A:303
HIS A:310
PHE A:228
MET A:502

4h

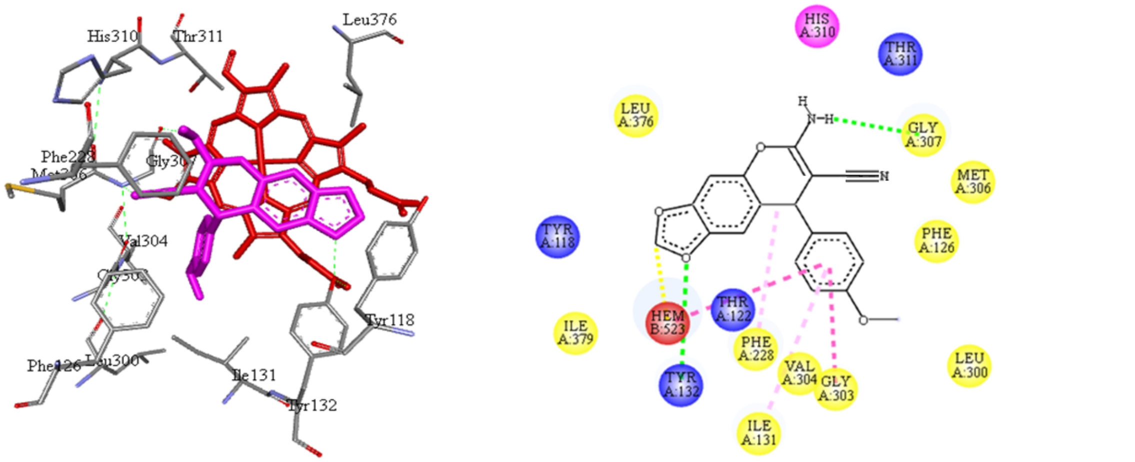
Leu376
His310
Thr311
Phe228
Gly307
Val304
Tyr118
Ile131
Tyr132
HIS A:310
THR A:311
LEU A:376
GLY A:307
MET A:306
TYR A:118
PHE A:126
HEM B:523
THR A:122
ILE A:379
PHE A:228
VAL A:304
GLY A:303
LEU A:300
TYR A:132
ILE A:131

4i

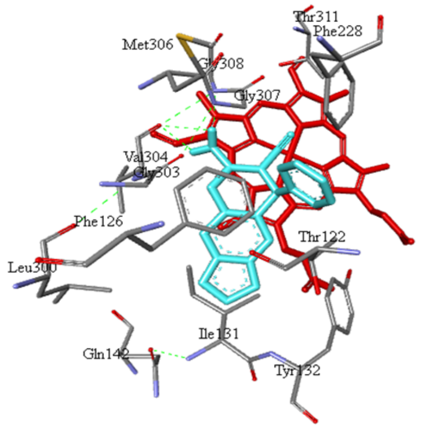

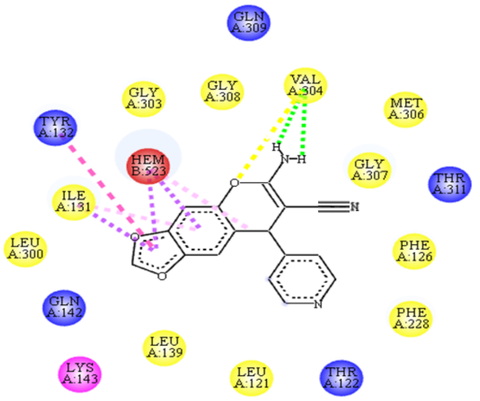

Figure S149. Schematic representation of mode of binding between 2-amino-3-cyano-4-aryl-6,7-methylendioxy-4*H*-chromenes **4a-i** and the reference compound fluconazole **8** at the active site of CYP51CP (*C. parapsilosis*). Models 3D and 2D are shown. In 3D model hydrophilic bonds and amino acid residues that are part of the active site of CYP51 are represented. In the 2D model, the following interactions are portrayed with dotted lines: conventional hydrogen bonds (green), carbon-hydrogen (yellow), π-cation (orange), π-sigma (purple), π-Alkyl (pink), T-shaped π-π (fushia), and halogen (cyan). Heme group is in red. The solvent accessible surface is illustrated for the amino acid residues and ligands. The amino acids are denoted in pink (basic), yellow (hydrophobic) and blue (hydrophilic).

6a

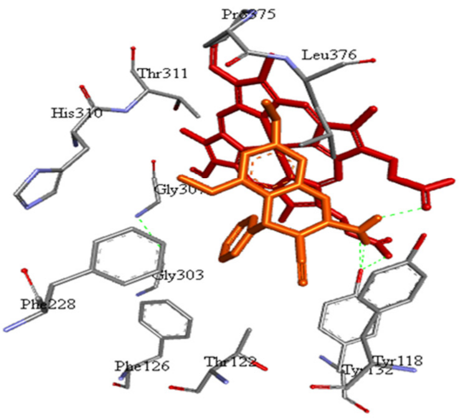

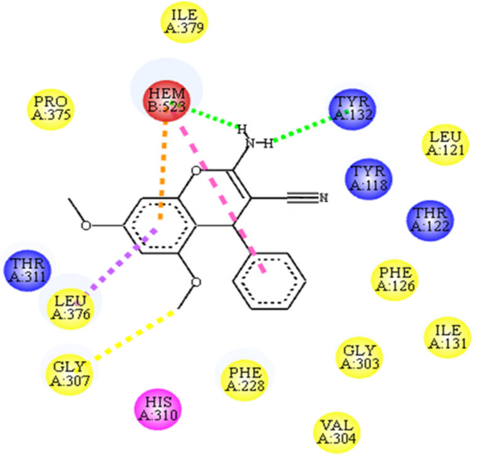

6b

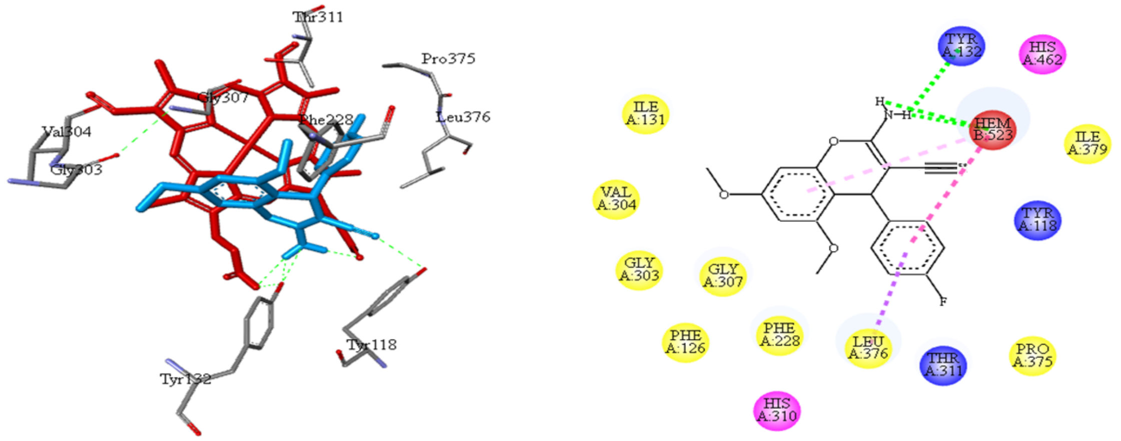
Thr311
Pro375
Gly307
Phe228
Leu376
Val304
Gly303
Tyr118
Tyr132
TYR A:132
HIS A:462
ILE A:131
HEM B:523
ILE A:379
VAL A:304
TYR A:118
GLY A:303
GLY A:307
F
PHE A:126
PHE A:228
LEU A:376
THR A:311
PRO A:375
HIS A:310

6c

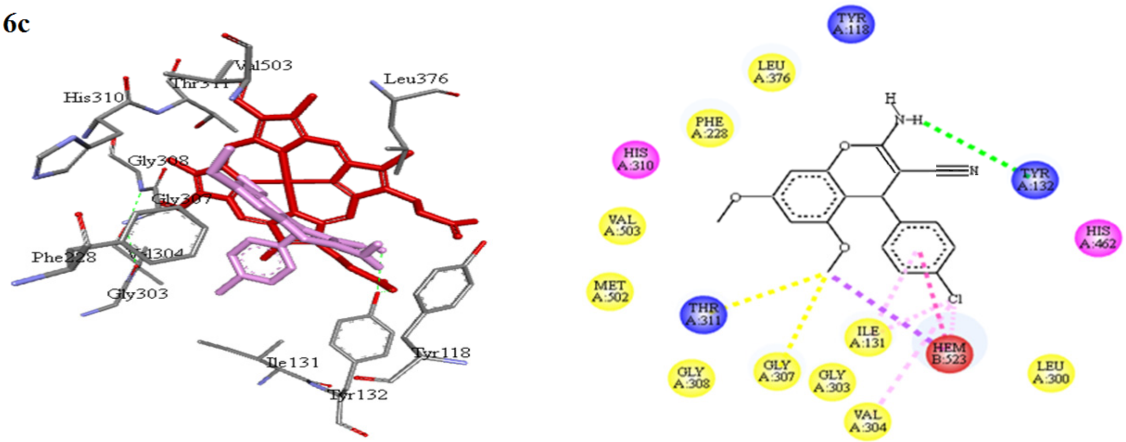
Val503
Leu376
His310
Gly308
Gly307
Phe228
Gly303
Ile131
Tyr118
Tyr132
TYR A:118
LEU A:376
PHE A:228
HIS A:310
TYR A:132
HIS A:462
VAL A:503
MET A:502
Cl
THR A:311
ILE A:131
HEM B:523
GLY A:308
GLY A:307
GLY A:303
LEU A:300
VAL A:304

6d

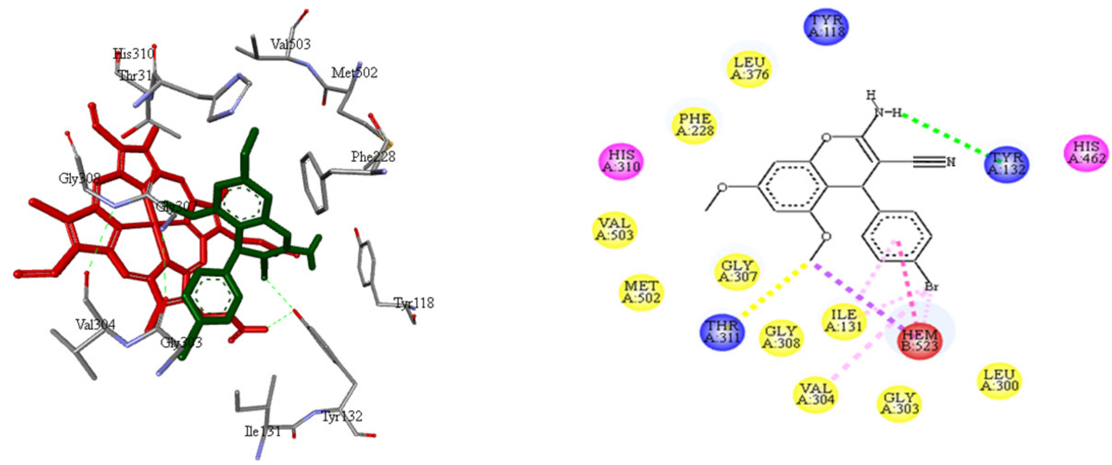
His310
Val503
Met502
Phe228
Tyr118
Val304
Tyr132
Ile131
TYR A:118
LEU A:376
PHE A:228
HIS A:310
TYR A:132
HIS A:462
VAL A:503
GLY A:307
MET A:502
Br
THR A:311
GLY A:308
ILE A:131
HEM B:523
VAL A:304
GLY A:303
LEU A:300

6e

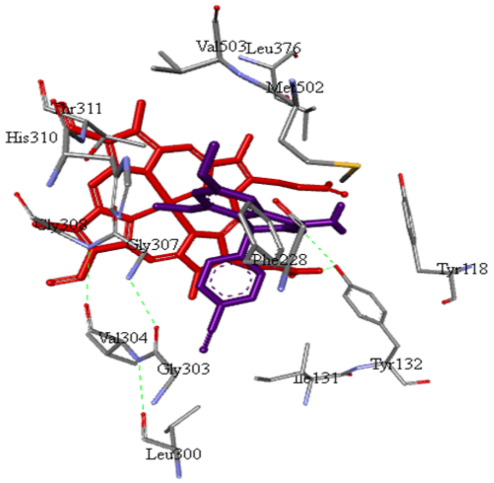
Val503
Leu376
Met502
Thr311
His310
Gly308
Gly307
Phe228
Tyr118
Val304
Gly303
Ile131
Tyr132
Leu300

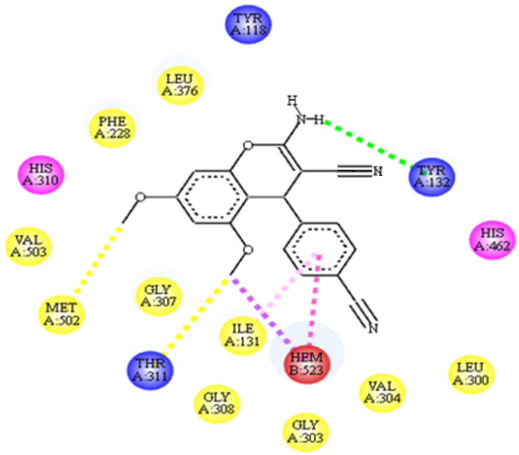
TYR A:118
LEU A:376
PHE A:228
HIS A:310
TYR A:132
HIS A:462
VAL A:503
GLY A:307
MET A:502
ILE A:131
HEM B:523
THR A:311
VAL A:304
LEU A:300
GLY A:308
GLY A:303

6f

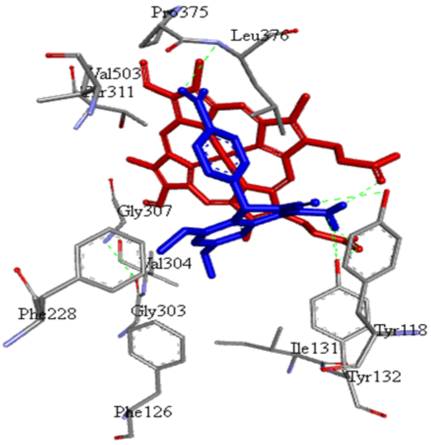
Pro375
Leu376
Val503
Thr311
Gly307
Val304
Phe228
Gly303
Tyr118
Ile131
Tyr132
Phe126

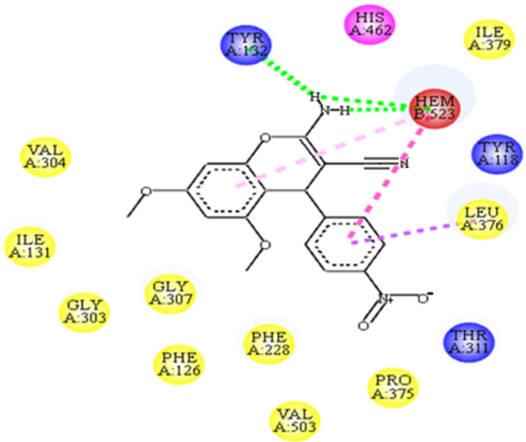
HIS A:462
TYR A:132
ILE A:379
HEM B:523
TYR A:118
VAL A:304
LEU A:376
ILE A:131
GLY A:307
GLY A:303
PHE A:228
THR A:311
PHE A:126
PRO A:375
VAL A:503

6g

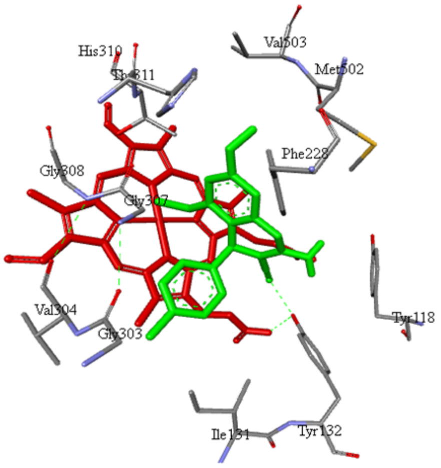
His310
Thr311
Val503
Met502
Phe228
Gly308
Gly307
Val304
Gly303
Tyr118
Ile131
Tyr132

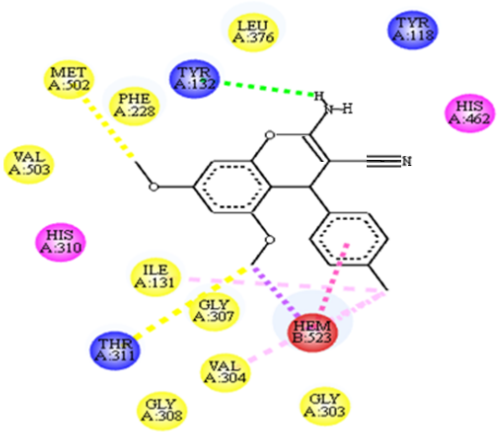
LEU A:376
TYR A:118
MET A:502
TYR A:132
PHE A:228
HIS A:462
VAL A:503
HIS A:310
ILE A:131
GLY A:307
THR A:311
HEM B:523
VAL A:304
GLY A:308
GLY A:303

6h

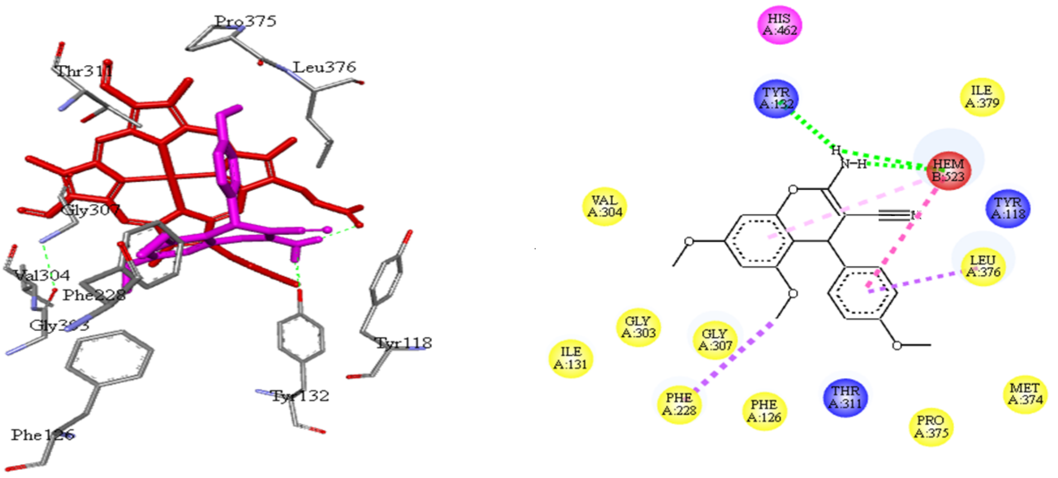

Figure S150. Schematic representation of mode of binding between 2-amino-3-cyano-5,7-dimethoxy-4-aryl-4*H*-chromenes **6a-h** at the active site of CYP51CP (*C. parapsilosis*). Models 3D and 2D are shown. In 3D model hydrophilic bonds and amino acid residues that are part of the active site of CYP51 are represented. In the 2D model, the following interactions are portrayed with dotted lines: conventional hydrogen bonds (green), carbon-hydrogen (yellow), π-cation (orange), π-sigma (purple), π-Alkyl (pink), T-shaped π-π (fushia), and halogen (cyan). Heme group is in red. The solvent accessible surface is illustrated for the amino acid residues and ligands. The amino acids are denoted in pink (basic), yellow (hydrophobic) and blue (hydrophilic).

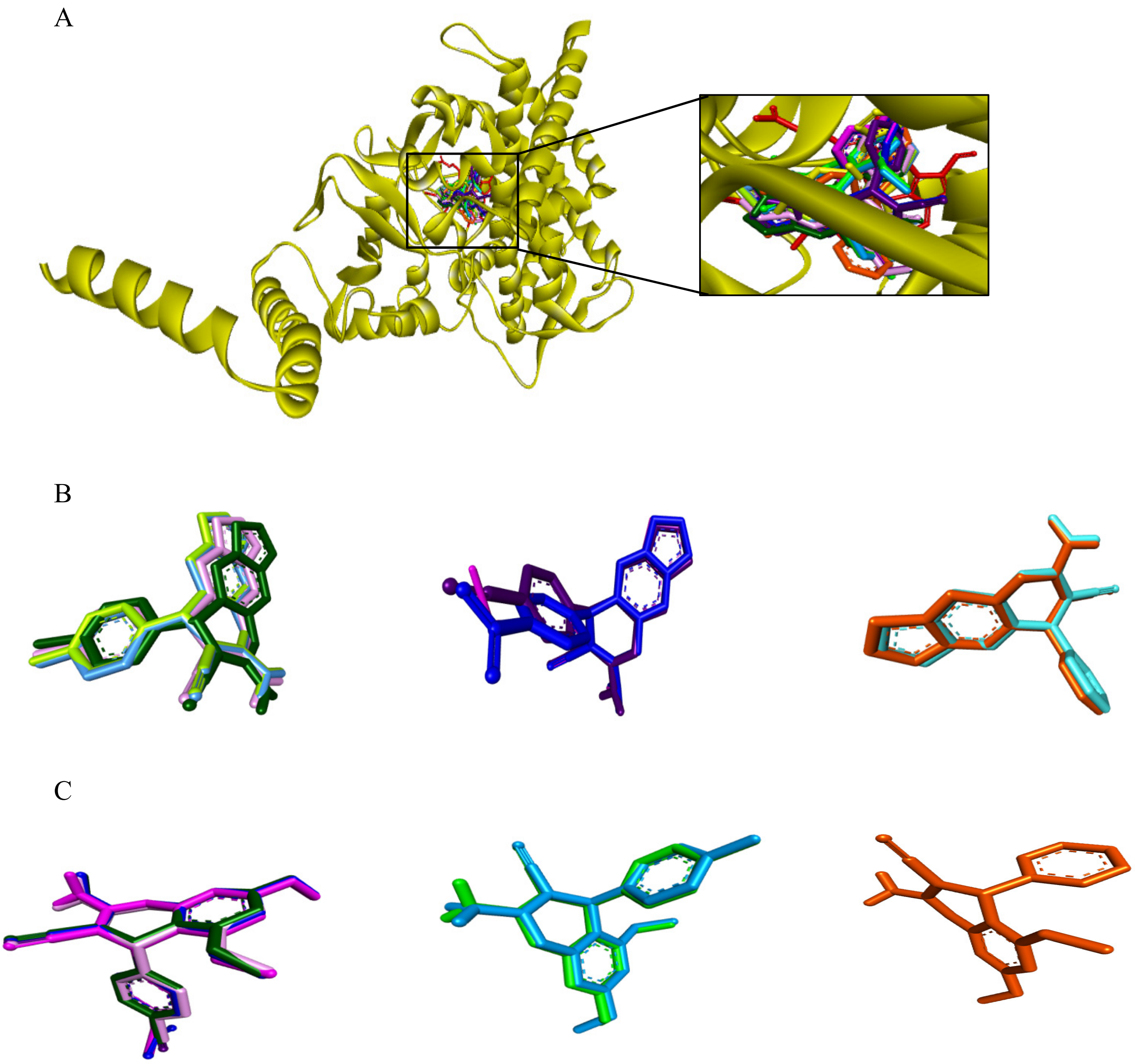

Figure S151. Figure A: Mode of binding and orientation of 2-amino-3-cyano-4-aryl-6,7-methylendioxy-4*H*-chromenes **4a-i** and 2-amino-3-cyano-5,7-dimethoxy-4-aryl-4*H*-chromenes **6a-h** and reference compound fluconazole **8** at the active site of CYP51CA. Figures B and C shows the orientation compounds **4a-i** and **6a-h** respectively at the active site of CYP51CA. Fluconazole (yellow), compounds; H (orange), F (light blue), Cl (pink), Br (dark green), CN (purple), NO_2 (dark blue), Me (light green), MeO (dark pink) and Py (Cyan). Heme group is in red.

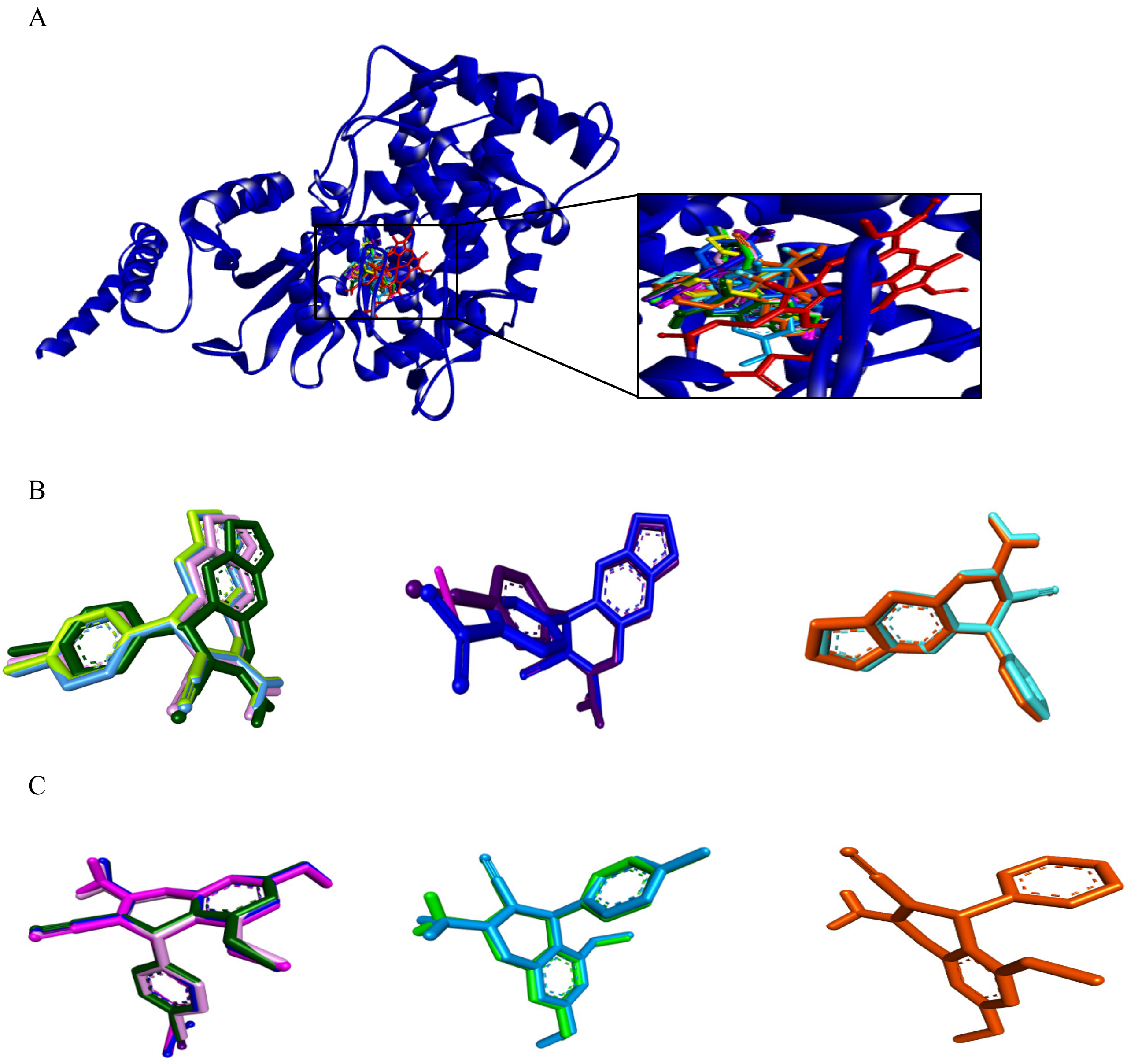

Figure S152. Figure A: Mode of binding and orientation of 2-amino-3-cyano-4-aryl-6,7-methylendioxy-4*H*-chromenes **4a-i** and 2-amino-3-cyano-5,7-dimethoxy-4-aryl-4*H*-chromenes **6a-h** and the reference compound fluconazole **8** at the active site of CYP51CD. Figures B and C shows the orientation compounds **4a-i** and **6a-h** respectively in the active site of CYP51CD. Fluconazole (yellow), compounds; H (orange), F (light blue), Cl (pink), Br (dark green), CN (purple), NO_2 (dark blue), Me (light green), MeO (dark pink) and Py (Cyan). Heme group is in red.

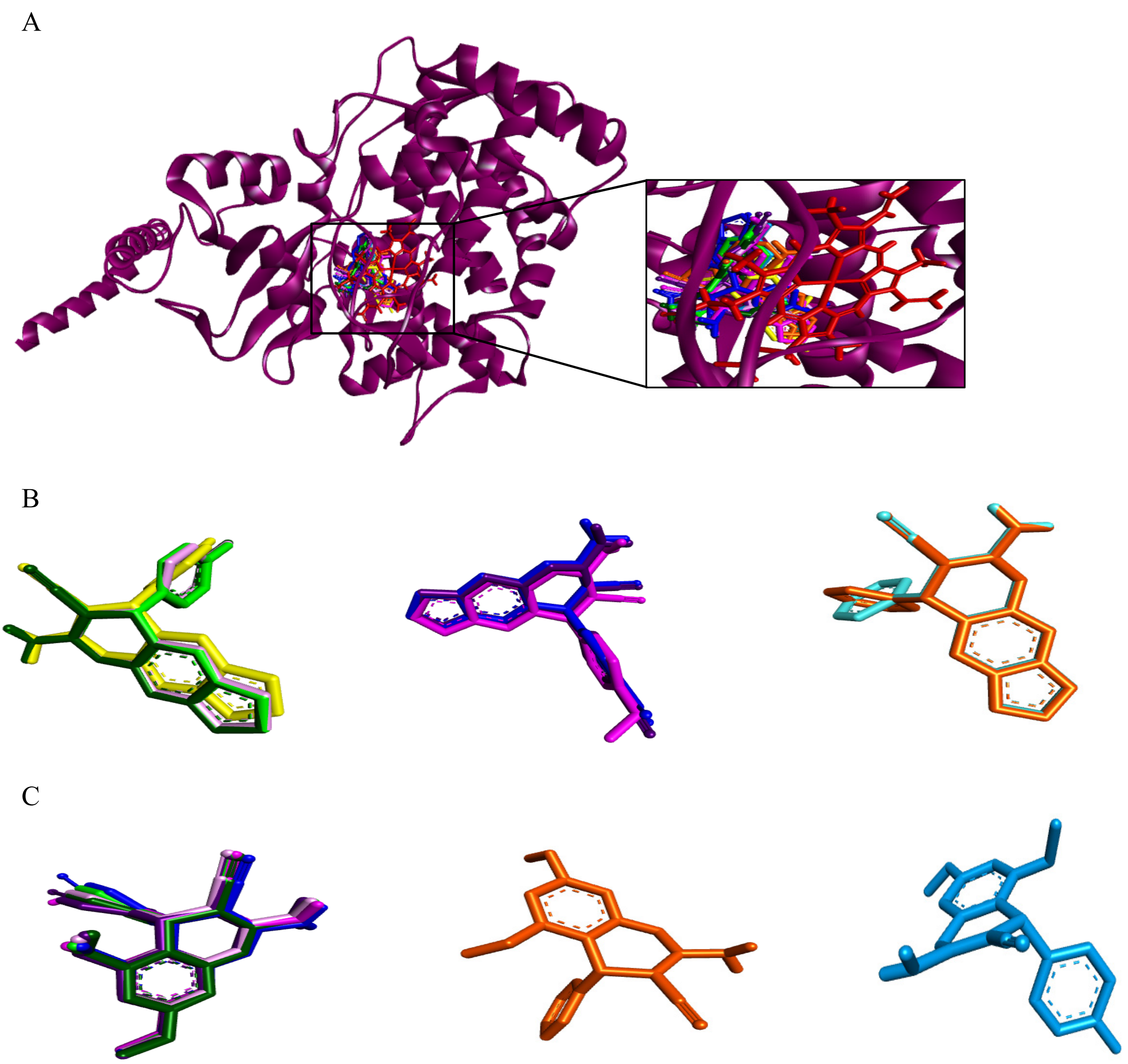

Figure S153. Figure A: Mode of binding and orientation of 2-amino-3-cyano-4-aryl-6,7-methylendioxy-4*H*-chromenes **4a-i** and 2-amino-3-cyano-5,7-dimethoxy-4-aryl-4*H*-chromenes **6a-h** and the reference compound fluconazole **8** at the active site of CYP51CG. Figures B and C shows the orientation compounds **4a-i** and **6a-h** respectively in the active site of CYP51CG. Fluconazole (yellow), compounds; H (orange), F (light blue), Cl (pink), Br (dark green), CN (purple), NO_2 (dark blue), Me (light green), MeO (dark pink) and Py (Cyan). Heme group is in red.

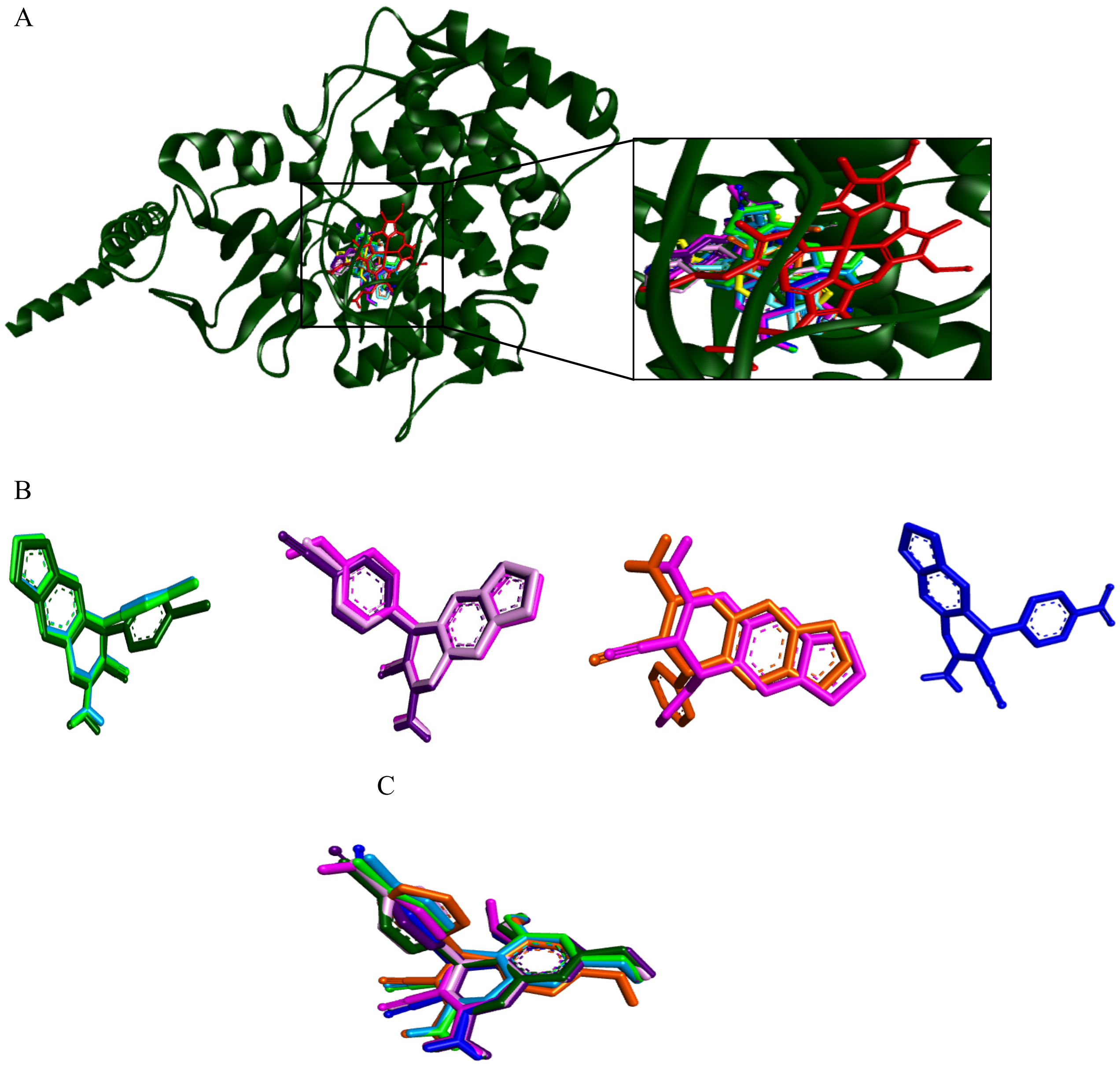

Figure S154. Figure A: Mode of binding and orientation of 2-amino-3-cyano-4-aryl-6,7-methylendioxy-4*H*-chromenes **4a-i** and 2-amino-3-cyano-5,7-dimethoxy-4-aryl-4*H*-chromenes **6a-h** and the reference compound fluconazole **8** at the active site of CYP51CKE. Figures B and C shows the orientation compounds **4a-i** and **6a-h** respectively in the active site of CYP51CKE. Fluconazole (yellow), compounds; H (orange), F (light blue), Cl (pink), Br (dark green), CN (purple), NO_2 (dark blue), Me (light green), MeO (dark pink) and Py (Cyan). Heme group is in red.

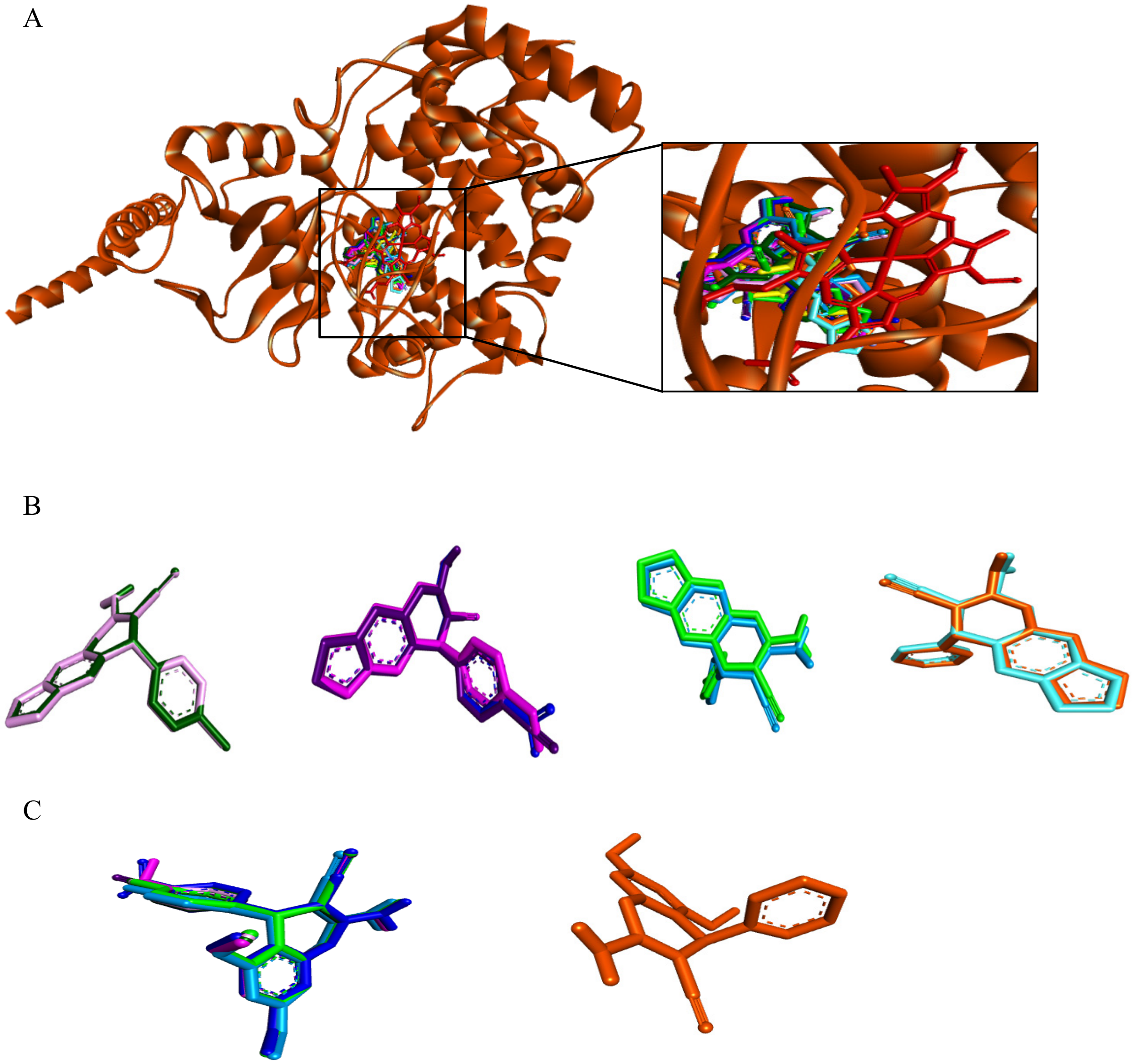

Figure S155. Figure A: Mode of binding and orientation of 2-amino-3-cyano-4-aryl-6,7-methylendioxy-4*H*-chromenes **4a-i** and 2-amino-3-cyano-5,7-dimethoxy-4-aryl-4*H*-chromenes **6a-h** and the reference compound fluconazole **8** at the active site of CYP51CK. Figures B and C shows the orientation compounds **4a-i** and **6a-h** respectively in the active site of CYP51CK. Fluconazole (yellow), compounds; H (orange), F (light blue), Cl (pink), Br (dark green), CN (purple), NO_2 (dark blue), Me (light green), MeO (dark pink) and Py (Cyan). Heme group is in red.

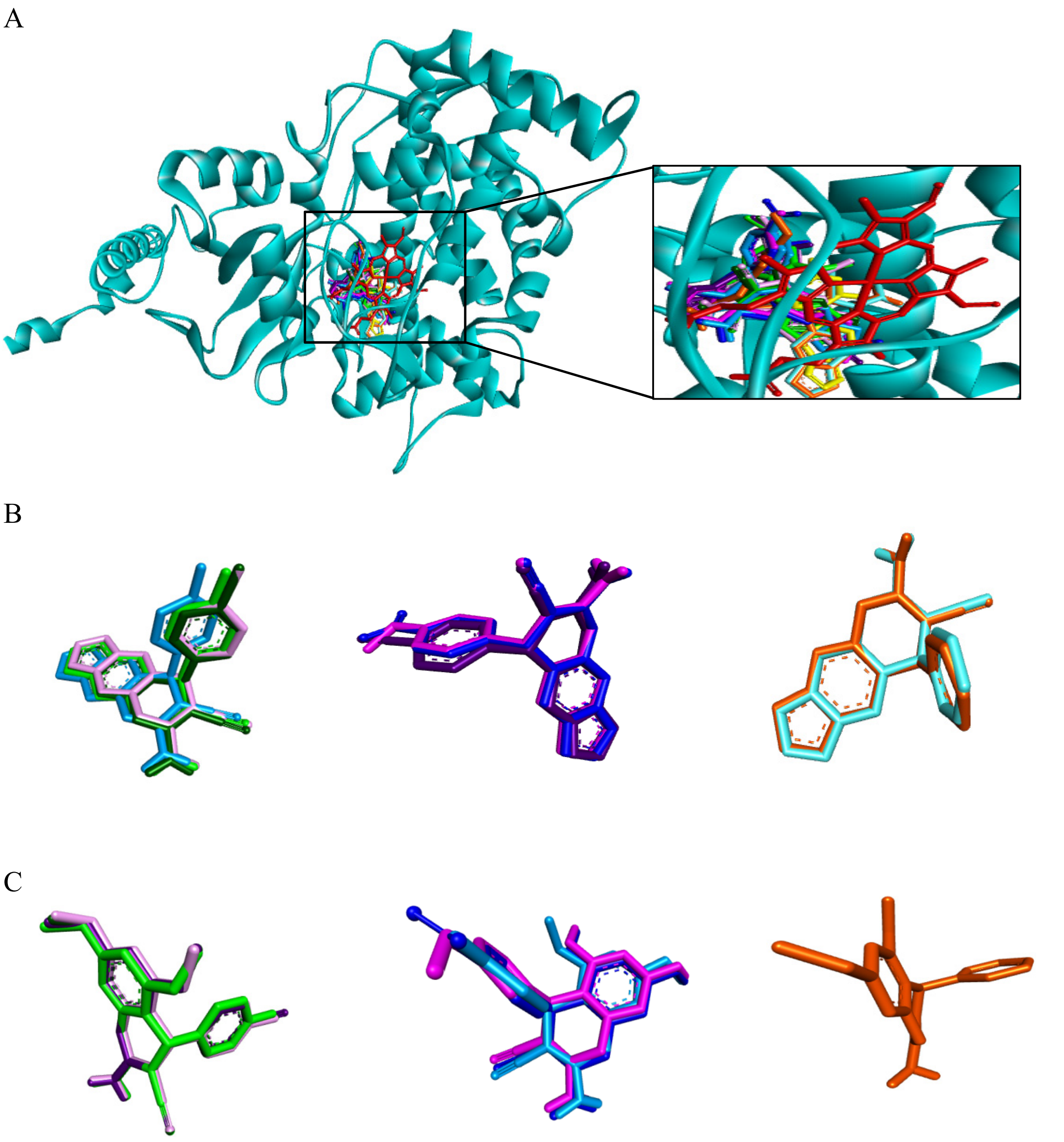

Figure S156. Figure A: Mode of binding and orientation of 2-amino-3-cyano-4-aryl-6,7-methylendioxy-4*H*-chromenes **4a-i** and 2-amino-3-cyano-5,7-dimethoxy-4-aryl-4*H*-chromenes **6a-h** and the reference compound fluconazole (**8**) at the active site of CYP51CP. The mode of binding of the 4*H*-chromene derivatives and reference compound (fluconazole) in CYP51CP is shown in A. Figures B and C shows the orientation compounds **4a-i** and **6a-h** respectively in the active site of CYP51CP. **8** (yellow), compounds; H (orange), F (light blue), Cl (pink), Br (dark green), CN (purple), NO_2 (dark blue), Me (light green), MeO (dark pink) and Py (Cyan). Heme group is in red.

Topotecan (7)

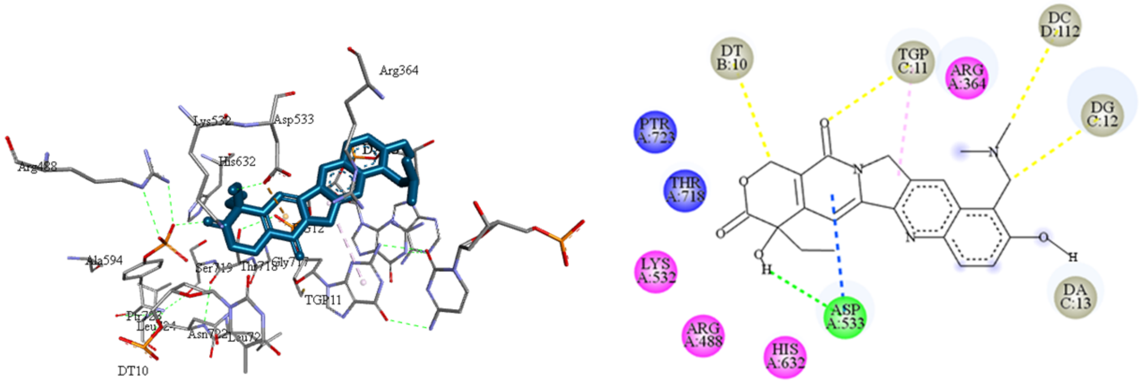

4d

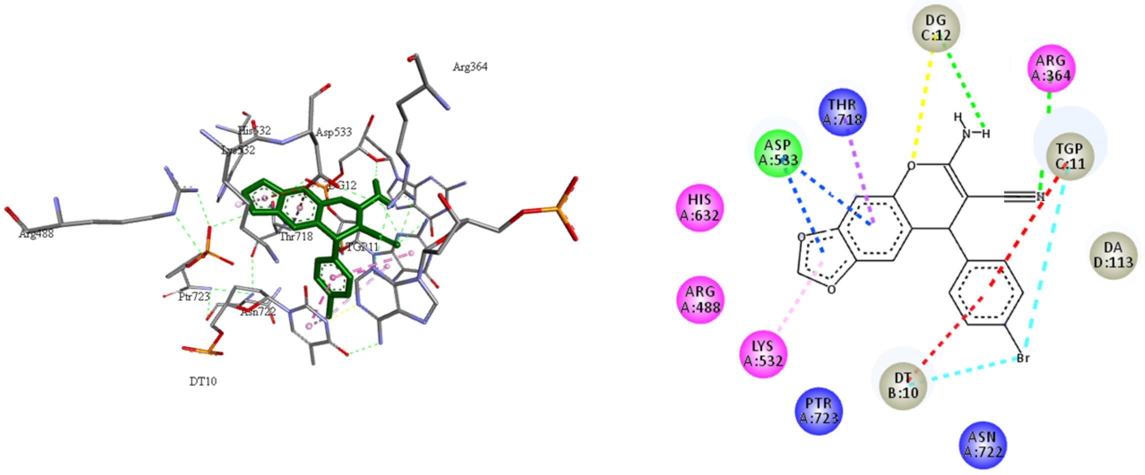

4e

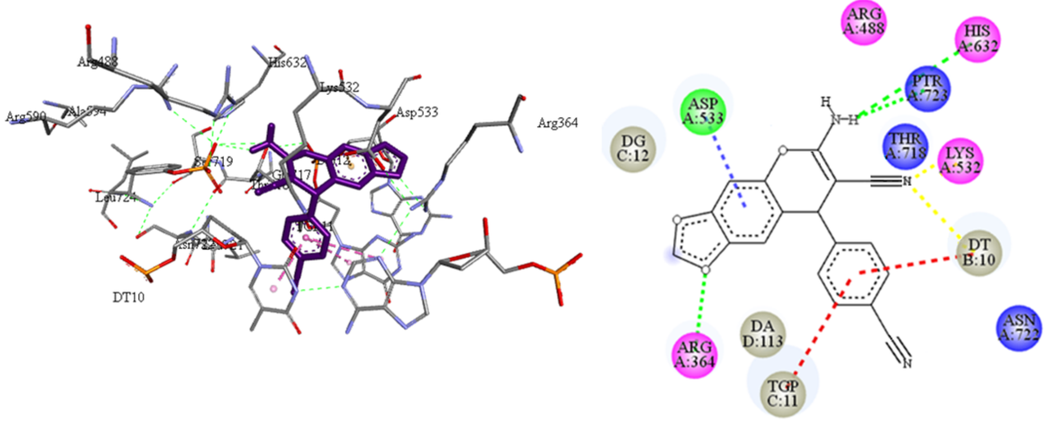
Arg488
His632
Lys532
Asp533
Arg590
Ala594
Arg364
Ser719
Leu724
DT10
ARG A:488
HIS A:632
PTR A:723
ASP A:533
DG C:12
THR A:718
LYS A:532
DT B:10
ASN A:722
DA D:113
ARG A:364
TGP C:11

4f

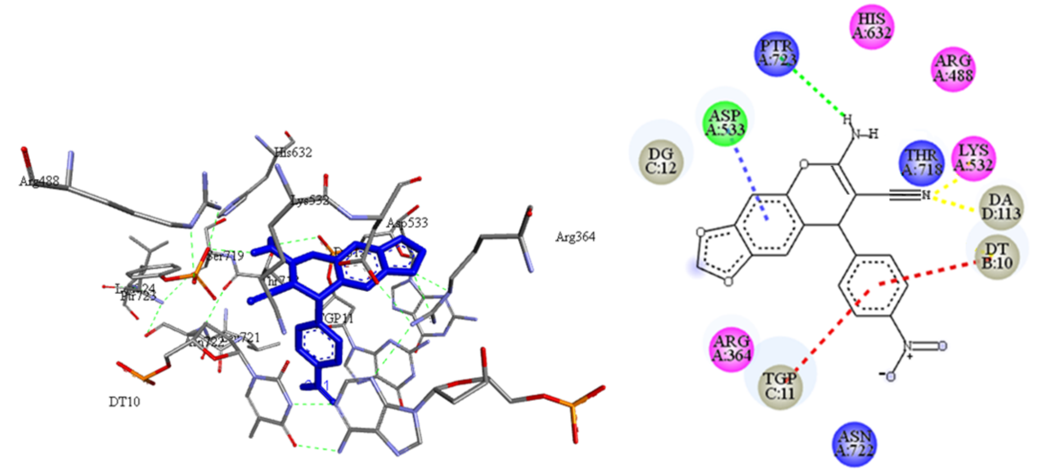
His632
Arg488
Lys532
Asp533
Arg364
Ser719
GP11
DT10
HIS A:632
PTR A:723
ARG A:488
ASP A:533
DG C:12
THR A:718
LYS A:532
DA D:113
DT B:10
ARG A:364
TGP C:11
ASN A:722

4h

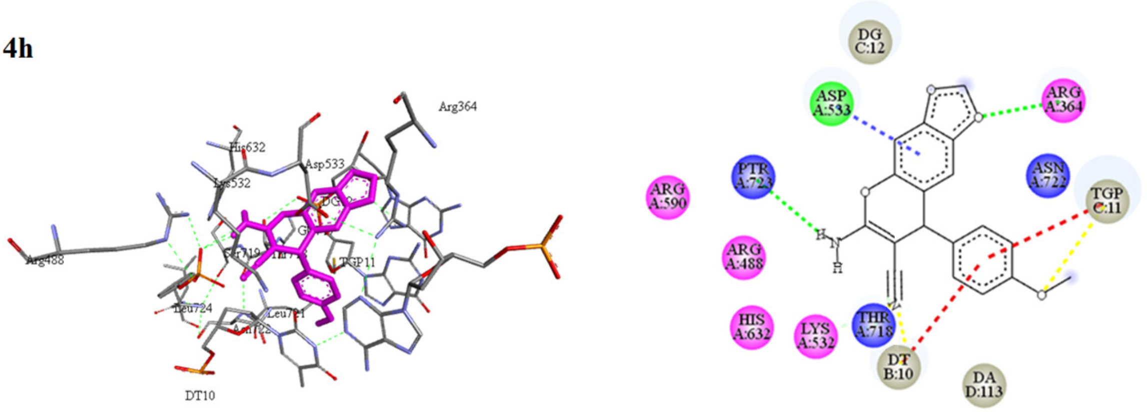
Arg364
His632
Asp533
Lys532
Arg488
Ser719
TGP11
Leu724
Leu721
DT10
DG C:12
ASP A:533
ARG A:364
PTR A:723
ASN A:722
ARG A:590
TGP C:11
ARG A:488
HIS A:632
LYS A:532
THR A:718
DT B:10
DA D:113

4i

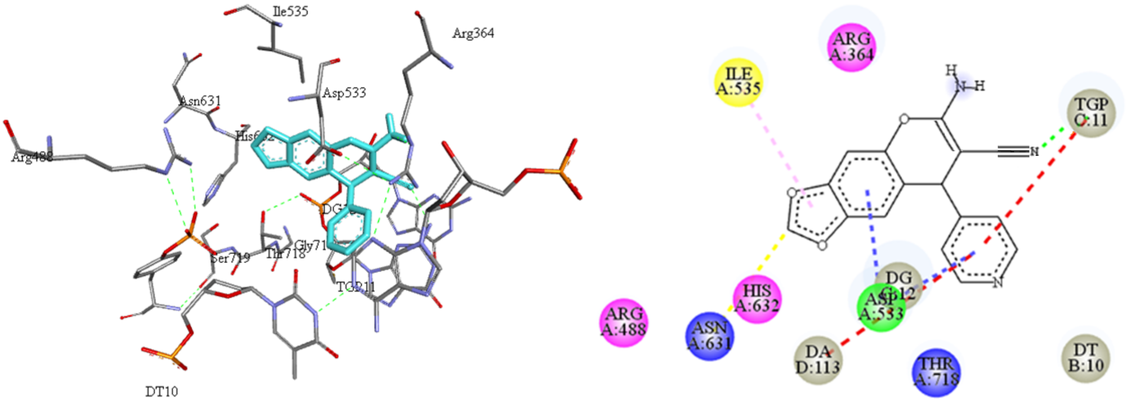

Figure S157. Schematic representation of mode of binding between 2-amino-3-cyano-4-aryl-6,7-methylendioxy-4*H*-chromenes **4d-f** and **4h-i** and the reference compound topotecan **7** at the active site of topoisomerase I. In 3D models, hydrophilic bonds and amino acid residues that are part of the active site of the enzyme are represented. In the 2D model, the following interactions are portrayed with dotted lines: conventional hydrogen bonds (green), carbon-hydrogen (yellow), π-cation (orange), π-anion (blue), π-sigma (purple), π-alkyl (pink), π-π-statcked (red), π-π T-shaped (fuchsia), and halogen (cyan). The solvent accessible surface is illustrated for the amino acid residues and ligands. The amino acids are denoted in pink (basic), green (acidic), yellow (hydrophobic) and blue (hydrophilic).

6a

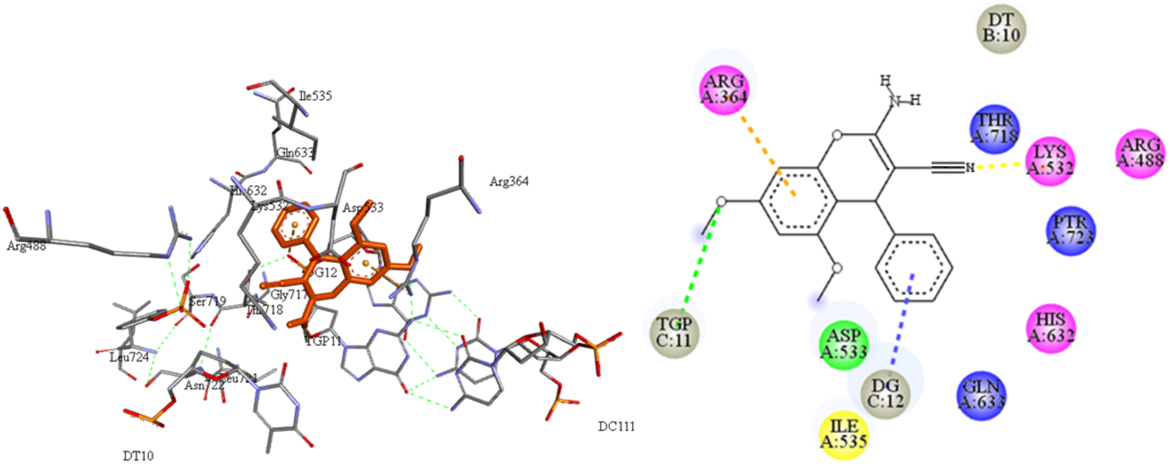

6b

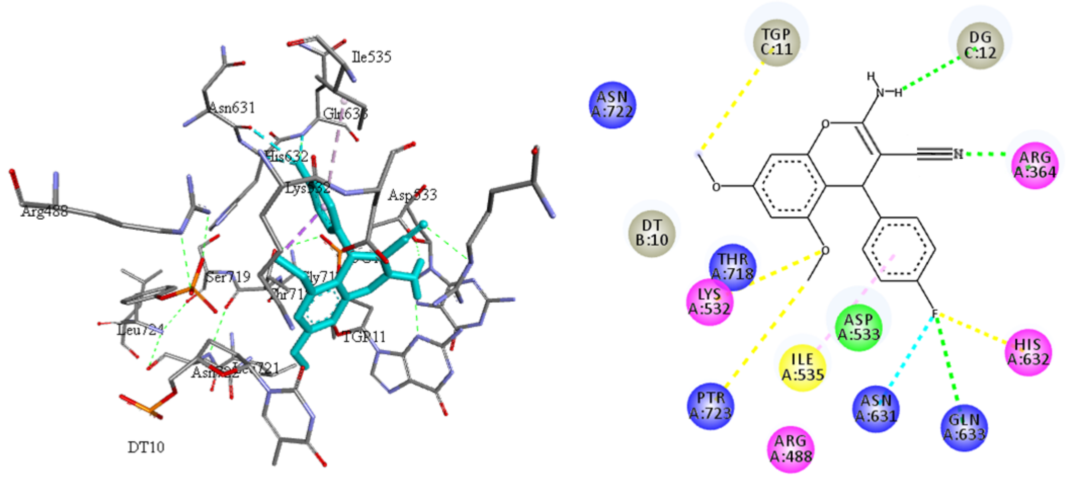
Ile535
Asn631
Gln633
His632
Lys532
Asp533
Arg488
Ser719
Leu724
TGP11
DT10
TGP C:11
DG C:12
ASN A:722
ARG A:364
DT B:10
THR A:718
LYS A:532
ASP A:533
HIS A:632
ILE A:535
PTR A:723
ASN A:631
GLN A:633
ARG A:488

6c

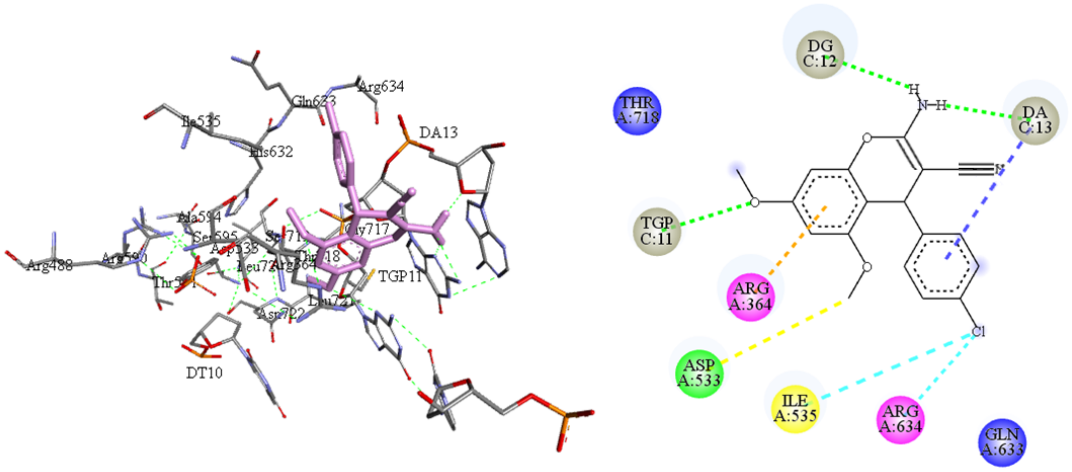
Arg634
Gln633
Ile535
His632
DA13
Gly717
Arg488
TGP11
DT10
Asn722
DG C:12
THR A:718
DA C:13
TGP C:11
ARG A:364
ASP A:533
ILE A:535
ARG A:634
GLN A:633

6d

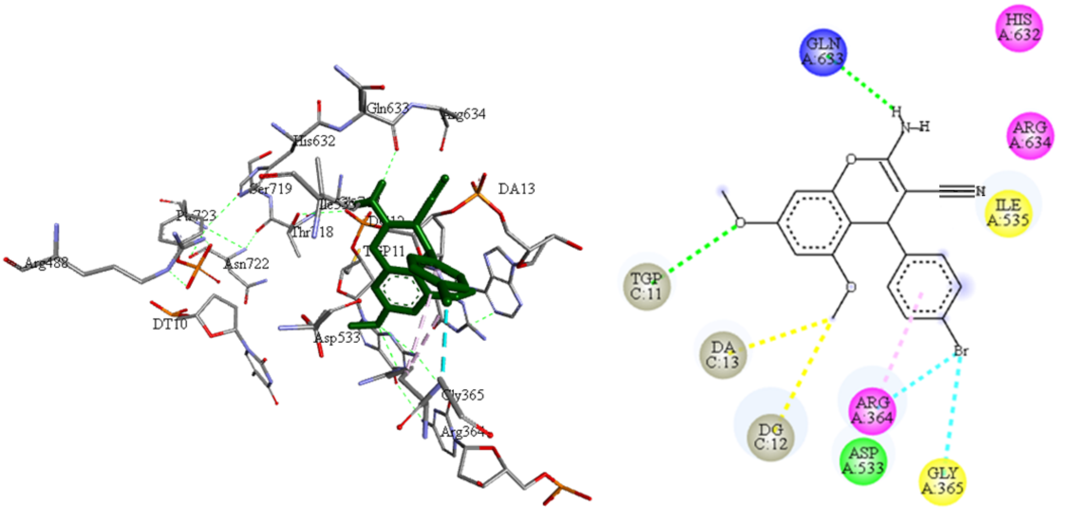
Gln633
Arg634
His632
Ser719
DA13
Arg488
Asn722
DT10
TGP11
Asp533
Gly365
Arg364
HIS A:632
GLN A:633
ARG A:634
ILE A:535
TGP C:11
DA C:13
DG C:12
ARG A:364
ASP A:533
GLY A:365

6e

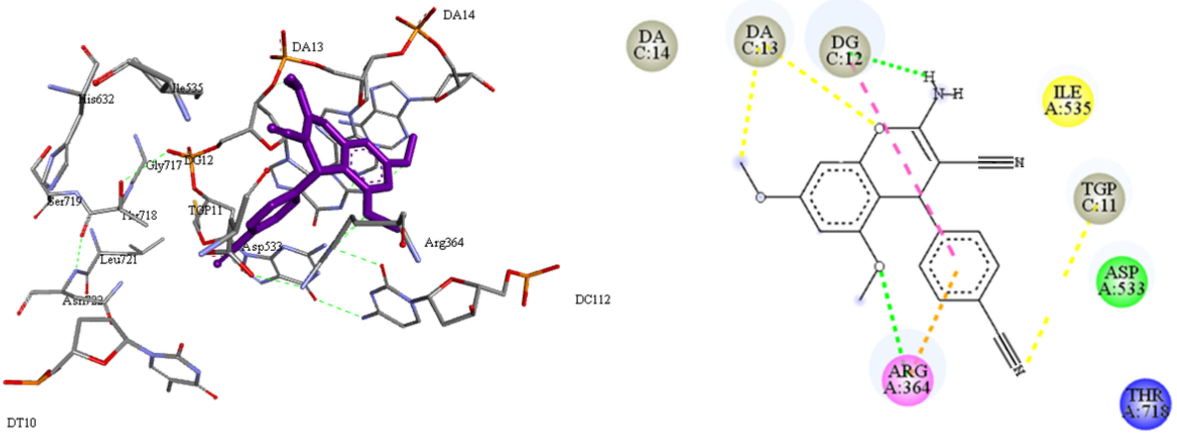
DA14
DA13
Ile535
His632
Gly717
DG12
Ser719
Thr718
TGP11
Asp533
Arg364
Leu721
Asn722
DC112
DT10
DA C:14
DA C:13
DG C:12
ILE A:535
TGP C:11
ASP A:533
ARG A:364
THR A:718

6f

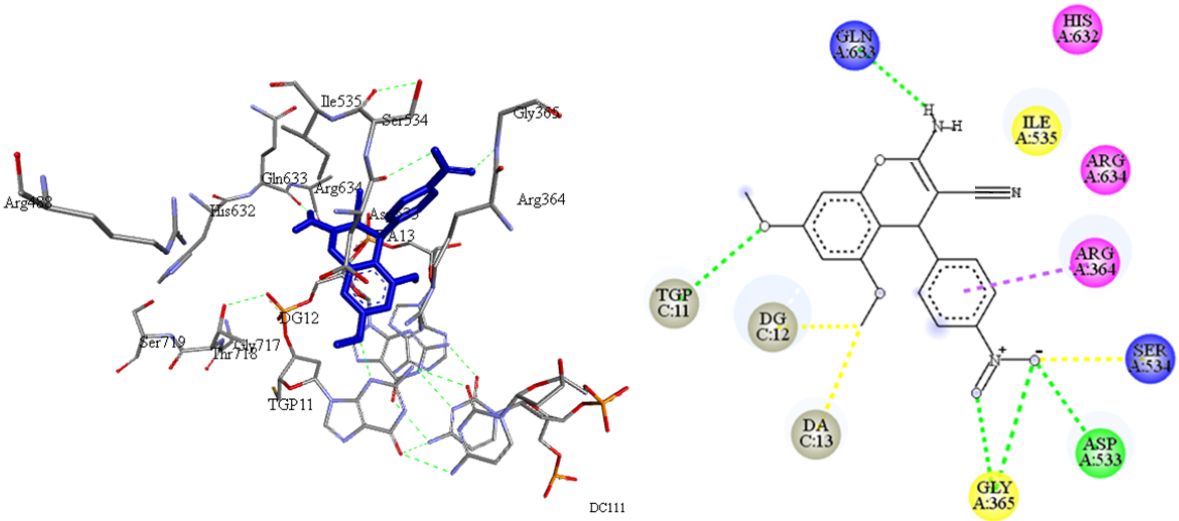
Ile535
Ser534
Gly365
Gln633
Arg634
Arg488
His632
Arg364
A13
DG12
Ser719
Gly717
Thr718
TGP11
DC111
GLN A:633
HIS A:632
ILE A:535
ARG A:634
ARG A:364
TGP C:11
DG C:12
DA C:13
SER A:534
ASP A:533
GLY A:365

6g

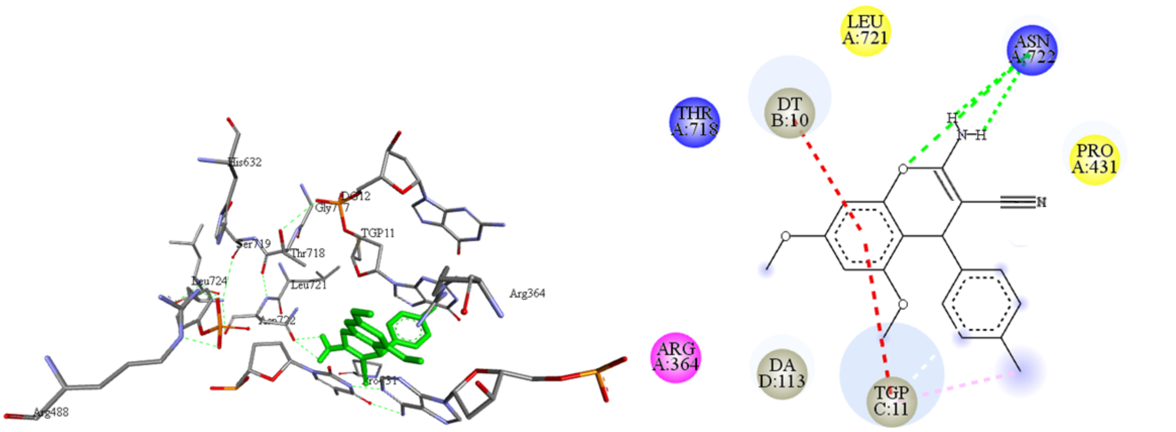
His632
DG12
Gly717
TGP11
Ser719
Thr718
Leu724
Leu721
Arg364
Arg488
LEU A:721
ASN A:722
THR A:718
DT B:10
PRO A:431
ARG A:364
DA D:113
TGP C:11

6h

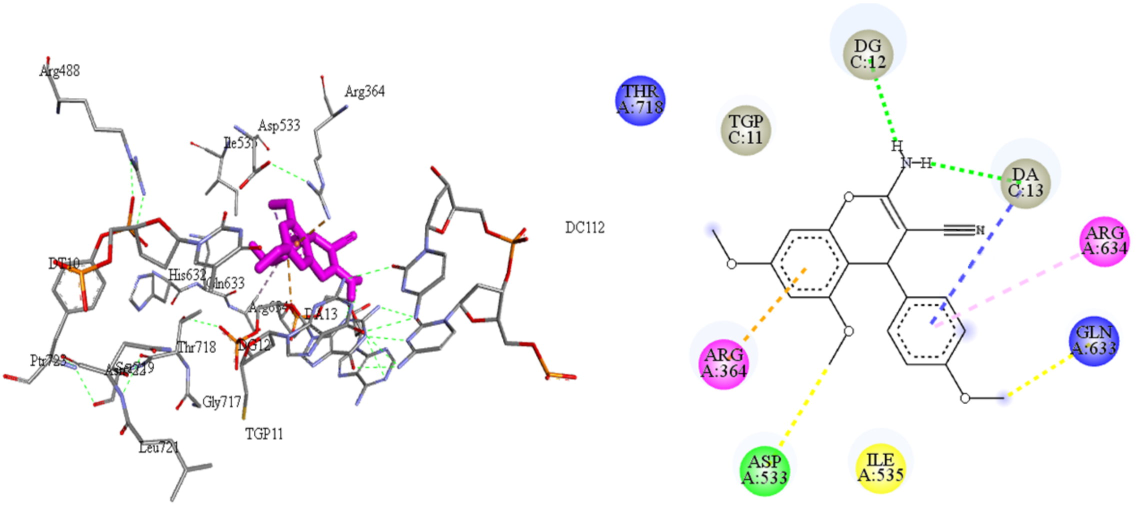

Figure S158. Schematic representation of union mode between 2-amino-3-cyano-5,7-dimethoxy-4-aryl-4*H*-chromenes **6a-i** at the active site of topoisomerase I. In 3D models, hydrophilic bonds and amino acid residues that are part of the active site of the enzyme are represented. In the 2D model, the following interactions are portrayed with dotted lines: conventional hydrogen bonds (green), carbon-hydrogen (yellow), π-cation (orange), π-anion (blue), π-sigma (purple), π-alkyl (pink), π-π-statcked (red), π-π T-shaped (fuchsia), and halogen (cyan). The solvent accessible surface is illustrated for the amino acid residues and ligands. The amino acids are denoted in pink (basic), green (acidic), yellow (hydrophobic) and blue (hydrophilic).

A)

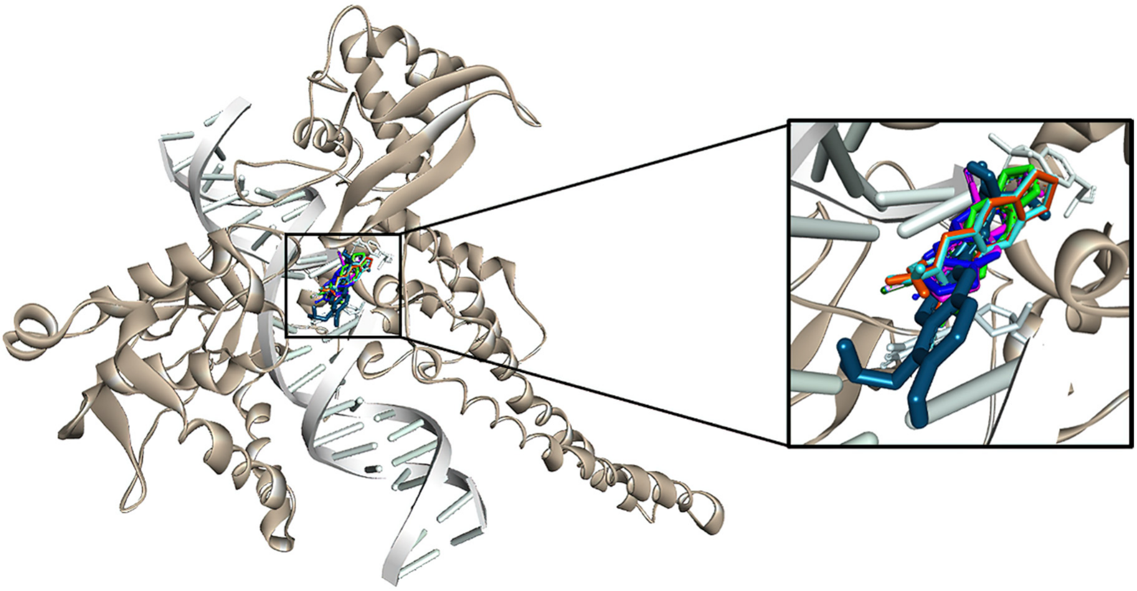

B)

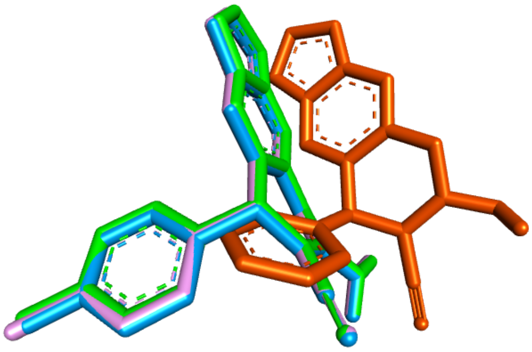

Figure S159. A) Binding mode of **4a-i** and **7** at the active site of topoisomerase I. B) Binding orientation of **4a**, **4b**, **4c** and **4g**. In A and B, the following color codes were used: **4a** (orange), **4b** (light blue), **4c** (pink), **4g** (light green).

A)

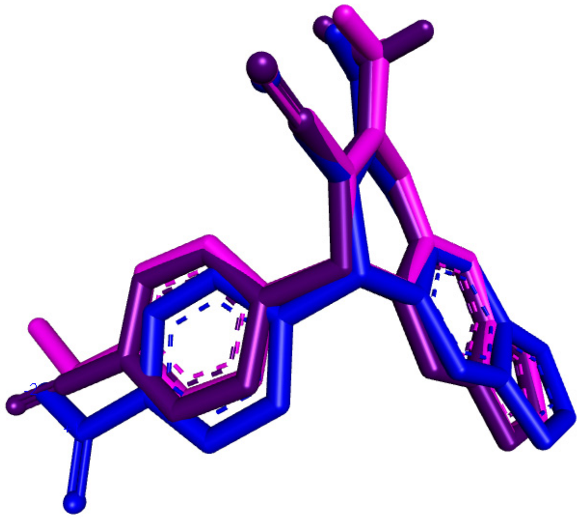

B)

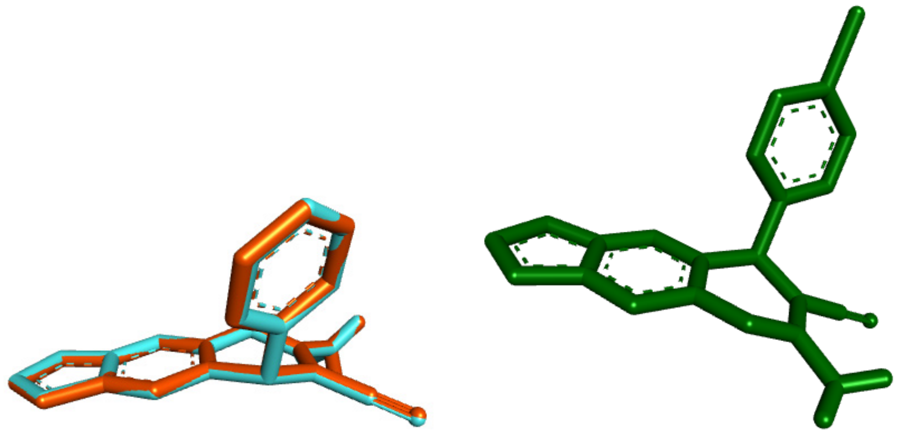

Figure S160. A) Orientation of 2-amino-3-cyano-4-aryl-6,7-methylendioxy-4*H*-chromenes **4e**, **4f**, **4h** and topotecan **7** at the active site of topoisomerase I. B) Orientation of compounds **4d**, **4i** and topotecan at the active site of topoisomerase I. Topotecan (cerulean), compounds; **4d** (dark green), **4e** (purple), **4f** (dark blue), **4h** (dark pink) and **4i** (Cyan).

A)

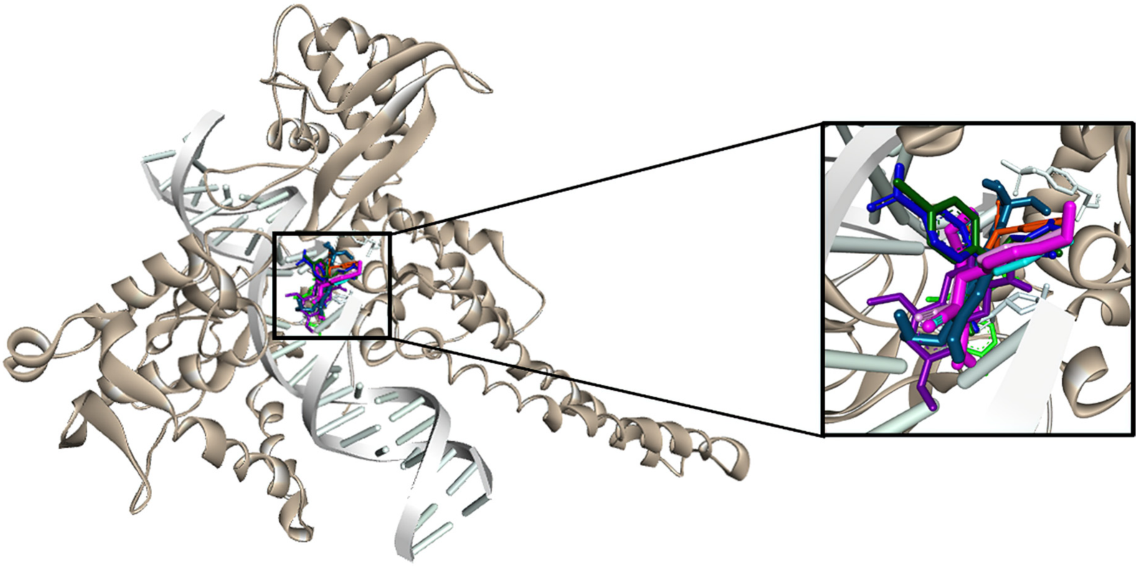

B)

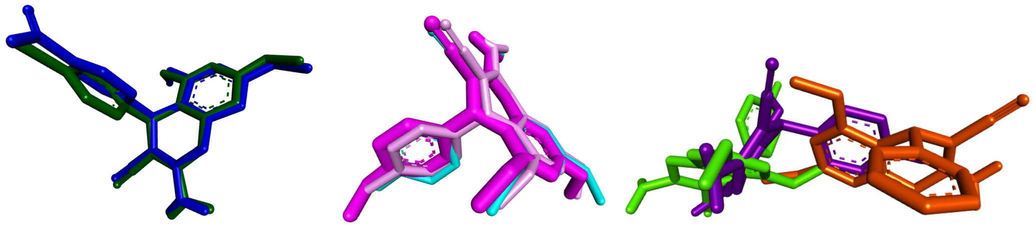

Figure S161: A) Binding mode of topotecan (**7**) and 2-amino-3-cyano-5,7-dimethoxy-4-aryl-4*H*-chromenes **6a-h** at the active site of topoisomerase I, B) Orientations of all compounds. **7** (cerulean), compounds; **6a** (orange), **6b** (light blue), **6c** (pink), **6d** (dark green), **6e** (purple), **6f** (dark blue), **6g** (light green), **6h** (dark pink).

Table S1. Estimated druglikeness properties and compliance of Lipinski rules of 2-amino-3-cyano-4-aryl-6,7-methylendioxy-4*H*-chromenes **4a-o** and the inhibitor of topoisomerase I [topotecan (**7**)].

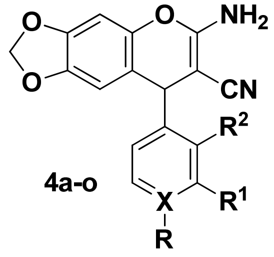

4a-o

Compound	X	R	R^1	R^2	MR	Rotable bonds	Lipinski´s rule of 5	MLog P	Ghose	Egan	Veber	Muegge
**4a**	C	H	H	H	77.93	1	Yes	1.67	Yes	Yes	Yes	Yes
**4b**	C	F	H	H	77.89	1	Yes	2.06	Yes	Yes	Yes	Yes
**4c**	C	Cl	H	H	82.94	1	Yes	2.17	Yes	Yes	Yes	Yes
**4d**	C	Br	H	H	85.63	1	Yes	2.29	Yes	Yes	Yes	Yes
**4e**	C	CN	H	H	82.65	1	Yes	1.02	Yes	Yes	Yes	Yes
**4f**	C	NO_2	H	H	86.75	2	Yes	0.75	Yes	Yes	Yes	Yes
**4g**	C	Me	H	H	82.90	1	Yes	1.91	Yes	Yes	Yes	Yes
**4h**	C	OMe	H	H	84.42	2	Yes	1.36	Yes	Yes	Yes	Yes
**4i**	N	-	H	H	75.73	1	Yes	0.61	Yes	Yes	Yes	Yes
**4j**	C	H	F	H	77.89	1	Yes	2.06	Yes	Yes	Yes	Yes
**4k**	C	H	CN	H	82.65	1	Yes	1.02	Yes	Yes	Yes	Yes
**4l**	C	H	H	F	77.89	1	Yes	2.06	Yes	Yes	Yes	Yes
**4m**	C	H	H	Cl	82.94	1	Yes	2.17	Yes	Yes	Yes	Yes
**4n**	C	H	H	Br	85.63	1	Yes	2.29	Yes	Yes	Yes	Yes
**4o**	C	H	H	NO_2	86.75	2	Yes	0.75	Yes	Yes	Yes	Yes
**Topotecan (7)**	-	-	-	-	114.81	3	Yes	0.98	Yes	Yes	Yes	Yes

Lipinski´s rule of five (MW≤500; MLog P≤4.15; H-A≤ 10; H-D≤5). **Ghose** (160≤MW≤480; -0.4≤WLog P≤5.6; 40≤Molar Refractivity≤130, 20≤atoms≤70). **Egan** (WLog P≤5.88; PSA≤131.6). **Veber** (rotatable bonds ≤ 10, PSA ≤ 140). **Muegge** (200≤MW≤600; -0.2≤XLog P≤5.0; PSA ≤ 150; number of rings ≤ 7; number of carbons > 4; number of heteroatoms > 1; Number of rotable bonds ≤ 15; H-A≤ 10; H-D≤5).

Table S2. Estimated druglikeness properties and compliance of Lipinski rules of 2-amino-3-cyano-5,7-dimethoxy-4-aryl-4*H*-chromenes **6a-h** and the inhibitor of topoisomerase I [topotecan (**7**)].

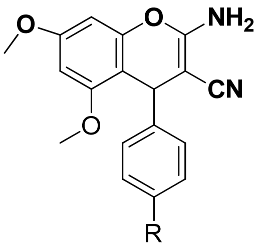

Compound	R	MR	Rotable bonds	Lipinski´s rule of 5	MLog P	Ghose	Egan	Veber	Muegge
**6a**	H	84.85	3	Yes	1.50	Yes	Yes	Yes	Yes
**6b**	F	84.81	3	Yes	1.88	Yes	Yes	Yes	Yes
**6c**	Cl	89.86	3	Yes	2.0	Yes	Yes	Yes	Yes
**6d**	Br	92.55	3	Yes	2.11	Yes	Yes	Yes	Yes
**6e**	CN	89.57	3	Yes	0.84	Yes	Yes	Yes	Yes
**6f**	NO_2	93.68	4	Yes	0.58	Yes	Yes	Yes	Yes
**6g**	Me	89.82	3	Yes	1.73	Yes	Yes	Yes	Yes
**6h**	OMe	91.35	4	Yes	1.19	Yes	Yes	Yes	Yes
**Topotecan (7)**	-	114.81	3	Yes	0.98	Yes	Yes	Yes	Yes

Lipinski´s rule of 5 (MW ≤ 500; MLog P ≤ 4.15; H-A ≤ 10; H-D ≤ 5). **Ghose** (160 ≤ MW ≤ 480; -0.4 ≤ WLog P ≤ 5.6; 40 ≤ Molar Refractivity ≤ 130, 20 ≤ atoms ≤ 70). **Egan** (WLog P ≤ 5.88; PSA ≤ 131.6). **Veber** (rotatable bonds ≤ 10, PSA ≤ 140). **Muegge** (200 ≤ MW ≤ 600; -0.2 ≤ XLog P ≤ 5.0; PSA ≤ 150; number of rings ≤ 7; number of carbons > 4; number of heteroatoms > 1; Number of rotable bonds ≤ 15; H-A ≤ 10; H-D ≤ 5).

Table S3. Pharmacokinetics properties of 2-amino-3-cyano-4-aryl-6,7-methylendioxy-4*H*-chromenes **4a-i** and the inhibitor of topoisomerase I [topotecan (**7**)].

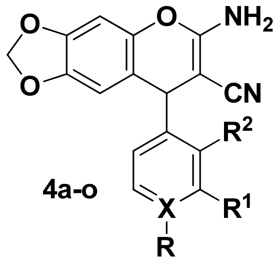

4a-o

Compound	X	R	R^1	R^2	GI absorpt-ion	BBB permeant	P-gp substrate	Log Kp (skin permeation) cm/s	Inhibitor				
									CYP1A2	CYP2C19	CYP2C9	CYP2D6	CYP3A4
**4a**	C	H	H	H	High	Yes	Yes	-5.86	Yes	Yes	Yes	Yes	Yes
**4b**	C	F	H	H	High	Yes	Yes	-5.91	Yes	Yes	Yes	Yes	Yes
**4c**	C	Cl	H	H	High	Yes	Yes	-5.64	Yes	Yes	Yes	Yes	Yes
**4d**	C	Br	H	H	High	Yes	Yes	-5.87	Yes	Yes	Yes	No	Yes
**4e**	C	CN	H	H	High	No	No	-6.23	Yes	Yes	Yes	Yes	Yes
**4f**	C	NO_2	H	H	High	No	Yes	-6.27	Yes	Yes	Yes	Yes	Yes
**4g**	C	Me	H	H	High	Yes	Yes	-5.70	Yes	Yes	Yes	Yes	Yes
**4h**	C	OMe	H	H	High	No	Yes	-6.08	Yes	Yes	Yes	Yes	Yes
**4i**	N	-	H	H	High	No	Yes	-6.64	Yes	Yes	No	Yes	Yes
**4j**	C	H	F	H	High	Yes	Yes	-5.91	Yes	Yes	Yes	Yes	Yes
**4k**	C	H	CN	H	High	No	No	-6.23	Yes	Yes	Yes	Yes	Yes
**4l**	C	H	H	F	High	Yes	Yes	-5.91	Yes	Yes	Yes	Yes	Yes
**4m**	C	H	H	Cl	High	Yes	Yes	-5.64	Yes	Yes	Yes	Yes	Yes
**4n**	C	H	H	Br	High	Yes	Yes	-5.87	Yes	Yes	Yes	No	Yes
**4o**	C	H	H	NO_2	High	No	Yes	-6.27	Yes	Yes	Yes	Yes	Yes
**Topotecan (7)**	-	-	H	H	High	No	Yes	-8.0	No	No	Yes	No	Yes

GI absorption = Gastrointestinal absortion, BBB permeant = Blood-brain barrier permeant, P-gp substrate= P glycoprotein substrate, Kp = skin permeability, CYP1A2 = cytochrome p450 1A2, CYP2C19 = cytochrome p450 2C19, CYP2C9 = cytochrome p450 2C9, CYP2D6= cytochrome p450 2D6, CYP3A4= cytochrome p450 3A4.

Table S4. Pharmacokinetics properties of 2-amino-3-cyano-5,7-dimethoxy-4-aryl-4*H*-chromenes **6a-h** and the inhibitor of topoisomerase I [topotecan (**7**)].

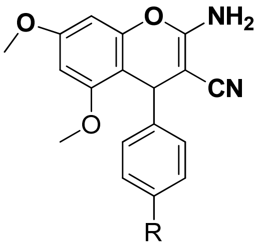

Compound	R	GI absorption	BBB permeant	P-gp substrate	Log Kp (skin permeation)	Inhibitor				
						CYP1A2	CYP2C19	CYP2C9	CYP2D6	CYP3A4
**6a**	H	High	Yes	No	-5.88	Yes	Yes	Yes	Yes	Yes
**6b**	F	High	Yes	No	-5.92	Yes	Yes	Yes	Yes	Yes
**6c**	Cl	High	Yes	No	-5.65	Yes	Yes	Yes	No	Yes
**6d**	Br	High	Yes	No	-5.65	Yes	Yes	Yes	No	Yes
**6e**	CN	High	No	No	-6.24	Yes	Yes	Yes	No	Yes
**6f**	NO_2	High	No	No	-6.28	Yes	Yes	Yes	Yes	Yes
**6g**	Me	High	Yes	No	-5.71	Yes	Yes	Yes	Yes	Yes
**6h**	OMe	High	No	No	-6.08	Yes	Yes	Yes	Yes	Yes
**Topotecan (7)**	-	High	No	Yes	-8.00	No	No	Yes	No	Yes

GI absorption = Gastrointestinal absortion, BBB permeant = Blood-brain barrier permeant, P-gp substrate= P glycoprotein substrate, Kp = skin permeability, CYP1A2 = cytochrome p450 1A2, CYP2C19 = cytochrome p450 2C19, CYP2C9 = cytochrome p450 2C9, CYP2D6= cytochrome p450 2D6, CYP3A4= cytochrome p450 3A4.

Table S5. Estimated toxicity risk of 2-amino-3-cyano-4-aryl-6,7-methylendioxy-4*H*-chromenes **4a-i** and the inhibitor of topoisomerase I [topotecan (**7**)].

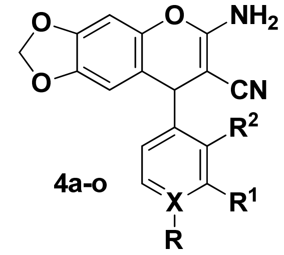

Compound	X	R	R^1	R^2	Tumorigenic	Mutagenic	Reproductive effects	Irritant
**4a**	C	H	H	H	1	1	1	1
**4b**	C	F	H	H	1	1	1	1
**4c**	C	Cl	H	H	1	1	1	1
**4d**	C	Br	H	H	1	1	1	1
**4e**	C	CN	H	H	1	1	1	1
**4f**	C	NO_2	H	H	1	1	1	1
**4g**	C	Me	H	H	1	1	1	1
**4h**	C	OMe	H	H	1	1	3	1
**4i**	N	-	H	H	1	1	1	1
**4j**	C	H	F	H	1	1	1	1
**4k**	C	H	CN	H	1	1	1	1
**4l**	C	H	H	F	1	1	1	1
**4m**	C	H	H	Cl	1	1	1	1
**4n**	C	H	H	Br	1	1	1	1
**4o**	C	H	H	H	1	1	1	1
**Topotecan (7)**	-	-	-	-	1	1	1	1

Reference values to indicate the risk of toxicity: 1 = none, 2 = low and 3 = high.

Table S6. Estimated toxicity risk of 2-amino-3-cyano-5,7-dimethoxy-4-aryl-4*H*-chromenes **6a-h** and the inhibitor of topoisomerase I [topotecan (**7**)].

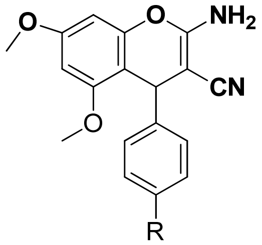

Compound	R	Tumorigenic	Mutagenic	Reproductive effects	Irritant
**6a**	H	1	1	1	1
**6b**	F	1	1	1	1
**6c**	Cl	1	1	1	1
**6d**	Br	1	1	1	1
**6e**	CN	1	1	1	1
**6f**	NO_2	1	1	1	1
**6g**	Me	1	1	1	1
**6h**	OMe	1	1	3	1
**Topotecan (7)**	-	1	1	1	1

Reference values to indicate the risk of toxicity: 1 = none, 2 = low and 3 = high.

Table S7. The binding energy of **4a-o**, **6a-h** and **8** at the active site of CYP51 of *Candida* spp.

**Compound**	**Binding energy between 2-amino-3-cyano-4*H*-chromenes and the CYP51 of *Candida* spp. (kcal/mol)**					
	CA[a]	CD[a]	CG[a]	CKE[a]	CK[a]	CP[a]
**8**	-4.86	-5.03	-3.94	-4.84	-4.96	-4.73
**4a**	-8.64	-9.62	-9.06	-9.53	-9.14	-10.25
**4b**	-9.12	-9.47	-9.13	-10.03	-9.0	-8.96
**4c**	-9.15	-9.54	-8.75	-9.69	-9.08	-9.53
**4d**	-9.16	-9.84	-9.6	-9.85	-9.1	-9.63
**4e**	-8.58	-9.41	-8.47	-9.37	-8.44	-8.78
**4f**	-8.13	-9.03	-8.05	-8.47	-8.01	-8.80
**4g**	-9.20	-9.72	-9.49	-9.97	-9.15	-9.41
**4h**	-8.3	-9.01	-9.03	-9.0	-8.52	-8.78
**4i**	-8.25	-9.23	-8.66	-9.08	-8.7	-9.87
**4j**	-9.11	-9.45	-9.07	-9.94	**-9.94**	-9.3
**4k**	-9.29	-10.28	-9.93	-10.28	-9.55	**-10.88**
**4l**	-8.06	-9.69	-9.65	-10.51	-8.73	-9.87
**4m**	**-9.31**	-10.69	**-10.37**	**-10.83**	-9.29	-10.53
**4n**	-9.17	**-10.76**	-10.29	-10.76	-9.43	-10.51
**4o**	-8.38	-9.75	-9.05	-9.61	-9.02	8.95
**6a**	-8.77	**-9.68**	-8.75	**-10.25**	-8.98	-9.72
**6b**	-8.3	-9.01	-8.68	-9.05	-8.39	-9.06
**6c**	-8.92	-9.34	-9.13	-9.26	-8.98	-9.43
**6d**	**-9.09**	-9.03	**-9.23**	-9.50	**-9.21**	-9.7
**6e**	-9.07	-9.35	-8.97	-9.63	-9.14	**-9.73**
**6f**	-8.55	-8.71	-8.64	-9.03	-8.54	-9.2
**6g**	-8.81	-9.42	-9.17	-9.2	-8.82	-9.27
**6h**	-8.61	-9.01	-9.17	-9.22	-8.62	-9.39

[a]**CA (*C.*** *albicans*), CD (*C. dubliniensis*), CG (*C. glabrata*), CKE (*C. kefyr*), CK (*C. krusei*), and CP (*C. parapsilosis*).

Table S8. Results of the main interactions of the 2-amino-3-cyano-4*H*-chromenes and fluconazole (**8**) with best binding energies in CYP51 of *Candida* spp.

Compound	Interacting residues	Interactions	
		Polar	Hydrophobic
CA	Tyr118, Thr122, Phe126, Ile131, Tyr132, Phe228, Gly303, Ile304, Gly307, Gly308, Thr311, Leu376	---	π-π-shaped (Tyr118)<br>π-sigma (Ile131)<br>π-alkyl (Leu376)
**Fluconazole (8)**			
**4g**	Tyr118, Ile131, Tyr132, Phe228, Gly303, Ile304, Gly307, Gly308, His310, Thr311, Leu376, Arg381, Val509	N-H……O (Gly307)<br>N……H-O (Thr311)	π-π shaped (Tyr118)<br>π-alkyl (Ile131)<br>π-alkyl (Ile304)<br>π-alkyl (Leu376)
**6d**	Tyr118, Ile131, Tyr132, Phe228, Gly303, Ile304, Gly307, Gly308, His310, Thr311, Leu376, His468, Met508, Val509	N……H-O (Tyr132)	π-alkyl (Ile304)<br>π-alkyl (Ile131)<br>π-sigma (Leu376)
**CG**	Leu123, Thr124, Phe128, Ile133, Tyr134, Phe230, Leu301, Gly304, Val305, Gly308,		π-π-shaped (Phe230)<br>π-alkyl (Ile133)<br>halogen (Gly304)

**Fluconazole (8)**	Gly309, Thr312, Leu374	---	halogen (Val305)<br>π-alkyl (Val305)<br>halogen (Gly308)
**4g**	Tyr120, Leu123, Thr124, Phe128, Tyr134, Gly304, Val305, Gly308, Thr312, His372, Pro373, Leu374, Arg379, Val506	N-H…..O (Tyr134)	π-alkyl (Val305)<br>π-alkyl (Pro373)<br>π-alkyl (Leu374)<br>π-sigma (Leu374)
**6d**	Tyr120, Phe128, Ile133, Tyr134, Phe230, Gly304, Val305, Gly308, Thr312, Pro373, Leu374, His463, Val506	----	π-alkyl (Phe230)<br>π-alkyl (Pro373)<br>π-alkyl (Leu374)
**CK**<br>**Fluconazole (8)**	Tyr126, Leu129, Thr130, Phe134, Ile139, Tyr140, Phe231, Gly305, Val306, Gly309, Thr313, Leu376, Met510	O-H…..O (Tyr140)	π-alkyl (Ile139)<br>π-alkyl (Val306)
**4g**	Tyr126, Ile139, Tyr140, Phe231, Gly305, Val306, Gly309, His312, Thr313, Leu376, Ile379, Val511	N-H…..O (Tyr140)<br>O-H…..O (Tyr140)<br>O…..H-C (Val306)	π-alkyl (Ile139)<br>π-alkyl (Phe231)<br>π-alkyl (His312)<br>π-alkyl (Leu376)<br>π-alkyl (Val306)

			π-alkyl (Val511)
**6d**	Tyr126, Ile139, Tyr140, Phe231, Gly305, Val306, Gly309, Gly310, His312, Thr313, Leu376, Ile379, His473, Met510, Val511	N-H…..O (Tyr140)<br>C-H…..O (Gly309)	π-alkyl (Ile139)<br>π-alkyl (Val306)<br>π-sigma (Leu376)

CA (*C. albicans*), CG (*C. glabrata*) and CK (*C. krusei)*.

Table S9. Binding energy and interactions of 2-amino-3-cyano-4*H*-chromenes **4e-f, 4h-i** and **6a-h**, and topotecan (**7**) at the active site of topoisomerase I, 1SC7.

Compound	Binding energy ΔG (Kcal/mol)	Interacting residues	Interactions	
			Polar	Hydrophobic
**Topotecan (7)**	-9.56	Arg364, Arg488, Lys532, Asp533, His632, Gly717, Thr718, Ser719, Ptr723. DT10, TGP11, DG12, DC112, DA113.	C-H......O (DT10)<br>O…..H-C (TGP11)<br>C-H…..N (DG12)<br>C-H…..O (DC112)<br>O-H…..O (Asp533)	π-alkyl (TGP11).<br>π-anion (Asp533)
**4e**	-7.72	Arg364, Arg488, Lys532, Asp533, His632, Thr718, Asn722, Ptr723. DT10, TGP11, DG12, DA113.	N…..H-C (DT10)<br>O.....H-N (Arg364)<br>N…..H-C (Lys532)<br>N-H…..N (Hys632)<br>N-H…..O (Ptr723)	π-anion (Asp533)<br>π-π stacked (DT10, TGP11)
**4f**	-7.69	Arg364, Arg488, Lys532, Asp533, His632, Thr718, Asn722, Ptr723. DT10, TGP11, DG12, DA113.	N…..H-C (DT10)<br>N…..H-C (Lys532)<br>N-H…..O (Ptr723)	π-anion (Asp533)<br>π-π stacked (DT10, TGP11)
**4h**	-7.71	Arg364, Arg488, Lys532, Asp533, Arg590, Arg632, Thr718, Asn722, Ptr723, DT10, TGP11, DG12, DA113.	N…..H-C (DT10)<br>O…..H-C (TGP11)<br>O…..H-N (Arg364)<br>N-H…..O (Arg364)	π-anion (Asp533)<br>π-π stacked (DT10, TGP11)
**4i**	-7.18	Arg364, Arg488, Asp533, Ile535, Asn631, His632, Thr718. DT10, TGP11, DG12, DA113.	N…..H-N (TGP11)<br>C-H…..O (Asn631)	π-anion (Asp533)<br>π-π stacked (TGP11, DA113)
**6a**	-9.1	Arg364, Arg488, Lys532, Asp533, Ile535, His632, Gln633, Thr718, Ptr723, DT10, TGP11, DG12	O…...H-N (TGP11)<br>N…...H-C (Lys532)	π-anion (DG12)<br>π-cation (Arg364)
**6b**	-7.74	Arg364, Arg488, Lys532, Asp533, Ile535, Asn631, His632, Gln633, Thr718, Asn722, Ptr723, DT10, TGP11, DG12	C-H…..O (TGP11)<br>N-H…..O (DG12)<br>N…..H-N (Arg364)<br>O…..H-C (Lys532)<br>F…...H-C (His632)	π-alkyl (Ile535)<br>halogen (Asn631)

			F…..H-N (Gln633)<br>O…...H-C (Ptr723)	
**6c**	-8.42	Arg364, Arg488, Asp533, Ile535, Gln633, Gln634, Ptr723, TGP11, DG12, DA13	O…..H-N (TGP11)<br>N…..H-N (DG12)<br>O…..H-N (DA13)<br>C-H…..O (DA13)	π-anion (DA13)<br>π-cation (Arg364)<br>halogen (Ile535, Arg634)
**6d**	-8.64	Arg364, Gly365, Asp533, Ile535, His632, Gln633, Gln634, Ptr723, TGP11, DG12, DA13	N…..H-N (TGP11)<br>O…..H-C (DG12)<br>O…..H-C (DA13)<br>N-H…..O (Gln633)	π-alkyl (Arg364)<br>halogen (Arg364, Gly365)
**6e**	-8.29	Arg364, Asp533, Ile535, Asn631, Thr718, TGP11, DG12, DA13, DA14	N…..H-C (TGP11)<br>N-H…..O (DG12)<br>O…...H-C (DA13)<br>C-H…..O (DA13)<br>O…..H-N (Arg364)	π-cation (Arg364)<br>π- π T shaped (DG12)
**6f**	-7.66	Arg364, Gly365, Asp533, Ser534, Ile535, His632, Gln633, Arg634, TGP11, DG12, DA13	O…...H-N (TGP11)<br>C-H…..N (DG12)<br>C-H…..O (DA13)<br>O…...H-N (Gly365)<br>O…...H-O (Asp533)	π-sigma (Arg364)
**6g**	-7.45	Arg364, Pro431, Thr718, Leu721, Asn722, Ptr723, DT10, TGP11, DA113	O…...H-N (Asn722)<br>N-H…..O (Asn722)	π-alkyl (TGP11)<br>π-π stacked (DT10, TGP11)
**6h**	-7.99	Arg364, Asp533, Ile535, Gln633, Arg634, Thr718, TGP11, DG12, DA13	N-H…..N (DG12)<br>N-H…..N (DA13)<br>C-H…..O (Asp533)<br>C-H…..O (Gln633)	π-alkyl (Arg634)<br>π-anion (DA13)<br>π-cation (Arg364)

Table S10. Binding energy and interactions of the enantiomers 2-amino-3-cyano-6,7-methylendioxy-4*H*-chromenes **4a-c**, **4g** and **4j-o** (*R*) and (*S*) at the active site of topoisomerase I (PDB entry no. 1SC7).

Compound	Binding energy ΔG (Kcal/mol)	Interacting residues
**4a (*R*)**	-7.55	Arg364, Arg488, Lys532, Asp533, ile535, Asn631, His632, Gln633, Thr718.<br>DT10, TGP11, DG12, DA113.
**4a (*S*)**	-9.29	Arg364, Arg488, Lys532, Asp533, Asn631, Thr718, Asn722, Ptr723.<br>DT10, TGP11, DG12, DA113.
**4b (*R*)**	-7.37	Arg364, Arg488, Lys532, Asp533, Ile535, Asn631, His632, Gln633, Thr718.<br>DT10, TGP11, DG12, DA113.
**4b (*S*)**	-9.27	Arg364, Arg488, Lys532, Asp533, His632, Thr718, Asn722, Ptr723.<br>DT10, TGP11, DG12, DA113.
**4c (*R*)**	-7.59	Arg364, Arg488, Lys532, Asp533, Ile535, Asn631, His632, Gln633, Thr718.<br>DT10, TGP11, DG12, DA113.
**4c (*S*)**	-9.87	Arg364, Arg488, Lys532, Asp533, His632, Thr718, Asn722, Ptr723,<br>DT10, TGP11, DG12, DA113.
**4g (*R*)**	-7.77	Arg364, Arg488, Lys532, Asp533, Ile535, Asn631, His632, Gln633, Thr718.<br>DT10, TGP11, DG12, DA113.
**4g (*S*)**	-9.75	Arg364, Arg488, Lys532, Asp533, His632, Thr718, Asn722, Ptr723,<br>DT10, TGP11, DG12, DA113.
**4j (*R*)**	-7.48	Arg364, Arg488, Lys532, Asp533, Ile535, Asn631, His632, Gln633, Thr718.<br>DT10, TGP11, DG12, DA113.
**4j (*S)***	-9.08	Arg364, Arg488, Lys532, Asp533, His632, Thr718, Asn722, Ptr723,<br>DT10, TGP11, DG12, DA113.
**4k (*R*)**	-8.37	Arg364, Arg488, Lys532, Asp533, Ile535, Asn631, His632, Gln633, Thr718.<br>DT10, TGP11, DG12, DA113.
**4k (*S*)**	-10.0	Arg364, Arg488, Lys532, Asp533, His632, Thr718, Asn722, Ptr723,<br>DT10, TGP11, DG12, DA113.
**4l (*R*)**	-8.26	Arg364, Arg488, Lys532, Asp533, Ile535, Asn631, His632, Gln633, Thr718.<br>DT10, TGP11, DG12, DA113.
**4l (*S*)**	-8.7	Arg364, Arg488, Lys532, Asp533, His632, Thr718, Asn722, Ptr723,<br>DT10, TGP11, DG12, DA113.
**4m (*R*)**	-7.59	Arg364, Arg488, Lys532, Asp533, Ile535, Asn631, His632, Gln633, Thr718.

		DT10, TGP11, DG12, DA113.
**4m (*S*)**	-8.22	Arg364, Arg488, Lys532, Asp533, His632, Thr718, Asn722, Ptr723, DT10, TGP11, DG12, DA113.
**4n (*R*)**	-7.89	Arg364, Arg488, Lys532, Asp533, Ile535, Asn631, His632, Gln633, Thr718. DT10, TGP11, DG12, DA113.
**4n (S)**	-8.55	Arg364, Arg488, Lys532, Asp533, His632, Thr718, Asn722, Ptr723, DT10, TGP11, DG12, DA113.
**4o (*R*)**	-7.88	Arg364, Arg488, Lys532, Asp533, Ile535, Asn631, His632, Gln633, Thr718. DT10, TGP11, DG12, DA113.
**4o (*S*)**	-7.66	Arg364, Arg488, Lys532, Asp533, His632, Thr718, Asn722, Ptr723, DT10, TGP11, DG12, DA113.

In the SK-LU-1 cell line (Table S-11), **4a** exhibited greater anticancer activity than the other 2-amino-3-cyano-4*H*-chromenes. Whereas the IC_{50} values for **4a** and **4b** were better than those of the two reference drugs (**7** and **9**), the IC_{50} of **4c** (R = Cl) was slightly higher (6.60 ± 0.44 μM) than that of **9**, while the IC_{50} of **4g** (R = Me) was 7.79 ± 0.5 μM.

Table S11. IC_{50} (μM) concentration for compounds **4a**, **4b**, **4c** and **4g**.[a]

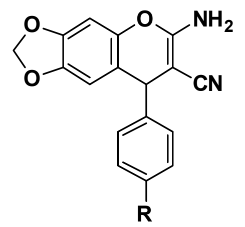

4a,**4b**,**4c** and **4g**

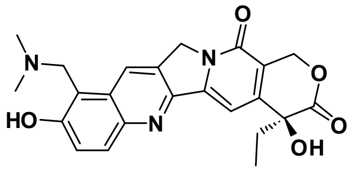

7

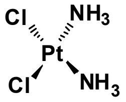

9

Compound (R)	SK-LU-1	PC-3
**4a**	0.52 ± 0.03	1.31 ± 0.12
**4b**	1.26 ± 0.13	2.54 ± 0.30
**4c**	6.60 ± 0.44	25.77 ± 1.70
**4g**	7.79 ± 0.50	27.97 ± 2.10
**9**	4.30 ± 0.50	8.30 ± 0.70
**7**	2.00 ± 0.10	0.40 ± 0.10

[a] Data represent the average of three or four independent assays and are expressed as the mean ± standard error (SE).

In the cytotoxicity assay at 5 µM of all compounds **4a-o** and **6a-h** in the non-cancerous monkey kidney cell line COS-7 (Table S-12), the most of compounds **4a-i** and **6a-h** were not cytotoxic, and only compound 4**f** presented inhibition of 13.5%. For their part, compounds **4j-o** shown cytotoxic activity less than 62.7% in the COS-7 line, however in all cases the inhibition values in the COS-7 line were lower than those observed in human cancer cell line.

Table S12. Cytotoxicity assay results from 2-amino-3-cyano-6,7-methylendioxy-4*H*-chromenes **4a-o** and **6a-h** in COS-7 monkey kidney cell line. Concentration: 5 µM, vehicle: dmso.

Compound	COS-7
**4a**	4.2
**4b**	5.1
**4c**	NC
**4d**	5.7
**4e**	NC
**4f**	13.5
**4g**	12.0
**4h**	NC
**4i**	NC
**4j**	47.5
**4k**	61.2
**4l**	62.7
**4m**	48.4
**4n**	55.3
**4o**	54.9
**6a**	NC
**6b**	NC
**6c**	NC
**6d**	NC
**6e**	NC
**6f**	NC
**6g**	NC
**6h**	NC

NC = No cytotoxic

In the cytotoxicity assay at 25 µM for compounds **4j-o** (Table S-13), compounds **4j-o** showed high cytotoxic activity against the six cancer cell lines. According to (Table S-13) in the cell lines K-562 (human chronic myelogenous leukemia) and SKLU (human lung adenocarcinoma) the compounds showed a 100% inhibition, while in U251 (human glioblastoma), HCT-15 (human colorectal adenocarcinoma), MCF-7 (human mammary adenocarcinoma) the inhibition was higher than 80%. Finally, in PC-3 (human prostatic adenocarcinoma) and MCF-7 (human mammary adenocarcinoma) inhibition percentages greater than 70% were observed.

Table S13. Cytotoxicity assay results in human cancer cell lines from 2-amino-3-cyano-6,7-methylendioxy-4*H*-chromenes **4j-o**. Concentration: 25 µM, vehicle: dmso.

Structure	Compound	U251	PC-3	K562	HCT-15	MCF-7	SKLU-1
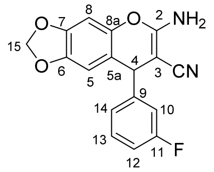	**4j**	84.8	74.9	100	95.2	85.9	100
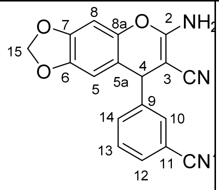	**4k**	91.7	76.7	100	100	82.8	100
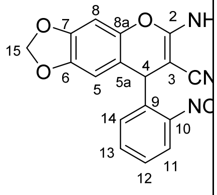	**4l**	91.9	75.7	100	93	96.7	100
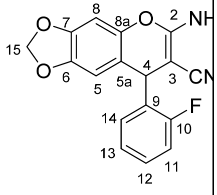	**4m**	81.2	71.2	100	86	72.5	100
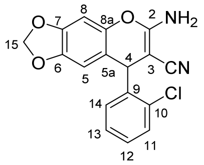	**4n**	82.2	72.9	100	91.4	74.9	100

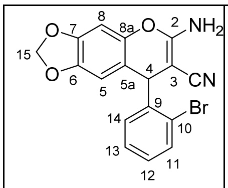	**4o**	80.1	78.5	100	91.9	73.2	100
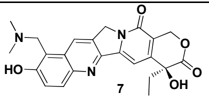	**7**	100	99	97.5	98	100	97.2

U251= human glioblastoma, PC-3= human prostatic adenocarcinoma, K562= human chronic myelogenous leukemia, HCT-15= human colorectal adenocarcinoma, MCF-7= human mammary adenocarcinoma, SKLU= human lung adenocarcinoma.
